# Density Functional Studies on Secondary Amides: Role of Steric Factors in Cis/Trans Isomerization

**DOI:** 10.3390/molecules23102455

**Published:** 2018-09-25

**Authors:** Balmukund S. Thakkar, John Sigurd M. Svendsen, Richard A. Engh

**Affiliations:** Department of Chemistry, UiT The Arctic University of Norway, N-9037 Tromsø, Norway; john-sigurd.svendsen@uit.no

**Keywords:** density functional theory, cis/trans isomerization, secondary amides, dipeptides, steric effects, *tert*-butyl, additivity principle

## Abstract

Cis/trans isomerization of amide bonds is a key step in a wide range of biological and synthetic processes. Occurring through C-N amide bond rotation, it also coincides with the activation of amides in enzymatic hydrolysis. In recently described QM studies of cis/trans isomerization in secondary amides using density functional methods, we highlighted that a peptidic prototype, such as glycylglycine methyl ester, can suitably represent the isomerization and complexities arising out of a larger molecular backbone, and can serve as the primary scaffold for model structures with different substitution patterns in order to assess and compare the steric effect of the substitution patterns. Here, we describe our theoretical assessment of such steric effects using *tert*-butyl as a representative bulky substitution. We analyze the geometries and relative stabilities of both trans and cis isomers, and effects on the cis/trans isomerization barrier. We also use the additivity principle to calculate absolute steric effects with a gradual increase in bulk. The study establishes that bulky substitutions significantly destabilize cis isomers and also increases the isomerization barrier, thereby synergistically hindering the cis/trans isomerization of secondary amides. These results provide a basis for the rationalization of kinetic and thermodynamic properties of peptides with potential applications in synthetic and medicinal chemistry.

## 1. Introduction

The chemistry of the amide bond has attracted the interest of chemists with diverse specializations. Its unique characteristics arise from the delocalization of electrons from nitrogen to the carbonyl group, which confers a partial double-bond character to the C-N bond and stabilizes a planar geometry with a relatively high energy rotational barrier that hinders the free rotation, giving rise to cis and trans isomers [1,2,3]. The resonance effect also protects the amide moiety against nucleophilic attacks at the carbonyl carbon (e.g., it is virtually immune to hydrolysis at ambient temperature and pH in non-enzymatic conditions); hence, it is a common practice to activate amides using Lewis acids for chemical transformation. However, studies on enzymatic hydrolysis of amides have revealed that distortion in the amide bond planarity via C-N bond rotation also results in amide bond activation, increasing susceptibility to nucleophilic attack [4,5,6]. Cis/trans isomerization is one phenomenon whereby the amide moiety loses its planarity, as significant geometric and hybridizational changes occur throughout C-N bond rotation [7]. Therefore, information regarding the stabilities of cis and trans forms of amides, C-N bond rotation in terms of cis/trans isomerization, and relevant energy barriers can be useful for understanding the activation by deformation for a variety of amides, especially peptides. 

While 3°-amides (e.g., prolyl peptide bonds) have often been observed to undergo cis/trans isomerization due to small energy differences between cis and trans isomers [8,9,10], 2°-amides also undergo cis/trans isomerization via higher energy states in diverse important phenomena, such as chemo-mechanical cycling of motor proteins [11], the protein folding [12,13,14] and catalytic activity [15] of enzymes (such as cyclophilin A), cascade dissociation of peptide cation radicals for peptide sequencing [16], and cyclization reactions of peptides (e.g., as in the formation of piperazine-2,5-diones) [17].

**Figure 1:**
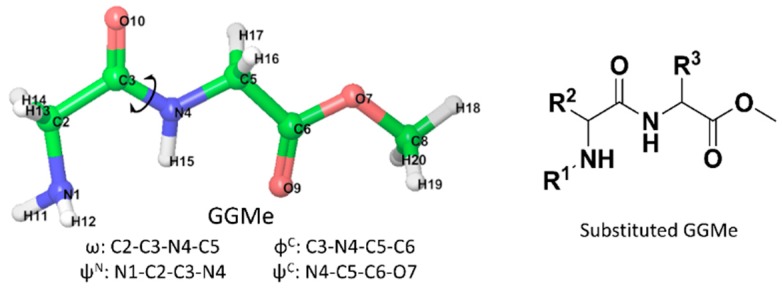
The structures of glycylglycine methyl ester (**left**) and the substituted GGMe scaffold (**right**). The relevant dihedral angles are defined by reference to the GGMe atom names (**left**).

**Figure 2:**
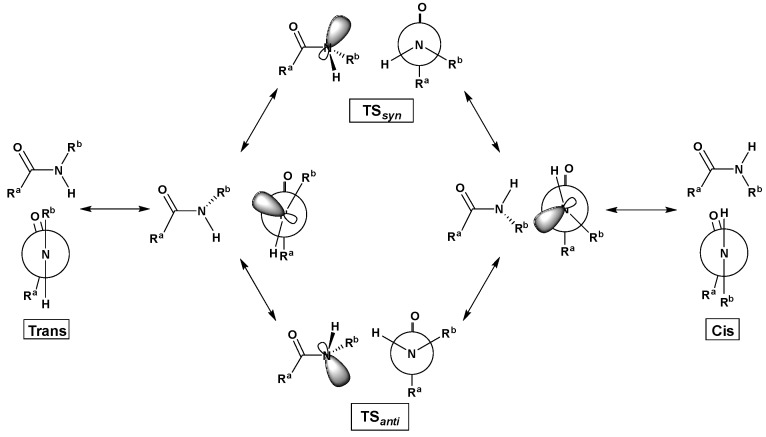
Two paths of cis/trans isomerization via the *syn* transition state (TS*_syn_*) and *anti* transiton state (TS*_anti_*), respectively. R^a^ and R^b^ represent substitutions attached to the carbonyl carbon and amide nitrogen, respectively. For simplicity, the rotation is shown only for positive ω values between 0° (cis) and 180° (trans). See [7] for more details.

With advances in computational capabilities since the 1990s, theoretical studies on trans and cis isomers of 2°-amides and their interconversion have revealed diverse phenomena, such as: effects of pyramidalization of the amide and geometries of transition states [18]; the role of conjugation [19]; simulated solvent effects with molecular dynamics [20]; comparison of theoretically obtained rotational barrier values with experimental values [21,22]; and the generation of ensembles of transition state geometries [23]. Recently, we have conducted theoretical studies [7,24] on secondary amides using density functional methods and molecular dynamics to provide a detailed account of geometry changes during cis/trans isomerization, as well as the effects of solvent models, using glycylglycine methyl ester (GGMe, Figure 1) as an example. We described that cis/trans isomerization can occur via either of the two paths: one via the *anti*-type transition state, and one via the *syn*-type transition state (Figure 2). We also showed that the salient features of the cis/trans isomerization remained consistent when the studies were extended from *N*-methylacetamide to the peptidic scaffolds of GGMe, thus serving as a simple peptide prototype to study conformational flexibilities and complexities relevant to larger molecular backbones. In the present work, we extend our studies to substituted derivatives of GGMe (Figure 1).

It has been observed experimentally [25,26] that the steric bulk on both sides of the amide moiety affects the cis/trans isomerization barrier. This demonstrates the key role of amino-acid side-chains in peptides in the rate of isomerization and extent of equilibria between the two isomers. Corresponding attempts have been made to tailor the flexibility of the rotamers and overall conformations by introducing constraints, such as intramolecular hydrogen bonding and/or steric bulk around the amide bonds [27,28]. In addition to targeted flexibility design, the use of unnatural isomers (especially d-amino acids) and the introduction of *N*-alkylated chains or functionalities in peptide chemistry for the generation of novel peptidomimetics [29,30] provide further variation of the steric bulk around the peptide bond (as well as diversification towards enzymatic activation). Thus, a systematic theoretical study on the effect of steric bulk on the relative stabilities of trans and cis isomers and their interconversion via cis/trans isomerization is in order. 

The diversity of side chains and substitutions, along with their differing extents of steric effects, greatly complicates their study. Bigger and bulkier side chains and substitutions introduce more potential interactions, asymmetry, flexibility, and resonance effects, and hence have prohibitively greater demands for CPU time for QM studies at higher levels of theory. Hence, a systematic study requires representative substitution group(s) that can serve to introduce simple “bulk”, devoid of any resonance or hydrogen-bonding effects. With the A value > 4 [31,32] and υ_ef_ value = 1.2 [33,34], the *tert*-butyl group can therefore be an ideal choice for “bulk”. In synthetic chemistry also, it is a common practice to use *tert*-butyl groups as bulky substitutions to analyze the effect of steric bulk [35,36,37].

With this background, we present our theoretical assessment of the impact of stereospecific patterns of steric bulk on α-carbons and the N-terminal amino group with respect to the geometries of the trans and cis isomers, their relative stabilities, and effects on the cis/trans isomerization barrier.

## 2. Results and Discussion

### 2.1. Model Structures

**Figure 3:**
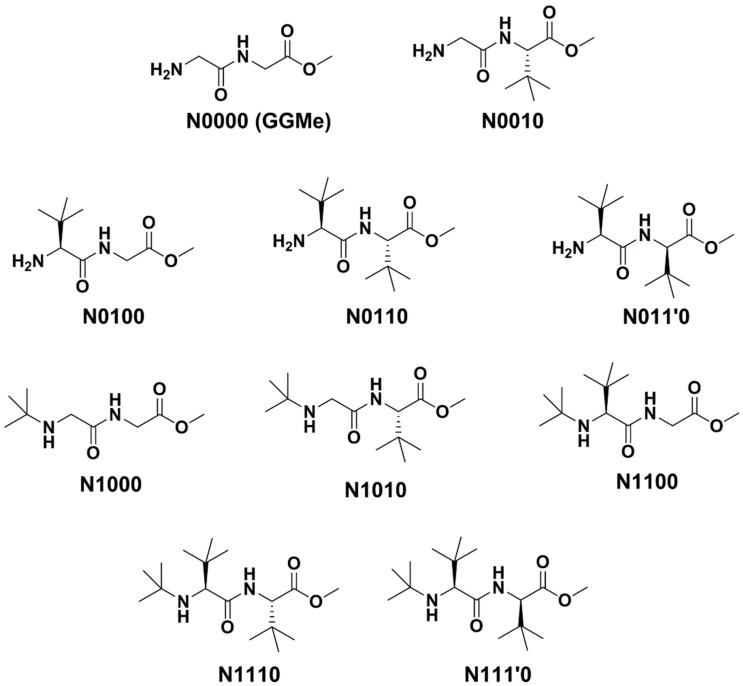
GGMe and its *tert*-butyl-substituted derivatives as model dipeptide esters.

The GGMe structure was taken as the primary scaffold to introduce bulk at different positions. As shown in Figure 1, among the available three positions for substitutions, two are α-carbons of amino acids and are thus chiral centers. In line with the natural amino acids, the default configuration of substitution at α-carbons was kept as the *S*-configuration. However, when both chiral centers had substitutions (i.e., where neither R^2^ nor R^3^ was hydrogen), the R^2^ configuration was kept constant (*S*), and both isomers with different configurations (*R* and *S*) of R^3^ were included. Hence, along with compounds with the natural *S*-configuration, two compounds with unnatural *R*-configurations at the α-carbon were also included. Thus, a total of 10 model structures with different substitution patterns of *tert*-butyl groups were generated, as summarized in Table 1 and Figure 3.

### 2.2. Trans Geometries

**Figure 4:**
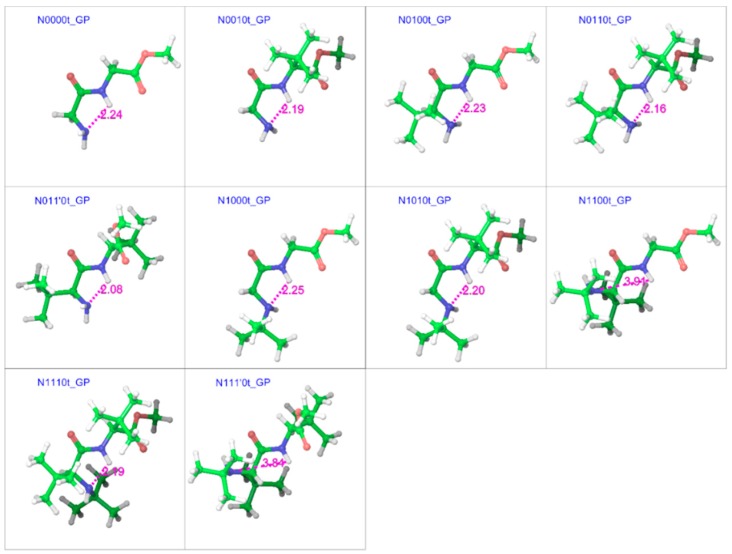
Optimized minimum energy geometries of substituted GGMe derivatives in the gas phase, where all geometries are aligned at the amide moiety. The distance between N-terminal amino nitrogen and amidic hydrogen, in magenta, highlights the presence (or absence when distance > 2.3 Å) of the H-bond between the two atoms. The “t” after the compound name indicates the “trans” isomer.

**Figure 5:**
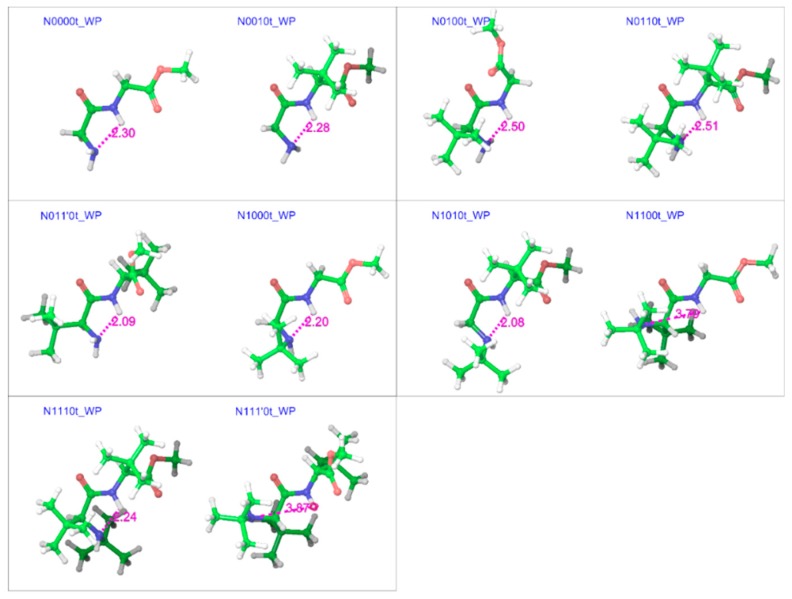
Optimized minimum energy geometries of substituted GGMe derivatives in the water phase, where all geometries are aligned at the amide moiety. The distance between N-terminal amino nitrogen and amidic hydrogen, in magenta, highlights the presence (or absence when distance > 2.3 Å) of the H-bond between the two atoms. The “t” after the compound name indicates the “trans” isomer.

In both gas and water phases (Figure 4 and Figure 5), the optimized minimum energy geometries of trans isomers showed an interesting blend of peptidic features with many of the characteristics previously observed for GGMe [24]. For example, due to the absence of an amide group at the C-terminal, C_7_-forms (γ-foldings) were not observed and the geometries with unsubstituted chiral carbons showed a preference for the extended planar C_5_-form [38]. However, the substitutions at chiral carbons introduced peptidic folding. A majority of geometries also showed the presence of a hydrogen bond between the N-terminal amino group and amidic hydrogen [38]. 

The amide bond planarity is often described quantitatively in terms of the dihedral, ω. The minimum energy geometries showed that the presence of bulky groups on both α-carbons can cause distortion in the amide bond planarity beyond ±5° of ideal perfect planarity (Table 2), and therefore can be used as an alternate approach towards acyclic twisted amides in combination with non-covalent bonding strategies. 

**Figure 6:**
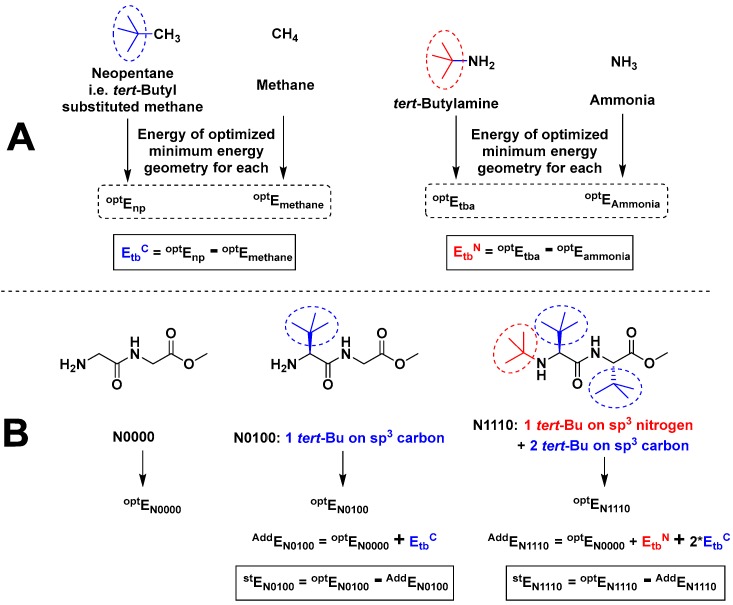
Using the additivity principle: (**A**) Calculation of the additive energy values of *tert*-butyl substitution (**B**) Calculation of the magnitude of the steric effect in terms of energy based on GGMe, explained with examples of N0100 and N1110.

In order to assess the magnitude of steric factors in terms of energy (^st^E), the energy values calculated based on the additivity principle [31] for *tert*-butyl-substituted structures (^Add^E) were compared with their direct energy estimates for their minimum energy geometries (^opt^E). As explained in Figure 6A, the energy difference between methane and neopentane was taken to represent the additive energy value of *tert*-butyl substitution on an sp^3^-carbon (E_tb_^C^), while the energy difference between ammonia and *tert*-butylamine was taken to represent the additive energy value of *tert*-butyl substitution on pyramidal sp^3^-nitrogen of the amino group (E_tb_^N^). Both values were calculated for both gas and water phases (Table 3 and Table 4) each. Then, as shown in Figure 6B, the energy value for the optimized minimum energy geometry of N0000 (GGMe), or ^opt^E_N0000_, was taken as the basis for the calculations of other compounds having *tert*-butyl substitutions on the α-carbon or amino nitrogen.

For each compound, the additive energy value (^Add^E) was determined by the addition of corresponding additive energy values of the *tert*-butyl group (E_tb_), depending on the atom of attachment (carbon or nitrogen), number of *tert*-butyl groups, and the phase (gas or water). The steric effect (^st^E) was calculated as the difference between ^Add^E and the energy values for optimized minimum energy geometries of respective compounds (^opt^E) in their respective phase—that is, the gas or water phases (Table 5 and Table 6).

**Figure 7:**
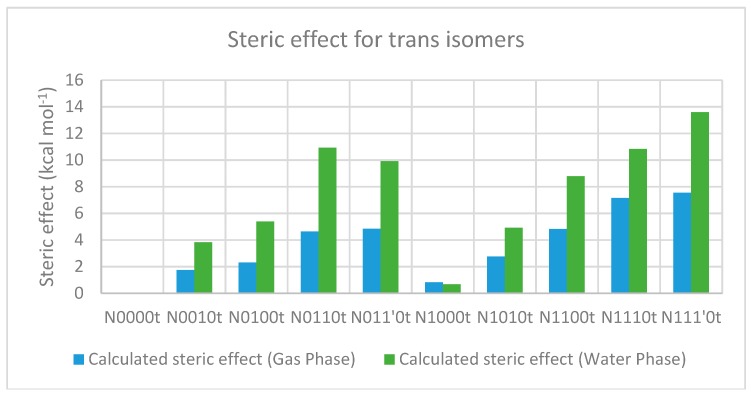
Calculated steric effect for trans-isomers in the gas and water phase, using the additivity principle. The “t” after the compound name indicates the “trans” isomer.

**Figure 8:**
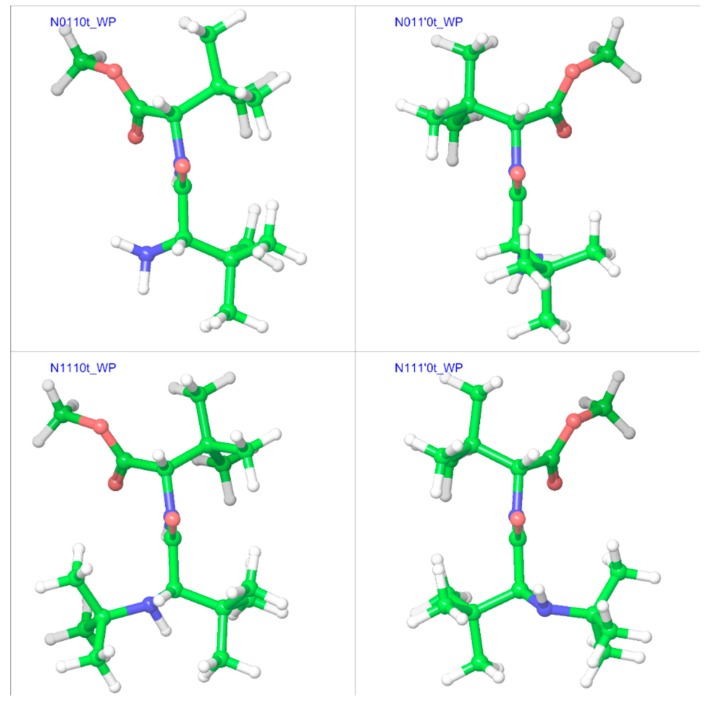
Comparison of minimum energy geometries of N0110, N011′0, N1110, and N111′0 in the water phase. The “t” after the compound name indicates the “trans” isomer.

The trend, as shown in Figure 7, suggests that adding a bulky substitution to an α-carbon results in a considerable steric effect. The bulky substitution on the N-terminal α-carbon has a slightly larger impact than that on the C-terminal α-carbon. A bulky substitution on the N-terminal amino nitrogen also leads to a steric effect that is consistently present across corresponding pairs, becoming stronger with bulk on α-carbons. This steric effect seen in terms of energies is also reflected in strained geometries, and is stronger when using the polarizable continuum model (the water phase). The comparison between different substitution patterns using the additivity principle provides interesting insights about the importance of the hydrogen bond between the N-terminal amino nitrogen and the amide hydrogen for conformational stability. For example, the difference between the stabilities of the minimum energy conformers of N0010 and N0100 in the gas phase is ~0.5 kcal mol^−1^, but with *tert*-butyl substitution on the N-terminal nitrogen (N1010 and N1100), the same stability difference increases to ~2.0 kcal mol^−1^, as N1100 lacks the hydrogen bond (Figure 4). Similarly, in the water phase, the minimum energy geometry of N011’0 is more stable (by ~1 kcal mol^−1^) than that of N0110, while N1110 is more stable (by ~2.7 kcal mol^−1^) than N111’0. Both of the more stable geometries (N011’0 and N1110) show the presence of the hydrogen bond, while both less-stable geometries lack it (Figure 5). Additionally, as is evident from Figure 8, the *tert*-butyl groups in the less-substituted N0110 and N011’0 can remain apart, but the same is not possible for the highly substituted geometries of N1110 and N111’0 with their additional bulk on the N-terminal amino nitrogen, ultimately resulting in the higher energy difference.

### 2.3. Cis Geometries

**Figure 9:**
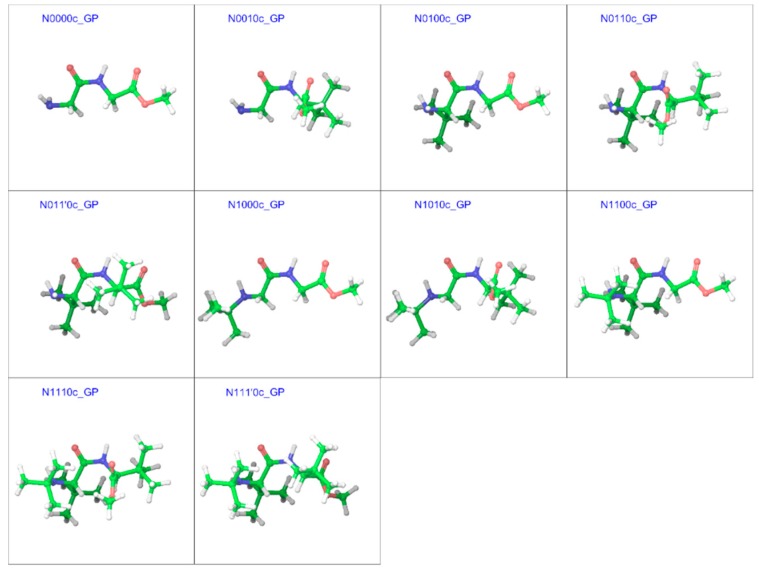
Optimized minimum energy geometries of the cis isomer of the substituted GGMe derivatives in the gas phase; all geometries are aligned at the amide moiety. The “c” after the compound name indicates the “cis” isomer.

**Figure 10:**
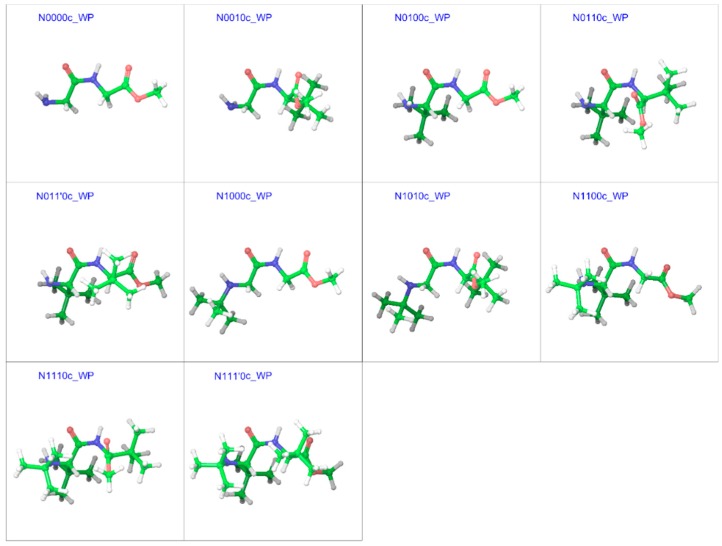
Optimized minimum energy geometries of the cis isomers of the substituted GGMe derivatives in the water dielectric phase; all geometries are aligned at the amide moiety. The “c” after the compound name indicates the “cis” isomer.

**Figure 11:**
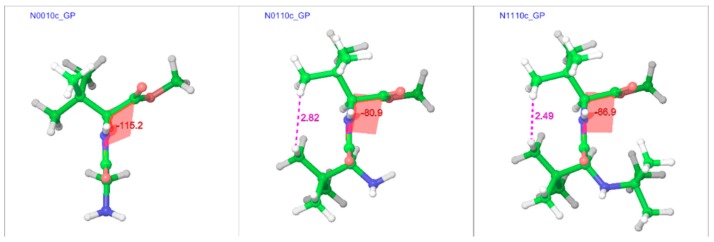
Comparison of cis geometries of N0010, N0110, and N1110 in the gas phase shows increasing strain in the geometries with the successive addition of *tert*-butyl groups. The “c” after the compound name indicates the “cis” isomer.

In line with the trans isomers, the minimum energy geometries of the cis isomers of the compounds in both gas and water phases showed a preference for the extended form at unsubstituted α-carbons, but also showed folding at substituted α-carbons (Figure 9 and Figure 10). With both α-carbons in proximity of each other in the cis-geometries, the impact of steric bulk was evident from the strained geometries. For example, compounds N0010, N0110, and N1110 in the gas phase show similar chain-folding, but the dihedral Φ for the C-terminal residue shows dramatic changes, while the *tert*-butyl groups on both α-carbons are also forced to come closer (Figure 11). With the *tert*-butyl group only on the C-terminal α-carbon, the dihedral Φ for N0010 stands at −115°, which narrows sharply to −81° with the addition of a *tert*-butyl group at the N-terminal α-carbon, as both *tert*-butyl groups try to stay apart (2.82 Å) by pushing the amino and ester moieties towards the other side. However, the repulsion due to another *tert*-butyl group on the amino nitrogen in N1110 counteracts this and broadens the Φ to −87°, in turn “squeezing” the *tert*-butyl groups on both α-carbons even closer (2.49 Å).

#### 2.3.1. The Relative Stabilities of Cis vs. Trans Isomers 

**Figure 12:**
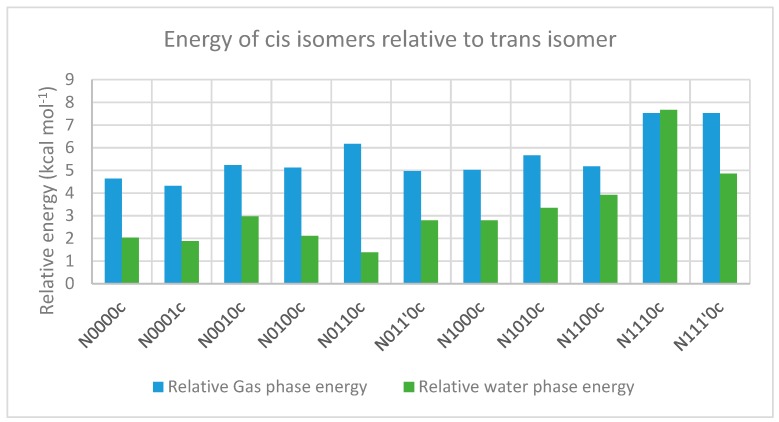
Energies of minimum-energy geometries of cis isomers relative to that of trans-isomers in the gas phase (blue bars) and the water phase (green bars). The “c” after the compound name indicates the “cis” isomer.

**Figure 13:**
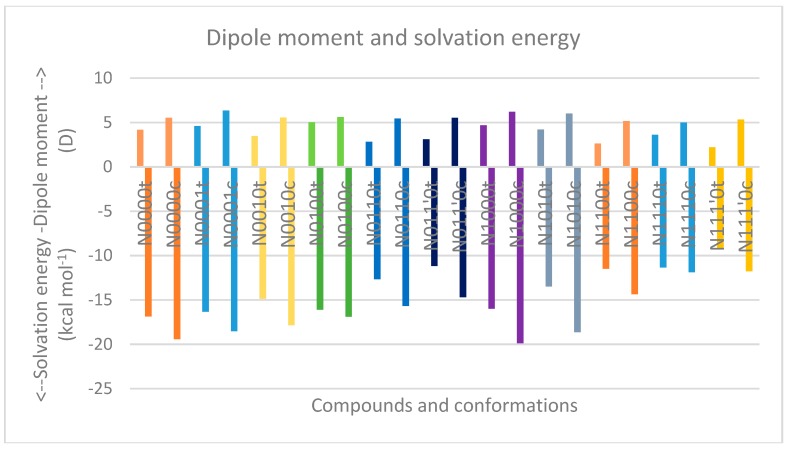
Comparison of the dipole moment and solvation energy for trans and cis isomers (denoted by the “t” and “c” after the compound names, respectively).

Table 7 and Table 8 describe the relative energies of the minimum energy cis geometries in gas and water phases, respectively, along with the corresponding Gibbs free energy change estimates. Interestingly, the relative energy values of the minimum energy geometries of most cis isomers (compared to the minimum energy geometries of the trans isomers) were found to be higher in the gas phase than in the water phase (Figure 12). This may be attributed partially to the fact that the trans isomers in the water phase already have much higher energy values than the corresponding gas-phase structures, as revealed in steric-factor calculations using the additivity principle for the trans isomers. Furthermore, the solvation energy values for most cis isomers are higher (i.e., more negative) than corresponding trans isomers by 2–3 kcal mol^−1^. This indicates better stabilization/solvation of the cis isomers than the trans isomers in the water phase, which may, in turn, be attributed to the higher dipole moment of cis isomers compared to trans isomers, as is evident from Figure 13.

#### 2.3.2. The Effect of Bulk on Cis Isomers 

**Figure 14:**
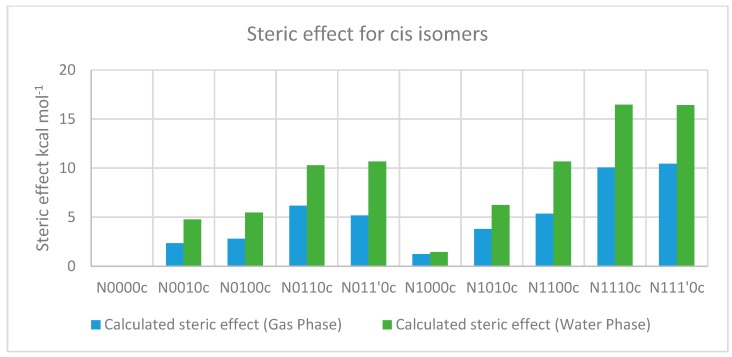
Steric effect for cis-isomers in the gas and water phases using the additivity principle. The “c” after the compound name indicates the “cis” isomer.

Table 9 and Table 10 describe the calculation of steric effects on cis-isomers based on the additivity principle in the gas and water phases respectively, which show a trend similar to that for the trans-isomers (Figure 14), as discussed in Section 2.2. 

### 2.4. Cis/Trans Isomerization Barrier

The calculation of energy barriers for substituted derivatives of GGMe is a complex task. As we have previously reported [7], the existence of multiple rotamers makes it difficult to identify every possible transition state. In the case of GGMe, however, the lack of substitutions on α-carbons still significantly simplifies the calculation, as the isomerization study along the rotation coordinate ω between 180° and 0° suffices due to the symmetry. This becomes more complex with the introduction of substitutions and stereospecificity at α-carbons, which introduces asymmetry along the rotation coordinates, such as the ω-dihedral, resulting in different energy barriers for rotation along the positive vs. negative directions of rotation, with the simultaneous existence of multiple rotamers. Therefore, the number of possible transition-state conformations increases enormously. 

**Figure 15:**
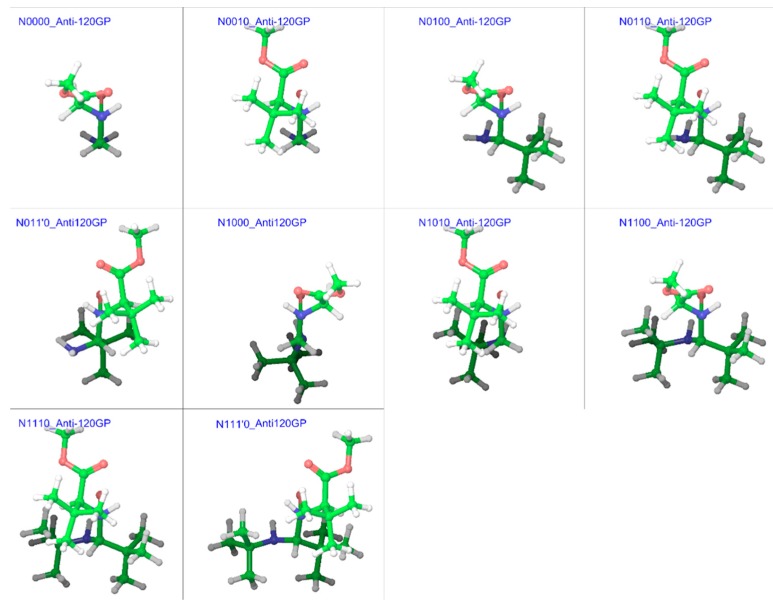
Cis/trans isomerization energy barrier geometries of substituted GGMe derivatives in the gas phase; all geometries are aligned at the amide moiety.

**Figure 16:**
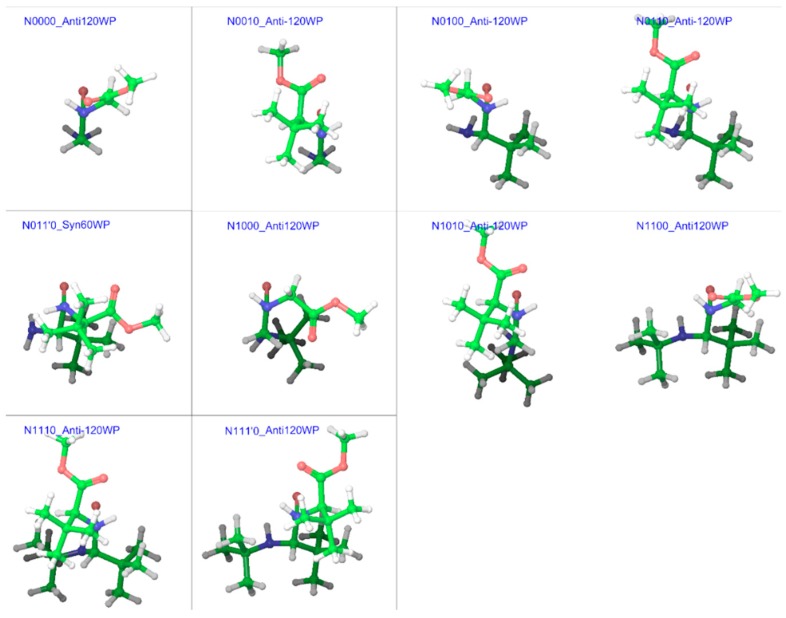
Cis/trans isomerization energy barrier geometries of substituted GGMe derivatives in the water phase; all geometries are aligned at the amide moiety.

In order to simplify this complex problem, it was necessary to use a specific method to enable comparison among same types of geometries for different compounds. Such a comparison can provide information about the overall effect of substitution patterns, if not for specific conformers. From the examples of *N*-methylacetamide and GGMe [7], it became evident that the energy-barrier geometries obtained with stepwise RCS would provide a reasonably accurate estimation of transition states and corresponding energies. Therefore, we decided to find energy-barrier geometries (EBGs) to estimate energy-barrier values. For compounds without substitutions on the α-carbon, the calculation in one direction (between 0° and 180°, or between 180° (=−180°) and 360° (=0°)) was sufficient. However, for other substituted compounds, calculation in both directions was carried out. Correspondingly, from the trans isomers, *syn* EBGs were obtained close to ω = ±60° and from the cis isomers, *anti* EBGs were obtained close to ω = ±120°. Figure 15 and Figure 16 represent the most stable energy barrier geometry for each compound in the gas phase and water phase, respectively. 

Table 11 and Table 12 describe energy-barrier values corresponding to *syn*/*anti* EBGs in the gas phase and water phase, respectively. It is evident that the energy-barrier value strongly depends on the direction of rotation for compounds with substitution on chiral α-carbons, and in such cases, the energy difference between the same types of energy-barrier geometries in two different directions of rotation can be significant, from 1 to 6 kcal mol^−1^.

Assuming the minimum-value energy barrier (G_eff_) to be the “real” barrier, it appears that bulky substitution patterns have no significant effect on the energy barrier values in the gas phase, as even the most substituted compounds N1110 and N111’0 have energy barriers similar to the unsubstituted N0000 (GGMe). However, the effect of stereospecific bulky substitution patterns becomes significant in the water phase, and can show an increase in the energy barrier by up to 4–5 kcal mol^−1^. Diastereomers N0110 and N011’0 (with S- and R- configuation on the C-terminal α-carbon) show a difference of ~5 kcal mol^−1^. A similar difference is seen also in the case of the other pairs of diastereomers, N1110 and N111’0. Thus, the stereochemistry of substitutions has a profound effect on the energy-barrier heights and can provide an opportunity to control the flexibility selectively. Interestingly, a *syn* EBG was found to be more stable than both *anti* EBGs in the case of compound N011’0 in the water phase. 

Overall, bulky substitutions were found to affect cis/trans isomerization in two ways: by making the cis isomer more unstable and thereby shifting the equilibrium towards the trans isomer, and by increasing the reaction barrier height and thereby decreasing the reaction rate. As both effects discourage trans to cis isomerization, compounds with bulky substitutions are expected to take longer and/or require higher temperatures for the same effect. Moreover, as trans to cis isomerization is an essential step in the cyclization of dipeptides to piperazine-2,5-diones, it is certain that slow isomerization would also result in decelerated dipeptide cyclization.

## 3. Methods

### 3.1. Generation of Structures

The relevant structures were initially generated as 2D trans isomers using Marvin Sketch 15.6.8, 2015, (ChemAxon, Budapest, Hungary), and were imported to the Maestro [39] module of the Schrodinger suite (Schrödinger, LLC. New York, NY, USA), from which all further studies were performed. The cis geometries were generated by torsional adjustment of the ω dihedral. 

### 3.2. Conformational Search

All trans and cis geometries were subjected to the MacroModel [40] conformational search algorithm, using the MMFFs force-field. From the conformational search output, the geometries within a potential energy of up to 21 kJ/mol over the minimum energy geometry were kept.

All further studies were performed using the Jaguar [41,42] program of the Schrodinger suite. 

### 3.3. Geometric Optimization

The geometries kept after the conformational search were subjected to geometric optimization at the B3LYP/6-31++G** level with maximum grid density and the “accurate” accuracy level of SCF. Frequency calculation studies were carried out to confirm zero imaginary frequencies for an optimized geometry. For each compound, the most stable geometry (i.e., with the minimum energy) was chosen for further study. Calculations for the water dielectric continuum (water phase) were performed using the same parameters and the additional Poisson Boltzmann Finite (PBF) solvent model. The Cartesian coordinates of the optimized geometries are provided in the supplementary information.

### 3.4. Relaxed Coordinate Scan (RCS)

The optimized minimum energy cis and trans geometries of all compounds were subjected to relaxed coordinate scans (RCS), on the ω dihedral coordinate at the B3LYP/6-31++G** level with maximum grid density and the “accurate” accuracy level of SCF. The optimized geometry at each step was used to generate the starting geometry for the next step. For water-phase calculations, the Poisson Boltzmann Finite (PBF) solvent model was used. Each RCS was performed in three rounds, with a 15° step-size for the first round and a 2° step-size (near the rotation barriers) for the second round. The step-size for the third round (closest to the rotation barrier) was kept at 0.125° and 0.250° for the gas and water phase calculations, respectively. Except for compounds with only hydrogens on both chiral α-carbons, the relaxed coordinate scan was performed in both directions (positive and negative direction, clockwise and anticlockwise). The *syn* and *anti* energy-barrier geometries (EBG) were identified and used as plausible energy-barrier geometries. (Henceforth, EBGs refers to the energy-barrier geometries obtained from RCS). The Cartesian coordinates of the EBGs are provided in the supplementary information.

### 3.5. Single-Point Energy Calculations

Accurate single-point energies were calculated for all optimized and reaction-barrier geometries at the B3LYP/6-311++G(3df,3pd) level with maximum grid density and the “accurate” accuracy level of SCF. Vibrational analyses were carried out at the B3LYP/6-31++G** level, and the free energy values obtained for 298.15 K were used to calculate relative Gibbs free energies. For water phase calculations, the Poisson Boltzmann Finite (PBF) solvent model was used.

## 4. Conclusions

In this work, we conducted a systematic theoretical study of the effects of steric bulk on the relative stabilities with varied substitution patterns on a peptidic scaffold using density functional methods. We used model compounds based on the simple glycylglycine methyl ester peptidic scaffold with patterns of stereospecific substitutions of the *tert*-butyl group to represent steric bulk. The study establishes how bulky substitutions significantly destabilize the cis isomers and also increase the energy barrier for cis/trans isomerization (especially in the water phase). These effects synergistically discourage the cis/trans isomerization of secondary amides. Therefore, secondary amides with bulky and more substitutions are much less likely to undergo cis/trans isomerization than the unsubstituted or less substituted second amides. Because cis/trans isomerization is an important step in the synthesis of piperazine-2,5-diones via cyclization of dipeptide esters, the rate of reaction for highly substituted dipeptide esters can be expected to be much slower than those with less substitutions. Moreover, for medicinal chemistry purposes, such highly substituted peptidomimetics can be expected to possess a very rigid peptide backbone. 

## Figures and Tables

**Table 1:** Model structures and their substitutions.

Compound ^a^	R^1^	R^2^	R^3^
N0000 (GGMe)	H	H	H
N0010	H	H	*S*-*t*-Bu
N0100	H	*S*-*t*-Bu	H
N0110	H	*S*-*t*-Bu	*S*-*t*-Bu
N011’0	H	*S*-*t*-Bu	R-t-Bu
N1000	t-Bu	H	H
N1010	*t*-Bu	H	*S*-*t*-Bu
N1100	*t*-Bu	*S*-*t*-Bu	H
N1110	*t*-Bu	*S*-*t*-Bu	*S*-*t*-Bu
N111’0	*t*-Bu	*S*-*t*-Bu	*R*-*t*-Bu

^a^ The first, second, and third digits in the name of each compound represent the variants of R^1^, R^2^, and R^3^, respectively. The fourth digit (0 in all) represents methyl ester and is specified in order to enable comparison to future variation at this site.

**Table 2:** Values (in degrees) of different dihedrals for the minimum energy geometries of different compounds in gas and water phases. The presence of bulky groups on both α-carbons can cause distortion in the planarity of amide moiety in terms of the ω dihedral, as highlighted in grey.

Compound	Gas Phase				Water Phase			
	ψ^N^	ω	φ^C^	ψ^C^	ψ^N^	ω	φ^C^	ψ^C^
N0000t	12.8	178.3	175.6	−179.3	15.2	179.2	178.5	−177.4
N0010t	−14.3	177.0	−122.7	141.7	−19.3	178.8	−124.4	142.6
N0100t	−22.8	−179.7	−173.1	178.8	−40.5	−177.9	66.3	−146.4
N0110t	−23.8	169.5	−114.3	141.2	−50.8	171.3	−135.3	152.0
N011’0t	14.3	−178.8	123.0	−140.9	13.4	178.5	127.3	−143.2
N1000t	−15.6	−178.0	−179.1	179.6	12.7	179.5	175.3	−177.3
N1010t	−17.9	176.6	−123.7	141.8	−12.2	178.7	−127.5	141.4
N1100t	140.7	177.5	−175.0	−179.9	134.6	174.6	−163.7	171.2
N1110t	−27.1	172.5	−117.0	140.5	−32.7	174.2	−123.1	141.1
N111’0t	143.0	−169.7	127.2	−145.3	148.2	−173.4	136.1	−147.7

The “t” after the compound name indicates the “trans” isomer.

**Table 3:** Calculation of *tert*-Bu additive energy in the gas phase.

Compound	^opt^E	Attachment Point	*tert*-Bu Additive Energy kcal mol^−1^ (E_tb_)
NH_3_	−35,508.975	sp^3^ nitrogen	E_tb_^N^ = −98,709.131
*tert*-Bu-NH_2_	−134,218.106		
CH_4_	−25,437.531	sp^3^ carbon	E_tb_^C^ = −98,712.134
*tert*-Bu-CH_3_	−124,149.665		

**Table 4:** Calculation of *tert*-Bu additive energy in the water phase.

Compound	^opt^E	Attachment Point	*tert*-Bu Additive Energy Kcal mol^−1^ (E_tb_)
NH_3_	−35,514.577	sp^3^ nitrogen	E_tb_^N^ = −98,707.646
*tert*-Bu-NH_2_	−134,222.223		
CH_4_	−25,436.175	sp^3^ carbon	E_tb_^C^ = −98,711.962
*tert*-Bu-CH_3_	−124,148.137		

**Table 5:** Calculation of steric energies in the gas phase.

Compound ^a^	^opt^E kcal mol^−1^	No. of *t*-Bu on sp^3^ Nitrogen	No. of *t*-Bu on sp^3^ Carbon	^Add^E kcal mol^−1^	^st^E kcal mol^−1^
N0000t	−333,806.071			−333,806.071	0
N0010t	−432,516.46		1	−432,518.205	1.745
N0100t	−432,515.901		1	−432,518.205	2.304
N0110t	−531,225.709		2	−531,230.339	4.63
N011’0t	−531,225.494		2	−531,230.339	4.845
N1000t	−432,514.375	1		−432,515.202	0.827
N1010t	−531,224.575	1	1	−531,227.336	2.761
N1100t	−531,222.518	1	1	−531,227.336	4.818
N1110t	−629,932.315	1	2	−629,939.47	7.155
N111’0t	−629,931.92	1	2	−629,939.47	7.55

^a^ The “t” after the compound name indicates the “trans” isomer.

**Table 6:** Calculation of steric energies in the water phase.

Compound ^a^	Solvation Energy (kcal mol^−1^)	^opt^E kcal mol^−1^	No. of *t*-Bu on sp^3^ nitrogen	No. of *t*-Bu on sp^3^ carbon	^Add^E kcal mol^-1^	^st^E kcal mol^−1^
N0000t	−16.33	−381,811.506			−381,811.506	0
N0010t	−16.85	−480,519.643		1	−480,523.468	3.825
N0100t	−14.86	−480,518.077		1	−480,523.468	5.391
N0110t	−16.1	−579,224.507		2	−579,235.43	10.923
N011’0t	−12.66	−579,225.518		2	−579,235.43	9.912
N1000t	−11.16	−480,518.478	1		−480,519.152	0.674
N1010t	−15.97	−579,226.192	1	1	−579,231.114	4.922
N1100t	−13.47	−579,222.324	1	1	−579,231.114	8.79
N1110t	−11.49	−677,932.254	1	2	−677,943.076	10.822
N111’0t	−11.34	−677,929.486	1	2	−677,943.076	13.59

^a^ The “t” after the compound name indicates the “trans” isomer.

**Table 7:** Dihedral angles (in degree) and relative energies of minimum energy cis isomers in the gas phase.

Compound ^a^	ψ^N^	ω	φ^C^	ψ^C^	ΔE ^b^	ΔG ^c^
N0000c	179.9	0.1	−179.9	179.8	4.63	4.92
N0010c	−176.7	−6.1	−115.2	132.8	5.23	5.14
N0100c	130.7	−2.4	−178.5	−179.3	5.12	4.92
N0110c	130.0	−8.5	−80.9	131.9	6.17	6.07
N011’0c	131.2	0.0	127.3	−136.5	4.96	5.18
N1000c	−162.2	2.1	−177.6	178.5	5.02	5.77
N1010c	−158.2	−5.1	−121.5	135.7	5.67	6.11
N1100c	137.5	−6.4	−175.1	−178.3	5.17	5.05
N1110c	138.9	−3.5	−86.9	132.0	7.52	7.71
N111’0c	140.4	6.9	104.4	−143.9	7.52	7.61

^a^ The “c” after the compound name indicates the “cis” isomer. ^b^ Gas-phase energy relative to the minimum energy trans geometry, calculated in kcal mol^−1^ at the B3LYP/6-311++G(3df,3pd) level. ^c^ Gibbs free energy change at 298.15 K relative to minimum energy trans geometry, calculated in kcal mol^−1^ at the B3LYP/6-31++G** level.

**Table 8:** Dihedral angles (in degree) and relative energies of minimum energy cis-isomers in the water dielectric phase.

Compound ^a^	Solvation Energy (kcal mol^−1^)	φ^N^	ω	φ^C^	ψ^C^	ΔE ^b^	ΔG ^c^
N0000c	−19.43	179.5	0.1	−179.6	179.7	2.03	2.1
N0010c	−17.85	−163.8	−0.6	−129.8	144.4	2.97	2.9
N0100c	−16.88	127.5	−2.1	−176.4	−177.4	2.11	2.3
N0110c	−15.67	128.2	4.3	−80.8	143.6	1.38	1.7
N011’0c	−14.7	130.7	8.3	133.4	−141.9	2.80	2.4
N1000c	−19.86	−174.0	0.7	−167.2	178.3	2.79	2.9
N1010c	−18.63	−159.0	0.0	−128.3	143.1	3.34	3.1
N1100c	−14.35	140.1	0.7	113.8	−163.8	3.92	3.7
N1110c	−11.87	134.6	−5.4	−94.3	138.8	7.66	8.1
N111’0c	−11.76	141.2	9.5	109.1	−147.5	4.86	4.94

^a^ The “c” after the compound name indicates the “cis” isomer. ^b^ Water phase energy relative to the minimum energy trans geometry, calculated in kcal mol^−1^ at the B3LYP/6-311++G(3df,3pd) level. ^c^ Gibbs free energy change at 298.15 K relative to minimum energy trans geometry, calculated in kcal mol^−1^ at the B3LYP/6-31++G ** level.

**Table 9:** Steric-factor effect on the cis-isomer based on the additivity principle in the gas phase.

Compound ^a^	^opt^E kcal mol^−1^	No. of *t*-Bu on sp^3^ Nitrogen	No. of *t*-Bu on sp^3^ Carbon	^Add^E kcal mol^−1^	^st^E kcal mol^−1^
N0000c	−333,801.441			−333,801.441	0
N0010c	−432,511.233		1	−432,513.575	2.342
N0100c	−432,510.785		1	−432,513.575	2.79
N0110c	−531,219.543		2	−531,225.709	6.166
N011’0c	−531,220.53		2	−531,225.709	5.179
N1000c	−432,509.352	1		−432,510.572	1.22
N1010c	−531,218.91	1	1	−531,222.706	3.796
N1100c	−531,217.348	1	1	−531,222.706	5.358
N1110c	−629,924.792	1	2	−629,934.84	10.048
N111’0c	−629,924.4	1	2	−629,934.84	10.44

^a^ The “c” after the compound name indicates the “cis” isomer.

**Table 10:** Steric-factor effect on the cis-isomer based on the additivity principle in the water phase.

Compound ^a^	^opt^E kcal mol^−1^	No. of *t*-Bu on sp^3^ Nitrogen	No. of *t*-Bu on sp^3^ Carbon	^Add^E kcal mol^−1^	^st^E kcal mol^−1^
N0000c	−333,819.556			−333,819.556	0
N0010c	−432,526.761		1	−432,531.518	4.757
N0100c	−432,526.055		1	−432,531.518	5.463
N0110c	−531,233.207		2	−531,243.48	10.273
N011’0c	−531,232.806		2	−531,243.48	10.674
N1000c	−432,525.775	1		−432,527.202	1.427
N1010c	−531,232.932	1	1	−531,239.164	6.232
N1100c	−531,228.49	1	1	−531,239.164	10.674
N1110c	−629,934.673	1	2	−629,951.126	16.453
N111’0c	−629,934.711	1	2	−629,951.126	16.415

^a^ The “c” after the compound name indicates the “cis” isomer.

**Table 11:** Energy-barrier values corresponding to *syn* and *anti* EBGs generated from optimized minimum energy trans and cis geometries, respectively, in the gas phase.

Compound	Anti120GP		Anti-120GP		Syn60GP		Syn-60GP		ΔG_eff_
	ΔE ^a^	ΔG ^b^	ΔE ^a^	ΔG ^b^	ΔE ^a^	ΔG ^b^	ΔE ^a^	ΔG ^b^	
N0000	19.53	13.70	19.53	13.72	25.46	19.91	25.26	19.84	13.70
N0010	24.22	18.34	19.34	11.93	25.22	17.86	27.99	20.23	11.93
N0100	20.96	13.40	20.51	12.43	26.30	18.63	25.26	20.38	12.43
N0110	25.64	18.72	19.76	12.34	23.98	17.63	28.18	22.10	12.34
N011’0	19.99	12.91	24.92	17.54	27.33	19.06	29.92	22.79	12.91
N1000	19.55	11.03	19.82	11.09	ND ^c^	ND	25.09	19.31	11.03
N1010	24.33	17.92	20.10	14.82	24.95	17.65	27.80	22.20	14.82
N1100	21.54	12.90	20.59	13.14	26.61	19.13	25.40	17.49	12.90
N1110	26.08	17.27	20.15	11.17	28.29	20.62	34.39	28.17	11.17
N111’0	21.43	12.59	26.18	17.45	30.79	22.72	23.68	14.51	12.59

^a^ Gas-phase energy relative to the minimum-energy trans geometry, calculated in kcal mol^−1^ at the B3LYP/6-311++G(3df,3pd) level. ^b^ Gibbs free energy at 298.15 K relative to the minimum-energy trans geometry, calculated in kcal mol^−1^ at the B3LYP/6-31++G** level. ^c^ ND: Not determined, assumed similar geometry as for the rotation in the opposite direction due to symmetry.

**Table 12:** Energy-barrier values corresponding to *syn* and *anti* EBGs generated from the optimized minimum-energy trans and cis geometries, respectively, in the water phase.

Compound	Anti120WP		Anti-120WP		Syn60WP		Syn-60WP		ΔG_eff_
	ΔE ^a^	ΔG ^b^	ΔE ^a^	ΔG ^b^	ΔE ^a^	ΔG ^b^	ΔE ^a^	ΔG ^b^	
N0000	19.18	13.82	19.18	13.74	24.29	20.07	ND ^c^	ND	13.74
N0010	25.40	20.48	19.03	13.67	29.42	23.23	24.23	20.47	13.67
N0100	22.45	18.28	20.66	16.24	23.31	18.6	26.09	19.91	16.24
N0110	25.13	21.06	19.32	13.46	22.23	19.06	20.47	16.61	13.46
N011’0	26.44	20.83	26.55	20.67	23.39	18.55	26.51	21.71	18.55
N1000	22.30	16.89	22.35	16.85	ND	ND	23.86	19.40	16.85
N1010	26.60	20.05	19.00	12.72	29.60	23.96	24.29	19.71	12.72
N1100	22.54	14.06	23.71	16.92	26.50	19.28	23.04	16.86	14.06
N1110	29.46	21.96	24.53	19.22	29.24	24.21	30.37	23.98	19.22
N111’0	23.15	14.50	27.71	21.34	26.65	19.74	23.78	17.50	14.50

^a^ Water-phase energy relative to the minimum-energy trans geometry, calculated in kcal mol^−1^ at the B3LYP/6-311++G(3df,3pd) level. ^b^ Gibbs free energy at 298.15 K relative to the minimum energy trans geometry, calculated in kcal mol^-1^ at the B3LYP/6-31++G** level. ^c^ ND: Not determined, assumed similar geometry as for the rotation in the opposite direction due to symmetry.

## Supplementary Materials

Supplementary Materials are available online.
