# Supplementary material for: Density Functional Studies on Secondary Amides: Role of Steric Factors in Cis/Trans Isomerization
Source: Molecules. 2018 Sep 25;23(10):2455. doi: 10.3390/molecules23102455 (PMC6222500; doi:10.3390/molecules23102455)
Supplement: Supplementary file 1 [file molecules-23-02455-s001.zip › SI_Coordinates.docx]

Density Functional Studies on Secondary Amides: Role of Steric Factors in cis-trans isomerization

Balmukund S. Thakkar, John-Sigurd M. Svendsen and Richard A. Engh*

Department of Chemistry, UiT The Arctic University of Norway, Tromsø-9037, Norway

**Supporting Information**

This document provides Cartesian coordinates (in Å) for the optimized geometries of GGMe (N0000) and its substituted derivatives. The geometries include the minimum energy geometries of trans and cis isomers as well as energy barrier geometries (EBGs) for each compound i.e. “*anti*” type with ω at ~120˚ and ~(-120)˚, and “*syn*” type with ω at 60˚ and ~(-60)˚.

Each trans/cis geometry is named as: [Compound][t/c]^a^_[GP/WP]^b^, while each energy barrier geometry is named as [Compound]_[Anti/Syn]^c^[ω value]^d^[GP/WP]^b^

1. N0000t_GP

N 9.67400 -6.19120 2.11600

C 9.08180 -4.97260 1.55780

C 9.11010 -4.92990 0.02620

N 9.41290 -6.10200 -0.58000

C 9.44090 -6.22240 -2.01770

C 9.91300 -7.60580 -2.41440

O 9.95840 -7.73010 -3.75240

C 10.39010 -9.01140 -4.25740

O 10.20810 -8.49090 -1.63650

O 8.85080 -3.89370 -0.58670

H 9.24860 -6.44800 3.00000

H 10.67270 -6.09040 2.27080

H 9.54020 -4.03750 1.90510

H 8.02520 -4.92710 1.84370

H 9.60480 -6.91420 -0.00060

H 10.10490 -5.47140 -2.46260

H 8.45080 -6.04530 -2.45830

H 10.35430 -8.91930 -5.34200

H 11.40630 -9.22810 -3.92020

H 9.71800 -9.80040 -3.91260

^a^ t: trans, c: cis; ^b^ GP: Gas phase; WP: water phase (water dielectric continuum)

^c^ Anti: EBG of “*anti*” type; Syn: EBG of “*syn*” type

^d^ Among 120, -120, 60 or -60, which value (degrees) is closest from the ω of the EBG.

1. N0010t_GP

N 9.18410 -6.30900 2.11330

C 9.15040 -4.95490 1.55340

C 9.10980 -4.92840 0.01930

N 9.41490 -6.10970 -0.57740

C 9.37000 -6.30770 -2.01560

C 8.40920 -7.46310 -2.29820

O 7.67010 -7.24570 -3.40300

C 6.75100 -8.29590 -3.76820

O 8.31440 -8.46420 -1.61430

O 8.84920 -3.88740 -0.58250

C 10.79530 -6.53110 -2.64980

C 10.66130 -6.73590 -4.17190

C 11.63170 -5.26230 -2.38990

C 11.50350 -7.74850 -2.02410

H 8.25080 -6.67790 2.26940

H 9.68090 -6.34090 2.99690

H 10.06510 -4.42620 1.84360

H 8.31000 -4.33800 1.89640

H 9.54010 -6.90540 0.04120

H 8.95030 -5.39550 -2.44690

H 7.29230 -9.22430 -3.96570

H 6.02990 -8.46350 -2.96500

H 6.25110 -7.94090 -4.66870

H 10.11370 -5.91170 -4.64190

H 11.65680 -6.78110 -4.62680

H 10.14300 -7.66730 -4.42110

H 11.16480 -4.37680 -2.83320

H 11.75420 -5.07520 -1.31930

H 12.62960 -5.37840 -2.82750

H 11.65410 -7.61650 -0.94750

H 10.94180 -8.67550 -2.17500

H 12.49200 -7.87510 -2.47970

1. N0100t_GP

N 8.89260 -6.36140 2.01520

C 9.10300 -4.97220 1.57340

C 9.10990 -4.92900 0.03410

N 9.41210 -6.09850 -0.58370

C 9.46020 -6.19920 -2.02240

C 9.65160 -7.64420 -2.43460

O 9.67320 -7.76070 -3.77390

C 9.85910 -9.09480 -4.29230

O 9.77630 -8.57690 -1.66650

O 8.85190 -3.89800 -0.59090

C 10.41000 -4.36090 2.19930

C 10.32330 -4.46040 3.73960

C 10.51190 -2.86780 1.82600

C 11.67110 -5.10400 1.71520

H 8.98060 -6.44400 3.02260

H 7.95460 -6.67340 1.77580

H 8.27880 -4.30400 1.86600

H 9.60640 -6.91940 -0.01880

H 10.27760 -5.60020 -2.44630

H 8.53970 -5.80970 -2.47330

H 10.81480 -9.50240 -3.95500

H 9.04920 -9.74720 -3.95820

H 9.84590 -8.98630 -5.37600

H 10.41390 -5.48980 4.10460

H 11.14410 -3.89470 4.19330

H 9.38410 -4.03820 4.11710

H 9.65160 -2.30420 2.20480

H 11.41740 -2.43880 2.27010

H 10.54960 -2.71770 0.74510

H 11.83210 -4.96460 0.64120

H 12.55540 -4.71750 2.23420

H 11.60460 -6.17860 1.91150

1. N0110t_GP

N 8.85950 -6.35310 2.01470

C 9.09740 -4.96880 1.57130

C 9.10930 -4.92650 0.02730

N 9.41440 -6.10780 -0.58140

C 9.25780 -6.32070 -2.01170

C 8.16520 -7.37420 -2.20750

O 7.37540 -7.09360 -3.26130

C 6.32620 -8.04420 -3.53870

O 8.01340 -8.35100 -1.49800

O 8.85020 -3.89120 -0.58640

C 10.39640 -4.37280 2.22530

C 11.65060 -5.18130 1.83940

C 10.57200 -2.90390 1.78980

C 10.23460 -4.39510 3.76310

C 10.60650 -6.70200 -2.72950

C 10.34190 -6.98680 -4.22170

C 11.55840 -5.49520 -2.61550

C 11.25760 -7.93750 -2.07720

H 7.91080 -6.64490 1.79150

H 8.96550 -6.44260 3.01970

H 8.27730 -4.28950 1.84870

H 9.48880 -6.91490 0.03050

H 8.90400 -5.37760 -2.43460

H 5.80350 -7.65060 -4.40960

H 6.75020 -9.02830 -3.75260

H 5.65070 -8.12200 -2.68370

H 12.52510 -4.79270 2.37330

H 11.54140 -6.24040 2.09240

H 11.85930 -5.11000 0.76770

H 11.46260 -2.48310 2.27030

H 10.68310 -2.80690 0.70810

H 9.70990 -2.29530 2.08470

H 11.05880 -3.84550 4.23020

H 9.29900 -3.91570 4.07490

H 10.25950 -5.40840 4.18020

H 11.29400 -7.13260 -4.74330

H 9.74440 -7.89210 -4.37050

H 9.81700 -6.15530 -4.70430

H 12.51670 -5.72740 -3.09350

H 11.14030 -4.60870 -3.10420

H 11.75710 -5.24020 -1.57100

H 11.51830 -7.74900 -1.03070

H 10.60160 -8.81300 -2.11060

H 12.18400 -8.18980 -2.60520

1. N011'0t_GP

N 9.67810 -6.27420 2.01980

C 9.05320 -5.01500 1.58120

C 9.10960 -4.92790 0.03060

N 9.41320 -6.10290 -0.58250

C 9.54180 -6.23460 -2.02270

C 10.93780 -6.78390 -2.31880

O 11.48380 -6.20980 -3.40880

C 12.79340 -6.68270 -3.78590

O 11.50020 -7.63600 -1.65830

O 8.85110 -3.89470 -0.58860

C 9.55710 -3.71590 2.31370

C 9.58400 -3.98370 3.83510

C 8.58650 -2.54160 2.06350

C 10.97360 -3.33310 1.84010

C 8.39620 -7.10650 -2.66460

C 8.60830 -7.20180 -4.18870

C 7.05420 -6.39830 -2.39190

C 8.36000 -8.52300 -2.05820

H 9.31430 -6.57340 2.91780

H 10.68640 -6.17850 2.11610

H 7.98270 -5.11020 1.81160

H 9.65190 -6.87860 0.02720

H 13.06440 -6.10630 -4.66980

H 13.50580 -6.50940 -2.97630

H 12.76160 -7.75080 -4.01440

H 9.82230 -3.05770 4.36940

H 8.60780 -4.32960 4.19880

H 10.33630 -4.72620 4.12070

H 8.91770 -1.65970 2.62490

H 8.52890 -2.28880 1.00510

H 7.57560 -2.79190 2.41000

H 10.97630 -3.06090 0.78110

H 11.34520 -2.47460 2.41060

H 11.69200 -4.15000 1.98850

H 7.75580 -7.71330 -4.64870

H 8.69230 -6.20970 -4.64560

H 9.50950 -7.76680 -4.44720

H 6.23340 -6.97280 -2.83580

H 6.85930 -6.30850 -1.31950

H 7.03800 -5.39120 -2.82090

H 7.55310 -9.10220 -2.52070

H 9.29780 -9.06400 -2.21760

H 8.16580 -8.49470 -0.98080

H 9.47100 -5.22760 -2.44050

1. N1000t_GP

N 9.13650 -6.31130 2.11400

C 9.14080 -4.95790 1.55960

C 9.11010 -4.92960 0.02730

N 9.41280 -6.10160 -0.58060

C 9.46560 -6.21680 -2.01780

C 9.80340 -7.63990 -2.41160

O 9.84850 -7.76810 -3.74920

C 10.16860 -9.08260 -4.25160

O 10.00990 -8.54860 -1.63200

O 8.85090 -3.89430 -0.58730

C 9.70650 -6.51880 3.47130

C 9.39600 -7.97740 3.84660

C 9.08910 -5.57150 4.52390

C 11.23250 -6.32730 3.41560

H 8.18430 -6.67090 2.10700

H 10.06550 -4.44770 1.84710

H 8.31470 -4.31730 1.89920

H 9.62280 -6.90800 0.00050

H 10.21510 -5.54010 -2.44820

H 8.50940 -5.93380 -2.47540

H 11.15580 -9.39180 -3.90070

H 9.42220 -9.80740 -3.91900

H 10.15700 -8.98590 -5.33650

H 8.31350 -8.15070 3.89400

H 9.81970 -8.66390 3.10710

H 9.81290 -8.22210 4.82870

H 9.49550 -5.77580 5.52070

H 9.29610 -4.52140 4.29240

H 8.00100 -5.69880 4.57300

H 11.67800 -6.55900 4.38870

H 11.67300 -6.99040 2.66480

H 11.51240 -5.29780 3.16970

1. N1010t_GP

N 9.11200 -6.30530 2.11330

C 9.15790 -4.95420 1.55440

C 9.10970 -4.92800 0.02070

N 9.41480 -6.10950 -0.57810

C 9.35670 -6.31010 -2.01560

C 8.41910 -7.48750 -2.28200

O 7.66160 -7.29170 -3.37790

C 6.76220 -8.36400 -3.72800

O 8.35570 -8.48810 -1.59310

O 8.84930 -3.88810 -0.58310

C 10.77820 -6.50340 -2.66880

C 11.58880 -5.21460 -2.42570

C 11.52200 -7.70130 -2.04720

C 10.62890 -6.71850 -4.18810

C 9.67590 -6.52490 3.47130

C 9.31700 -7.97000 3.85520

C 11.20740 -6.38360 3.41350

C 9.09150 -5.55130 4.51870

H 8.14910 -6.63540 2.10800

H 10.10310 -4.47650 1.83120

H 8.35760 -4.28500 1.89950

H 9.52620 -6.90690 0.04120

H 8.91190 -5.40870 -2.44400

H 7.32200 -9.28100 -3.92720

H 6.05380 -8.54370 -2.91620

H 6.24470 -8.02460 -4.62470

H 12.58310 -5.31050 -2.87620

H 11.09610 -4.34200 -2.86640

H 11.72090 -5.01960 -1.35770

H 12.50770 -7.80730 -2.51390

H 11.68130 -7.56060 -0.97300

H 10.98010 -8.64160 -2.18760

H 10.13070 -7.66370 -4.42630

H 10.05460 -5.91060 -4.65430

H 11.61900 -6.74110 -4.65640

H 9.72790 -8.22340 4.83760

H 8.22960 -8.10650 3.90550

H 9.71510 -8.67470 3.11860

H 11.64590 -6.61870 4.38890

H 11.62640 -7.06920 2.67050

H 11.52030 -5.36650 3.15560

H 7.99980 -5.64190 4.56910

H 9.49170 -5.76420 5.51610

H 9.33330 -4.50990 4.28180

1. N1100t_GP

N 9.69340 -3.70790 2.11140

C 9.11630 -4.94530 1.58520

C 9.10940 -4.92750 0.04160

N 9.41130 -6.09540 -0.58740

C 9.38320 -6.21070 -2.02870

C 9.64140 -7.65070 -2.42350

O 9.62400 -7.79610 -3.75700

C 9.85120 -9.13170 -4.25760

O 9.83830 -8.55760 -1.63800

O 8.85310 -3.90260 -0.59470

C 7.67270 -5.19050 2.17720

C 6.74540 -3.99230 1.89530

C 7.80980 -5.37860 3.70060

C 7.05200 -6.46820 1.57680

C 11.16050 -3.61600 2.32330

C 11.99580 -3.95620 1.06660

C 11.43160 -2.15550 2.72650

C 11.56980 -4.53810 3.48640

H 9.40200 -2.95080 1.49530

H 9.73060 -5.79040 1.91680

H 9.58680 -6.94650 -0.06870

H 10.13810 -5.56730 -2.49760

H 8.41630 -5.89070 -2.43640

H 10.83330 -9.49130 -3.94330

H 9.07960 -9.81000 -3.88690

H 9.79980 -9.04320 -5.34190

H 5.75070 -4.18690 2.31280

H 7.13000 -3.08120 2.36330

H 6.63520 -3.80510 0.82310

H 6.82190 -5.51520 4.15530

H 8.41190 -6.26370 3.94150

H 8.28480 -4.50650 4.15590

H 7.70010 -7.34220 1.72120

H 6.09890 -6.68610 2.07100

H 6.84990 -6.36910 0.50550

H 13.06480 -3.81220 1.26190

H 11.85800 -4.99840 0.75920

H 11.71320 -3.31470 0.22520

H 12.49420 -2.00300 2.94260

H 11.15280 -1.46780 1.91900

H 10.85230 -1.89010 3.61620

H 10.99710 -4.29550 4.38660

H 11.40500 -5.59570 3.25370

H 12.63550 -4.41820 3.70890

1. N1110t_GP

N 8.84020 -6.31800 2.06750

C 9.13000 -4.96310 1.57630

C 9.10940 -4.92700 0.02700

N 9.41440 -6.10770 -0.58110

C 9.28960 -6.30420 -2.01660

C 8.23930 -7.38880 -2.25620

O 7.45430 -7.10670 -3.31290

C 6.44840 -8.08820 -3.63900

O 8.11460 -8.39180 -1.57830

O 8.85010 -3.89090 -0.58630

C 10.47070 -4.38420 2.17440

C 10.76420 -2.99020 1.58150

C 11.67270 -5.31080 1.89420

C 10.30450 -4.21430 3.70170

C 10.66260 -6.63130 -2.71720

C 11.57330 -5.39690 -2.56830

C 11.34080 -7.85570 -2.07270

C 10.43330 -6.89900 -4.21850

C 7.44810 -6.66010 2.47380

C 6.49060 -6.46730 1.28540

C 7.47460 -8.14690 2.86830

C 6.95940 -5.82230 3.67730

H 9.46000 -6.55920 2.83320

H 8.34590 -4.25040 1.86420

H 9.41260 -6.91630 0.03500

H 8.91470 -5.36600 -2.43320

H 5.92550 -7.68710 -4.50640

H 6.91500 -9.04720 -3.87690

H 5.76200 -8.22010 -2.79970

H 11.64870 -2.56640 2.07060

H 10.95140 -3.02740 0.50660

H 9.92600 -2.30430 1.74000

H 12.58170 -4.87980 2.32850

H 11.54660 -6.30900 2.32760

H 11.84230 -5.43500 0.82100

H 10.14470 -5.16340 4.22440

H 11.20840 -3.76280 4.12490

H 9.46100 -3.55630 3.93760

H 12.54650 -5.59130 -3.03270

H 11.13520 -4.51660 -3.05060

H 11.74610 -5.15100 -1.51710

H 12.28840 -8.06560 -2.58130

H 11.56650 -7.67810 -1.01630

H 10.71670 -8.75230 -2.13880

H 9.86720 -7.81980 -4.39320

H 9.89160 -6.07590 -4.69680

H 11.39850 -7.00670 -4.72520

H 5.47450 -6.74940 1.58020

H 6.45620 -5.42530 0.95050

H 6.78800 -7.09550 0.44160

H 6.48100 -8.48180 3.18430

H 7.79800 -8.76290 2.02410

H 8.16360 -8.32100 3.70450

H 6.93490 -4.75240 3.44690

H 5.94450 -6.11750 3.96590

H 7.61020 -5.96750 4.54700

1. N111'0t_GP

N 9.64300 -3.67420 2.09290

C 9.11470 -4.94130 1.58480

C 9.10900 -4.92560 0.03610

N 9.41320 -6.10290 -0.58550

C 9.66460 -6.20830 -2.01920

C 11.01110 -6.91520 -2.17120

O 11.71030 -6.46510 -3.22560

C 12.98600 -7.09950 -3.46140

O 11.41090 -7.78670 -1.42070

O 8.85160 -3.89700 -0.59100

C 7.69190 -5.24410 2.19750

C 7.14750 -6.58640 1.66950

C 6.69380 -4.11820 1.86590

C 7.84550 -5.35050 3.72770

C 8.50550 -6.92490 -2.81230

C 8.88040 -7.03570 -4.30400

C 8.22780 -8.32980 -2.24410

C 7.23690 -6.05800 -2.68780

C 11.10510 -3.52950 2.31120

C 11.53370 -4.39560 3.50990

C 11.96110 -3.88980 1.07380

C 11.32810 -2.04750 2.66130

H 9.33290 -2.94220 1.45620

H 9.76870 -5.75550 1.91820

H 9.73840 -6.87970 -0.02450

H 13.64080 -6.96260 -2.59810

H 12.85050 -8.16760 -3.64660

H 13.39490 -6.60320 -4.34050

H 6.21170 -6.83600 2.18150

H 6.93700 -6.55840 0.59680

H 7.84970 -7.40930 1.85650

H 5.71720 -4.34390 2.30950

H 7.03220 -3.16180 2.27600

H 6.55750 -3.99600 0.78720

H 8.25840 -4.42760 4.14070

H 6.87030 -5.53660 4.19230

H 8.50950 -6.17860 4.00570

H 8.02460 -7.42120 -4.86850

H 9.14910 -6.06110 -4.72560

H 9.72040 -7.71660 -4.47310

H 7.41450 -8.80200 -2.80600

H 9.10390 -8.98290 -2.31110

H 7.91890 -8.28420 -1.19500

H 6.92710 -5.94060 -1.64650

H 7.39320 -5.05680 -3.10160

H 6.41180 -6.53000 -3.23290

H 12.59490 -4.23910 3.73200

H 10.95030 -4.13530 4.39810

H 11.39810 -5.46510 3.31600

H 13.02300 -3.70240 1.27070

H 11.86290 -4.94810 0.80810

H 11.66390 -3.29140 0.20620

H 10.73160 -1.76650 3.53480

H 12.38310 -1.85480 2.88190

H 11.03730 -1.39960 1.82560

H 9.75550 -5.19070 -2.40710

1. N0000t_WP

N 9.71170 -6.13760 2.17170

C 9.07820 -4.95880 1.56200

C 9.11280 -4.94070 0.03210

N 9.41120 -6.09530 -0.57960

C 9.46600 -6.24230 -2.02260

C 9.86520 -7.66100 -2.38560

O 9.97620 -7.80560 -3.70720

C 10.33930 -9.12110 -4.21900

O 10.05450 -8.55580 -1.57490

O 8.84980 -3.88960 -0.59300

H 9.38230 -6.24610 3.13250

H 10.72280 -6.00450 2.23770

H 9.50340 -4.00090 1.89010

H 8.01670 -4.94140 1.84030

H 9.59190 -6.90600 0.00510

H 10.19100 -5.54680 -2.46640

H 8.49150 -6.02990 -2.48520

H 10.40790 -8.99380 -5.29940

H 11.30060 -9.43020 -3.80350

H 9.56300 -9.84550 -3.96330

1. N0010t_WP

N 9.06100 -6.27150 2.17060

C 9.14630 -4.93750 1.55770

C 9.11200 -4.93720 0.02650

N 9.41340 -6.10390 -0.57810

C 9.41010 -6.30790 -2.02390

C 8.47550 -7.48370 -2.32980

O 7.78030 -7.29640 -3.45590

C 6.89580 -8.36880 -3.89060

O 8.38140 -8.48380 -1.63200

O 8.84840 -3.88440 -0.58890

C 10.85750 -6.50410 -2.63030

C 10.76250 -6.62160 -4.16640

C 11.69550 -5.25510 -2.28520

C 11.55260 -7.75700 -2.06100

H 8.08660 -6.57650 2.21800

H 9.38600 -6.22410 3.13800

H 10.10180 -4.47610 1.83920

H 8.35750 -4.24530 1.87890

H 9.57160 -6.89650 0.03790

H 8.98320 -5.40910 -2.47560

H 7.47340 -9.27740 -4.07530

H 6.13350 -8.55410 -3.13070

H 6.44250 -8.00520 -4.81290

H 10.23550 -5.76360 -4.60020

H 11.77270 -6.64580 -4.59140

H 10.24910 -7.53470 -4.48540

H 11.24060 -4.34310 -2.68830

H 11.81400 -5.13360 -1.20390

H 12.69590 -5.35560 -2.72200

H 11.65140 -7.71020 -0.97020

H 11.01760 -8.67700 -2.31850

H 12.56340 -7.83310 -2.47830

1. N0100t_WP

N 8.53240 -6.17790 2.10520

C 9.12850 -4.92680 1.58160

C 9.11140 -4.93550 0.04340

N 9.41050 -6.09230 -0.58610

C 9.45980 -6.16970 -2.03490

C 10.60170 -5.33820 -2.62900

O 10.25740 -4.82420 -3.81780

C 11.25380 -4.03160 -4.52330

O 11.69630 -5.18480 -2.11310

O 8.85190 -3.89770 -0.59790

C 10.56780 -4.64890 2.16800

C 10.46140 -4.54810 3.70650

C 11.08370 -3.29310 1.63670

C 11.58530 -5.75020 1.80800

H 8.47430 -6.12360 3.12410

H 7.55900 -6.23370 1.79430

H 8.52270 -4.04870 1.84850

H 9.56980 -6.93140 -0.03830

H 8.51510 -5.84050 -2.47940

H 9.62960 -7.21460 -2.32120

H 11.54450 -3.17270 -3.91410

H 12.12780 -4.64450 -4.75430

H 10.76020 -3.70320 -5.43830

H 10.20690 -5.50700 4.17160

H 11.42820 -4.23740 4.11990

H 9.71330 -3.80540 4.00950

H 10.38850 -2.48010 1.87720

H 12.04890 -3.05990 2.10190

H 11.23050 -3.30260 0.55260

H 11.76470 -5.79790 0.72990

H 12.54340 -5.53500 2.29620

H 11.25230 -6.73680 2.14640

1. N0110t_WP

N 8.24670 -6.08810 2.03180

C 9.09150 -4.95740 1.57620

C 9.11130 -4.93450 0.03170

N 9.41300 -6.10240 -0.58120

C 9.30910 -6.34160 -2.02130

C 8.63610 -7.71020 -2.17780

O 7.94790 -7.82330 -3.31390

C 7.32820 -9.11230 -3.59400

O 8.73670 -8.60880 -1.35270

O 8.84950 -3.88860 -0.59110

C 10.51530 -4.96290 2.25060

C 11.33820 -6.23300 1.95070

C 11.30270 -3.72520 1.77070

C 10.32590 -4.85100 3.78080

C 10.69160 -6.26320 -2.79680

C 10.43050 -6.26330 -4.31820

C 11.39420 -4.93870 -2.43420

C 11.61850 -7.44070 -2.43240

H 7.31760 -5.99800 1.61180

H 8.08410 -5.99950 3.03690

H 8.64250 -3.98910 1.83930

H 9.48020 -6.92600 0.00780

H 8.65180 -5.57530 -2.44370

H 6.82070 -8.98100 -4.54960

H 8.09650 -9.88560 -3.66690

H 6.61470 -9.36170 -2.80560

H 12.25570 -6.22300 2.55080

H 10.78600 -7.14460 2.20210

H 11.63880 -6.28850 0.90010

H 12.27460 -3.68210 2.27630

H 11.49350 -3.75080 0.69250

H 10.76390 -2.79740 1.99600

H 11.30120 -4.71610 4.26300

H 9.69840 -3.99150 4.04610

H 9.87700 -5.75400 4.20990

H 11.38060 -6.11940 -4.84590

H 9.99290 -7.20200 -4.66960

H 9.75960 -5.44430 -4.60510

H 12.31820 -4.85260 -3.01720

H 10.76550 -4.07200 -2.66160

H 11.66650 -4.89990 -1.37600

H 11.79730 -7.50180 -1.35300

H 11.21580 -8.40360 -2.76660

H 12.58980 -7.30230 -2.92170

1. N011'0t_WP

N 9.64610 -6.29180 2.04350

C 9.04730 -5.02060 1.58500

C 9.11130 -4.93470 0.03420

N 9.41230 -6.09950 -0.58220

C 9.49620 -6.26880 -2.02580

C 10.85340 -6.90180 -2.35090

O 11.37410 -6.42150 -3.48470

C 12.63760 -6.98470 -3.94030

O 11.39880 -7.75220 -1.66090

O 8.85020 -3.89130 -0.59250

C 9.57330 -3.72790 2.31980

C 9.61550 -4.00740 3.83940

C 8.60550 -2.54440 2.10020

C 10.98820 -3.34960 1.83650

C 8.27920 -7.08790 -2.61620

C 8.42140 -7.20670 -4.14730

C 6.98400 -6.31040 -2.30050

C 8.19170 -8.49520 -1.99400

H 9.27200 -6.54950 2.95600

H 10.65830 -6.21080 2.15890

H 7.97120 -5.09210 1.80530

H 9.62460 -6.87900 0.03450

H 12.85400 -6.47350 -4.87870

H 13.41970 -6.78820 -3.20350

H 12.53070 -8.05990 -4.10120

H 9.86330 -3.08320 4.37410

H 8.64180 -4.35590 4.20680

H 10.36850 -4.75550 4.10620

H 8.94020 -1.67720 2.68300

H 8.54790 -2.25810 1.04980

H 7.59470 -2.80460 2.43950

H 10.98900 -3.06390 0.77980

H 11.36990 -2.49910 2.41360

H 11.69730 -4.17700 1.96810

H 7.51930 -7.67390 -4.55900

H 8.53530 -6.22220 -4.61620

H 9.27640 -7.82610 -4.43890

H 6.12380 -6.85140 -2.71120

H 6.82820 -6.20450 -1.22240

H 6.99930 -5.30970 -2.74660

H 7.32120 -9.02090 -2.40280

H 9.07930 -9.09690 -2.21540

H 8.06700 -8.45080 -0.90610

H 9.47230 -5.27310 -2.47470

1. N1000t_WP

N 9.66690 -6.20140 2.13480

C 9.08270 -4.98670 1.55980

C 9.11320 -4.94200 0.02530

N 9.41210 -6.09890 -0.57630

C 9.48820 -6.29380 -2.00990

C 9.96550 -7.70690 -2.30380

O 10.10430 -7.91040 -3.61490

C 10.55720 -9.22770 -4.04490

O 10.19170 -8.54960 -1.44870

O 8.84850 -3.88460 -0.58960

C 11.04930 -6.11820 2.70090

C 11.44490 -7.55060 3.10050

C 11.10570 -5.20790 3.94810

C 12.03470 -5.59940 1.63800

H 9.04620 -6.56900 2.85580

H 9.55120 -4.05640 1.90790

H 8.02390 -4.91790 1.84250

H 9.60120 -6.88510 0.04370

H 10.18850 -5.58400 -2.47100

H 8.51140 -6.14710 -2.49210

H 11.55390 -9.42500 -3.64420

H 9.85410 -9.99150 -3.70630

H 10.58120 -9.17760 -5.13330

H 10.74410 -7.95760 3.83990

H 11.44460 -8.21030 2.22590

H 12.44720 -7.56180 3.54390

H 12.12070 -5.18780 4.36180

H 10.82610 -4.17510 3.71190

H 10.42920 -5.57880 4.72730

H 13.04740 -5.59190 2.05570

H 12.03810 -6.24070 0.75100

H 11.80560 -4.57450 1.32480

1. N1010t_WP

N 9.22690 -6.34800 2.08430

C 9.15060 -4.98600 1.55440

C 9.11270 -4.93990 0.01700

N 9.41430 -6.10760 -0.57320

C 9.43360 -6.37380 -2.00350

C 8.56080 -7.61140 -2.24610

O 7.83630 -7.51550 -3.36550

C 7.00070 -8.65600 -3.71760

O 8.53120 -8.58100 -1.50240

O 8.84680 -3.87800 -0.58430

C 10.89720 -6.53840 -2.57880

C 11.65690 -5.21710 -2.33740

C 11.65430 -7.69250 -1.89200

C 10.82810 -6.80040 -4.09700

C 10.15670 -6.63680 3.21180

C 9.92500 -8.11010 3.59080

C 11.61310 -6.46230 2.74290

C 9.88750 -5.74180 4.44150

H 8.29230 -6.67330 2.33020

H 10.03020 -4.40060 1.84650

H 8.27620 -4.42360 1.90840

H 9.53500 -6.87220 0.09350

H 8.97010 -5.52260 -2.50940

H 7.62140 -9.54440 -3.85430

H 6.26110 -8.83010 -2.93280

H 6.51580 -8.37420 -4.65210

H 12.67100 -5.29880 -2.74540

H 11.16080 -4.37300 -2.83000

H 11.74520 -4.98940 -1.27030

H 12.67010 -7.75300 -2.29910

H 11.74450 -7.53450 -0.81170

H 11.17000 -8.66010 -2.05850

H 10.37090 -7.76800 -4.33060

H 10.25780 -6.01830 -4.61190

H 11.84290 -6.80650 -4.51120

H 10.58800 -8.40310 4.41250

H 8.89020 -8.27170 3.91750

H 10.12180 -8.76500 2.73480

H 12.29970 -6.72690 3.55490

H 11.82450 -7.11480 1.88860

H 11.83670 -5.42950 2.45220

H 8.85310 -5.85980 4.78570

H 10.55490 -6.01200 5.26830

H 10.05460 -4.68290 4.21360

1. N1100t_WP

N 9.79610 -3.85600 2.19380

C 9.09780 -4.98780 1.58300

C 9.11120 -4.93450 0.03930

N 9.41160 -6.09670 -0.58450

C 9.36040 -6.26170 -2.02670

C 9.35450 -7.73960 -2.37750

O 9.11850 -7.92690 -3.67710

C 9.09290 -9.29900 -4.16830

O 9.55120 -8.64070 -1.57560

O 8.85100 -3.89430 -0.59530

C 7.62620 -5.11810 2.15130

C 6.85130 -3.79150 2.03180

C 7.73490 -5.50500 3.64070

C 6.85260 -6.22890 1.41200

C 11.25850 -3.93910 2.44710

C 12.09330 -4.16270 1.16520

C 11.65180 -2.59010 3.07700

C 11.55520 -5.06180 3.45970

H 9.59330 -3.00720 1.66710

H 9.60860 -5.90990 1.87990

H 9.65620 -6.91590 -0.03630

H 10.22350 -5.79610 -2.52710

H 8.46010 -5.78960 -2.43740

H 10.06650 -9.76860 -4.01180

H 8.31140 -9.86220 -3.65350

H 8.87070 -9.21380 -5.23210

H 5.84580 -3.91330 2.45230

H 7.35070 -2.99180 2.58780

H 6.74610 -3.47590 0.98900

H 6.73570 -5.56500 4.08860

H 8.21610 -6.48380 3.76250

H 8.31800 -4.76380 4.19420

H 7.39400 -7.18250 1.43790

H 5.87970 -6.38540 1.89310

H 6.66390 -5.97350 0.36320

H 13.16500 -4.16690 1.39650

H 11.85320 -5.12100 0.69220

H 11.90540 -3.36530 0.43700

H 12.72530 -2.56560 3.29470

H 11.42610 -1.75960 2.39620

H 11.10180 -2.42760 4.01010

H 10.96660 -4.92380 4.37270

H 11.33850 -6.05750 3.05670

H 12.61750 -5.04650 3.72800

1. N1110t_WP

N 8.69120 -6.29570 2.06200

C 9.12040 -4.97410 1.57860

C 9.11150 -4.93540 0.02760

N 9.41370 -6.10490 -0.57910

C 9.34970 -6.33590 -2.01840

C 8.40600 -7.51840 -2.26770

O 7.64190 -7.33510 -3.35030

C 6.75150 -8.42070 -3.73740

O 8.35950 -8.51820 -1.56560

O 8.84860 -3.88510 -0.58900

C 10.50720 -4.54170 2.20200

C 10.99190 -3.21850 1.57320

C 11.59690 -5.61720 2.00330

C 10.30840 -4.29640 3.71640

C 10.77110 -6.55440 -2.67250

C 11.58250 -5.25530 -2.48950

C 11.52450 -7.72750 -2.01460

C 10.61490 -6.82720 -4.18280

C 7.24450 -6.49530 2.38330

C 6.37740 -6.20270 1.14650

C 7.09440 -7.97620 2.77130

C 6.77780 -5.61240 3.56350

H 9.22780 -6.56420 2.88360

H 8.41130 -4.18360 1.86090

H 9.44930 -6.90960 0.04410

H 8.90930 -5.44560 -2.47450

H 6.25090 -8.06900 -4.63980

H 7.33180 -9.32310 -3.94340

H 6.02750 -8.61120 -2.94200

H 11.89970 -2.88420 2.08860

H 11.23280 -3.32620 0.51160

H 10.23780 -2.42940 1.66970

H 12.53610 -5.27040 2.44920

H 11.34130 -6.56830 2.48300

H 11.78400 -5.81370 0.94310

H 9.99980 -5.19870 4.25560

H 11.25280 -3.96170 4.16050

H 9.55730 -3.51840 3.89690

H 12.57530 -5.37670 -2.93750

H 11.09170 -4.40540 -2.97770

H 11.72190 -5.01010 -1.43260

H 12.51840 -7.82070 -2.46750

H 11.66490 -7.57030 -0.93970

H 11.00210 -8.67920 -2.15600

H 10.12160 -7.78450 -4.38400

H 10.04200 -6.03470 -4.67860

H 11.60700 -6.86480 -4.64710

H 5.32760 -6.41550 1.37510

H 6.43770 -5.15270 0.83980

H 6.67520 -6.83040 0.30140

H 6.05560 -8.19940 3.03880

H 7.38620 -8.62490 1.93850

H 7.72500 -8.22010 3.63570

H 6.87750 -4.54430 3.34090

H 5.72200 -5.80340 3.78740

H 7.35960 -5.83010 4.46680

1. N111'0t_WP

N 9.53120 -3.66510 2.12850

C 9.12190 -4.96190 1.58780

C 9.11060 -4.93180 0.03540

N 9.41300 -6.10230 -0.58360

C 9.59130 -6.25190 -2.02890

C 10.83460 -7.12620 -2.22660

O 11.51180 -6.81680 -3.33330

C 12.67970 -7.62750 -3.65700

O 11.16430 -8.01910 -1.45660

O 8.85030 -3.89140 -0.59240

C 7.74440 -5.42570 2.21680

C 7.33420 -6.81890 1.69840

C 6.62290 -4.41320 1.91540

C 7.93570 -5.53020 3.74410

C 8.32170 -6.82880 -2.78010

C 8.57450 -6.83880 -4.30280

C 7.98100 -8.25420 -2.30260

C 7.12310 -5.89730 -2.50350

C 10.97350 -3.40390 2.37910

C 11.47820 -4.30670 3.52080

C 11.86370 -3.60420 1.13010

C 11.05940 -1.93450 2.82930

H 9.16700 -2.93250 1.52070

H 9.85420 -5.71980 1.88970

H 9.70760 -6.88660 -0.00970

H 13.42160 -7.54550 -2.85980

H 12.38270 -8.67010 -3.79160

H 13.06420 -7.21210 -4.58850

H 6.44990 -7.16750 2.24440

H 7.08080 -6.81340 0.63500

H 8.13100 -7.55740 1.85600

H 5.68980 -4.74050 2.38960

H 6.86880 -3.42220 2.31090

H 6.43590 -4.31830 0.84020

H 8.24760 -4.57290 4.16790

H 6.99360 -5.83360 4.21620

H 8.69340 -6.28270 3.99630

H 7.65040 -7.12470 -4.81860

H 8.86710 -5.84610 -4.66550

H 9.35140 -7.55180 -4.59520

H 7.05720 -8.58990 -2.78780

H 8.76910 -8.97170 -2.55560

H 7.81910 -8.29370 -1.22020

H 6.87260 -5.85560 -1.44050

H 7.32150 -4.87660 -2.84610

H 6.24360 -6.27320 -3.03880

H 12.51680 -4.05490 3.76350

H 10.86850 -4.16890 4.41970

H 11.45700 -5.36830 3.24960

H 12.90760 -3.35780 1.35720

H 11.84000 -4.64320 0.78210

H 11.53130 -2.96070 0.30790

H 10.45760 -1.77390 3.73030

H 12.09670 -1.65800 3.04770

H 10.68920 -1.26290 2.04450

H 9.79380 -5.26040 -2.44380

1. N0000c_GP

N 8.77670 -3.67320 2.20740

C 9.10150 -4.94150 1.58020

C 9.10840 -4.92330 0.04070

N 9.41220 -6.09880 -0.58820

C 9.72780 -7.36360 0.03740

C 10.00410 -8.40460 -1.03560

O 10.30450 -9.58920 -0.48210

C 10.58810 -10.66740 -1.40260

O 9.95870 -8.19350 -2.22940

O 8.85330 -3.90340 -0.59300

H 7.86740 -3.35530 1.87970

H 9.43440 -2.96200 1.89620

H 8.38710 -5.70190 1.92270

H 10.08620 -5.27310 1.93570

H 9.41470 -6.08420 -1.60340

H 8.90770 -7.74130 0.66310

H 10.61580 -7.30590 0.68140

H 10.80620 -11.52980 -0.77470

H 9.72050 -10.85810 -2.03790

H 11.44730 -10.41330 -2.02690

1. N0010c_GP

N 8.68150 -3.64670 2.17850

C 9.08490 -4.91160 1.58870

C 9.10800 -4.92200 0.04710

N 9.41160 -6.09660 -0.59140

C 9.84710 -7.36210 -0.01430

C 11.27340 -7.63600 -0.50400

O 12.08770 -8.00350 0.50420

C 13.45530 -8.29470 0.13990

O 11.63430 -7.52880 -1.65870

O 8.85420 -3.90690 -0.59620

C 8.86650 -8.55330 -0.33200

C 7.52750 -8.26750 0.37540

C 8.62210 -8.69800 -1.84700

C 9.46120 -9.86290 0.22380

H 7.73940 -3.41340 1.87350

H 9.27010 -2.90080 1.81440

H 8.41350 -5.69950 1.94890

H 10.08670 -5.16860 1.95690

H 9.50720 -6.00230 -1.59690

H 9.89060 -7.25830 1.07010

H 13.48990 -9.13430 -0.55820

H 13.91610 -7.42010 -0.32410

H 13.95440 -8.54770 1.07430

H 6.80860 -9.06370 0.15430

H 7.64920 -8.22490 1.46440

H 7.09110 -7.32230 0.03850

H 8.12870 -7.81270 -2.26150

H 9.55180 -8.86140 -2.39910

H 7.96240 -9.55290 -2.03140

H 8.73770 -10.67690 0.10970

H 10.37200 -10.15900 -0.30750

H 9.70470 -9.77750 1.28880

1. N0100c_GP

N 9.90610 -3.73430 2.06120

C 9.12390 -4.88510 1.59270

C 9.10850 -4.92400 0.05040

N 9.41080 -6.09310 -0.59230

C 9.80960 -7.36480 -0.02830

C 10.07260 -8.35270 -1.15430

O 10.43070 -9.54870 -0.66120

C 10.71200 -10.57910 -1.63530

O 9.97530 -8.09710 -2.33630

O 8.85460 -3.90840 -0.59860

C 7.67820 -4.86820 2.21130

C 6.95440 -3.53890 1.91730

C 7.81640 -5.04420 3.73680

C 6.84320 -6.03470 1.64580

H 10.89970 -3.90160 1.92510

H 9.66850 -2.92240 1.49380

H 9.61620 -5.78520 1.97790

H 9.40430 -6.04450 -1.60710

H 9.04000 -7.79920 0.62040

H 10.72930 -7.29390 0.56790

H 10.98070 -11.45850 -1.05210

H 9.82700 -10.77240 -2.24520

H 11.53850 -10.27260 -2.28000

H 7.48420 -2.69230 2.36500

H 6.86000 -3.35590 0.84380

H 5.94930 -3.56410 2.35380

H 6.83070 -5.00460 4.21430

H 8.26890 -6.01230 3.98690

H 8.44250 -4.25630 4.16240

H 7.31030 -7.00510 1.85570

H 5.85380 -6.04810 2.11570

H 6.69120 -5.94970 0.56460

1. N0110c_GP

N 9.90870 -3.71290 2.04740

C 9.11080 -4.86210 1.59920

C 9.10770 -4.92120 0.05700

N 9.41070 -6.09270 -0.59600

C 9.92220 -7.34740 -0.06170

C 11.44310 -7.25460 0.13880

O 11.80590 -7.69730 1.36230

C 13.22100 -7.67770 1.64960

O 12.23030 -6.83090 -0.68180

O 8.85540 -3.91150 -0.60150

C 7.66500 -4.81310 2.21370

C 6.95960 -3.47840 1.90140

C 6.81770 -5.97610 1.66230

C 7.80000 -4.97020 3.74150

C 9.52390 -8.57010 -0.96900

C 10.15110 -9.85120 -0.38080

C 7.98860 -8.70900 -0.94980

C 10.00590 -8.39520 -2.42470

H 9.68480 -2.90810 1.46480

H 10.89990 -3.89810 1.91660

H 9.59160 -5.75700 2.00570

H 9.53390 -5.94380 -1.59120

H 9.47280 -7.52380 0.91630

H 13.31310 -8.03880 2.67310

H 13.75640 -8.33230 0.95780

H 13.61220 -6.66210 1.55830

H 7.50130 -2.63300 2.33700

H 6.86760 -3.30910 0.82540

H 5.95430 -3.48350 2.33850

H 5.83360 -5.98360 2.14330

H 6.65600 -5.89270 0.58270

H 7.28460 -6.94790 1.86430

H 6.81480 -4.90800 4.21800

H 8.23710 -5.94170 4.00490

H 8.43870 -4.18660 4.15620

H 9.78940 -10.72570 -0.93140

H 11.24370 -9.84720 -0.45560

H 9.88490 -9.98440 0.67350

H 7.68240 -9.54480 -1.58820

H 7.61770 -8.90750 0.06190

H 7.49680 -7.80440 -1.31820

H 9.49610 -7.57010 -2.93460

H 11.08310 -8.21840 -2.48270

H 9.77680 -9.30300 -2.99310

1. N011'0c_GP

N 9.88050 -3.69150 2.05230

C 9.11180 -4.85600 1.59700

C 9.10820 -4.92300 0.05630

N 9.41020 -6.09090 -0.59520

C 9.76820 -7.40990 -0.09100

C 8.82550 -8.42310 -0.74860

O 8.36860 -9.33070 0.13460

C 7.48270 -10.34110 -0.39630

O 8.52350 -8.41370 -1.92490

O 8.85540 -3.91160 -0.60160

C 7.65670 -4.83530 2.19560

C 6.94170 -3.50280 1.89550

C 7.77790 -5.01320 3.72220

C 6.82540 -5.99850 1.61780

C 11.28620 -7.77130 -0.32580

C 11.54920 -9.21540 0.14700

C 12.14300 -6.80580 0.51650

C 11.67640 -7.63320 -1.81050

H 9.65480 -2.89470 1.45950

H 10.87730 -3.86170 1.94560

H 9.60530 -5.74010 2.01040

H 9.32000 -6.01580 -1.60340

H 6.59490 -9.87390 -0.82770

H 7.99270 -10.92630 -1.16500

H 7.21760 -10.96700 0.45470

H 5.92940 -3.52660 2.31520

H 7.46730 -2.65990 2.35500

H 6.86530 -3.31610 0.82110

H 8.41140 -4.23370 4.15300

H 6.78860 -4.95880 4.19080

H 8.21320 -5.98820 3.97650

H 7.30080 -6.97030 1.79850

H 5.84080 -6.02600 2.09750

H 6.66110 -5.89130 0.54020

H 11.03430 -9.95310 -0.47700

H 12.62100 -9.43330 0.09350

H 11.22670 -9.36810 1.18310

H 11.93130 -6.90850 1.58750

H 13.20640 -7.02170 0.36750

H 11.97370 -5.76420 0.22670

H 11.56780 -6.60180 -2.16120

H 12.72740 -7.91270 -1.94120

H 11.07210 -8.27760 -2.45540

H 9.58400 -7.44690 0.98220

1. N1000c_GP

N 8.41510 -3.78630 2.08140

C 9.14510 -4.92900 1.57160

C 9.10860 -4.92440 0.04160

N 9.41170 -6.09670 -0.58830

C 9.72690 -7.36720 0.02610

C 10.05690 -8.37930 -1.05900

O 10.38740 -9.56360 -0.52180

C 10.71180 -10.61770 -1.45670

O 10.01990 -8.14850 -2.24930

O 8.85350 -3.90430 -0.59380

C 8.80040 -3.22540 3.39170

C 8.64350 -4.31080 4.47170

C 7.81390 -2.07910 3.67240

C 10.24440 -2.66810 3.40850

H 8.41140 -3.06640 1.36050

H 8.71800 -5.86550 1.95310

H 10.21790 -4.94440 1.84800

H 9.41720 -6.07290 -1.60290

H 8.89010 -7.77100 0.61230

H 10.58940 -7.30420 0.70250

H 11.56960 -10.32840 -2.06760

H 10.94910 -11.48370 -0.84050

H 9.85660 -10.82370 -2.10380

H 9.35290 -5.13370 4.32780

H 8.82670 -3.89180 5.46670

H 7.62990 -4.72450 4.45070

H 6.78630 -2.45450 3.68070

H 8.02610 -1.60830 4.63820

H 7.88550 -1.30480 2.89920

H 10.36440 -1.88440 2.65190

H 10.48630 -2.23540 4.38590

H 10.98750 -3.44770 3.20720

1. N1010c_GP

N 8.30710 -3.79260 2.04680

C 9.12110 -4.89920 1.57920

C 9.10830 -4.92310 0.04810

N 9.41110 -6.09460 -0.59150

C 9.85880 -7.36600 -0.03670

C 11.21040 -7.69380 -0.67910

O 12.10460 -8.13490 0.22540

C 13.40460 -8.49440 -0.29310

O 11.45230 -7.57030 -1.86310

O 8.85450 -3.90780 -0.59710

C 8.81120 -8.52930 -0.23050

C 8.39550 -8.66900 -1.70820

C 9.42000 -9.85680 0.26420

C 7.56790 -8.20010 0.61850

C 8.58880 -3.22500 3.38090

C 10.00330 -2.60610 3.48740

C 7.53760 -2.12500 3.60720

C 8.41520 -4.32360 4.44520

H 8.31840 -3.06750 1.33150

H 8.74330 -5.84410 1.98230

H 10.18790 -4.83530 1.87080

H 9.49050 -5.99630 -1.59800

H 10.03030 -7.24900 1.03400

H 13.86940 -7.63200 -0.77550

H 13.98180 -8.81330 0.57370

H 13.31140 -9.30640 -1.01790

H 7.69170 -9.50180 -1.81320

H 7.89030 -7.76750 -2.06940

H 9.25180 -8.86550 -2.35970

H 9.78890 -9.77590 1.29300

H 10.25140 -10.18980 -0.36600

H 8.65830 -10.64320 0.24230

H 7.80780 -8.16660 1.68780

H 7.12720 -7.24020 0.33370

H 6.80410 -8.97210 0.47590

H 10.16600 -2.16980 4.47950

H 10.79030 -3.35150 3.32840

H 10.13440 -1.81240 2.74310

H 7.62040 -1.34330 2.84250

H 6.52890 -2.54530 3.55300

H 7.67160 -1.65160 4.58560

H 9.17070 -5.11120 4.34500

H 8.51460 -3.90210 5.45100

H 7.42560 -4.78400 4.35830

1. N1100c_GP

N 9.72760 -3.65990 2.08210

C 9.08580 -4.88360 1.59620

C 9.10840 -4.92400 0.05160

N 9.41060 -6.09230 -0.59290

C 9.85570 -7.34990 -0.03380

C 10.17760 -8.31600 -1.16310

O 10.55940 -9.50640 -0.67370

C 10.89590 -10.51670 -1.65110

O 10.10170 -8.05080 -2.34470

O 8.85480 -3.90930 -0.59930

C 7.61260 -4.99400 2.16190

C 7.70290 -5.12690 3.69470

C 6.90200 -6.24250 1.60110

C 6.78270 -3.74360 1.81080

C 11.19620 -3.63020 2.29350

C 11.53230 -2.17230 2.65650

C 11.56310 -4.53630 3.48280

C 12.01690 -4.03920 1.04810

H 9.47160 -2.90950 1.44300

H 9.63250 -5.74380 1.99450

H 9.42190 -6.03500 -1.60720

H 9.09680 -7.82250 0.60000

H 10.76270 -7.24400 0.57700

H 11.17640 -11.39430 -1.07060

H 10.03240 -10.72950 -2.28490

H 11.72830 -10.17830 -2.27170

H 6.69820 -5.17980 4.12980

H 8.23980 -6.03850 3.98670

H 8.22410 -4.26930 4.12660

H 5.89460 -6.31750 2.02460

H 6.79690 -6.20910 0.51170

H 7.43060 -7.16480 1.87230

H 6.70000 -3.59910 0.73020

H 5.77220 -3.84570 2.22320

H 7.22740 -2.84270 2.24390

H 12.60050 -2.06070 2.86950

H 11.28390 -1.49600 1.82990

H 10.96500 -1.85730 3.53770

H 12.63240 -4.45600 3.70590

H 10.99880 -4.24470 4.37370

H 11.35350 -5.59220 3.27930

H 11.75660 -3.41740 0.18540

H 13.09090 -3.92980 1.23780

H 11.84160 -5.08480 0.77220

1. N1110c_GP

N 9.70440 -3.58720 2.05170

C 9.09490 -4.84370 1.60740

C 9.10770 -4.92110 0.06050

N 9.41010 -6.09040 -0.59760

C 9.82200 -7.39290 -0.09420

C 11.34750 -7.44790 0.08140

O 11.68450 -7.94760 1.29050

C 13.09900 -8.09370 1.54310

O 12.15980 -7.09740 -0.74880

O 8.85610 -3.91400 -0.60340

C 7.63330 -4.95760 2.20400

C 7.75470 -5.03240 3.73870

C 6.92940 -6.23290 1.70600

C 6.77590 -3.73460 1.82230

C 9.32120 -8.56420 -1.02590

C 9.85490 -9.90310 -0.47430

C 7.78080 -8.59770 -1.00240

C 9.80270 -8.39270 -2.48240

C 11.17160 -3.47000 2.24220

C 11.63520 -4.43930 3.34400

C 11.98690 -3.70330 0.95000

C 11.40400 -2.02520 2.72280

H 9.40240 -2.85980 1.40650

H 9.66870 -5.67680 2.02270

H 9.50680 -5.94050 -1.59520

H 9.37400 -7.55090 0.88720

H 13.17190 -8.49660 2.55250

H 13.54270 -8.78070 0.81860

H 13.59800 -7.12500 1.47370

H 8.31140 -5.92470 4.05220

H 8.27060 -4.15210 4.12880

H 6.75900 -5.08480 4.19410

H 5.93620 -6.31120 2.16190

H 6.79200 -6.23360 0.62090

H 7.48230 -7.13740 1.98620

H 6.67590 -3.62910 0.73840

H 5.77260 -3.84160 2.25080

H 7.20760 -2.81080 2.21800

H 9.41990 -10.73340 -1.04010

H 10.94340 -9.98310 -0.56330

H 9.59040 -10.04040 0.57990

H 7.41910 -9.39340 -1.66260

H 7.39790 -8.79810 0.00360

H 7.35040 -7.65380 -1.34810

H 9.35110 -7.52100 -2.96930

H 10.88920 -8.29590 -2.54910

H 9.49980 -9.26800 -3.06720

H 12.69140 -4.26500 3.57580

H 11.05230 -4.28900 4.25810

H 11.53750 -5.48810 3.04420

H 13.05590 -3.53740 1.12870

H 11.87570 -4.72210 0.56850

H 11.66520 -3.01830 0.15860

H 10.85410 -1.83520 3.64950

H 12.46880 -1.84110 2.90070

H 11.06270 -1.30420 1.97020

1. N111'0c_GP

N 9.66880 -3.54870 2.04850

C 9.09650 -4.82330 1.60830

C 9.10750 -4.92050 0.06270

N 9.41000 -6.08990 -0.59870

C 9.64000 -7.45090 -0.13480

C 8.40360 -8.30630 -0.45090

O 8.16030 -9.21620 0.51300

C 7.03360 -10.09120 0.28980

O 7.73410 -8.19550 -1.45720

O 8.85640 -3.91520 -0.60450

C 7.62680 -4.95560 2.19070

C 7.73780 -5.04070 3.72550

C 6.93200 -6.23040 1.67910

C 6.76620 -3.73390 1.81270

C 10.93890 -8.09040 -0.77260

C 12.15880 -7.25750 -0.34030

C 11.11680 -9.53260 -0.25310

C 10.86400 -8.11080 -2.31330

C 11.11870 -3.39280 2.31450

C 11.99880 -3.60200 1.06280

C 11.28960 -1.94420 2.80970

C 11.55850 -4.35400 3.43310

H 9.38060 -2.83920 1.37790

H 9.68060 -5.64200 2.03830

H 9.24870 -5.99610 -1.59610

H 6.11470 -9.50730 0.20400

H 7.17880 -10.67200 -0.62410

H 6.99830 -10.74380 1.16130

H 6.73930 -5.09920 4.17400

H 8.29490 -5.93350 4.03730

H 8.24690 -4.16060 4.12500

H 5.93920 -6.31610 2.13540

H 6.78740 -6.21730 0.59380

H 7.48910 -7.13350 1.94970

H 6.67700 -3.61820 0.72890

H 5.75900 -3.85340 2.22820

H 7.18740 -2.81210 2.22330

H 13.07510 -7.70580 -0.73920

H 12.09630 -6.23380 -0.71620

H 12.25590 -7.21980 0.75110

H 12.07600 -9.92980 -0.60130

H 11.11510 -9.57160 0.84180

H 10.33280 -10.20590 -0.61170

H 9.99720 -8.66840 -2.67890

H 10.82150 -7.10090 -2.73490

H 11.76540 -8.58650 -2.71470

H 13.05650 -3.42560 1.29130

H 11.90940 -4.62210 0.68210

H 11.70310 -2.91750 0.26060

H 12.34070 -1.73040 3.03130

H 10.95770 -1.22980 2.04690

H 10.69750 -1.77450 3.71400

H 11.51700 -5.40340 3.12130

H 12.59440 -4.14550 3.72050

H 10.92400 -4.23400 4.31670

H 9.77720 -7.44210 0.94760

1. N0000c_WP

N 8.78130 -3.64290 2.18740

C 9.10240 -4.93770 1.58260

C 9.11140 -4.93560 0.04870

N 9.40950 -6.08840 -0.58840

C 9.73410 -7.38210 -0.00290

C 10.00370 -8.37130 -1.12940

O 10.31560 -9.57920 -0.66020

C 10.59990 -10.61880 -1.64280

O 9.94110 -8.08910 -2.31560

O 8.85290 -3.90150 -0.60090

H 7.86900 -3.32530 1.85120

H 9.44540 -2.93840 1.85790

H 8.38100 -5.69050 1.92850

H 10.08650 -5.27150 1.93850

H 9.40500 -6.04590 -1.60610

H 8.91330 -7.77960 0.61100

H 10.62940 -7.33760 0.63330

H 10.82190 -11.51100 -1.05750

H 9.72490 -10.77800 -2.27670

H 11.46000 -10.32820 -2.24990

1. N0010c_WP

N 8.42280 -3.72550 2.15140

C 9.11280 -4.89130 1.59100

C 9.11100 -4.93410 0.05390

N 9.40910 -6.08650 -0.59110

C 9.77240 -7.40220 -0.05900

C 11.04750 -7.84810 -0.78990

O 11.87260 -8.53260 0.00460

C 13.07300 -9.09800 -0.59810

O 11.26960 -7.61800 -1.96990

O 8.85380 -3.90490 -0.60340

C 8.60420 -8.46760 -0.20020

C 7.50420 -8.12050 0.82450

C 8.00380 -8.44480 -1.61950

C 9.12920 -9.88610 0.10810

H 7.43720 -3.74920 1.87770

H 8.79670 -2.87270 1.72920

H 8.66470 -5.79700 2.00890

H 10.16230 -4.88740 1.91850

H 9.44670 -5.99210 -1.60510

H 10.01940 -7.31240 1.00090

H 12.79980 -9.74630 -1.43390

H 13.72950 -8.29460 -0.94020

H 13.54680 -9.67250 0.19800

H 6.67710 -8.83340 0.72780

H 7.88010 -8.18510 1.85310

H 7.09300 -7.11830 0.66280

H 7.54090 -7.47860 -1.84670

H 8.75630 -8.65890 -2.38610

H 7.22340 -9.21060 -1.69320

H 8.28450 -10.58410 0.12310

H 9.83280 -10.24160 -0.65290

H 9.62450 -9.93520 1.08450

1. N0100c_WP

N 9.97910 -3.78860 2.06500

C 9.12650 -4.90200 1.59660

C 9.11120 -4.93500 0.05410

N 9.40910 -6.08650 -0.59090

C 9.80510 -7.37520 -0.03590

C 10.11700 -8.33550 -1.17500

O 10.42980 -9.54560 -0.71070

C 10.76500 -10.57520 -1.68770

O 10.08430 -8.03620 -2.35830

O 8.85350 -3.90400 -0.60370

C 7.67380 -4.85650 2.21430

C 6.99550 -3.48590 2.01380

C 7.77590 -5.14890 3.72660

C 6.78820 -5.94860 1.57370

H 10.94730 -3.96920 1.78790

H 9.71260 -2.92840 1.58180

H 9.58960 -5.82320 1.96680

H 9.40190 -6.02760 -1.60820

H 9.01500 -7.83080 0.57340

H 10.70360 -7.29900 0.59200

H 10.96100 -11.47150 -1.09920

H 9.92320 -10.73200 -2.36550

H 11.65410 -10.27730 -2.24780

H 7.55130 -2.68220 2.50870

H 6.89290 -3.23310 0.95440

H 5.99170 -3.51000 2.45520

H 6.77770 -5.12450 4.18030

H 8.20210 -6.14280 3.90920

H 8.39870 -4.40910 4.23760

H 7.21480 -6.94950 1.71150

H 5.80120 -5.94950 2.05080

H 6.63300 -5.78290 0.50160

1. N0110c_WP

N 9.99280 -3.73140 2.02170

C 9.15770 -4.87600 1.60100

C 9.11040 -4.93190 0.05660

N 9.40930 -6.08750 -0.59290

C 9.71550 -7.40960 -0.04710

C 11.18350 -7.49000 0.41110

O 11.29940 -8.23650 1.51640

C 12.63360 -8.47170 2.04820

O 12.12320 -6.94460 -0.14580

O 8.85410 -3.90610 -0.60420

C 7.73400 -4.85630 2.27810

C 7.00480 -3.51580 2.05790

C 6.85490 -5.99680 1.71870

C 7.91610 -5.09200 3.79300

C 9.33890 -8.54540 -1.07850

C 9.63590 -9.92680 -0.45580

C 7.82320 -8.45770 -1.36400

C 10.12710 -8.40860 -2.39790

H 9.66120 -2.88250 1.55910

H 10.94920 -3.86270 1.68300

H 9.65770 -5.77420 1.97610

H 9.45760 -5.97390 -1.60290

H 9.09890 -7.58530 0.83580

H 12.48460 -9.12180 2.91100

H 13.25440 -8.96430 1.29610

H 13.08630 -7.52500 2.35210

H 7.55750 -2.67450 2.48990

H 6.84030 -3.31260 0.99540

H 6.02610 -3.55170 2.55140

H 5.87990 -5.98620 2.21980

H 6.67080 -5.89480 0.64330

H 7.29800 -6.98230 1.90590

H 6.93870 -5.09350 4.29000

H 8.39110 -6.06120 3.98850

H 8.52950 -4.31150 4.25190

H 9.26830 -10.70800 -1.13050

H 10.70740 -10.09670 -0.30770

H 9.13170 -10.05150 0.50970

H 7.54190 -9.24940 -2.06740

H 7.23640 -8.59990 -0.44880

H 7.53810 -7.49910 -1.80870

H 9.91200 -7.47280 -2.92820

H 11.20790 -8.46880 -2.23390

H 9.84320 -9.22660 -3.06970

1. N011'0c_WP

N 9.94040 -3.66900 2.01030

C 9.15890 -4.85620 1.60430

C 9.11060 -4.93260 0.05970

N 9.40880 -6.08540 -0.59410

C 9.65330 -7.44710 -0.10400

C 8.82700 -8.38140 -1.00360

O 8.28260 -9.39250 -0.32560

C 7.53310 -10.38470 -1.08700

O 8.69440 -8.21590 -2.20760

O 8.85440 -3.90750 -0.60610

C 7.73920 -4.88930 2.28800

C 6.95090 -3.58570 2.05540

C 7.94270 -5.09360 3.80520

C 6.91000 -6.07700 1.75240

C 11.18140 -7.85980 -0.06810

C 11.30410 -9.34900 0.32230

C 11.89650 -7.01820 1.00880

C 11.86170 -7.62080 -1.42900

H 9.60050 -2.85170 1.49930

H 10.91400 -3.78040 1.71730

H 9.69760 -5.72630 1.98490

H 9.37850 -5.99100 -1.60870

H 6.68210 -9.90980 -1.58030

H 8.18560 -10.85440 -1.82690

H 7.19680 -11.11380 -0.34980

H 5.98260 -3.65100 2.56600

H 7.47710 -2.71460 2.46060

H 6.76090 -3.41070 0.99220

H 8.51510 -4.27390 4.24820

H 6.97030 -5.14250 4.30940

H 8.47270 -6.03180 4.01110

H 7.40040 -7.03730 1.95050

H 5.93790 -6.10440 2.25800

H 6.71690 -6.00020 0.67640

H 10.92230 -10.01650 -0.45770

H 12.36070 -9.59510 0.47740

H 10.76810 -9.56890 1.25320

H 11.47070 -7.18920 2.00490

H 12.95410 -7.30190 1.04940

H 11.85570 -5.94720 0.78500

H 11.82220 -6.56510 -1.71920

H 12.91750 -7.90600 -1.35950

H 11.40550 -8.21690 -2.22590

H 9.26450 -7.54680 0.90970

1. N1000c_WP

N 8.66480 -3.69770 2.13700

C 9.14120 -4.95960 1.57110

C 9.11110 -4.93440 0.04630

N 9.41040 -6.09170 -0.58760

C 9.74580 -7.36410 0.03540

C 10.31580 -8.31360 -1.00690

O 10.66540 -9.47530 -0.45210

C 11.22860 -10.50080 -1.32200

O 10.43200 -8.04780 -2.19280

O 8.85230 -3.89950 -0.59920

C 8.78950 -3.55020 3.62000

C 7.84020 -4.54160 4.31750

C 8.35220 -2.11140 3.94570

C 10.23270 -3.76470 4.13180

H 9.18570 -2.93710 1.69560

H 8.51080 -5.78750 1.91830

H 10.17620 -5.21460 1.86000

H 9.42370 -6.05810 -1.60550

H 8.86940 -7.84880 0.49070

H 10.49840 -7.24250 0.82500

H 12.15110 -10.13440 -1.77730

H 11.43150 -11.34740 -0.66580

H 10.50380 -10.77250 -2.09240

H 8.12870 -5.58270 4.13610

H 7.86270 -4.38000 5.40080

H 6.80910 -4.40260 3.97330

H 7.32400 -1.93080 3.61260

H 8.40060 -1.93270 5.02540

H 9.00770 -1.38110 3.45460

H 10.93350 -3.09330 3.62080

H 10.28630 -3.55390 5.20610

H 10.57300 -4.79540 3.98470

1. N1010c_WP

N 8.34370 -3.80580 2.11590

C 9.13900 -4.91290 1.58020

C 9.11070 -4.93270 0.05190

N 9.41000 -6.09000 -0.59060

C 9.77550 -7.39150 -0.02500

C 11.07320 -7.84800 -0.70630

O 11.88420 -8.48530 0.14200

C 13.11240 -9.05650 -0.39380

O 11.32680 -7.66490 -1.88740

O 8.85320 -3.90280 -0.60180

C 8.62190 -8.47140 -0.14840

C 8.14160 -8.61430 -1.60570

C 9.12050 -9.83840 0.36770

C 7.43900 -8.02320 0.73530

C 8.08650 -3.84350 3.58990

C 9.37150 -4.04930 4.42310

C 7.45890 -2.48640 3.95200

C 7.07720 -4.96380 3.90750

H 8.82360 -2.93170 1.89480

H 8.74710 -5.85050 1.97580

H 10.20510 -4.87770 1.86790

H 9.44620 -6.00780 -1.60550

H 10.00040 -7.26960 1.03570

H 13.75100 -8.26330 -0.78870

H 13.58790 -9.55070 0.45370

H 12.87450 -9.77830 -1.17860

H 7.34170 -9.36200 -1.65030

H 7.73430 -7.67380 -1.99320

H 8.94520 -8.94540 -2.27140

H 9.53210 -9.76300 1.38110

H 9.88610 -10.27580 -0.28180

H 8.27780 -10.53860 0.40000

H 7.72080 -7.99000 1.79460

H 7.05410 -7.04120 0.44130

H 6.61810 -8.74250 0.63610

H 9.14290 -3.98990 5.49340

H 9.82280 -5.03020 4.23730

H 10.11620 -3.27790 4.19250

H 8.15240 -1.66290 3.74080

H 6.53780 -2.31820 3.38260

H 7.21330 -2.45380 5.01890

H 7.48320 -5.95970 3.69920

H 6.81390 -4.93810 4.97080

H 6.15910 -4.83540 3.32370

1. N1100c_WP

N 9.68940 -3.57380 2.05240

C 9.10980 -4.84770 1.61430

C 9.11020 -4.93130 0.06300

N 9.40850 -6.08420 -0.59590

C 9.75240 -7.40060 -0.09000

C 8.68810 -8.45180 -0.41760

O 8.84670 -9.54320 0.34020

C 7.93470 -10.65870 0.11780

O 7.82740 -8.33010 -1.27280

O 8.85510 -3.91000 -0.60760

C 7.66550 -5.04250 2.23980

C 7.81690 -5.07790 3.77470

C 7.04050 -6.37590 1.78760

C 6.72730 -3.88140 1.85970

C 11.12420 -3.49860 2.43400

C 11.41250 -2.00720 2.68640

C 11.36240 -4.27500 3.74380

C 12.08140 -4.02440 1.33970

H 9.48410 -2.86090 1.35470

H 9.72140 -5.66070 2.01540

H 9.40740 -5.98470 -1.61060

H 9.91760 -7.38470 0.98790

H 10.69000 -7.74530 -0.55070

H 8.23900 -11.42150 0.83460

H 6.90690 -10.34100 0.30660

H 8.03620 -11.02610 -0.90570

H 6.83520 -5.21600 4.24330

H 8.45890 -5.90890 4.09240

H 8.25230 -4.14770 4.14730

H 6.06580 -6.51130 2.27020

H 6.87600 -6.41220 0.70590

H 7.66460 -7.22960 2.07960

H 6.54120 -3.84240 0.78160

H 5.76140 -4.00670 2.36430

H 7.14730 -2.92010 2.17240

H 12.45000 -1.86510 3.00830

H 11.25820 -1.41660 1.77470

H 10.75050 -1.61570 3.46650

H 12.40800 -4.16980 4.05540

H 10.72240 -3.88720 4.54280

H 11.16120 -5.34640 3.63250

H 11.93270 -3.48260 0.39840

H 13.12590 -3.89330 1.64690

H 11.92940 -5.09350 1.15030

1. N1110c_WP

N 9.78720 -3.64260 2.07590

C 9.08950 -4.84730 1.61290

C 9.10970 -4.92940 0.06510

N 9.40890 -6.08550 -0.59730

C 9.85440 -7.39320 -0.11210

C 11.39160 -7.47700 -0.15740

O 11.88620 -8.07880 0.93130

C 13.32040 -8.32860 0.97520

O 12.07980 -7.06440 -1.07800

O 8.85520 -3.91050 -0.60830

C 7.62050 -4.88360 2.21330

C 7.74840 -5.01610 3.74530

C 6.83630 -6.09920 1.69060

C 6.83700 -3.59830 1.88670

C 9.21240 -8.58880 -0.93050

C 9.80680 -9.92340 -0.42750

C 7.69140 -8.61490 -0.68330

C 9.47170 -8.45850 -2.44600

C 11.22310 -3.68080 2.45390

C 11.44040 -4.64120 3.63870

C 12.15330 -4.07220 1.28470

C 11.56930 -2.25060 2.90900

H 9.63050 -2.88770 1.41040

H 9.60070 -5.72210 2.02200

H 9.45540 -5.94370 -1.60490

H 9.54660 -7.51530 0.92780

H 13.49580 -8.81180 1.93660

H 13.60670 -8.98880 0.15280

H 13.86800 -7.38630 0.90960

H 8.26260 -5.94520 4.02170

H 8.30700 -4.17640 4.16600

H 6.75220 -5.03600 4.20320

H 5.84990 -6.13720 2.16790

H 6.67530 -6.05430 0.60900

H 7.34760 -7.03920 1.92760

H 6.67780 -3.48180 0.81050

H 5.85430 -3.63660 2.37240

H 7.36020 -2.71170 2.25780

H 9.26840 -10.75480 -0.89560

H 10.86600 -10.03460 -0.68470

H 9.70440 -10.02620 0.65920

H 7.24580 -9.43150 -1.26250

H 7.45940 -8.79050 0.37250

H 7.21140 -7.68270 -0.99420

H 8.98820 -7.57350 -2.87680

H 10.53940 -8.42020 -2.68140

H 9.04720 -9.33180 -2.95390

H 12.48430 -4.59200 3.96730

H 10.79940 -4.36510 4.48200

H 11.23320 -5.68490 3.37800

H 13.20510 -4.03920 1.59310

H 11.94860 -5.08700 0.93220

H 12.02210 -3.38780 0.43850

H 10.94700 -1.95770 3.76120

H 12.62190 -2.18660 3.20580

H 11.40260 -1.53090 2.09780

1. N111'0c_WP

N 9.66800 -3.56240 2.06820

C 9.11770 -4.84190 1.61400

C 9.10980 -4.92960 0.06480

N 9.40880 -6.08520 -0.59720

C 9.60260 -7.45730 -0.12470

C 8.40100 -8.29840 -0.59270

O 8.09360 -9.26800 0.27180

C 7.01090 -10.17390 -0.08420

O 7.80740 -8.10900 -1.64410

O 8.85530 -3.91070 -0.60820

C 7.67010 -5.03810 2.24200

C 7.84190 -5.16880 3.76940

C 6.98590 -6.31690 1.72500

C 6.76210 -3.83160 1.93520

C 10.97310 -8.08850 -0.60590

C 12.12530 -7.22600 -0.05810

C 11.12170 -9.51460 -0.03180

C 11.06850 -8.14090 -2.14370

C 11.10910 -3.38360 2.37910

C 12.01990 -3.48330 1.13580

C 11.22900 -1.96420 2.96770

C 11.56230 -4.39790 3.44540

H 9.37330 -2.82880 1.42630

H 9.73560 -5.65170 2.01150

H 9.31050 -5.98270 -1.60590

H 6.07970 -9.61480 -0.20030

H 7.25380 -10.69910 -1.01090

H 6.94090 -10.87340 0.74910

H 6.86390 -5.29280 4.24960

H 8.45420 -6.04310 4.02420

H 8.32440 -4.28050 4.18410

H 6.01070 -6.43370 2.21160

H 6.80330 -6.28330 0.64520

H 7.56960 -7.21330 1.95840

H 6.59050 -3.71630 0.85990

H 5.78790 -3.97380 2.41820

H 7.19410 -2.90240 2.31840

H 13.08430 -7.68190 -0.32880

H 12.10530 -6.21790 -0.47920

H 12.08830 -7.15130 1.03530

H 12.12400 -9.89120 -0.26530

H 11.00470 -9.52320 1.05840

H 10.39750 -10.21620 -0.45670

H 10.29070 -8.77400 -2.58300

H 10.99970 -7.14440 -2.59530

H 12.03960 -8.56010 -2.43010

H 13.06890 -3.31000 1.40540

H 11.95650 -4.47130 0.67410

H 11.73160 -2.73760 0.38580

H 12.27290 -1.73510 3.21000

H 10.87730 -1.21330 2.24910

H 10.62920 -1.87360 3.87930

H 11.54910 -5.43000 3.07850

H 12.59150 -4.17570 3.74910

H 10.92190 -4.34050 4.33170

H 9.61330 -7.46450 0.96620

1. N0000_Anti120GP

N 8.78610 -3.74660 2.27810

C 9.11430 -4.96140 1.55800

C 9.10040 -4.89210 0.03860

N 9.42600 -6.15290 -0.59610

C 8.33580 -6.67700 -1.42700

C 8.89840 -7.65100 -2.44430

O 7.94660 -8.48100 -2.90440

C 8.35320 -9.40210 -3.93970

O 10.05160 -7.65380 -2.82390

O 8.84740 -3.88050 -0.58300

H 7.86240 -3.41380 2.01370

H 9.43530 -3.00310 2.03430

H 8.43280 -5.77250 1.84980

H 10.10900 -5.32080 1.85360

H 10.22970 -6.00680 -1.21160

H 7.81630 -5.88990 -1.99880

H 7.59000 -7.18700 -0.80990

H 7.45940 -9.97610 -4.18060

H 8.71010 -8.85420 -4.81470

H 9.14710 -10.05570 -3.57200

1. N0010_Anti120GP

N 8.95610 -3.71310 2.26990

C 9.25490 -4.93170 1.54410

C 9.10100 -4.89420 0.02810

N 9.42730 -6.15790 -0.59090

C 8.40590 -6.91730 -1.35070

C 9.02610 -8.31460 -1.44170

O 8.23480 -9.25670 -0.89160

C 8.74910 -10.60510 -0.91310

O 10.11050 -8.55230 -1.93560

O 8.84560 -3.87340 -0.57770

C 7.93290 -6.40030 -2.76810

C 6.85720 -5.30750 -2.59620

C 9.11260 -5.84750 -3.58860

C 7.28580 -7.57990 -3.53130

H 7.98040 -3.45460 2.14910

H 9.50250 -2.93890 1.90110

H 8.64490 -5.76130 1.92560

H 10.29120 -5.24270 1.73620

H 10.24640 -6.02940 -1.18470

H 7.52020 -6.99850 -0.71290

H 8.90350 -10.93630 -1.94270

H 9.69660 -10.65610 -0.37200

H 7.98910 -11.21160 -0.42190

H 6.48870 -4.99480 -3.58000

H 5.99750 -5.68610 -2.02890

H 7.24830 -4.42490 -2.08880

H 9.52300 -4.94040 -3.13380

H 9.91330 -6.58680 -3.69300

H 8.76920 -5.58020 -4.59410

H 6.84550 -7.21550 -4.46550

H 8.01590 -8.35260 -3.79360

H 6.48430 -8.04760 -2.94790

1. N0100_Anti120GP

N 10.61200 -5.26030 1.91720

C 9.20300 -4.98640 1.57570

C 9.09930 -4.88780 0.05040

N 9.42590 -6.15230 -0.60250

C 8.34390 -6.62880 -1.47150

C 8.90590 -7.59770 -2.49340

O 7.94600 -8.39790 -2.98880

C 8.35410 -9.30990 -4.03110

O 10.06700 -7.62170 -2.84840

O 8.84870 -3.88540 -0.58840

C 8.61730 -3.80680 2.40300

C 9.42190 -2.50510 2.20430

C 8.66590 -4.20560 3.89370

C 7.14490 -3.57580 2.00270

H 10.90420 -6.14740 1.51600

H 11.22920 -4.53350 1.56370

H 8.64650 -5.89180 1.85180

H 10.23900 -6.00200 -1.20420

H 7.86130 -5.81530 -2.03850

H 7.56830 -7.12930 -0.88380

H 7.45390 -9.86180 -4.29840

H 8.73850 -8.75530 -4.89010

H 9.12770 -9.98530 -3.65970

H 10.45560 -2.61580 2.55130

H 9.43110 -2.19180 1.15740

H 8.97270 -1.69860 2.79480

H 8.26840 -3.39350 4.51280

H 8.05760 -5.09820 4.08550

H 9.68850 -4.42010 4.21320

H 6.56130 -4.50160 2.08890

H 6.68720 -2.83980 2.67310

H 7.05360 -3.20370 0.97960

1. N0110_Anti120GP

N 10.76100 -5.11850 1.79710

C 9.31550 -4.95210 1.55970

C 9.10000 -4.89040 0.03950

N 9.42720 -6.15740 -0.59700

C 8.41440 -6.86250 -1.41870

C 8.98590 -8.28150 -1.49600

O 8.13380 -9.19940 -0.99770

C 8.59770 -10.56590 -1.01000

O 10.08540 -8.55580 -1.93550

O 8.84670 -3.87780 -0.58300

C 8.71260 -3.79510 2.40800

C 9.43390 -2.45330 2.16250

C 7.21150 -3.64780 2.08360

C 8.86130 -4.17810 3.89620

C 8.03160 -6.31630 -2.85360

C 7.40670 -7.47210 -3.67080

C 6.96690 -5.20640 -2.72930

C 9.26680 -5.77600 -3.59700

H 11.30140 -4.37030 1.37040

H 11.08510 -6.00510 1.42120

H 8.84630 -5.88490 1.89610

H 10.26760 -6.03650 -1.16070

H 7.49540 -6.91730 -0.82690

H 7.79210 -11.14800 -0.56410

H 8.79040 -10.89250 -2.03460

H 9.51450 -10.65870 -0.42370

H 10.48780 -2.50670 2.45810

H 9.37440 -2.14810 1.11500

H 8.97130 -1.66820 2.77140

H 6.74740 -2.93570 2.77490

H 7.04760 -3.28460 1.06620

H 6.68520 -4.60430 2.19920

H 8.46540 -3.37840 4.53240

H 8.30400 -5.09410 4.12810

H 9.90920 -4.34480 4.15800

H 7.02710 -7.08520 -4.62230

H 8.13510 -8.25570 -3.90340

H 6.56420 -7.93140 -3.14100

H 6.66280 -4.87370 -3.72860

H 6.06890 -5.57760 -2.21910

H 7.34260 -4.33970 -2.18510

H 9.66780 -4.88400 -3.10530

H 10.05740 -6.53020 -3.66750

H 8.98730 -5.48760 -4.61640

1. N011'0_Anti120GP

N 10.60650 -5.22390 1.92610

C 9.19300 -4.98470 1.57490

C 9.09930 -4.88790 0.04780

N 9.42650 -6.15480 -0.60140

C 8.33470 -6.62270 -1.48500

C 8.41850 -5.90980 -2.83860

O 7.21270 -5.51720 -3.28550

C 7.20180 -4.81650 -4.54710

O 9.45730 -5.72880 -3.44500

O 8.84800 -3.88280 -0.58690

C 8.57460 -3.81780 2.39710

C 9.35450 -2.49950 2.20990

C 8.61420 -4.21770 3.88780

C 7.10250 -3.61650 1.98010

C 8.35240 -8.18680 -1.62460

C 7.25770 -8.63100 -2.61590

C 8.04590 -8.78820 -0.23900

C 9.72480 -8.69250 -2.10940

H 11.20900 -4.48710 1.56790

H 10.92190 -6.10810 1.53670

H 8.65560 -5.90120 1.84930

H 10.23840 -5.99200 -1.20160

H 7.81080 -3.91280 -4.47740

H 7.59210 -5.45670 -5.34180

H 6.15720 -4.56710 -4.72940

H 8.88150 -1.70340 2.79580

H 10.38580 -2.58980 2.56970

H 9.37050 -2.18500 1.16340

H 9.63700 -4.41420 4.21810

H 8.19600 -3.41370 4.50400

H 8.01970 -5.12110 4.07240

H 6.53690 -4.55410 2.05890

H 6.62180 -2.89060 2.64540

H 7.01560 -3.24580 0.95610

H 7.46960 -8.30340 -3.63890

H 7.19150 -9.72420 -2.62860

H 6.27440 -8.23800 -2.33450

H 7.04820 -8.49570 0.11080

H 8.07260 -9.88240 -0.28800

H 8.78010 -8.46050 0.50180

H 10.51440 -8.44400 -1.39320

H 9.70160 -9.78320 -2.20990

H 9.99700 -8.26830 -3.08030

H 7.37850 -6.35000 -1.02730

1. N1000_Anti120GP

N 8.62350 -3.75210 2.15810

C 9.14490 -4.95390 1.54530

C 9.10060 -4.89290 0.03840

N 9.42580 -6.15220 -0.59580

C 8.34710 -6.65540 -1.45530

C 8.92610 -7.60690 -2.48400

O 7.98770 -8.44150 -2.96310

C 8.41340 -9.34340 -4.00710

O 10.08210 -7.59030 -2.85600

O 8.84740 -3.88040 -0.58310

C 9.06030 -3.40460 3.52650

C 8.64490 -4.53320 4.48730

C 8.31390 -2.11310 3.90080

C 10.58460 -3.15910 3.62960

H 8.74900 -2.96810 1.52210

H 8.58430 -5.84920 1.84880

H 10.20110 -5.18830 1.78340

H 10.24200 -6.00740 -1.19520

H 7.83930 -5.85400 -2.01720

H 7.59060 -7.17630 -0.86090

H 9.20990 -9.99490 -3.64070

H 7.52740 -9.92240 -4.26500

H 8.77390 -8.77990 -4.87090

H 9.17510 -5.46740 4.27180

H 8.87420 -4.25830 5.52220

H 7.56960 -4.72330 4.40880

H 7.23220 -2.26750 3.84520

H 8.57280 -1.79320 4.91550

H 8.57640 -1.29710 3.21650

H 10.89370 -2.35300 2.95420

H 10.86610 -2.87120 4.64870

H 11.16250 -4.05460 3.37590

1. N1010_Anti120GP

N 8.75720 -3.72770 2.16150

C 9.24190 -4.93570 1.53170

C 9.10130 -4.89530 0.02700

N 9.42710 -6.15740 -0.59010

C 8.42850 -6.88220 -1.40960

C 9.03010 -8.28730 -1.49970

O 8.18930 -9.23020 -1.03140

C 8.67930 -10.58700 -1.06380

O 10.14080 -8.53060 -1.92900

O 8.84540 -3.87280 -0.57740

C 8.02860 -6.33390 -2.83920

C 9.25090 -5.76670 -3.58350

C 7.42040 -7.49370 -3.66250

C 6.94550 -5.24400 -2.69870

C 9.25700 -3.37790 3.50740

C 10.78320 -3.12590 3.53620

C 8.52380 -2.08990 3.91770

C 8.89410 -4.50720 4.48820

H 8.86090 -2.94740 1.51710

H 8.70920 -5.82720 1.88940

H 10.31440 -5.16100 1.69710

H 10.28860 -6.05750 -1.12590

H 7.51420 -6.95880 -0.81340

H 9.58820 -10.67480 -0.46430

H 7.87780 -11.19330 -0.64320

H 8.89460 -10.88890 -2.09160

H 8.96140 -5.47320 -4.59870

H 9.64060 -4.87390 -3.08460

H 10.05320 -6.50750 -3.66510

H 6.58820 -7.97170 -3.13330

H 8.16160 -8.26280 -3.90420

H 7.03090 -7.10600 -4.60980

H 6.05570 -5.63640 -2.19010

H 7.30670 -4.37650 -2.14550

H 6.63270 -4.90480 -3.69320

H 11.11450 -2.84190 4.54140

H 11.35070 -4.01750 3.24820

H 11.05470 -2.31490 2.85070

H 8.74530 -1.27410 3.21900

H 7.44170 -2.25070 3.91870

H 8.83320 -1.76600 4.91700

H 9.42420 -5.43680 4.25410

H 9.16380 -4.22550 5.51140

H 7.81850 -4.70830 4.45600

1. N1100_Anti120GP

N 10.01280 -3.72750 2.03890

C 9.20170 -4.86370 1.59000

C 9.09980 -4.89010 0.05260

N 9.42470 -6.14760 -0.60290

C 8.31830 -6.75980 -1.34990

C 8.88000 -7.59670 -2.48420

O 7.97810 -8.49430 -2.91840

C 8.37650 -9.30050 -4.04790

O 9.98680 -7.45370 -2.96310

O 8.84930 -3.88780 -0.59020

C 7.78900 -4.85310 2.29270

C 8.01460 -5.08490 3.79920

C 6.90900 -5.99240 1.74260

C 7.06730 -3.50790 2.08760

C 11.48770 -3.87560 2.13920

C 12.02900 -2.46840 2.44620

C 11.82700 -4.81090 3.31420

C 12.14930 -4.40490 0.84630

H 9.79250 -2.92170 1.45820

H 9.69340 -5.79220 1.88750

H 10.13820 -5.92620 -1.30270

H 7.65010 -6.01490 -1.81420

H 7.70930 -7.39670 -0.70450

H 7.52700 -9.95160 -4.25000

H 8.59180 -8.66480 -4.90980

H 9.26390 -9.88650 -3.79870

H 7.05960 -5.04070 4.33540

H 8.45860 -6.06980 3.98920

H 8.67970 -4.32260 4.21210

H 5.99040 -6.07560 2.33380

H 6.60470 -5.81190 0.70590

H 7.42250 -6.96010 1.79100

H 6.90980 -3.28510 1.02800

H 6.08690 -3.53600 2.57700

H 7.63620 -2.68540 2.53120

H 13.11480 -2.49280 2.58270

H 11.81210 -1.77560 1.62390

H 11.57220 -2.07150 3.35810

H 12.91230 -4.87480 3.44700

H 11.38460 -4.43610 4.24210

H 11.46000 -5.82910 3.14800

H 11.91950 -3.75590 -0.00640

H 13.23920 -4.44120 0.95430

H 11.81310 -5.42070 0.61100

1. N1110_Anti120GP

N 10.02910 -3.67690 1.99590

C 9.26210 -4.85320 1.57740

C 9.10040 -4.89200 0.04120

N 9.42620 -6.15380 -0.59730

C 8.45090 -6.94370 -1.38630

C 9.18010 -8.27750 -1.57210

O 8.46520 -9.31590 -1.09540

C 9.07770 -10.61640 -1.21980

O 10.27990 -8.39560 -2.07640

O 8.84720 -3.87970 -0.58440

C 7.87670 -4.90960 2.33310

C 8.16910 -5.13680 3.82870

C 7.02420 -6.08170 1.81070

C 7.09210 -3.59470 2.16390

C 7.91420 -6.38500 -2.76500

C 7.32530 -7.56170 -3.57800

C 6.77480 -5.37660 -2.50930

C 9.03470 -5.71210 -3.57850

C 11.51100 -3.75380 2.05800

C 11.92560 -4.65360 3.23670

C 12.16410 -4.27230 0.75600

C 11.99450 -2.31830 2.32900

H 9.75530 -2.88820 1.41450

H 9.80690 -5.75690 1.85750

H 10.24560 -5.99360 -1.18390

H 7.58690 -7.13920 -0.74920

H 8.36960 -11.31300 -0.77230

H 9.24570 -10.85840 -2.27200

H 10.03200 -10.63750 -0.68870

H 8.65920 -6.10350 3.99670

H 8.81760 -4.34920 4.21990

H 7.23430 -5.13320 4.40110

H 6.13770 -6.21060 2.44130

H 6.66480 -5.90160 0.79170

H 7.58000 -7.02620 1.82040

H 6.88800 -3.37120 1.11210

H 6.13060 -3.66850 2.68510

H 7.64030 -2.75170 2.59470

H 6.83580 -7.17770 -4.47930

H 8.09730 -8.26750 -3.90180

H 6.57420 -8.11490 -3.00290

H 6.36570 -5.03070 -3.46550

H 5.95310 -5.84220 -1.95060

H 7.12030 -4.50090 -1.95850

H 9.40050 -4.81030 -3.07790

H 9.87640 -6.39220 -3.74440

H 8.64870 -5.40980 -4.55870

H 13.01630 -4.67050 3.33700

H 11.49630 -4.28140 4.17170

H 11.59560 -5.68850 3.09860

H 13.25680 -4.25250 0.83490

H 11.87530 -5.30870 0.54880

H 11.87900 -3.65160 -0.10080

H 11.54070 -1.92710 3.24490

H 13.08320 -2.29090 2.44070

H 11.72710 -1.65040 1.50110

1. N111'0_Anti120GP

N 10.19430 -3.83830 2.00050

C 9.22620 -4.86310 1.58860

C 9.09960 -4.88910 0.05010

N 9.42610 -6.15290 -0.60210

C 8.30940 -6.76710 -1.35820

C 8.12300 -6.04660 -2.69810

O 6.83180 -5.75190 -2.93870

C 6.56060 -5.05340 -4.17200

O 9.03080 -5.77650 -3.46050

O 8.84820 -3.88350 -0.58850

C 7.84850 -4.65410 2.32670

C 8.08120 -4.91310 3.82820

C 6.80420 -5.66170 1.80840

C 7.31080 -3.22310 2.13820

C 8.53380 -8.31110 -1.53910

C 8.47470 -8.96820 -0.14600

C 7.40430 -8.89210 -2.41450

C 9.89840 -8.61510 -2.18710

C 11.63770 -4.16700 2.11140

C 12.24020 -4.77260 0.82330

C 12.34390 -2.83600 2.42450

C 11.85370 -5.13670 3.28780

H 10.07260 -3.01390 1.41770

H 9.60160 -5.84250 1.89110

H 10.13930 -5.92330 -1.29900

H 7.10030 -4.10430 -4.19050

H 6.86480 -5.66050 -5.02810

H 5.48370 -4.88810 -4.17910

H 7.15630 -4.73800 4.38970

H 8.39170 -5.94900 4.01230

H 8.85410 -4.24750 4.22030

H 5.89990 -5.61580 2.42530

H 6.49880 -5.44130 0.77960

H 7.17860 -6.69140 1.84860

H 7.15180 -2.98290 1.08330

H 6.35230 -3.11810 2.65960

H 8.00020 -2.48590 2.56060

H 8.66250 -10.04450 -0.22910

H 9.22730 -8.53900 0.52060

H 7.48860 -8.83790 0.31560

H 7.48890 -9.98350 -2.45300

H 6.41500 -8.64640 -2.01240

H 7.45040 -8.52370 -3.44490

H 9.99880 -8.14410 -3.16900

H 10.72270 -8.27100 -1.55430

H 10.01160 -9.69740 -2.31510

H 13.31790 -4.93640 0.93680

H 11.78430 -5.73980 0.58850

H 12.09290 -4.10150 -0.03100

H 13.41830 -2.99210 2.56460

H 12.21580 -2.12010 1.60340

H 11.93490 -2.38870 3.33570

H 11.37040 -6.10420 3.11740

H 12.92290 -5.32830 3.42850

H 11.45260 -4.71300 4.21330

H 7.38870 -6.64220 -0.78530

1. N0000_Anti-120GP

N 8.79550 -3.74440 2.27830

C 9.09680 -4.96590 1.55790

C 9.10040 -4.89210 0.03870

N 9.42600 -6.15280 -0.59620

C 10.64950 -6.09040 -1.40420

C 10.63700 -7.20920 -2.42800

O 11.87750 -7.48470 -2.86570

C 11.97750 -8.48290 -3.90460

O 9.63120 -7.75740 -2.83010

O 8.84740 -3.88050 -0.58300

H 7.87590 -3.39690 2.01860

H 9.45540 -3.01180 2.02980

H 8.38790 -5.75640 1.83830

H 10.07700 -5.35640 1.86470

H 8.66160 -6.40190 -1.22840

H 11.53660 -6.18750 -0.77110

H 10.74230 -5.14780 -1.96930

H 13.04100 -8.56130 -4.12600

H 11.58340 -9.43750 -3.54920

H 11.41930 -8.16630 -4.78850

1. N0010_Anti-120GP

N 8.81270 -3.74140 2.27730

C 9.11140 -4.96340 1.55630

C 9.10040 -4.89200 0.03570

N 9.42680 -6.15600 -0.59490

C 10.64670 -6.08460 -1.43040

C 10.30160 -5.50580 -2.80640

O 11.21010 -4.60790 -3.22630

C 10.95290 -3.99950 -4.50970

O 9.32110 -5.82820 -3.44940

O 8.84660 -3.87760 -0.58130

C 11.35020 -7.48540 -1.52480

C 11.84900 -7.85850 -0.11490

C 10.37680 -8.56940 -2.02630

C 12.55950 -7.39390 -2.47790

H 7.89130 -3.39570 2.02160

H 9.46970 -3.00860 2.02160

H 8.40790 -5.75570 1.84550

H 10.09640 -5.34790 1.85410

H 8.65090 -6.40250 -1.21360

H 11.35280 -5.39870 -0.95200

H 10.93450 -4.75960 -5.29440

H 9.99470 -3.47570 -4.48950

H 11.77370 -3.30080 -4.66730

H 12.32500 -8.84520 -0.13190

H 12.59220 -7.13790 0.24860

H 11.02040 -7.88960 0.59750

H 9.53790 -8.69850 -1.33500

H 9.97380 -8.33150 -3.01500

H 10.89890 -9.53010 -2.09500

H 13.11800 -8.33580 -2.45820

H 12.25510 -7.21500 -3.51460

H 13.24520 -6.59050 -2.18580

1. N0100_Anti-120GP

N 9.57920 -3.64450 2.14720

C 9.11860 -4.91310 1.57860

C 9.10010 -4.89110 0.04680

N 9.42530 -6.15010 -0.60000

C 10.61450 -6.05120 -1.45590

C 10.58770 -7.16300 -2.48670

O 11.81770 -7.41330 -2.96890

C 11.89890 -8.40180 -4.01820

O 9.57920 -7.72780 -2.85890

O 8.84840 -3.88430 -0.58720

C 7.71790 -5.31980 2.18410

C 6.69860 -4.17760 2.00370

C 7.92470 -5.59000 3.68750

C 7.17320 -6.59860 1.51710

H 10.58190 -3.54210 2.01690

H 9.14180 -2.86700 1.65760

H 9.82390 -5.69890 1.87020

H 8.64500 -6.40950 -1.20850

H 11.52830 -6.13170 -0.85930

H 10.66220 -5.10260 -2.01640

H 12.95590 -8.46360 -4.27350

H 11.52940 -9.36460 -3.65870

H 11.30900 -8.08640 -4.88180

H 7.00370 -3.27950 2.54930

H 6.56130 -3.91180 0.95020

H 5.72450 -4.48700 2.39890

H 6.96360 -5.80710 4.16760

H 8.57960 -6.45530 3.84580

H 8.37420 -4.72430 4.18000

H 7.90600 -7.41110 1.53330

H 6.27710 -6.93630 2.04950

H 6.88020 -6.43080 0.47450

1. N0110_Anti-120GP

N 9.58470 -3.64050 2.14140

C 9.11980 -4.91010 1.57780

C 9.10010 -4.89110 0.04400

N 9.42610 -6.15300 -0.59880

C 10.63110 -6.05300 -1.45480

C 10.26930 -5.42490 -2.80490

O 11.16730 -4.50200 -3.19420

C 10.89670 -3.84540 -4.45040

O 9.28980 -5.73320 -3.45620

O 8.84770 -3.88140 -0.58570

C 7.71900 -5.30890 2.18710

C 6.70180 -4.16620 1.99990

C 7.17080 -6.59140 1.53040

C 7.92680 -5.56920 3.69210

C 11.32780 -7.45200 -1.60970

C 12.52230 -7.33070 -2.57820

C 11.84820 -7.87630 -0.22230

C 10.34360 -8.51560 -2.13330

H 9.15630 -2.86450 1.64110

H 10.58890 -3.54600 2.01640

H 9.82360 -5.69570 1.87250

H 8.64570 -6.39210 -1.21520

H 11.34910 -5.38780 -0.96490

H 11.71210 -3.13550 -4.58640

H 10.87720 -4.57390 -5.26450

H 9.93550 -3.32900 -4.40230

H 7.01260 -3.26370 2.53490

H 6.56080 -3.91040 0.94440

H 5.72860 -4.46840 2.40300

H 6.27290 -6.92210 2.06410

H 6.88080 -6.43150 0.48570

H 7.90120 -7.40620 1.55700

H 6.96630 -5.78450 4.17440

H 8.58350 -6.43230 3.85620

H 8.37540 -4.69970 4.17840

H 13.07700 -8.27490 -2.60170

H 12.20190 -7.11340 -3.60270

H 13.21600 -6.54100 -2.26840

H 12.31910 -8.86390 -0.28130

H 12.60040 -7.17190 0.15400

H 11.03110 -7.92840 0.50210

H 9.51580 -8.66760 -1.43360

H 9.92540 -8.24150 -3.10610

H 10.86210 -9.47440 -2.24450

1. N011'0_Anti-120GP

N 9.46170 -3.69160 2.18780

C 9.01580 -4.94230 1.56730

C 9.10090 -4.89370 0.03500

N 9.42670 -6.15550 -0.59410

C 10.65940 -6.29510 -1.40720

C 10.80470 -7.81570 -1.51680

O 11.97610 -8.25180 -1.01270

C 12.18980 -9.67860 -1.05520

O 9.95970 -8.55270 -1.98580

O 8.84630 -3.87630 -0.58140

C 7.56760 -5.32370 2.07190

C 6.58960 -4.15210 1.85530

C 7.67570 -5.62790 3.57980

C 7.02950 -6.57500 1.35000

C 10.76560 -5.60030 -2.82500

C 11.86060 -6.32110 -3.64670

C 11.20230 -4.12950 -2.65890

C 9.42920 -5.66770 -3.58600

H 9.07530 -2.89810 1.68090

H 10.47320 -3.61010 2.13040

H 9.68300 -5.74560 1.89700

H 8.63230 -6.46170 -1.15520

H 11.41120 -10.19420 -0.48820

H 12.17860 -10.03380 -2.08830

H 13.16810 -9.83740 -0.60250

H 5.58590 -4.44510 2.18350

H 6.88260 -3.27420 2.43910

H 6.52430 -3.86040 0.80190

H 8.11850 -4.78440 4.11500

H 6.68160 -5.82560 3.99710

H 8.29580 -6.51460 3.75950

H 7.73950 -7.40690 1.38910

H 6.09790 -6.90010 1.82620

H 6.79480 -6.37830 0.29740

H 11.57500 -7.34560 -3.90700

H 12.03150 -5.78110 -4.58420

H 12.81400 -6.35590 -3.10680

H 12.16440 -4.06140 -2.13510

H 11.33320 -3.66980 -3.64550

H 10.46430 -3.54350 -2.11050

H 8.65810 -5.07300 -3.08650

H 9.55710 -5.25690 -4.59390

H 9.07430 -6.69870 -3.68710

H 11.49380 -5.93500 -0.79740

1. N1000_Anti-120GP

N 8.61520 -3.74830 2.15510

C 9.11780 -4.95950 1.54650

C 9.10070 -4.89320 0.03890

N 9.42560 -6.15130 -0.59590

C 10.67650 -6.10280 -1.36220

C 10.67780 -7.21360 -2.39470

O 11.92740 -7.51190 -2.79050

C 12.04340 -8.50420 -3.83330

O 9.67600 -7.73800 -2.83620

O 8.84750 -3.88100 -0.58350

C 9.06710 -3.39270 3.51560

C 8.68590 -4.52620 4.48470

C 8.30680 -2.11280 3.90150

C 10.58910 -3.12350 3.59140

H 8.73360 -2.97020 1.51070

H 8.53020 -5.84160 1.83470

H 10.16420 -5.21930 1.80460

H 8.67830 -6.37540 -1.25720

H 11.54000 -6.22050 -0.70080

H 10.80470 -5.15790 -1.91660

H 11.52270 -8.16990 -4.73340

H 13.11230 -8.60240 -4.01850

H 11.61830 -9.45310 -3.49950

H 9.23010 -5.45130 4.26470

H 8.92410 -4.24370 5.51550

H 7.61340 -4.73650 4.42160

H 7.22720 -2.28700 3.87090

H 8.58280 -1.78350 4.90880

H 8.53860 -1.29560 3.20760

H 10.87130 -2.30670 2.91700

H 10.88790 -2.84030 4.60690

H 11.17510 -4.00780 3.31680

1. N1010_Anti-120GP

N 8.74830 -3.74350 2.24660

C 9.04320 -4.98160 1.55170

C 9.10050 -4.89240 0.03660

N 9.42660 -6.15530 -0.59520

C 10.66040 -6.08490 -1.40970

C 10.34120 -5.49730 -2.78830

O 11.26180 -4.60300 -3.18900

C 11.02950 -3.98740 -4.47370

O 9.36940 -5.81090 -3.44900

O 8.84670 -3.87780 -0.58190

C 11.35820 -7.48910 -1.50000

C 10.38760 -8.56490 -2.02390

C 12.58470 -7.40010 -2.43090

C 11.82970 -7.87290 -0.08370

C 9.86580 -3.00800 2.89060

C 11.04070 -2.71520 1.92870

C 9.27040 -1.67840 3.38270

C 10.36580 -3.81540 4.10260

H 8.26860 -3.11950 1.60130

H 8.27970 -5.74950 1.76220

H 9.98010 -5.43470 1.89340

H 8.66070 -6.38920 -1.23130

H 11.36200 -5.40530 -0.91600

H 10.07480 -3.45710 -4.46690

H 11.85760 -3.29380 -4.61510

H 11.01860 -4.74410 -5.26180

H 10.90500 -9.52850 -2.08750

H 9.53540 -8.69150 -1.34880

H 10.00430 -8.31990 -3.01880

H 13.26970 -6.60230 -2.12280

H 12.29970 -7.21450 -3.47190

H 13.13720 -8.34550 -2.40620

H 12.57120 -7.15940 0.29660

H 10.98810 -7.90240 0.61360

H 12.29920 -8.86280 -0.09740

H 11.82510 -2.14120 2.43540

H 11.50320 -3.63880 1.56180

H 10.70060 -2.14060 1.06110

H 8.90440 -1.07730 2.54140

H 8.43230 -1.86300 4.06170

H 10.02500 -1.08680 3.91110

H 10.80170 -4.77590 3.80610

H 11.14200 -3.25930 4.63940

H 9.53950 -4.01680 4.79110

1. N1100_Anti-120GP

N 9.40300 -3.62480 2.16440

C 9.05840 -4.92020 1.58010

C 9.10020 -4.89130 0.04850

N 9.42500 -6.14860 -0.60070

C 10.64950 -6.08370 -1.40910

C 10.58700 -7.14160 -2.49410

O 11.81330 -7.44620 -2.95200

C 11.86960 -8.39260 -4.04100

O 9.55770 -7.62470 -2.92050

O 8.84870 -3.88560 -0.58830

C 7.64050 -5.41130 2.10350

C 7.75950 -5.62750 3.62440

C 7.22190 -6.74450 1.45140

C 6.56350 -4.34620 1.82320

C 10.81910 -3.33210 2.50160

C 10.85490 -1.84870 2.90910

C 11.24870 -4.19220 3.70420

C 11.79900 -3.55640 1.32670

H 9.05270 -2.89570 1.54690

H 9.77820 -5.67890 1.90110

H 8.66640 -6.36380 -1.25310

H 11.53590 -6.25140 -0.79120

H 10.77570 -5.11510 -1.92100

H 12.92930 -8.51220 -4.26310

H 11.42680 -9.34380 -3.73750

H 11.33310 -8.00310 -4.90930

H 6.78690 -5.91630 4.03900

H 8.47150 -6.42860 3.85740

H 8.09180 -4.71350 4.12200

H 6.32380 -7.12630 1.95000

H 6.97430 -6.63290 0.39070

H 8.00580 -7.50370 1.53170

H 6.45910 -4.13880 0.75280

H 5.59230 -4.69730 2.19000

H 6.79420 -3.40700 2.33420

H 11.86250 -1.55570 3.22130

H 10.56430 -1.20410 2.07070

H 10.16510 -1.66340 3.73810

H 12.27130 -3.94050 4.00560

H 10.58330 -4.02010 4.55520

H 11.23200 -5.26230 3.47180

H 11.51100 -2.95990 0.45470

H 12.81850 -3.27280 1.61130

H 11.83090 -4.61010 1.02730

1. N1110_Anti-120GP

N 9.41840 -3.59790 2.14390

C 9.08580 -4.90660 1.58130

C 9.10010 -4.89100 0.04600

N 9.42570 -6.15170 -0.59970

C 10.63130 -6.07010 -1.45630

C 10.27680 -5.43130 -2.80370

O 11.18420 -4.51410 -3.18690

C 10.91860 -3.84570 -4.43790

O 9.29470 -5.72440 -3.45810

O 8.84800 -3.88290 -0.58680

C 7.68270 -5.41040 2.13360

C 7.82440 -5.60040 3.65610

C 7.27260 -6.75990 1.51050

C 6.58640 -4.36540 1.85270

C 11.29870 -7.48260 -1.62120

C 12.49160 -7.37840 -2.59370

C 11.81630 -7.92680 -0.23890

C 10.29280 -8.52440 -2.14790

C 10.82370 -3.29440 2.51510

C 11.21920 -4.11870 3.75390

C 11.83830 -3.55230 1.37750

C 10.84740 -1.79990 2.88020

H 9.08320 -2.88350 1.50150

H 9.82240 -5.65080 1.89740

H 8.64370 -6.38830 -1.21470

H 11.36170 -5.42110 -0.96710

H 11.73910 -3.14080 -4.56830

H 10.89400 -4.56750 -5.25790

H 9.96110 -3.32300 -4.38620

H 8.55510 -6.38300 3.89390

H 8.14390 -4.67200 4.13480

H 6.86280 -5.90130 4.08730

H 6.38600 -7.14450 2.02690

H 7.00940 -6.66940 0.45140

H 8.06680 -7.50810 1.59620

H 6.45830 -4.18140 0.78050

H 5.62710 -4.72030 2.24620

H 6.81660 -3.41330 2.33920

H 13.02840 -8.33260 -2.62500

H 12.17120 -7.14930 -3.61550

H 13.20110 -6.60360 -2.28200

H 12.26360 -8.92480 -0.30640

H 12.58680 -7.24290 0.13760

H 11.00190 -7.96430 0.48930

H 9.46690 -8.66880 -1.44430

H 9.87340 -8.23450 -3.11560

H 10.79400 -9.49100 -2.27080

H 12.23780 -3.86680 4.06860

H 10.53870 -3.91330 4.58530

H 11.19640 -5.19540 3.55530

H 12.84670 -3.24700 1.67900

H 11.88860 -4.61650 1.12210

H 11.56990 -2.99250 0.47530

H 10.13050 -1.59020 3.68010

H 11.84450 -1.49860 3.21760

H 10.58600 -1.17970 2.01420

1. N111'0_Anti-120GP

N 9.31030 -3.67480 2.19890

C 8.96100 -4.94730 1.56670

C 9.10080 -4.89340 0.03740

N 9.42640 -6.15440 -0.59520

C 10.68080 -6.31970 -1.37070

C 10.81760 -7.84350 -1.44870

O 11.95900 -8.27960 -0.87770

C 12.16090 -9.70850 -0.88280

O 9.99240 -8.58350 -1.94680

O 8.84670 -3.87780 -0.58260

C 7.49410 -5.39120 1.99080

C 7.51900 -5.65510 3.50920

C 7.05330 -6.68600 1.27900

C 6.47820 -4.27350 1.68710

C 10.82320 -5.65380 -2.79790

C 11.23090 -4.17310 -2.65020

C 11.95500 -6.37750 -3.56530

C 9.51430 -5.75590 -3.60210

C 10.70760 -3.44220 2.64440

C 11.76240 -3.69590 1.54330

C 10.77220 -1.96570 3.07240

C 11.01230 -4.33040 3.86480

H 9.02880 -2.92170 1.57510

H 9.63120 -5.73460 1.92210

H 8.64610 -6.42700 -1.19210

H 11.35000 -10.20680 -0.34670

H 12.19810 -10.08360 -1.90830

H 13.11370 -9.86720 -0.37870

H 6.51310 -5.91010 3.86200

H 8.18100 -6.49380 3.75670

H 7.86280 -4.77100 4.05100

H 6.11670 -7.04300 1.72170

H 6.85810 -6.53120 0.21220

H 7.79890 -7.48140 1.37270

H 6.44400 -4.02720 0.62060

H 5.47470 -4.59590 1.98720

H 6.71660 -3.36200 2.24260

H 11.38500 -3.73240 -3.64210

H 10.46550 -3.58940 -2.13770

H 12.17370 -4.07670 -2.09730

H 12.15400 -5.85410 -4.50670

H 12.88840 -6.39020 -2.99050

H 11.69050 -7.41030 -3.81460

H 9.17420 -6.79270 -3.69030

H 8.71920 -5.15830 -3.14460

H 9.67190 -5.36650 -4.61410

H 12.76860 -3.45590 1.90510

H 11.77480 -4.74770 1.23590

H 11.56460 -3.08070 0.65910

H 11.76430 -1.71790 3.46350

H 10.57260 -1.30100 2.22300

H 10.02980 -1.75990 3.84960

H 10.96530 -5.39660 3.61940

H 12.02100 -4.12690 4.24060

H 10.29670 -4.13570 4.66890

H 11.50240 -5.95380 -0.74930

1. N0000_Syn60GP

N 10.46030 -5.16780 2.06520

C 9.10730 -4.85740 1.59010

C 9.09930 -4.88820 0.06040

N 9.42170 -6.13550 -0.60680

C 8.55490 -7.27270 -0.34500

C 8.91090 -8.16580 0.84200

O 7.95560 -9.08880 1.04620

C 8.19900 -10.04940 2.09730

O 9.93570 -8.09870 1.49470

O 8.85290 -3.90170 -0.59410

H 10.60170 -6.17070 2.14960

H 10.63870 -4.73150 2.96300

H 8.83780 -3.84220 1.88760

H 8.32840 -5.53770 1.97820

H 10.40380 -6.36510 -0.46730

H 8.55210 -7.92910 -1.22610

H 7.51880 -6.93660 -0.23580

H 7.32790 -10.70330 2.09610

H 9.10950 -10.61520 1.88860

H 8.29960 -9.54060 3.05850

1. N0010_Syn60GP

N 10.51630 -5.09750 2.02890

C 9.13590 -4.85360 1.58930

C 9.09980 -4.89040 0.05850

N 9.42130 -6.13390 -0.60540

C 8.60870 -7.30970 -0.28590

C 9.10500 -8.04750 0.96220

O 8.09540 -8.56630 1.68660

C 8.46700 -9.32410 2.85830

O 10.27630 -8.15160 1.28300

O 8.85280 -3.90120 -0.59370

C 8.51550 -8.26460 -1.54130

C 7.70870 -9.53240 -1.19690

C 7.78390 -7.49230 -2.65630

C 9.92110 -8.66650 -2.02860

H 10.73760 -6.08830 2.07320

H 10.68770 -4.68300 2.93840

H 8.83490 -3.84890 1.89220

H 8.39720 -5.56360 1.99990

H 10.41740 -6.33010 -0.52810

H 7.58790 -6.97290 -0.07760

H 9.09100 -10.17560 2.57750

H 9.01470 -8.69240 3.56120

H 7.52700 -9.66070 3.29370

H 6.72900 -9.28720 -0.77270

H 7.54280 -10.11730 -2.10780

H 8.23110 -10.17910 -0.48420

H 6.75120 -7.26310 -2.36690

H 8.29250 -6.55180 -2.87980

H 7.74760 -8.09770 -3.56880

H 10.49060 -7.79500 -2.36560

H 10.49690 -9.17640 -1.24930

H 9.83360 -9.34910 -2.88100

1. N0100t_Syn60GP

N 10.47150 -5.36600 1.97810

C 9.12640 -4.89430 1.61180

C 9.09980 -4.89010 0.06130

N 9.42080 -6.13200 -0.60660

C 8.54580 -7.26380 -0.30870

C 9.14490 -8.52940 -0.89660

O 8.19130 -9.44670 -1.14860

C 8.65520 -10.72320 -1.63690

O 10.33070 -8.71310 -1.07140

O 8.85330 -3.90340 -0.59510

C 8.61140 -3.59590 2.31990

C 8.62940 -3.84530 3.84470

C 7.15280 -3.31940 1.89630

C 9.49030 -2.36790 2.00690

H 11.15840 -4.61980 1.90900

H 10.50020 -5.72490 2.92630

H 8.43790 -5.69560 1.91470

H 10.36920 -6.40030 -0.33740

H 7.55330 -7.09960 -0.73730

H 8.40870 -7.47600 0.76760

H 9.18400 -10.59520 -2.58390

H 9.32420 -11.18770 -0.90880

H 7.75630 -11.32300 -1.77410

H 9.64600 -3.98290 4.22900

H 8.19820 -2.98720 4.37150

H 8.03840 -4.72950 4.11650

H 6.51250 -4.18970 2.09140

H 6.74620 -2.48090 2.47310

H 7.08040 -3.06870 0.83640

H 9.47690 -2.12660 0.94230

H 9.12310 -1.49700 2.56170

H 10.53130 -2.52290 2.31680

1. N0110_Syn60GP

N 10.67410 -4.72140 1.79570

C 9.22380 -4.85920 1.60100

C 9.10020 -4.89190 0.05870

N 9.42080 -6.13210 -0.60500

C 8.57850 -7.29110 -0.27600

C 9.33730 -8.25820 0.63340

O 8.53950 -8.81590 1.56960

C 9.16340 -9.78470 2.43860

O 10.52440 -8.50080 0.53800

O 8.85280 -3.90150 -0.59420

C 8.29050 -3.85910 2.36150

C 8.64350 -2.38260 2.09260

C 6.82940 -4.12770 1.94600

C 8.43280 -4.13780 3.87360

C 8.04770 -7.99190 -1.58640

C 7.18530 -9.21670 -1.22330

C 7.17620 -6.96330 -2.33310

C 9.21700 -8.42380 -2.48970

H 10.96940 -5.01030 2.72190

H 10.98420 -3.76770 1.63650

H 8.96490 -5.85970 1.96700

H 10.40690 -6.35340 -0.46900

H 7.69170 -6.95350 0.27280

H 8.38010 -10.10290 3.12590

H 9.53460 -10.63180 1.85680

H 9.99570 -9.32970 2.98040

H 7.96750 -1.73230 2.65930

H 9.66180 -2.14370 2.42180

H 8.55570 -2.13520 1.03310

H 6.14420 -3.51870 2.54580

H 6.65900 -3.88290 0.89390

H 6.55630 -5.17870 2.10590

H 7.76630 -3.48340 4.44580

H 8.17080 -5.17460 4.11900

H 9.45280 -3.95090 4.22840

H 6.72500 -9.62470 -2.12960

H 7.77370 -10.01870 -0.76520

H 6.37950 -8.95390 -0.52840

H 6.81600 -7.39030 -3.27560

H 6.29810 -6.67990 -1.73940

H 7.74550 -6.05780 -2.55740

H 9.82510 -7.56170 -2.77770

H 9.86830 -9.15360 -1.99810

H 8.82680 -8.88290 -3.40490

1. N011'0_Syn60GP

N 10.63750 -5.21960 1.86760

C 9.22950 -4.86480 1.60730

C 9.09910 -4.88770 0.06450

N 9.42130 -6.13410 -0.60870

C 8.70230 -7.37050 -0.22520

C 7.27510 -7.21440 -0.77410

O 6.60840 -6.30620 -0.02230

C 5.29090 -5.94350 -0.48490

O 6.77910 -7.76690 -1.73110

O 8.85340 -3.90380 -0.59620

C 8.68450 -3.59100 2.33710

C 8.79620 -3.83250 3.85920

C 7.19440 -3.39060 1.98990

C 9.47690 -2.31690 1.97840

C 9.45630 -8.64710 -0.71560

C 8.62000 -9.89050 -0.34260

C 10.80370 -8.74160 0.03740

C 9.72510 -8.63090 -2.23430

H 10.76400 -5.57900 2.80800

H 11.24470 -4.41080 1.76420

H 8.63150 -5.70790 1.97320

H 10.41370 -6.28500 -0.43500

H 4.92870 -5.20070 0.22470

H 5.34960 -5.52090 -1.49010

H 4.63980 -6.82040 -0.49610

H 8.35960 -2.99070 4.40770

H 8.25890 -4.73950 4.16390

H 9.83740 -3.92610 4.18750

H 6.77920 -2.56780 2.58280

H 7.05810 -3.15170 0.93330

H 6.61270 -4.29160 2.21860

H 9.40920 -2.08990 0.91270

H 9.07840 -1.46350 2.53890

H 10.53620 -2.40330 2.25090

H 9.17670 -10.79990 -0.59350

H 8.40780 -9.92160 0.73380

H 7.67230 -9.91680 -0.88480

H 11.29900 -9.68490 -0.21560

H 11.50040 -7.93880 -0.22820

H 10.66350 -8.71640 1.12430

H 10.29750 -9.52310 -2.51400

H 8.79230 -8.62640 -2.80100

H 10.30330 -7.75030 -2.52860

H 8.60200 -7.47220 0.86840

1. N1010_Syn60GP

N 10.53400 -5.05010 2.00810

C 9.14260 -4.84720 1.58920

C 9.09980 -4.89030 0.05950

N 9.42120 -6.13360 -0.60580

C 8.60710 -7.30820 -0.28740

C 9.11650 -8.05400 0.95080

O 8.11650 -8.59410 1.67280

C 8.50150 -9.35850 2.83560

O 10.29000 -8.14660 1.26610

O 8.85290 -3.90170 -0.59420

C 8.49670 -8.25350 -1.54880

C 7.76070 -7.46780 -2.65170

C 9.89580 -8.65960 -2.05090

C 7.68460 -9.51910 -1.20850

C 10.94820 -4.49340 3.31980

C 12.37840 -5.00430 3.56320

C 10.96890 -2.95630 3.24190

C 10.03200 -4.95340 4.47640

H 10.76100 -6.04140 1.97660

H 8.80610 -3.85300 1.88340

H 8.43280 -5.57870 2.01570

H 10.41660 -6.33110 -0.52480

H 7.58970 -6.97010 -0.06440

H 9.13720 -10.19770 2.54440

H 9.04100 -8.72590 3.54390

H 7.56720 -9.71300 3.26910

H 7.71330 -8.06570 -3.56870

H 6.73170 -7.23510 -2.35200

H 8.27290 -6.52840 -2.87150

H 9.79790 -9.33420 -2.90850

H 10.46800 -7.78830 -2.38390

H 10.47470 -9.18000 -1.28070

H 8.20890 -10.17610 -0.50680

H 6.71080 -9.27120 -0.77220

H 7.50570 -10.09400 -2.12340

H 12.77280 -4.61450 4.50710

H 12.40270 -6.09950 3.61620

H 13.04000 -4.68740 2.75140

H 11.36670 -2.53510 4.17140

H 11.59940 -2.62790 2.41040

H 9.96930 -2.53420 3.09630

H 10.02010 -6.04740 4.55200

H 10.38140 -4.55530 5.43550

H 9.00100 -4.61050 4.33540

1. N1100_Syn60GP

N 10.20160 -3.76180 1.97110

C 9.18790 -4.76750 1.61770

C 9.09880 -4.88680 0.07260

N 9.42060 -6.13100 -0.61260

C 8.59500 -7.30850 -0.39660

C 9.02740 -8.22310 0.74710

O 8.12400 -9.19890 0.95260

C 8.45680 -10.18390 1.95520

O 10.07010 -8.12270 1.36390

O 8.85440 -3.90770 -0.60050

C 7.80450 -4.41260 2.29380

C 7.36570 -2.96800 1.98470

C 7.97840 -4.56660 3.81760

C 6.70300 -5.38230 1.82380

C 11.61390 -4.13650 2.22770

C 12.27110 -4.94610 1.08580

C 12.36770 -2.80530 2.39950

C 11.70320 -4.94060 3.53750

H 10.15860 -2.99630 1.30410

H 9.50500 -5.73260 2.01870

H 10.39940 -6.35610 -0.44800

H 8.61760 -7.92730 -1.30460

H 7.54860 -7.02880 -0.26110

H 9.38240 -10.69810 1.68730

H 8.57650 -9.70660 2.93030

H 7.61770 -10.87840 1.96580

H 6.41820 -2.75600 2.49380

H 8.10540 -2.24860 2.34810

H 7.22220 -2.80450 0.91360

H 7.04760 -4.30420 4.33340

H 8.23300 -5.59800 4.09120

H 8.77110 -3.90950 4.18380

H 6.96660 -6.42810 2.02200

H 5.77090 -5.17800 2.36220

H 6.48500 -5.26970 0.75550

H 13.34330 -5.07710 1.27070

H 11.83650 -5.94840 1.01410

H 12.15990 -4.43190 0.12380

H 13.41690 -2.98590 2.65390

H 12.34560 -2.21760 1.47340

H 11.91420 -2.20700 3.19580

H 11.28870 -4.36500 4.37050

H 11.16230 -5.88960 3.47000

H 12.74820 -5.17750 3.76510

1. N1110_Syn60GP

N 8.31410 -5.83430 2.25100

C 9.07190 -4.75400 1.61750

C 9.10020 -4.89190 0.06860

N 9.41930 -6.12630 -0.60940

C 8.55750 -7.31180 -0.48110

C 9.27620 -8.38680 0.32950

O 8.42580 -9.22110 0.95590

C 9.02490 -10.30210 1.69810

O 10.48730 -8.50050 0.39500

O 8.85430 -3.90730 -0.59970

C 10.52030 -4.54550 2.22150

C 11.30480 -3.53900 1.35280

C 11.33610 -5.85030 2.33110

C 10.37470 -3.94100 3.63540

C 8.11750 -7.82540 -1.91170

C 7.34380 -6.67670 -2.58860

C 9.34700 -8.18470 -2.76550

C 7.18630 -9.04850 -1.79690

C 6.92480 -5.57360 2.72190

C 6.04340 -5.11500 1.54620

C 6.40000 -6.91570 3.26140

C 6.85310 -4.51630 3.84810

H 8.84240 -6.25940 3.00500

H 8.56890 -3.79110 1.76470

H 10.38510 -6.39600 -0.42980

H 7.65150 -7.05410 0.06620

H 8.19000 -10.85700 2.12510

H 9.61410 -10.94010 1.03490

H 9.67240 -9.90980 2.48580

H 12.25460 -3.29330 1.84000

H 11.53920 -3.93840 0.36080

H 10.74500 -2.60940 1.20820

H 12.31720 -5.62560 2.76470

H 10.86410 -6.59200 2.98470

H 11.50620 -6.33510 1.36750

H 9.82370 -4.59910 4.31440

H 11.36510 -3.77500 4.07320

H 9.85590 -2.97680 3.60620

H 7.04920 -6.97030 -3.60250

H 6.42830 -6.43400 -2.03470

H 7.95450 -5.77290 -2.65060

H 9.02800 -8.49480 -3.76680

H 10.01000 -7.32170 -2.87580

H 9.92450 -9.00740 -2.32960

H 7.70160 -9.93390 -1.41320

H 6.33410 -8.84760 -1.13750

H 6.78960 -9.29930 -2.78690

H 5.01840 -4.94940 1.89350

H 6.39700 -4.17800 1.10530

H 6.00790 -5.87040 0.75570

H 5.36860 -6.81450 3.61490

H 6.43310 -7.68520 2.48500

H 7.00660 -7.26150 4.10820

H 7.23580 -3.54450 3.52080

H 5.81660 -4.36840 4.17150

H 7.43280 -4.83330 4.72200

1. N111'0_Syn60GP

N 10.20690 -3.76010 1.95690

C 9.18870 -4.76960 1.61690

C 9.09910 -4.88780 0.06820

N 9.42110 -6.13310 -0.61040

C 8.53160 -7.31090 -0.46180

C 7.59970 -7.24340 -1.68600

O 6.58680 -6.38450 -1.44080

C 5.72380 -6.08370 -2.55630

O 7.74030 -7.81770 -2.74510

O 8.85370 -3.90460 -0.59840

C 7.82220 -4.40310 2.32290

C 6.72290 -5.41880 1.96240

C 7.34260 -2.98800 1.94400

C 8.05540 -4.45900 3.84650

C 9.30400 -8.65910 -0.31720

C 8.28150 -9.81640 -0.35420

C 10.35860 -8.86960 -1.42350

C 9.99850 -8.68500 1.06260

C 11.62810 -4.10820 2.18640

C 11.75410 -4.96900 3.45600

C 12.30490 -4.83280 0.99950

C 12.34560 -2.76510 2.41900

H 10.14000 -2.99160 1.29520

H 9.50460 -5.73590 2.02170

H 10.37510 -6.38820 -0.37300

H 6.30190 -5.62910 -3.36390

H 5.24180 -6.99380 -2.92060

H 4.98520 -5.38200 -2.16970

H 5.80150 -5.17130 2.50120

H 6.48690 -5.42130 0.89440

H 7.00280 -6.43720 2.25960

H 6.42010 -2.75870 2.48970

H 8.08430 -2.23210 2.21900

H 7.13860 -2.89720 0.87450

H 8.82870 -3.74970 4.14980

H 7.12900 -4.20900 4.37600

H 8.36130 -5.46190 4.16970

H 8.78400 -10.76380 -0.13200

H 7.49380 -9.67710 0.39670

H 7.81230 -9.90730 -1.33630

H 10.85410 -9.83600 -1.27600

H 9.90000 -8.85880 -2.41340

H 11.13980 -8.10140 -1.40530

H 10.75410 -7.89920 1.17120

H 9.27540 -8.57330 1.87900

H 10.51260 -9.64170 1.20220

H 12.80760 -5.18270 3.66580

H 11.32730 -4.44760 4.31750

H 11.24110 -5.93100 3.35320

H 13.38290 -4.93530 1.16670

H 11.90980 -5.84680 0.87460

H 12.16000 -4.28180 0.06370

H 11.88280 -2.22330 3.24920

H 13.40300 -2.92560 2.65260

H 12.29400 -2.13160 1.52510

H 7.89700 -7.21420 0.42630

1. N0000_Syn-60GP

N 7.99640 -5.79040 2.03820

C 9.04980 -4.87310 1.58940

C 9.09940 -4.88850 0.06020

N 9.42160 -6.13530 -0.60660

C 10.71680 -6.72970 -0.32360

C 10.80170 -7.69550 0.85710

O 12.07510 -8.06200 1.08110

C 12.29380 -9.03180 2.12930

O 9.85450 -8.12370 1.48990

O 8.85290 -3.90170 -0.59410

H 7.61810 -5.49840 2.93260

H 8.34530 -6.74150 2.12030

H 10.05020 -5.10540 1.99570

H 8.79990 -3.85320 1.88800

H 8.65980 -6.80190 -0.50020

H 11.46800 -5.94450 -0.19130

H 11.04860 -7.29850 -1.20310

H 13.36990 -9.19880 2.14610

H 11.94850 -8.63700 3.08730

H 11.76050 -9.95800 1.90460

1. N0010_Syn-60GP

N 8.24970 -6.02020 2.08900

C 9.01140 -4.87030 1.58800

C 9.09980 -4.89010 0.05550

N 9.42230 -6.13790 -0.60410

C 10.66670 -6.82900 -0.18340

C 11.82920 -5.96220 -0.69340

O 11.92810 -4.85220 0.07790

C 12.88140 -3.85890 -0.35470

O 12.55810 -6.17330 -1.63720

O 8.85180 -3.89750 -0.59190

C 10.69100 -8.31100 -0.67440

C 12.02840 -8.95060 -0.24220

C 9.54860 -9.08050 0.02890

C 10.51670 -8.43500 -2.20170

H 7.24950 -5.84850 2.03150

H 8.46470 -6.20890 3.06300

H 10.03370 -4.93110 1.97580

H 8.60830 -3.89020 1.87180

H 8.64580 -6.76920 -0.41160

H 10.76060 -6.86270 0.91400

H 13.88910 -4.28010 -0.36230

H 12.62540 -3.50590 -1.35590

H 12.80500 -3.04890 0.36990

H 12.17510 -8.87600 0.84290

H 12.03120 -10.01480 -0.50150

H 12.87960 -8.48210 -0.74140

H 9.58800 -8.96310 1.11740

H 8.55420 -8.76420 -0.30640

H 9.63070 -10.14800 -0.20160

H 9.58820 -7.96290 -2.53590

H 11.34470 -7.96520 -2.73520

H 10.47960 -9.49470 -2.48020

1. N0100_Syn-60GP

N 9.36300 -6.13710 2.20080

C 8.96580 -4.85250 1.60960

C 9.10000 -4.89110 0.06340

N 9.41990 -6.12850 -0.60730

C 10.75500 -6.68650 -0.40510

C 10.67730 -8.16320 -0.06320

O 11.88990 -8.73520 -0.07460

C 11.93980 -10.13830 0.26210

O 9.64520 -8.75780 0.18980

O 8.85400 -3.90590 -0.59660

C 9.73920 -3.65820 2.29180

C 9.62820 -3.80760 3.82680

C 9.10250 -2.30640 1.90870

C 11.22830 -3.66560 1.89680

H 9.11470 -6.16620 3.18410

H 8.88890 -6.91940 1.75730

H 7.89150 -4.63760 1.76240

H 8.71490 -6.84490 -0.43590

H 11.29130 -6.20500 0.42090

H 11.36820 -6.57130 -1.30750

H 11.57470 -10.29530 1.27950

H 11.33130 -10.71680 -0.43610

H 12.99000 -10.41540 0.18210

H 10.18740 -4.66980 4.20360

H 10.04320 -2.91840 4.31330

H 8.58270 -3.89650 4.14870

H 8.04290 -2.27570 2.19040

H 9.61240 -1.49750 2.44450

H 9.16750 -2.11110 0.83820

H 11.36050 -3.47460 0.82630

H 11.76670 -2.87870 2.43670

H 11.69690 -4.62310 2.14590

1. N0110_Syn-60GP

N 9.95040 -3.69450 1.96300

C 9.09690 -4.82360 1.59950

C 9.09970 -4.89000 0.05850

N 9.42170 -6.13570 -0.60550

C 10.76710 -6.67390 -0.32390

C 11.78540 -5.90090 -1.17960

O 12.77180 -5.40210 -0.40110

C 13.79420 -4.65200 -1.08460

O 11.74620 -5.75860 -2.38170

O 8.85240 -3.89980 -0.59360

C 7.62440 -4.82380 2.18000

C 6.87330 -3.52070 1.85190

C 6.84010 -6.02340 1.60890

C 7.71730 -4.98860 3.71120

C 10.84050 -8.21790 -0.59540

C 12.30630 -8.67730 -0.44530

C 9.99890 -8.95140 0.47230

C 10.32750 -8.58430 -2.00170

H 9.94330 -3.51950 2.96280

H 9.65000 -2.85300 1.47870

H 9.57180 -5.73290 1.98750

H 8.72770 -6.82640 -0.32640

H 11.06670 -6.52110 0.72270

H 14.47230 -4.30900 -0.30380

H 14.31920 -5.28810 -1.80190

H 13.35110 -3.80480 -1.61300

H 5.86240 -3.55830 2.27390

H 7.37510 -2.64840 2.28390

H 6.78800 -3.35870 0.77420

H 5.85680 -6.09790 2.08630

H 6.66350 -5.92200 0.53140

H 7.36160 -6.97120 1.79180

H 6.71510 -5.01330 4.15320

H 8.22650 -5.92060 3.98500

H 8.25430 -4.15770 4.18270

H 12.36430 -9.76760 -0.52890

H 12.94570 -8.25480 -1.22680

H 12.72270 -8.39110 0.52710

H 10.08600 -10.03430 0.33440

H 10.34130 -8.71460 1.48650

H 8.93040 -8.71420 0.41000

H 9.27490 -8.31750 -2.13430

H 10.89410 -8.06700 -2.77900

H 10.42420 -9.66510 -2.15630

1. N011'0_Syn-60GP

N 8.30410 -5.99220 2.10170

C 8.96840 -4.78370 1.61200

C 9.09980 -4.89070 0.06910

N 9.41930 -6.12630 -0.60990

C 10.70370 -6.80180 -0.40780

C 10.52000 -8.08950 0.39540

O 11.63800 -8.45100 1.05410

C 11.57370 -9.69030 1.79200

O 9.48330 -8.72450 0.44490

O 8.85470 -3.90860 -0.59970

C 10.22070 -4.24580 2.42790

C 9.65580 -3.51960 3.67080

C 11.01970 -3.22890 1.59030

C 11.15650 -5.36350 2.92440

C 11.40520 -7.06070 -1.80730

C 12.78410 -7.72470 -1.62580

C 11.60230 -5.68570 -2.47540

C 10.51310 -7.94130 -2.70020

H 7.91400 -5.85740 3.02870

H 8.90200 -6.81160 2.12150

H 8.22640 -3.98440 1.69920

H 8.65160 -6.78730 -0.49180

H 12.55870 -9.80810 2.24260

H 10.79960 -9.63580 2.56130

H 11.35300 -10.52170 1.11840

H 10.46930 -3.15460 4.30750

H 9.04250 -2.65830 3.38320

H 9.03740 -4.18630 4.28400

H 11.79280 -2.75790 2.20780

H 11.52120 -3.70170 0.73860

H 10.37390 -2.44070 1.19150

H 11.63320 -5.92190 2.11560

H 11.95970 -4.92540 3.52750

H 10.62870 -6.08060 3.56280

H 13.29880 -7.76210 -2.59210

H 13.41780 -7.16000 -0.93260

H 12.71170 -8.74930 -1.25070

H 12.04720 -5.81440 -3.46830

H 10.65110 -5.15990 -2.58430

H 12.28010 -5.05270 -1.88900

H 10.98640 -8.07810 -3.67880

H 10.34990 -8.93470 -2.26800

H 9.53790 -7.47320 -2.86090

H 11.38490 -6.15440 0.15140

1. N1000_Syn-60GP

N 8.61480 -6.06730 2.20200

C 9.09230 -4.83060 1.59040

C 9.10050 -4.89300 0.05300

N 9.42140 -6.13470 -0.60220

C 10.73020 -6.68410 -0.24780

C 10.79570 -8.13940 -0.66980

O 12.07040 -8.55100 -0.80310

C 12.25570 -9.94370 -1.13160

O 9.82800 -8.85500 -0.82490

O 8.85190 -3.89790 -0.59130

C 8.80820 -6.23870 3.66730

C 8.05330 -7.52310 4.04750

C 8.25350 -5.05230 4.48690

C 10.30610 -6.42760 3.96480

H 7.62180 -6.17210 2.00240

H 10.13040 -4.65710 1.90140

H 8.53100 -3.92490 1.85850

H 8.72120 -6.83990 -0.36610

H 10.94140 -6.67810 0.83490

H 11.52460 -6.12360 -0.74880

H 11.82810 -10.57780 -0.35130

H 11.77810 -10.17320 -2.08650

H 13.33410 -10.08350 -1.19520

H 6.97830 -7.42010 3.85410

H 8.42420 -8.37340 3.46720

H 8.17740 -7.74490 5.11220

H 8.35360 -5.24090 5.56140

H 8.78600 -4.12140 4.26570

H 7.19010 -4.89310 4.27240

H 10.45680 -6.62790 5.03050

H 10.70670 -7.27270 3.39630

H 10.89340 -5.53590 3.72050

1. N1010_Syn-60GP

N 7.66790 -5.62470 1.85110

C 8.91470 -4.89740 1.57750

C 9.09970 -4.88980 0.05760

N 9.42210 -6.13730 -0.60510

C 10.64480 -6.86110 -0.19000

C 11.83140 -5.97980 -0.61090

O 11.85240 -4.86880 0.16260

C 12.82040 -3.85960 -0.19420

O 12.63800 -6.18540 -1.49050

O 8.85210 -3.89850 -0.59300

C 10.66980 -8.31320 -0.76350

C 9.48860 -9.10210 -0.15220

C 10.55450 -8.34430 -2.30110

C 11.97790 -9.00190 -0.31700

C 7.38760 -6.01790 3.25820

C 5.95020 -6.56430 3.26790

C 8.35600 -7.13800 3.67600

C 7.49660 -4.83080 4.23980

H 6.88950 -5.06020 1.51510

H 9.74750 -5.41990 2.05350

H 8.92060 -3.85470 1.92250

H 8.61760 -6.74660 -0.46510

H 10.71600 -6.95830 0.90740

H 13.83250 -4.26420 -0.12090

H 12.63880 -3.51140 -1.21330

H 12.67250 -3.05080 0.52060

H 9.55700 -10.15400 -0.44910

H 9.49870 -9.06320 0.94340

H 8.51200 -8.74330 -0.49680

H 10.51550 -9.38470 -2.64440

H 9.64640 -7.83860 -2.64180

H 11.41010 -7.85730 -2.77230

H 12.85570 -8.52200 -0.75470

H 12.08250 -8.99150 0.77550

H 11.97240 -10.04970 -0.63630

H 5.67440 -6.91130 4.26870

H 5.23140 -5.78890 2.97580

H 5.85240 -7.40140 2.56980

H 8.11390 -7.49190 4.68350

H 8.28230 -7.98330 2.98520

H 9.39680 -6.79770 3.69530

H 6.82190 -4.01790 3.94670

H 7.22810 -5.13980 5.25590

H 8.51450 -4.42880 4.27660

1. N1100_Syn-60GP

N 9.44320 -3.50880 2.12250

C 9.01420 -4.81080 1.61050

C 9.09920 -4.88820 0.06980

N 9.42060 -6.13120 -0.61100

C 10.71050 -6.75600 -0.37190

C 10.80710 -7.73640 0.79520

O 12.09310 -8.04420 1.05250

C 12.32260 -9.04330 2.06980

O 9.86310 -8.22430 1.38370

O 8.85400 -3.90610 -0.59930

C 7.52100 -5.10790 2.08700

C 6.60030 -3.91410 1.76940

C 7.56220 -5.32650 3.61150

C 6.94010 -6.37480 1.42550

C 10.83860 -3.27910 2.56540

C 11.90270 -3.61720 1.49670

C 10.92490 -1.77780 2.89630

C 11.11630 -4.08900 3.84460

H 9.18430 -2.80060 1.44020

H 9.63440 -5.60060 2.04070

H 8.66420 -6.80260 -0.50780

H 11.48710 -5.99480 -0.27320

H 10.98580 -7.33390 -1.26560

H 11.92500 -8.70540 3.02930

H 11.84370 -9.98440 1.79070

H 13.40440 -9.15970 2.12050

H 5.57940 -4.14450 2.09470

H 6.92510 -3.01340 2.29680

H 6.56790 -3.69470 0.69730

H 6.54700 -5.47050 3.99860

H 8.14730 -6.21710 3.87040

H 8.00160 -4.46130 4.11400

H 7.59450 -7.24190 1.55420

H 5.97380 -6.61240 1.88390

H 6.74480 -6.22720 0.35560

H 12.90170 -3.31580 1.83150

H 11.94440 -4.69360 1.30340

H 11.69120 -3.10200 0.55350

H 11.91580 -1.52200 3.28520

H 10.74960 -1.16800 2.00180

H 10.17490 -1.50820 3.64600

H 10.40530 -3.82000 4.63110

H 11.03860 -5.16800 3.67290

H 12.12980 -3.88820 4.20880

1. N1110_Syn-60GP

N 8.46580 -6.06920 2.18730

C 8.90420 -4.79230 1.61840

C 9.09960 -4.88970 0.06800

N 9.42010 -6.12910 -0.60970

C 10.63740 -6.86840 -0.26210

C 11.84380 -6.14940 -0.88850

O 12.88020 -6.09000 -0.01120

C 14.09050 -5.48880 -0.51090

O 11.91100 -5.70720 -2.01260

O 8.85420 -3.90670 -0.59880

C 10.07570 -4.09470 2.42780

C 11.22630 -5.04540 2.81760

C 9.46870 -3.52100 3.72900

C 10.66010 -2.91880 1.61870

C 10.56150 -8.35440 -0.78570

C 9.47680 -9.11130 0.01070

C 10.23460 -8.41960 -2.29050

C 11.91260 -9.05310 -0.52340

C 7.01490 -6.30620 2.42400

C 6.24710 -6.24770 1.09100

C 6.90330 -7.72330 3.01260

C 6.38170 -5.30300 3.41640

H 8.97440 -6.28420 3.03750

H 8.09170 -4.05650 1.66850

H 8.62240 -6.74300 -0.46570

H 10.80840 -6.92970 0.81950

H 14.79240 -5.51240 0.32250

H 14.47800 -6.06060 -1.35780

H 13.90160 -4.46170 -0.83130

H 11.94160 -4.49860 3.44240

H 10.89030 -5.90450 3.41000

H 11.77500 -5.42140 1.95340

H 10.24630 -3.01660 4.31250

H 8.68370 -2.78780 3.51480

H 9.03720 -4.30100 4.36410

H 9.88080 -2.22220 1.29670

H 11.37660 -2.37030 2.24030

H 11.18680 -3.25500 0.72120

H 9.46890 -10.16490 -0.28930

H 9.66260 -9.06360 1.08870

H 8.46870 -8.72460 -0.17330

H 10.19560 -9.46730 -2.61020

H 9.27040 -7.95680 -2.51610

H 10.98880 -7.90310 -2.88910

H 12.72020 -8.62580 -1.12580

H 12.20600 -8.98670 0.53020

H 11.83550 -10.11350 -0.78600

H 5.18300 -6.44000 1.26170

H 6.32660 -5.26760 0.60990

H 6.61030 -7.00830 0.39220

H 5.85720 -7.98120 3.20730

H 7.32280 -8.46330 2.32520

H 7.44400 -7.79690 3.96440

H 6.44730 -4.27170 3.05620

H 5.32030 -5.52950 3.56660

H 6.87520 -5.35370 4.39290

1. N111'0_Syn-60GP

N 9.40270 -3.48830 2.11150

C 9.03260 -4.81280 1.61110

C 9.09970 -4.89000 0.06700

N 9.42050 -6.13070 -0.60920

C 10.74860 -6.71900 -0.38540

C 10.70460 -7.80100 0.69420

O 11.78570 -7.76590 1.49970

C 11.85990 -8.79050 2.51470

O 9.80160 -8.60610 0.82410

O 8.85370 -3.90480 -0.59830

C 7.56940 -5.18600 2.12200

C 7.04660 -6.49820 1.50270

C 6.57750 -4.05370 1.79310

C 7.64600 -5.36470 3.65040

C 11.34750 -7.26360 -1.74050

C 12.71190 -7.93440 -1.48570

C 10.38920 -8.27050 -2.40320

C 11.54540 -6.05310 -2.67320

C 10.78560 -3.18530 2.55380

C 11.10950 -3.97800 3.83270

C 11.86620 -3.46430 1.48470

C 10.78900 -1.68110 2.88380

H 9.11640 -2.80230 1.41710

H 9.70940 -5.56550 2.02770

H 8.70100 -6.83100 -0.44500

H 10.99690 -8.72880 3.18110

H 11.88740 -9.77980 2.05210

H 12.78350 -8.59240 3.05730

H 6.10860 -6.78140 1.99270

H 6.81470 -6.38870 0.43620

H 7.74610 -7.33090 1.62800

H 5.57680 -4.33020 2.14380

H 6.86270 -3.12280 2.28990

H 6.51350 -3.86490 0.71640

H 8.04130 -4.46390 4.12570

H 6.64730 -5.56020 4.05720

H 8.28670 -6.21270 3.92210

H 13.17520 -8.20020 -2.44180

H 13.39950 -7.26630 -0.95500

H 12.61990 -8.85570 -0.90040

H 10.82510 -8.63110 -3.34160

H 10.19940 -9.13910 -1.76480

H 9.43000 -7.80140 -2.64140

H 10.60560 -5.51450 -2.81830

H 12.28310 -5.35220 -2.26370

H 11.91090 -6.38780 -3.65040

H 12.10950 -3.71730 4.19610

H 10.38510 -3.75160 4.62040

H 11.09880 -5.05930 3.65900

H 12.84470 -3.09860 1.81640

H 11.97480 -4.53700 1.29890

H 11.62160 -2.96910 0.53890

H 10.02630 -1.45230 3.63430

H 11.76500 -1.37070 3.27070

H 10.57890 -1.08270 1.98900

H 11.42890 -5.93680 -0.04220

1. N0000_Anti120WP

N 8.78480 -3.69720 2.24560

C 9.12870 -4.94120 1.56030

C 9.10040 -4.89220 0.04650

N 9.42570 -6.15170 -0.59960

C 8.31310 -6.61230 -1.45770

C 8.72960 -7.81870 -2.27510

O 7.68230 -8.33190 -2.93230

C 7.92940 -9.45380 -3.82630

O 9.86800 -8.25300 -2.35500

O 8.84770 -3.88160 -0.58740

H 7.83850 -3.40990 1.98590

H 9.39480 -2.94230 1.92380

H 8.45890 -5.75400 1.87870

H 10.13730 -5.27540 1.84580

H 10.23930 -5.96800 -1.20090

H 7.96440 -5.84580 -2.16640

H 7.45490 -6.89150 -0.83410

H 6.95720 -9.69160 -4.25800

H 8.63580 -9.15810 -4.60480

H 8.31980 -10.30280 -3.26190

1. N0010_Anti120WP

N 8.95260 -3.63440 2.20680

C 9.27820 -4.89460 1.54000

C 9.10080 -4.89330 0.03350

N 9.42770 -6.15970 -0.59360

C 8.41600 -6.89410 -1.41570

C 9.17460 -8.14160 -1.88490

O 8.46400 -9.26370 -1.72970

C 9.03850 -10.50050 -2.23970

O 10.30090 -8.11530 -2.36110

O 8.84530 -3.87250 -0.58040

C 7.68720 -6.17850 -2.63260

C 6.51930 -5.31690 -2.09890

C 8.66080 -5.32850 -3.46970

C 7.05710 -7.25310 -3.55130

H 7.97350 -3.39830 2.02760

H 9.49040 -2.87410 1.78410

H 8.67980 -5.71700 1.95730

H 10.32550 -5.17380 1.72630

H 10.23760 -5.97000 -1.19630

H 7.63130 -7.23870 -0.73330

H 9.23230 -10.40590 -3.31050

H 9.96370 -10.73170 -1.70770

H 8.28360 -11.26410 -2.05170

H 5.98480 -4.86280 -2.94110

H 5.80370 -5.93700 -1.54570

H 6.84950 -4.51020 -1.44580

H 9.08210 -4.50450 -2.89030

H 9.48380 -5.93700 -3.86120

H 8.12590 -4.89910 -4.32540

H 6.45620 -6.75410 -4.31960

H 7.81120 -7.85700 -4.06880

H 6.39670 -7.92520 -2.99300

1. N0100_Anti120WP

N 10.68200 -4.80420 1.88580

C 9.23250 -4.92530 1.58570

C 9.09900 -4.88720 0.05800

N 9.42610 -6.15300 -0.60610

C 8.32380 -6.61320 -1.47960

C 7.17450 -7.22070 -0.67900

O 6.04940 -7.25070 -1.40980

C 4.86040 -7.82820 -0.79840

O 7.24730 -7.63610 0.46550

O 8.84870 -3.88530 -0.59240

C 8.34460 -3.91670 2.38910

C 8.85100 -2.46240 2.29970

C 8.34720 -4.35210 3.87190

C 6.88540 -3.98630 1.88250

H 11.18940 -5.61290 1.52150

H 11.08780 -3.97660 1.44410

H 8.93710 -5.93440 1.89120

H 10.22200 -5.95210 -1.22450

H 8.70790 -7.40350 -2.13730

H 7.91850 -5.82260 -2.12400

H 4.08570 -7.75810 -1.56170

H 5.04320 -8.87020 -0.52780

H 4.57860 -7.25250 0.08580

H 9.85230 -2.35690 2.73200

H 8.87670 -2.10430 1.26680

H 8.18090 -1.80590 2.86830

H 7.73790 -3.65890 4.46430

H 7.91970 -5.35510 3.99060

H 9.35750 -4.35670 4.29170

H 6.50370 -5.01440 1.90530

H 6.24080 -3.38370 2.53350

H 6.78090 -3.59980 0.86460

1. N0110_Anti120WP

N 10.79110 -4.87100 1.76260

C 9.32130 -4.93260 1.55840

C 9.10040 -4.89220 0.04010

N 9.42710 -6.15720 -0.59680

C 8.37770 -6.83000 -1.40750

C 8.79760 -8.30730 -1.38000

O 7.82280 -9.09970 -0.91090

C 8.09190 -10.52750 -0.83000

O 9.89340 -8.72370 -1.72790

O 8.84630 -3.87610 -0.58380

C 8.52090 -3.89530 2.40770

C 9.03680 -2.45100 2.24920

C 7.02870 -3.95980 2.00980

C 8.63390 -4.30650 3.89200

C 8.07470 -6.33280 -2.88270

C 7.41020 -7.48200 -3.67800

C 7.06680 -5.16270 -2.84840

C 9.36290 -5.89200 -3.60160

H 11.19900 -4.07350 1.27040

H 11.23230 -5.70870 1.37690

H 9.00530 -5.92880 1.88910

H 10.27140 -6.02470 -1.15950

H 7.44140 -6.76550 -0.84340

H 7.17480 -10.96860 -0.43910

H 8.31760 -10.92280 -1.82280

H 8.92840 -10.71200 -0.15240

H 10.07710 -2.35640 2.58080

H 8.96950 -2.11000 1.21300

H 8.43410 -1.77630 2.86940

H 6.43090 -3.35720 2.70380

H 6.85500 -3.57480 1.00100

H 6.65080 -4.98920 2.05610

H 8.07850 -3.60020 4.52020

H 8.21140 -5.30500 4.05800

H 9.67460 -4.31520 4.22990

H 7.10770 -7.11260 -4.66440

H 8.08890 -8.32700 -3.84020

H 6.51080 -7.85110 -3.17120

H 6.82720 -4.85590 -3.87370

H 6.12990 -5.46550 -2.36370

H 7.46430 -4.29650 -2.31900

H 9.80740 -5.01080 -3.12660

H 10.11070 -6.69260 -3.62140

H 9.13240 -5.62370 -4.63910

1. N011'0_Anti120WP

N 10.80990 -4.97930 1.74950

C 9.33210 -4.92480 1.56200

C 9.09970 -4.88940 0.04540

N 9.42770 -6.15940 -0.59990

C 8.42870 -6.87520 -1.43440

C 7.19460 -7.14160 -0.57680

O 6.53250 -8.24680 -0.94430

C 5.29210 -8.54180 -0.24130

O 6.83090 -6.41690 0.33600

O 8.84650 -3.87670 -0.58600

C 8.66340 -3.80130 2.41820

C 9.30750 -2.41580 2.20240

C 8.80930 -4.19160 3.90610

C 7.15380 -3.71140 2.10400

C 8.04610 -6.27640 -2.86290

C 7.64290 -7.44900 -3.78460

C 9.29010 -5.59750 -3.47110

C 6.88210 -5.26420 -2.80670

H 11.26680 -4.19550 1.27760

H 11.17860 -5.82860 1.31710

H 8.93840 -5.88400 1.91240

H 10.27300 -6.01440 -1.15680

H 5.49090 -8.70120 0.82080

H 4.58380 -7.72030 -0.37040

H 4.91250 -9.45320 -0.70300

H 8.80350 -1.67620 2.83660

H 10.36810 -2.41090 2.47780

H 9.21540 -2.08810 1.16370

H 9.85930 -4.26650 4.20310

H 8.32890 -3.43660 4.53960

H 8.32560 -5.15480 4.10970

H 6.66010 -4.67690 2.24870

H 6.68330 -2.98530 2.77780

H 6.96760 -3.38830 1.07650

H 6.76380 -7.98170 -3.41060

H 7.40660 -7.06540 -4.78440

H 8.46350 -8.17050 -3.88380

H 10.15340 -6.27540 -3.48670

H 9.07660 -5.31060 -4.50710

H 9.56550 -4.69320 -2.92240

H 7.10250 -4.42880 -2.13910

H 6.70350 -4.85890 -3.81000

H 5.94790 -5.73470 -2.47720

H 8.89090 -7.84750 -1.63880

1. N1000_Anti120WP

N 8.80680 -3.74550 2.28950

C 9.18460 -4.95960 1.56590

C 9.09920 -4.88770 0.05160

N 9.42640 -6.15430 -0.60310

C 8.32990 -6.68270 -1.44410

C 7.30090 -7.46520 -0.63160

O 6.22730 -7.75770 -1.38380

C 5.15610 -8.52560 -0.76340

O 7.41600 -7.79570 0.53680

O 8.84820 -3.88340 -0.58900

C 7.38450 -3.61550 2.75190

C 6.42730 -3.57610 1.54770

C 7.29720 -2.27650 3.50620

C 6.96280 -4.75400 3.71020

H 9.40270 -3.68260 3.11700

H 8.63360 -5.86510 1.86100

H 10.23800 -5.19780 1.77670

H 10.20370 -5.93680 -1.23930

H 8.75610 -7.38010 -2.17550

H 7.80210 -5.90740 -2.01620

H 4.74150 -7.97290 0.08240

H 4.40540 -8.65020 -1.54400

H 5.53130 -9.49570 -0.43130

H 6.41450 -4.52170 0.99520

H 5.40510 -3.39800 1.90010

H 6.69960 -2.77550 0.85320

H 7.55570 -1.43940 2.84840

H 6.28060 -2.11680 3.88250

H 7.97810 -2.26440 4.36610

H 7.61990 -4.78980 4.58740

H 5.93810 -4.58750 4.06290

H 6.98080 -5.73630 3.22610

1. N1010_Anti120WP

N 8.91220 -3.74600 2.27700

C 9.28950 -4.95200 1.54310

C 9.10010 -4.89070 0.03450

N 9.42820 -6.16160 -0.59470

C 8.42700 -6.87910 -1.43850

C 9.07480 -8.25090 -1.67100

O 8.24110 -9.26350 -1.40250

C 8.71870 -10.61570 -1.65400

O 10.22300 -8.40870 -2.06260

O 8.84550 -3.87310 -0.58030

C 7.91400 -6.25180 -2.80550

C 9.04880 -5.54670 -3.56880

C 7.33190 -7.37710 -3.69580

C 6.76120 -5.26220 -2.52440

C 7.50760 -3.66220 2.79870

C 7.20430 -4.76200 3.84280

C 7.37580 -2.28320 3.46820

C 6.49080 -3.76270 1.64790

H 9.53960 -3.65880 3.07900

H 8.79970 -5.87610 1.89160

H 10.36490 -5.14380 1.67590

H 10.26570 -5.99060 -1.16240

H 7.54440 -7.05390 -0.81450

H 9.57410 -10.83660 -1.01170

H 7.87660 -11.26480 -1.41170

H 8.99800 -10.72370 -2.70460

H 8.67160 -5.18430 -4.53240

H 9.43140 -4.68490 -3.01510

H 9.88100 -6.22970 -3.77220

H 6.55070 -7.94060 -3.17400

H 8.09850 -8.08220 -4.03640

H 6.88160 -6.93120 -4.58970

H 5.93500 -5.76460 -2.00630

H 7.07950 -4.41090 -1.92260

H 6.37090 -4.87480 -3.47320

H 6.18860 -4.64100 4.23660

H 7.26750 -5.76770 3.41240

H 7.90230 -4.70270 4.68660

H 8.10290 -2.17030 4.28190

H 7.54270 -1.48070 2.74160

H 6.37370 -2.15910 3.89420

H 6.50100 -4.74990 1.17200

H 5.48020 -3.61010 2.04390

H 6.68100 -3.00600 0.88150

1. N1100_Anti120WP

N 10.21770 -3.85550 2.01370

C 9.22760 -4.85850 1.59690

C 9.09950 -4.88890 0.05850

N 9.42530 -6.14970 -0.60590

C 8.29930 -6.72510 -1.37310

C 8.78360 -7.91640 -2.18150

O 7.76490 -8.70250 -2.54760

C 8.07080 -9.86230 -3.37390

O 9.94840 -8.12220 -2.48470

O 8.84910 -3.88680 -0.59320

C 7.84680 -4.63590 2.33200

C 8.07060 -4.91070 3.83250

C 6.79480 -5.62890 1.79990

C 7.32960 -3.19560 2.16140

C 11.63920 -4.24040 2.22040

C 12.38150 -2.93480 2.55830

C 11.75270 -5.20180 3.41900

C 12.28720 -4.88680 0.97600

H 10.16200 -3.03140 1.41770

H 9.58130 -5.84720 1.89700

H 10.14860 -5.90940 -1.29510

H 7.84450 -6.01500 -2.08360

H 7.51010 -7.06260 -0.69560

H 7.11160 -10.35110 -3.54380

H 8.51410 -9.54070 -4.31840

H 8.75370 -10.52720 -2.84140

H 7.14140 -4.73460 4.38720

H 8.37470 -5.94970 4.00740

H 8.84330 -4.25320 4.23910

H 5.87950 -5.56010 2.39940

H 6.51360 -5.41400 0.76250

H 7.15190 -6.66440 1.86110

H 7.13070 -2.95390 1.11330

H 6.39360 -3.07310 2.71980

H 8.05000 -2.47150 2.55430

H 13.44120 -3.13740 2.74760

H 12.31790 -2.22040 1.72790

H 11.95180 -2.46640 3.45040

H 12.80650 -5.43420 3.61020

H 11.32950 -4.74700 4.32040

H 11.23840 -6.15220 3.23860

H 12.21870 -4.21740 0.11030

H 13.34750 -5.09960 1.15590

H 11.79890 -5.83370 0.72230

1. N1110_Anti120WP

N 10.18740 -3.70740 1.94320

C 9.32290 -4.83190 1.57600

C 9.10000 -4.89050 0.04700

N 9.42680 -6.15590 -0.60030

C 8.42660 -6.94120 -1.38380

C 9.20290 -8.21670 -1.74030

O 8.54760 -9.32920 -1.38800

C 9.16720 -10.60600 -1.71310

O 10.29910 -8.22240 -2.28280

O 8.84710 -3.87910 -0.58720

C 7.96010 -4.78710 2.37610

C 8.27890 -5.02790 3.86470

C 7.01940 -5.90600 1.88560

C 7.25870 -3.42350 2.22690

C 7.72630 -6.31980 -2.66230

C 7.11350 -7.47120 -3.49570

C 6.55780 -5.40690 -2.22790

C 8.71620 -5.53370 -3.54070

C 11.65890 -3.90540 2.01960

C 11.99670 -4.83930 3.19750

C 12.27320 -4.47190 0.71910

C 12.25470 -2.51370 2.29690

H 9.97800 -2.90360 1.35370

H 9.81190 -5.76730 1.85530

H 10.19000 -5.93210 -1.24780

H 7.63120 -7.24170 -0.69670

H 8.46570 -11.36090 -1.35740

H 9.30770 -10.68780 -2.79340

H 10.12730 -10.69650 -1.19990

H 8.71870 -6.02070 4.02130

H 8.97950 -4.27710 4.23990

H 7.35850 -4.97070 4.45820

H 6.13020 -5.95470 2.52490

H 6.66450 -5.72690 0.86410

H 7.50760 -6.88790 1.91920

H 7.00150 -3.20620 1.18520

H 6.32910 -3.42240 2.80910

H 7.89070 -2.61270 2.60360

H 6.52460 -7.04850 -4.31730

H 7.87820 -8.11740 -3.94170

H 6.44440 -8.09390 -2.89080

H 6.03440 -5.03610 -3.11730

H 5.82930 -5.96160 -1.62340

H 6.89470 -4.54090 -1.65750

H 9.11760 -4.66410 -3.01300

H 9.55200 -6.16200 -3.86660

H 8.20030 -5.17150 -4.43770

H 13.08380 -4.93080 3.30140

H 11.59060 -4.44130 4.13310

H 11.59790 -5.84930 3.05150

H 13.36430 -4.53700 0.80230

H 11.90170 -5.48130 0.51040

H 12.03800 -3.82850 -0.13710

H 11.84180 -2.09550 3.22120

H 13.34340 -2.57710 2.39850

H 12.03230 -1.82130 1.47520

1. N111'0_Anti120WP

N 10.20800 -3.88740 2.02110

C 9.20250 -4.87320 1.59330

C 9.10030 -4.89200 0.05200

N 9.42550 -6.15080 -0.60210

C 8.32470 -6.75530 -1.38960

C 8.27900 -6.15190 -2.80110

O 7.02920 -5.92350 -3.22060

C 6.85420 -5.40070 -4.56820

O 9.27260 -5.91260 -3.47450

O 8.84800 -3.88260 -0.59040

C 7.82490 -4.63210 2.32870

C 8.04770 -4.92360 3.82760

C 6.75000 -5.60120 1.79880

C 7.32980 -3.18290 2.16750

C 8.43990 -8.32960 -1.41520

C 8.24780 -8.84500 0.02640

C 7.32380 -8.92450 -2.30070

C 9.81440 -8.78960 -1.93870

C 11.62510 -4.29580 2.21570

C 12.25340 -4.95780 0.96890

C 12.39050 -3.00100 2.54280

C 11.73240 -5.25570 3.41590

H 10.16460 -3.06080 1.42740

H 9.54190 -5.86740 1.88970

H 10.19680 -5.95540 -1.24840

H 7.35400 -4.43350 -4.66030

H 7.25980 -6.10640 -5.29670

H 5.77650 -5.29290 -4.69460

H 7.12110 -4.74500 4.38630

H 8.34150 -5.96830 3.99110

H 8.82880 -4.27920 4.23970

H 5.83740 -5.50970 2.39930

H 6.47160 -5.38190 0.76120

H 7.08110 -6.64400 1.86030

H 7.13470 -2.93400 1.12000

H 6.39510 -3.05120 2.72620

H 8.06070 -2.47110 2.56420

H 8.36930 -9.93430 0.04990

H 8.98330 -8.40280 0.70490

H 7.24340 -8.61490 0.40220

H 7.33230 -10.01710 -2.21000

H 6.33240 -8.57010 -1.99220

H 7.45950 -8.68130 -3.36100

H 9.98970 -8.46280 -2.96930

H 10.62940 -8.41270 -1.31080

H 9.86110 -9.88520 -1.92190

H 13.31320 -5.18000 1.14120

H 11.75350 -5.90150 0.72620

H 12.18490 -4.29320 0.09910

H 13.44770 -3.21910 2.72880

H 12.33400 -2.29110 1.70820

H 11.97190 -2.52060 3.43370

H 11.20110 -6.19760 3.24080

H 12.78360 -5.50480 3.60010

H 11.32270 -4.79240 4.31910

H 7.37300 -6.51850 -0.90730

1. N0000_Anti-120WP

N 8.79500 -3.69240 2.24420

C 9.09860 -4.94790 1.56030

C 9.10040 -4.89210 0.04640

N 9.42580 -6.15200 -0.59960

C 10.60690 -6.01070 -1.47770

C 10.82200 -7.26650 -2.29830

O 11.96010 -7.18580 -2.99880

C 12.28030 -8.28530 -3.89740

O 10.05580 -8.21600 -2.34460

O 8.84770 -3.88150 -0.58730

H 7.89110 -3.33360 1.92870

H 9.47700 -2.97950 1.97600

H 8.38230 -5.73100 1.84970

H 10.08070 -5.33050 1.87630

H 8.61510 -6.40260 -1.18020

H 11.50240 -5.83630 -0.86800

H 10.52730 -5.17080 -2.18400

H 13.21470 -7.99540 -4.37750

H 12.40870 -9.20750 -3.32730

H 11.48610 -8.40310 -4.63720

1. N0010_Anti-120WP

N 8.86360 -3.66490 2.22640

C 9.10620 -4.94220 1.55720

C 9.10080 -4.89390 0.04330

N 9.42580 -6.15220 -0.59770

C 10.63490 -6.01650 -1.45240

C 10.32690 -5.19070 -2.71210

O 11.21960 -4.20790 -2.89310

C 11.03260 -3.32350 -4.03180

O 9.38320 -5.39570 -3.46230

O 8.84720 -3.87950 -0.58600

C 11.28210 -7.41080 -1.77720

C 11.82470 -8.00890 -0.46260

C 10.26590 -8.38530 -2.40330

C 12.46590 -7.20670 -2.74710

H 7.98730 -3.26000 1.88890

H 9.58910 -2.99760 1.95290

H 8.35690 -5.69090 1.85430

H 10.07100 -5.36290 1.87430

H 8.62420 -6.41330 -1.18410

H 11.37770 -5.45210 -0.87780

H 11.08130 -3.89240 -4.96280

H 10.06960 -2.81390 -3.95020

H 11.85290 -2.60770 -3.97370

H 12.27760 -8.98720 -0.66040

H 12.59560 -7.36630 -0.02060

H 11.02800 -8.14410 0.27380

H 9.43360 -8.59690 -1.72300

H 9.85610 -7.99650 -3.34020

H 10.76140 -9.33840 -2.62140

H 12.98820 -8.15900 -2.89320

H 12.13900 -6.85610 -3.73280

H 13.18790 -6.48470 -2.34830

1. N0100_Anti-120WP

N 9.70370 -3.62200 2.10190

C 9.17790 -4.89710 1.58510

C 9.10040 -4.89230 0.04950

N 9.42550 -6.15070 -0.60090

C 10.52800 -5.99620 -1.56940

C 10.83280 -7.33070 -2.22290

O 12.09080 -7.37600 -2.67770

C 12.50820 -8.57720 -3.38630

O 10.02830 -8.24150 -2.34830

O 8.84800 -3.88250 -0.58910

C 7.80240 -5.29070 2.26110

C 6.79410 -4.12470 2.20690

C 8.07670 -5.66080 3.73330

C 7.18230 -6.51860 1.56040

H 10.65440 -3.48520 1.75090

H 9.16190 -2.84320 1.72320

H 9.89300 -5.68640 1.84540

H 8.59190 -6.48570 -1.09480

H 11.42350 -5.63960 -1.05090

H 10.31240 -5.28190 -2.37970

H 13.55540 -8.40760 -3.63850

H 12.40310 -9.44810 -2.73540

H 11.90760 -8.70380 -4.29020

H 7.13490 -3.25910 2.78490

H 6.59900 -3.79930 1.17890

H 5.83950 -4.44900 2.63870

H 7.13600 -5.91180 4.23770

H 8.73730 -6.53280 3.80430

H 8.54160 -4.83300 4.27610

H 7.88560 -7.35620 1.51630

H 6.29610 -6.84980 2.11410

H 6.85430 -6.28970 0.53900

1. N0110_Anti-120WP

N 9.57070 -3.60850 2.12200

C 9.15130 -4.91280 1.57920

C 9.10120 -4.89510 0.04470

N 9.42530 -6.15000 -0.59800

C 10.59640 -6.04810 -1.49780

C 10.21040 -5.43130 -2.84870

O 11.09100 -4.50530 -3.24970

C 10.84680 -3.84470 -4.52190

O 9.21710 -5.74370 -3.49080

O 8.84740 -3.88040 -0.58720

C 7.80340 -5.42390 2.23200

C 6.70270 -4.34800 2.14340

C 7.29760 -6.71050 1.54360

C 8.08600 -5.75460 3.71270

C 11.31520 -7.44300 -1.63670

C 12.52500 -7.31120 -2.58490

C 11.82510 -7.85240 -0.23860

C 10.35830 -8.52900 -2.16550

H 8.99070 -2.86700 1.72510

H 10.51920 -3.40520 1.79770

H 9.92110 -5.64750 1.84380

H 8.61080 -6.45770 -1.13770

H 11.32060 -5.37000 -1.03320

H 11.66300 -3.13060 -4.63300

H 10.86080 -4.57680 -5.33240

H 9.88430 -3.32910 -4.49470

H 6.96390 -3.44550 2.70650

H 6.49690 -4.06110 1.10560

H 5.77190 -4.74230 2.56870

H 6.44360 -7.10780 2.10450

H 6.95120 -6.52720 0.51970

H 8.07190 -7.48330 1.50920

H 7.16230 -6.08070 4.20530

H 8.81690 -6.56750 3.80050

H 8.47280 -4.88620 4.25290

H 13.08680 -8.25260 -2.59370

H 12.22430 -7.09740 -3.61670

H 13.20510 -6.51680 -2.25510

H 12.33020 -8.82350 -0.29800

H 12.54500 -7.12300 0.15330

H 10.99880 -7.93450 0.47250

H 9.49890 -8.66750 -1.50070

H 9.98680 -8.29220 -3.16740

H 10.88960 -9.48650 -2.22150

1. N011'0_Anti-120WP

N 9.36910 -3.68420 2.19010

C 8.94650 -4.95290 1.56320

C 9.10100 -4.89450 0.03770

N 9.42680 -6.15600 -0.59510

C 10.66410 -6.30600 -1.41590

C 10.65150 -7.80230 -1.76310

O 11.78030 -8.42300 -1.39880

C 11.89480 -9.84310 -1.70010

O 9.71370 -8.37100 -2.30380

O 8.84600 -3.87500 -0.58310

C 7.48610 -5.37130 2.00830

C 6.47250 -4.23740 1.75380

C 7.53250 -5.69050 3.51760

C 7.01280 -6.63930 1.26530

C 10.91850 -5.41180 -2.69700

C 12.01590 -6.08790 -3.55460

C 11.47170 -4.03400 -2.26840

C 9.64670 -5.23780 -3.54660

H 8.91580 -2.90150 1.71460

H 10.37000 -3.55300 2.02690

H 9.61910 -5.74350 1.91720

H 8.63290 -6.38110 -1.20420

H 11.10460 -10.39840 -1.18990

H 11.83030 -10.00140 -2.77900

H 12.87640 -10.13480 -1.32590

H 5.47210 -4.56920 2.05720

H 6.70760 -3.33710 2.33140

H 6.42370 -3.96470 0.69410

H 7.87570 -4.83060 4.09900

H 6.53190 -5.96680 3.87080

H 8.20460 -6.53310 3.72000

H 7.74300 -7.45080 1.34500

H 6.06910 -6.98430 1.70320

H 6.82330 -6.45370 0.20190

H 11.67860 -7.03180 -3.99820

H 12.29260 -5.42030 -4.37830

H 12.91990 -6.28480 -2.96650

H 12.38470 -4.14820 -1.67080

H 11.72910 -3.44970 -3.15950

H 10.75060 -3.45700 -1.69040

H 8.86420 -4.70930 -2.99540

H 9.88270 -4.64930 -4.44110

H 9.24960 -6.20390 -3.87630

H 11.51570 -6.14880 -0.74480

1. N1000_Anti-120WP

N 8.71950 -3.75520 2.30530

C 9.00370 -4.97990 1.56310

C 9.09960 -4.88930 0.05000

N 9.42640 -6.15430 -0.60200

C 10.65060 -6.07190 -1.42850

C 11.91930 -6.12100 -0.58150

O 12.99510 -5.81050 -1.32230

C 14.29460 -5.82470 -0.66440

O 11.97340 -6.40140 0.60510

O 8.84780 -3.88190 -0.58850

C 9.86340 -2.90740 2.77480

C 10.70630 -3.68700 3.80190

C 9.23150 -1.68920 3.47400

C 10.77390 -2.41120 1.62950

H 8.08980 -3.17090 1.75280

H 8.20940 -5.72530 1.73820

H 9.91400 -5.46660 1.92550

H 8.64780 -6.34730 -1.24570

H 10.69330 -5.17520 -2.06170

H 10.67600 -6.93940 -2.09970

H 14.51550 -6.82660 -0.29030

H 15.00700 -5.53510 -1.43710

H 14.30660 -5.10480 0.15710

H 11.22260 -4.54560 3.35890

H 11.47800 -3.02870 4.21680

H 10.08410 -4.04550 4.62950

H 8.62400 -2.00200 4.33040

H 10.01210 -1.01130 3.83720

H 8.59210 -1.12440 2.78400

H 10.20040 -1.83610 0.89480

H 11.56590 -1.76630 2.02910

H 11.26330 -3.24020 1.10780

1. N1010_Anti-120WP

N 8.82510 -3.71830 2.20830

C 9.15330 -4.97410 1.54140

C 9.10120 -4.89520 0.03880

N 9.42630 -6.15390 -0.59540

C 10.66300 -6.08520 -1.42060

C 10.29510 -5.84070 -2.88830

O 11.18570 -5.07060 -3.51980

C 10.97440 -4.81770 -4.93830

O 9.29770 -6.30370 -3.42580

O 8.84640 -3.87630 -0.58390

C 11.56560 -7.36490 -1.21890

C 10.79550 -8.65590 -1.55460

C 12.82970 -7.27280 -2.10070

C 12.00960 -7.39930 0.25880

C 9.07080 -3.67860 3.68510

C 10.53580 -4.00360 4.06070

C 8.74470 -2.24520 4.13750

C 8.12400 -4.66780 4.38780

H 9.37900 -2.97480 1.77580

H 8.46210 -5.77700 1.83340

H 10.15940 -5.36550 1.77020

H 8.63820 -6.41930 -1.19620

H 11.26050 -5.22710 -1.09330

H 10.02810 -4.29200 -5.08620

H 11.81490 -4.19390 -5.24350

H 10.97310 -5.76080 -5.48860

H 11.43510 -9.52510 -1.36190

H 9.89710 -8.75960 -0.93660

H 10.49690 -8.68900 -2.60820

H 13.37300 -6.33670 -1.92590

H 12.60030 -7.34130 -3.16940

H 13.50250 -8.10230 -1.85400

H 12.59110 -6.50650 0.52070

H 11.15160 -7.46270 0.93360

H 12.64520 -8.27450 0.43410

H 10.68660 -3.86730 5.13790

H 10.80230 -5.03960 3.82540

H 11.23220 -3.33840 3.53580

H 9.40120 -1.51910 3.64150

H 7.70510 -1.98960 3.90380

H 8.88920 -2.14450 5.21870

H 8.34410 -5.70670 4.11970

H 8.23580 -4.58080 5.47400

H 7.07900 -4.45660 4.13410

1. N1100_Anti-120WP

N 8.75450 -3.57790 2.11200

C 8.94420 -4.93410 1.58540

C 9.10150 -4.89660 0.05440

N 9.42270 -6.13970 -0.60190

C 10.73500 -6.14290 -1.27350

C 10.93550 -7.47180 -1.98370

O 12.23030 -7.70430 -2.23160

C 12.56880 -8.91960 -2.95960

O 10.03210 -8.22240 -2.31530

O 8.84970 -3.88910 -0.59310

C 7.78010 -5.93170 2.05180

C 7.51670 -5.71470 3.55720

C 8.19070 -7.41000 1.88220

C 6.48170 -5.64390 1.27430

C 9.89210 -2.90160 2.80480

C 9.38980 -1.48830 3.15200

C 10.25070 -3.63330 4.11130

C 11.15390 -2.78960 1.91770

H 8.46740 -2.97870 1.33890

H 9.87370 -5.37280 1.97500

H 8.71700 -6.25120 -1.33870

H 11.53520 -6.01480 -0.53670

H 10.84920 -5.34640 -2.02810

H 13.65580 -8.90830 -3.04110

H 12.23250 -9.79450 -2.39920

H 12.10630 -8.90340 -3.94880

H 6.72010 -6.39550 3.88030

H 8.40730 -5.94010 4.15400

H 7.21150 -4.68950 3.77360

H 7.41640 -8.05300 2.31840

H 8.33310 -7.68790 0.83840

H 9.12990 -7.61420 2.41220

H 6.58090 -5.87280 0.20620

H 5.66280 -6.25880 1.66570

H 6.18660 -4.59270 1.37210

H 10.16980 -0.91820 3.66880

H 9.11180 -0.93510 2.24610

H 8.51140 -1.54240 3.80490

H 11.04930 -3.08930 4.62840

H 9.38610 -3.68950 4.77940

H 10.61390 -4.65120 3.93070

H 10.93360 -2.24610 0.99180

H 11.94830 -2.25060 2.44770

H 11.55100 -3.77670 1.65310

1. N1110_Anti-120WP

N 9.40860 -3.60550 2.16690

C 9.10500 -4.91340 1.58380

C 9.10110 -4.89500 0.04670

N 9.42510 -6.14940 -0.59890

C 10.62010 -6.11090 -1.47290

C 10.21000 -5.77850 -2.91390

O 11.10130 -5.00450 -3.54150

C 10.84030 -4.67340 -4.93520

O 9.18140 -6.18540 -3.43790

O 8.84760 -3.88120 -0.58830

C 7.72510 -5.47630 2.15300

C 7.89900 -5.67810 3.67140

C 7.34460 -6.83500 1.53030

C 6.59040 -4.46660 1.90180

C 11.43310 -7.46120 -1.37410

C 12.64940 -7.42430 -2.32250

C 11.94500 -7.59830 0.07460

C 10.54970 -8.67630 -1.71550

C 10.79710 -3.30270 2.60990

C 11.16570 -4.17370 3.82650

C 11.85430 -3.49540 1.49880

C 10.78800 -1.82610 3.04390

H 9.09250 -2.87410 1.53220

H 9.86660 -5.64050 1.87880

H 8.61870 -6.44790 -1.15370

H 11.28650 -5.31160 -1.13540

H 11.68270 -4.05420 -5.24350

H 10.79320 -5.58660 -5.53270

H 9.90220 -4.12040 -5.01690

H 8.66490 -6.43340 3.88640

H 8.18820 -4.74530 4.16080

H 6.95540 -6.02380 4.11020

H 6.48160 -7.24760 2.06600

H 7.05640 -6.74960 0.47720

H 8.16470 -7.55680 1.59970

H 6.42130 -4.30130 0.83150

H 5.65530 -4.84580 2.33040

H 6.81010 -3.50270 2.37060

H 13.27000 -8.31120 -2.15040

H 12.35510 -7.42860 -3.37780

H 13.27190 -6.53930 -2.14490

H 12.49390 -8.54000 0.18740

H 12.62880 -6.78150 0.33580

H 11.11540 -7.59730 0.78660

H 9.68790 -8.74390 -1.04350

H 10.18380 -8.63870 -2.74710

H 11.13530 -9.59610 -1.60210

H 12.16890 -3.91200 4.18160

H 10.45730 -4.01420 4.64560

H 11.17410 -5.24210 3.58460

H 12.84970 -3.21070 1.85940

H 11.91560 -4.54210 1.18120

H 11.62030 -2.87850 0.62330

H 10.05340 -1.66540 3.84030

H 11.77480 -1.52940 3.41590

H 10.53380 -1.17030 2.20170

1. N111'0_Anti-120WP

N 8.66480 -3.61260 2.11080

C 8.81290 -4.96520 1.55990

C 9.10150 -4.89670 0.04680

N 9.42410 -6.14510 -0.59850

C 10.73080 -6.39350 -1.27320

C 10.59980 -7.85920 -1.71520

O 11.60630 -8.62340 -1.27410

C 11.59920 -10.03030 -1.65130

O 9.67240 -8.28610 -2.38900

O 8.84830 -3.88370 -0.58890

C 7.55220 -5.88830 1.90480

C 7.24810 -5.73220 3.41080

C 7.84170 -7.38620 1.66490

C 6.31850 -5.44700 1.09480

C 11.22780 -5.47070 -2.45680

C 11.79920 -4.15630 -1.88080

C 12.38790 -6.19520 -3.18090

C 10.11090 -5.16600 -3.47100

C 9.81150 -3.00440 2.84940

C 11.11600 -2.96660 2.02020

C 9.38150 -1.56310 3.17760

C 10.06620 -3.75970 4.16620

H 8.42890 -2.98910 1.33980

H 9.67240 -5.47140 2.01990

H 8.70710 -6.25050 -1.32600

H 10.70350 -10.51650 -1.25860

H 11.63390 -10.12550 -2.73900

H 12.49780 -10.45060 -1.19840

H 6.38660 -6.35930 3.66960

H 8.09620 -6.06240 4.02250

H 7.02220 -4.69720 3.67360

H 7.01560 -7.98130 2.07310

H 7.95770 -7.63380 0.60990

H 8.76140 -7.69340 2.17990

H 6.45200 -5.61090 0.01840

H 5.43780 -6.02040 1.40710

H 6.09920 -4.38450 1.25290

H 12.22630 -3.55660 -2.69310

H 11.03730 -3.55070 -1.38910

H 12.60200 -4.35890 -1.16050

H 12.82360 -5.52240 -3.92790

H 13.18380 -6.48020 -2.48270

H 12.05280 -7.09540 -3.70880

H 9.70470 -6.08320 -3.91010

H 9.29220 -4.60750 -3.00950

H 10.51630 -4.55520 -4.28620

H 11.91560 -2.47520 2.58720

H 11.46720 -3.97450 1.76960

H 10.97080 -2.41050 1.08760

H 10.17150 -1.04070 3.72840

H 9.17840 -0.99500 2.26120

H 8.47360 -1.56230 3.79080

H 10.37850 -4.79590 3.99430

H 10.86910 -3.26560 4.72530

H 9.16760 -3.76960 4.79070

H 11.50790 -6.36570 -0.50140

1. N0010_Syn-60WP

N 8.89580 -3.56230 2.17150

C 9.03460 -4.90110 1.58170

C 9.09980 -4.89020 0.06420

N 9.42210 -6.13720 -0.60790

C 10.71070 -6.76600 -0.20820

C 11.84120 -5.75270 -0.44170

O 12.79000 -5.85030 0.50050

C 13.94630 -4.97350 0.38470

O 11.88480 -4.95170 -1.36120

O 8.85200 -3.89810 -0.59670

C 10.97560 -8.12430 -0.97640

C 12.09540 -8.88950 -0.23520

C 9.69440 -8.98640 -0.93490

C 11.39240 -7.90550 -2.44420

H 9.67300 -2.97160 1.86430

H 9.00310 -3.64510 3.18590

H 8.15670 -5.51770 1.83400

H 9.90090 -5.45450 1.96920

H 8.65650 -6.79170 -0.42210

H 10.72440 -7.02050 0.85950

H 14.50590 -5.21110 -0.52300

H 13.62340 -3.93030 0.36530

H 14.54640 -5.17580 1.27210

H 11.81270 -9.09900 0.80390

H 12.27560 -9.84760 -0.73580

H 13.03960 -8.33580 -0.22490

H 9.31400 -9.10450 0.08760

H 8.89690 -8.57530 -1.56410

H 9.92390 -9.98620 -1.32010

H 10.66340 -7.29680 -2.98830

H 12.37070 -7.41880 -2.52170

H 11.46790 -8.87650 -2.94700

1. N0100_Syn-60WP

N 8.43000 -6.11080 2.15600

C 8.86550 -4.81040 1.61120

C 9.10050 -4.89340 0.07700

N 9.41830 -6.12230 -0.61270

C 10.61260 -6.90130 -0.26920

C 11.93560 -6.34940 -0.79750

O 11.91770 -5.04530 -1.06560

C 13.16900 -4.42370 -1.48270

O 12.93470 -7.05220 -0.89780

O 8.85500 -3.90990 -0.60480

C 9.97700 -4.04340 2.44720

C 9.39670 -3.79950 3.85890

C 10.29500 -2.67080 1.81780

C 11.28480 -4.84810 2.58100

H 9.20910 -6.68220 2.48870

H 7.84000 -5.94810 2.97470

H 7.98650 -4.15580 1.65350

H 8.58730 -6.72100 -0.62770

H 10.49950 -7.89770 -0.70650

H 10.76970 -7.05730 0.80910

H 13.93270 -4.57080 -0.71480

H 13.49990 -4.85060 -2.43270

H 12.93550 -3.36510 -1.59660

H 9.19800 -4.73670 4.38850

H 10.11020 -3.22520 4.46080

H 8.46150 -3.22930 3.80960

H 9.38940 -2.06450 1.70320

H 10.98710 -2.12460 2.47030

H 10.76730 -2.76030 0.83520

H 11.80810 -4.93770 1.62480

H 11.96440 -4.33410 3.27060

H 11.11900 -5.85280 2.98700

1. N0110_Syn-60WP

N 9.73140 -3.57460 2.02260

C 9.05840 -4.82030 1.60580

C 9.09980 -4.89060 0.06970

N 9.42080 -6.13180 -0.61030

C 10.75900 -6.69430 -0.31680

C 11.78620 -5.98470 -1.21930

O 12.91550 -5.71070 -0.54940

C 14.00990 -5.10560 -1.29370

O 11.61750 -5.70970 -2.39600

O 8.85330 -3.90310 -0.60000

C 7.57210 -5.00910 2.14420

C 6.68580 -3.79340 1.81520

C 6.92790 -6.27730 1.54460

C 7.65210 -5.19010 3.67570

C 10.80280 -8.26130 -0.49960

C 12.23860 -8.76050 -0.22440

C 9.86390 -8.91140 0.54220

C 10.37290 -8.69680 -1.91470

H 9.63950 -3.43940 3.03120

H 9.28410 -2.76920 1.58140

H 9.63710 -5.65700 2.01470

H 8.69280 -6.81970 -0.41430

H 11.06560 -6.49910 0.71940

H 14.81670 -4.98780 -0.56960

H 14.31590 -5.76550 -2.10890

H 13.70050 -4.13580 -1.68950

H 5.67960 -3.95640 2.21830

H 7.07440 -2.87220 2.26220

H 6.59230 -3.63690 0.73570

H 5.94770 -6.44640 2.00500

H 6.75800 -6.18930 0.46380

H 7.53370 -7.17010 1.73690

H 6.64750 -5.34610 4.08500

H 8.26370 -6.06100 3.93970

H 8.07460 -4.31170 4.17610

H 12.25040 -9.85570 -0.25080

H 12.95210 -8.40580 -0.97570

H 12.59400 -8.44310 0.76310

H 9.98350 -9.99970 0.50640

H 10.10070 -8.58190 1.56090

H 8.80500 -8.70740 0.34650

H 9.35040 -8.38280 -2.15030

H 11.03990 -8.29340 -2.68230

H 10.40660 -9.79040 -1.97980

1. N011'0_Syn-60WP

N 8.59160 -6.13220 2.18950

C 8.88600 -4.81080 1.60910

C 9.10080 -4.89450 0.07270

N 9.41860 -6.12330 -0.61050

C 10.68630 -6.82330 -0.31780

C 10.37020 -8.32140 -0.23670

O 11.32260 -9.01560 0.39050

C 11.16850 -10.46240 0.45430

O 9.35710 -8.82880 -0.69950

O 8.85450 -3.90760 -0.60270

C 9.93810 -3.92230 2.40570

C 9.34820 -3.68100 3.81310

C 10.14270 -2.54970 1.72990

C 11.31150 -4.60880 2.55810

C 11.81950 -6.50570 -1.39020

C 13.19180 -7.01550 -0.89830

C 11.92300 -4.97710 -1.57290

C 11.48580 -7.14510 -2.75050

H 8.01680 -6.01350 3.02740

H 9.43250 -6.62010 2.49970

H 7.94890 -4.24300 1.65880

H 8.62750 -6.76360 -0.50800

H 12.04530 -10.81530 0.99630

H 10.25130 -10.71660 0.98950

H 11.14490 -10.87900 -0.55550

H 10.01050 -3.02330 4.38780

H 8.36440 -3.19970 3.75250

H 9.24040 -4.61340 4.37580

H 10.77420 -1.92100 2.36950

H 10.63780 -2.63250 0.75910

H 9.18960 -2.03140 1.57980

H 11.83850 -4.69800 1.60170

H 11.94980 -4.00730 3.21620

H 11.23720 -5.60600 3.00990

H 13.96530 -6.69520 -1.60590

H 13.44930 -6.59940 0.08350

H 13.23450 -8.10530 -0.82450

H 12.73720 -4.75380 -2.27190

H 11.00240 -4.55320 -1.97960

H 12.15610 -4.46700 -0.63030

H 12.22780 -6.83550 -3.49540

H 11.50310 -8.23980 -2.70670

H 10.50030 -6.82830 -3.10420

H 11.09560 -6.53390 0.65780

1. N1000_Syn-60WP

N 8.70230 -6.06730 2.21800

C 9.13750 -4.82200 1.59040

C 9.10040 -4.89290 0.06590

N 9.42080 -6.13200 -0.60790

C 10.78050 -6.62490 -0.29400

C 11.02520 -7.94260 -1.01130

O 12.33180 -8.22130 -1.08770

C 12.71570 -9.48650 -1.70320

O 10.14550 -8.67620 -1.43760

O 8.85260 -3.90060 -0.59840

C 8.98470 -6.23710 3.67260

C 8.34060 -7.57400 4.07760

C 8.38420 -5.10040 4.53050

C 10.50420 -6.32000 3.90750

H 7.69490 -6.17310 2.07650

H 10.19020 -4.62370 1.84110

H 8.57320 -3.92280 1.87930

H 8.76400 -6.83710 -0.24900

H 10.93960 -6.80740 0.78060

H 11.52870 -5.89350 -0.62380

H 12.25660 -10.31970 -1.16490

H 12.41030 -9.49750 -2.75410

H 13.80190 -9.52500 -1.61910

H 7.25710 -7.55210 3.91130

H 8.76360 -8.39760 3.49260

H 8.51450 -7.77620 5.14080

H 8.54520 -5.30080 5.59640

H 8.84450 -4.13190 4.30370

H 7.30350 -5.01670 4.36180

H 10.70090 -6.53360 4.96440

H 10.94830 -7.12240 3.30850

H 11.01580 -5.38010 3.67020

1. N1010_Syn-60WP

N 8.17350 -5.93790 2.09960

C 9.02550 -4.85700 1.59450

C 9.10060 -4.89340 0.06520

N 9.42080 -6.13190 -0.60750

C 10.66440 -6.79840 -0.14920

C 11.86420 -5.90310 -0.49010

O 12.79920 -5.96210 0.46860

C 14.03150 -5.21940 0.26070

O 11.97360 -5.22500 -1.49880

O 8.85250 -3.90020 -0.59820

C 10.80600 -8.23900 -0.78150

C 9.58100 -9.08150 -0.36120

C 10.89250 -8.19200 -2.31780

C 12.07120 -8.92410 -0.21910

C 8.33200 -6.31460 3.53720

C 7.18710 -7.29440 3.84890

C 9.67820 -7.03340 3.74640

C 8.24030 -5.09740 4.48440

H 7.19750 -5.67420 1.94450

H 10.04670 -4.98090 1.97910

H 8.70430 -3.84450 1.87400

H 8.63340 -6.76030 -0.43210

H 10.67350 -6.94310 0.93740

H 14.52600 -5.56050 -0.65250

H 13.81580 -4.15000 0.19610

H 14.64610 -5.43690 1.13510

H 9.71500 -10.11260 -0.70680

H 9.46390 -9.10600 0.72700

H 8.64620 -8.71850 -0.80200

H 10.93530 -9.21450 -2.70920

H 10.02130 -7.69670 -2.75660

H 11.79080 -7.66700 -2.65690

H 12.99110 -8.41920 -0.53040

H 12.05800 -8.96230 0.87660

H 12.11790 -9.95430 -0.59040

H 7.24940 -7.63660 4.88780

H 6.21040 -6.81300 3.70940

H 7.23750 -8.17020 3.19220

H 9.76720 -7.35890 4.78900

H 9.74850 -7.91910 3.10610

H 10.53640 -6.38270 3.54070

H 7.29400 -4.56160 4.34070

H 8.29020 -5.42440 5.52920

H 9.06380 -4.39250 4.32140

1. N1100_Syn-60WP

N 9.23740 -3.48550 2.13280

C 8.99830 -4.83280 1.61730

C 9.09970 -4.89020 0.07610

N 9.41970 -6.12760 -0.61320

C 10.72480 -6.75150 -0.34060

C 10.70780 -8.17150 -0.88890

O 11.93740 -8.67690 -1.00310

C 12.05240 -10.05660 -1.46040

O 9.68570 -8.78310 -1.16030

O 8.85450 -3.90760 -0.60340

C 7.59180 -5.37300 2.14650

C 6.45810 -4.39550 1.78410

C 7.70300 -5.48190 3.68160

C 7.24740 -6.77180 1.59490

C 10.60770 -3.05510 2.51760

C 11.63310 -3.16030 1.36550

C 10.47420 -1.57860 2.93720

C 11.09740 -3.87240 3.72770

H 8.85060 -2.80770 1.47820

H 9.74380 -5.52080 2.03030

H 8.68650 -6.81640 -0.43830

H 10.97620 -6.83900 0.72970

H 11.53070 -6.18790 -0.82270

H 11.56050 -10.72470 -0.75030

H 11.60250 -10.15700 -2.44970

H 13.12320 -10.25430 -1.50070

H 5.50670 -4.77070 2.17940

H 6.63400 -3.40630 2.21710

H 6.34590 -4.28450 0.69940

H 6.74280 -5.80150 4.10360

H 8.46100 -6.22010 3.97100

H 7.97420 -4.51960 4.12130

H 8.07380 -7.48000 1.73180

H 6.38390 -7.17300 2.13780

H 6.96050 -6.74980 0.53630

H 12.61010 -2.77840 1.68410

H 11.78050 -4.19930 1.04840

H 11.30620 -2.57780 0.49670

H 11.44410 -1.17810 3.25160

H 10.11080 -0.96480 2.10320

H 9.76920 -1.47840 3.76940

H 10.39300 -3.78540 4.56160

H 11.21960 -4.93520 3.48960

H 12.07280 -3.49800 4.05860

1. N1110_Syn-60WP

N 8.44740 -6.05280 2.19810

C 8.89920 -4.78590 1.62950

C 9.09990 -4.89110 0.08030

N 9.41890 -6.12430 -0.61480

C 10.64810 -6.84580 -0.23590

C 11.83870 -6.15320 -0.92630

O 12.91990 -6.13420 -0.13410

C 14.14840 -5.57560 -0.67460

O 11.82410 -5.68160 -2.05290

O 8.85510 -3.91010 -0.60600

C 10.07100 -4.09080 2.45150

C 11.22920 -5.03860 2.82910

C 9.45720 -3.54960 3.76480

C 10.64540 -2.89190 1.66950

C 10.57900 -8.37800 -0.61910

C 9.46800 -9.05160 0.21490

C 10.29410 -8.59270 -2.11810

C 11.91770 -9.05540 -0.25240

C 6.99130 -6.26530 2.44700

C 6.22060 -6.23160 1.11480

C 6.86550 -7.66350 3.07720

C 6.37330 -5.23000 3.41620

H 8.96050 -6.28570 3.04290

H 8.09440 -4.03990 1.66630

H 8.61360 -6.74240 -0.50500

H 10.82750 -6.81440 0.84300

H 14.87490 -5.64930 0.13540

H 14.47490 -6.15770 -1.54030

H 13.99290 -4.53310 -0.96110

H 11.91950 -4.50320 3.49150

H 10.89360 -5.92730 3.37680

H 11.80730 -5.36530 1.96230

H 10.23060 -3.03060 4.34230

H 8.64900 -2.83650 3.56720

H 9.05920 -4.35080 4.39550

H 9.86110 -2.17900 1.39430

H 11.37490 -2.36800 2.29750

H 11.15950 -3.19630 0.75210

H 9.48360 -10.13130 0.02930

H 9.61390 -8.88450 1.28620

H 8.46530 -8.69980 -0.04930

H 10.24390 -9.66780 -2.32510

H 9.33990 -8.15060 -2.42060

H 11.08210 -8.16690 -2.74570

H 12.74700 -8.70040 -0.87180

H 12.18020 -8.88610 0.79810

H 11.82910 -10.13640 -0.40730

H 5.15730 -6.42430 1.29360

H 6.29350 -5.25570 0.62100

H 6.58620 -7.00110 0.42620

H 5.81610 -7.89380 3.29100

H 7.26120 -8.43140 2.40580

H 7.41980 -7.71500 4.02300

H 6.44310 -4.20750 3.02980

H 5.31040 -5.44780 3.57140

H 6.87010 -5.26310 4.39200

1. N111'0_Syn-60WP

N 9.31220 -3.52350 2.16350

C 8.99280 -4.83930 1.61090

C 9.10020 -4.89190 0.06920

N 9.42050 -6.13080 -0.60970

C 10.76770 -6.70270 -0.39160

C 10.61960 -8.11830 0.18030

O 11.64420 -8.47010 0.96210

C 11.64740 -9.82440 1.50170

O 9.66440 -8.84670 -0.05160

O 8.85320 -3.90280 -0.60000

C 7.53940 -5.28800 2.10020

C 7.07990 -6.61640 1.46610

C 6.49870 -4.19880 1.77950

C 7.61820 -5.48740 3.62750

C 11.64490 -6.67180 -1.70920

C 13.07240 -7.18010 -1.41800

C 11.00300 -7.52620 -2.81700

C 11.73920 -5.20670 -2.18180

C 10.68530 -3.17310 2.61540

C 11.12980 -4.10630 3.75640

C 11.73830 -3.19630 1.48440

C 10.58130 -1.73640 3.16310

H 8.97820 -2.80260 1.52670

H 9.68660 -5.58140 2.01850

H 8.69970 -6.83890 -0.47410

H 10.77710 -9.97240 2.14510

H 11.64020 -10.55050 0.68570

H 12.57060 -9.89830 2.07680

H 6.15190 -6.94400 1.94880

H 6.85650 -6.51840 0.39660

H 7.81600 -7.41670 1.60360

H 5.51430 -4.51450 2.14410

H 6.75050 -3.25330 2.26790

H 6.41240 -4.02180 0.70160

H 7.96540 -4.57680 4.12160

H 6.62750 -5.74270 4.02170

H 8.30260 -6.30510 3.88720

H 13.69660 -7.02840 -2.30620

H 13.53370 -6.63070 -0.58810

H 13.09680 -8.24650 -1.17410

H 11.58520 -7.42950 -3.74030

H 10.97640 -8.58890 -2.55090

H 9.97990 -7.19870 -3.02750

H 10.75250 -4.78720 -2.39310

H 12.23020 -4.57400 -1.43270

H 12.33610 -5.15690 -3.09940

H 12.09970 -3.77610 4.14490

H 10.40540 -4.08870 4.57710

H 11.25280 -5.14390 3.42560

H 12.70960 -2.84680 1.85420

H 11.89120 -4.20790 1.09440

H 11.43520 -2.54810 0.65430

H 9.86500 -1.69190 3.99010

H 11.55620 -1.39110 3.52500

H 10.24720 -1.04280 2.38130

H 11.31290 -6.11520 0.35530

1. N0000_Syn60WP

N 8.68940 -3.67040 2.21040

C 9.25600 -4.86710 1.57310

C 9.09970 -4.88990 0.06310

N 9.42250 -6.13870 -0.60760

C 8.58610 -7.32170 -0.30430

C 7.09710 -7.02620 -0.30350

O 6.38410 -8.15480 -0.19690

C 4.94190 -8.02470 -0.03570

O 6.60270 -5.91070 -0.33890

O 8.85170 -3.89680 -0.59610

H 8.81110 -3.75500 3.22300

H 7.67740 -3.65190 2.06110

H 8.86250 -5.80870 1.98120

H 10.34390 -4.89950 1.74270

H 10.40300 -6.37040 -0.41360

H 8.80750 -7.77760 0.67390

H 8.78670 -8.09100 -1.05870

H 4.58030 -9.04290 0.10830

H 4.72040 -7.40970 0.83920

H 4.50120 -7.58070 -0.93080

1. N0010_Syn60WP

N 9.43720 -5.86300 2.44560

C 9.19510 -4.69300 1.59450

C 9.10120 -4.89600 0.07430

N 9.41820 -6.12180 -0.61080

C 8.69850 -7.39030 -0.32470

C 9.40950 -8.46430 -1.16280

O 9.50240 -9.62680 -0.50380

C 10.06260 -10.75910 -1.22590

O 9.83530 -8.30080 -2.29620

O 8.85450 -3.90770 -0.60400

C 7.14890 -7.35350 -0.65890

C 6.56320 -8.77590 -0.49020

C 6.41450 -6.43940 0.34930

C 6.86720 -6.86260 -2.09170

H 8.55490 -6.27650 2.75140

H 9.89040 -5.54840 3.30550

H 10.04400 -4.00360 1.69680

H 8.30750 -4.10950 1.87630

H 10.43130 -6.26220 -0.52330

H 8.79000 -7.69420 0.72380

H 9.44960 -10.98030 -2.10340

H 11.08840 -10.53950 -1.53010

H 10.03680 -11.58920 -0.51970

H 6.78850 -9.19360 0.49790

H 5.47340 -8.72880 -0.59380

H 6.93370 -9.47250 -1.24950

H 6.59770 -6.74760 1.38590

H 6.67110 -5.38150 0.24110

H 5.33470 -6.51260 0.17520

H 7.23570 -5.84590 -2.25560

H 7.32400 -7.51800 -2.84020

H 5.78410 -6.86200 -2.26410

1. N0100_Syn60WP

N 10.28390 -3.86070 1.87320

C 9.15560 -4.79030 1.61480

C 9.09950 -4.88940 0.07340

N 9.42040 -6.13020 -0.61220

C 8.53110 -7.27410 -0.34480

C 8.50340 -8.25200 -1.51750

O 7.58620 -9.20600 -1.29610

C 7.38780 -10.21670 -2.32540

O 9.19630 -8.19330 -2.51930

O 8.85400 -3.90580 -0.60170

C 7.79500 -4.42070 2.32830

C 8.06540 -4.43000 3.84990

C 6.72310 -5.48700 2.02300

C 7.24880 -3.03730 1.92400

H 10.03960 -2.91540 1.56940

H 10.47390 -3.80460 2.87510

H 9.45880 -5.77010 2.00420

H 10.38210 -6.38210 -0.35040

H 7.50070 -6.92600 -0.22720

H 8.78550 -7.84420 0.55980

H 6.99900 -9.74930 -3.23340

H 8.32710 -10.73200 -2.53540

H 6.65420 -10.90730 -1.90800

H 8.74890 -3.62860 4.15060

H 7.12720 -4.27950 4.39540

H 8.49450 -5.38640 4.17190

H 7.07890 -6.49600 2.26040

H 5.83040 -5.29950 2.63120

H 6.40560 -5.46110 0.97380

H 6.94650 -3.00920 0.87350

H 6.36710 -2.80710 2.53420

H 7.98020 -2.23880 2.09110

1. N0110_Syn60WP

N 10.57690 -4.17890 1.79730

C 9.24890 -4.80750 1.60670

C 9.10010 -4.89180 0.06710

N 9.42060 -6.13120 -0.60880

C 8.56090 -7.30570 -0.32060

C 9.49230 -8.49270 -0.03370

O 8.86690 -9.51620 0.54610

C 9.64260 -10.72170 0.80470

O 10.68510 -8.49760 -0.30740

O 8.85310 -3.90250 -0.59880

C 8.04330 -4.15230 2.37760

C 7.83180 -2.66410 2.03280

C 6.74770 -4.93160 2.06760

C 8.33610 -4.28110 3.88850

C 7.52560 -7.60690 -1.48690

C 6.47530 -8.64380 -1.02910

C 6.77240 -6.30720 -1.83740

C 8.25000 -8.12190 -2.74440

H 10.86680 -4.25220 2.77400

H 10.54260 -3.18290 1.57160

H 9.34000 -5.83170 1.98900

H 10.39670 -6.36580 -0.40030

H 7.96870 -7.14890 0.59110

H 8.93820 -11.41840 1.25900

H 10.03070 -11.12220 -0.13430

H 10.46360 -10.49680 1.48870

H 7.04270 -2.25170 2.67320

H 8.73720 -2.07100 2.20760

H 7.53140 -2.52740 0.99120

H 5.91990 -4.54000 2.67080

H 6.45360 -4.83450 1.01680

H 6.85040 -5.99760 2.30790

H 7.48590 -3.90420 4.46810

H 8.50100 -5.32750 4.17280

H 9.21760 -3.70180 4.18500

H 5.70350 -8.73460 -1.80250

H 6.90400 -9.63510 -0.86620

H 5.97970 -8.32990 -0.10180

H 6.04600 -6.51710 -2.63060

H 6.21350 -5.91920 -0.97780

H 7.44730 -5.52600 -2.19340

H 9.01560 -7.41470 -3.07940

H 8.72740 -9.09430 -2.57450

H 7.52840 -8.24940 -3.55940

1. N011'0_Syn60WP

N 10.47560 -3.94890 1.78440

C 9.26230 -4.78170 1.60790

C 9.09980 -4.89050 0.07100

N 9.42040 -6.13020 -0.61090

C 8.71010 -7.35760 -0.19660

C 7.26040 -7.29030 -0.71150

O 6.45360 -8.08450 0.00350

C 5.06060 -8.18860 -0.40200

O 6.87870 -6.61550 -1.65440

O 8.85370 -3.90480 -0.60060

C 7.97730 -4.31240 2.38880

C 8.30030 -4.37820 3.89740

C 6.81340 -5.28150 2.09720

C 7.53860 -2.88000 2.02650

C 9.44270 -8.66450 -0.72090

C 8.92220 -9.89220 0.06080

C 10.95920 -8.54680 -0.44840

C 9.21030 -8.88070 -2.23070

H 10.75350 -3.94010 2.76750

H 10.28750 -2.97900 1.52330

H 9.51560 -5.77530 1.99740

H 10.43160 -6.25200 -0.51560

H 4.60960 -8.88210 0.30850

H 4.57710 -7.21050 -0.34450

H 4.99350 -8.58220 -1.41900

H 7.40600 -4.13490 4.48210

H 8.63070 -5.38280 4.18820

H 9.08220 -3.66530 4.18200

H 5.94220 -5.01740 2.70780

H 6.49470 -5.23170 1.04920

H 7.07970 -6.31740 2.33580

H 7.21760 -2.80530 0.98410

H 6.69420 -2.58850 2.66240

H 8.34010 -2.15070 2.19050

H 9.46120 -10.78550 -0.27440

H 9.09440 -9.77900 1.13880

H 7.85440 -10.06470 -0.09720

H 11.43420 -9.51020 -0.66270

H 11.45040 -7.80650 -1.09120

H 11.16580 -8.29970 0.60050

H 9.80790 -9.73250 -2.57370

H 8.16080 -9.10750 -2.45250

H 9.50200 -8.00330 -2.81770

H 8.65610 -7.45530 0.89720

1. N1010_Syn60WP

N 7.67970 -4.98440 1.99850

C 9.08620 -4.83010 1.59140

C 9.10060 -4.89330 0.06090

N 9.42190 -6.13640 -0.60560

C 8.44620 -7.27090 -0.47690

C 9.26950 -8.54840 -0.68220

O 8.82720 -9.55950 0.07210

C 9.47590 -10.85260 -0.09780

O 10.22210 -8.64810 -1.44280

O 8.85140 -3.89580 -0.59580

C 7.21770 -7.20600 -1.47590

C 6.34630 -5.97250 -1.15750

C 7.67030 -7.13720 -2.94540

C 6.33340 -8.45920 -1.27040

C 7.40200 -5.23400 3.44240

C 5.87210 -5.15960 3.59740

C 7.87640 -6.64660 3.82720

C 8.06160 -4.18260 4.36030

H 7.17240 -4.14620 1.71360

H 9.68350 -5.64730 2.00590

H 9.54490 -3.87820 1.89680

H 10.29630 -6.45450 -0.17200

H 8.02710 -7.31480 0.53510

H 9.36010 -11.19620 -1.12680

H 10.53520 -10.77570 0.15330

H 8.96230 -11.52270 0.59170

H 5.42190 -6.03190 -1.74310

H 6.08150 -5.93090 -0.09590

H 6.84410 -5.03730 -1.42280

H 6.78920 -7.06320 -3.59240

H 8.29800 -6.25880 -3.12710

H 8.22900 -8.02770 -3.24550

H 6.83020 -9.38260 -1.58910

H 6.03500 -8.57850 -0.22270

H 5.42260 -8.35310 -1.86740

H 5.58340 -5.35280 4.63640

H 5.49830 -4.16580 3.32240

H 5.38160 -5.90160 2.95650

H 7.60990 -6.86200 4.86890

H 7.39950 -7.39850 3.19090

H 8.96500 -6.75440 3.73810

H 7.74140 -3.17230 4.08790

H 7.77950 -4.35230 5.40560

H 9.15490 -4.22330 4.30020

1. N1100_Syn60WP

N 10.28870 -3.85040 1.98440

C 9.19820 -4.76660 1.62290

C 9.09930 -4.88900 0.07970

N 9.42000 -6.12860 -0.61510

C 8.57750 -7.30910 -0.39750

C 9.07520 -8.28300 0.66960

O 8.17560 -9.24790 0.90400

C 8.54880 -10.31030 1.82920

O 10.16870 -8.22440 1.20800

O 8.85460 -3.90800 -0.60500

C 7.84320 -4.31780 2.31040

C 7.55360 -2.81770 2.10280

C 7.98440 -4.58490 3.82400

C 6.65450 -5.14180 1.77590

C 11.66170 -4.31740 2.30590

C 12.37460 -5.00140 1.11800

C 12.44300 -3.04780 2.69740

C 11.63350 -5.27340 3.51260

H 10.32400 -3.05810 1.34610

H 9.43930 -5.75680 2.01510

H 10.40980 -6.35860 -0.51210

H 8.52700 -7.88950 -1.32970

H 7.54750 -7.01620 -0.17850

H 9.42910 -10.83700 1.45480

H 8.74970 -9.89230 2.81760

H 7.68640 -10.97600 1.86070

H 6.63620 -2.54630 2.64010

H 8.36700 -2.20230 2.50040

H 7.41310 -2.56980 1.04680

H 7.07850 -4.25930 4.35000

H 8.12720 -5.65310 4.03190

H 8.83620 -4.03800 4.23910

H 6.80660 -6.21790 1.92510

H 5.74130 -4.86800 2.31750

H 6.46390 -4.95620 0.71180

H 13.42460 -5.20470 1.35920

H 11.90790 -5.96210 0.87750

H 12.34830 -4.35930 0.22970

H 13.47370 -3.30250 2.96700

H 12.47900 -2.33540 1.86390

H 11.97070 -2.55420 3.55300

H 11.17010 -4.79120 4.37960

H 11.08860 -6.19880 3.29780

H 12.65810 -5.55520 3.78040

1. N1110_Syn60WP

N 8.25610 -5.71330 2.28160

C 9.12790 -4.73670 1.62110

C 9.10080 -4.89440 0.07470

N 9.41880 -6.12420 -0.61140

C 8.55340 -7.31570 -0.44680

C 9.42860 -8.49150 -0.00430

O 8.74350 -9.42230 0.66450

C 9.46070 -10.61650 1.08540

O 10.62800 -8.57710 -0.23890

O 8.85430 -3.90690 -0.60380

C 10.60270 -4.69280 2.19210

C 11.47490 -3.78760 1.29660

C 11.26480 -6.08250 2.30300

C 10.55080 -4.06410 3.60270

C 7.74090 -7.63970 -1.76760

C 6.94190 -6.37970 -2.16130

C 8.68430 -8.02000 -2.92250

C 6.73570 -8.78370 -1.51680

C 6.92590 -5.27640 2.79970

C 6.05330 -4.75180 1.64570

C 6.27230 -6.53440 3.39990

C 7.02020 -4.19180 3.89750

H 8.74850 -6.20860 3.01960

H 8.74120 -3.72100 1.76710

H 10.40310 -6.36970 -0.49560

H 7.83490 -7.13810 0.35540

H 8.71370 -11.23520 1.58470

H 9.87210 -11.13300 0.21540

H 10.26320 -10.34840 1.77670

H 12.46280 -3.65950 1.75250

H 11.62990 -4.21200 0.29780

H 11.02610 -2.79510 1.17650

H 12.25490 -5.96760 2.75870

H 10.69920 -6.77160 2.94110

H 11.40950 -6.56370 1.33220

H 9.95850 -4.66500 4.30030

H 11.56630 -3.99190 4.00920

H 10.12560 -3.05410 3.57500

H 6.35680 -6.58370 -3.06470

H 6.23860 -6.08780 -1.37250

H 7.59990 -5.53200 -2.36470

H 8.10800 -8.15170 -3.84510

H 9.42700 -7.23520 -3.09910

H 9.21470 -8.95890 -2.72540

H 7.22780 -9.74130 -1.32390

H 6.08040 -8.56200 -0.66570

H 6.10120 -8.90580 -2.40250

H 5.06170 -4.48100 2.02440

H 6.48080 -3.85770 1.17900

H 5.91960 -5.51600 0.87390

H 5.28390 -6.29460 3.80750

H 6.15540 -7.31300 2.63920

H 6.88290 -6.94100 4.21620

H 7.48700 -3.27220 3.52790

H 6.01690 -3.92990 4.25380

H 7.59990 -4.55170 4.75480

1. N111'0_Syn60WP

N 10.21000 -3.79510 1.97670

C 9.17130 -4.77890 1.62360

C 9.09910 -4.88780 0.07710

N 9.42080 -6.13200 -0.61430

C 8.52370 -7.28860 -0.38620

C 7.44650 -7.27870 -1.48930

O 6.23710 -7.56210 -0.98840

C 5.12080 -7.64050 -1.91670

O 7.65230 -7.05320 -2.67150

O 8.85400 -3.90570 -0.60330

C 7.80240 -4.40690 2.33040

C 6.67390 -5.37470 1.92690

C 7.36310 -2.96460 2.01040

C 8.02420 -4.53360 3.85270

C 9.29280 -8.66420 -0.33150

C 8.26880 -9.82010 -0.28170

C 10.21960 -8.85970 -1.54740

C 10.12390 -8.71590 0.97010

C 11.61180 -4.18360 2.27740

C 11.66260 -5.04040 3.55610

C 12.31690 -4.93180 1.12300

C 12.35450 -2.85850 2.53470

H 10.19710 -3.01930 1.31790

H 9.47000 -5.75540 2.01630

H 10.37940 -6.38080 -0.37070

H 4.98750 -6.68140 -2.42240

H 5.29840 -8.43130 -2.64960

H 4.25290 -7.87510 -1.29940

H 5.75350 -5.10530 2.45860

H 6.44920 -5.33580 0.85510

H 6.90870 -6.41100 2.19940

H 6.46090 -2.72530 2.58600

H 8.13780 -2.24380 2.28920

H 7.13050 -2.83410 0.94960

H 8.81250 -3.85740 4.19260

H 7.09940 -4.28220 4.38530

H 8.30330 -5.55700 4.13340

H 8.79970 -10.76560 -0.12490

H 7.55610 -9.69300 0.54110

H 7.70210 -9.91170 -1.21440

H 10.72010 -9.83130 -1.46660

H 9.65850 -8.84490 -2.48650

H 11.00090 -8.09360 -1.60580

H 10.91070 -7.95470 1.00480

H 9.48910 -8.59580 1.85610

H 10.62290 -9.68810 1.04720

H 12.70330 -5.28280 3.79920

H 11.22670 -4.49920 4.40170

H 11.12590 -5.98880 3.44300

H 13.38300 -5.06490 1.34080

H 11.89990 -5.93520 0.98260

H 12.22830 -4.37670 0.18170

H 11.88180 -2.30610 3.35370

H 13.39900 -3.05210 2.80110

H 12.34550 -2.22400 1.63950

H 7.99270 -7.20430 0.56660
