# Supplementary material for: Density Functional Studies on Secondary Amides: Role of Steric Factors in Cis/Trans Isomerization
Source: Molecules. 2018 Sep 25;23(10):2455. doi: 10.3390/molecules23102455 (PMC6222500; doi:10.3390/molecules23102455)
Supplement: Supplementary file 1 [file molecules-23-02455-s001.zip › SI_Coordinates.pdf]

Density Functional Studies on Secondary Amides: Role of Steric Factors in cis-trans isomerization

*Balmukund S. Thakkar, John-Sigurd M. Svendsen and Richard A. Engh**

Department of Chemistry, UiT The Arctic University of Norway, Tromsø-9037, Norway

Supporting Information

This document provides Cartesian coordinates (in Å) for the optimized geometries of GGMe (N0000) and its substituted derivatives. The geometries include the minimum energy geometries of trans and cis isomers as well as energy barrier geometries (EBGs) for each compound i.e. "*anti*" type with ω at ~120° and ~(-120)°, and "*syn*" type with ω at 60° and ~(-60)°.

Each trans/cis geometry is named as: [Compound][t/c][a]_[GP/WP][b], while each energy barrier geometry is named as [Compound]_[Anti/Syn][c][ω value][d][GP/WP][b]

1. N0000t_GP

N	9.67400	-6.19120	2.11600
C	9.08180	-4.97260	1.55780
C	9.11010	-4.92990	0.02620
N	9.41290	-6.10200	-0.58000
C	9.44090	-6.22240	-2.01770
C	9.91300	-7.60580	-2.41440
O	9.95840	-7.73010	-3.75240
C	10.39010	-9.01140	-4.25740
O	10.20810	-8.49090	-1.63650
O	8.85080	-3.89370	-0.58670
H	9.24860	-6.44800	3.00000
H	10.67270	-6.09040	2.27080
H	9.54020	-4.03750	1.90510
H	8.02520	-4.92710	1.84370
H	9.60480	-6.91420	-0.00060
H	10.10490	-5.47140	-2.46260
H	8.45080	-6.04530	-2.45830
H	10.35430	-8.91930	-5.34200
H	11.40630	-9.22810	-3.92020
H	9.71800	-9.80040	-3.91260

[a] t: trans, c: cis; [b] GP: Gas phase; WP: water phase (water dielectric continuum)
[c] Anti: EBG of "*anti*" type; Syn: EBG of "*syn*" type
[d] Among 120, -120, 60 or -60, which value (degrees) is closest from the ω of the EBG.

2. N0010t_GP

N	9.18410	-6.30900	2.11330
C	9.15040	-4.95490	1.55340
C	9.10980	-4.92840	0.01930
N	9.41490	-6.10970	-0.57740
C	9.37000	-6.30770	-2.01560
C	8.40920	-7.46310	-2.29820
O	7.67010	-7.24570	-3.40300
C	6.75100	-8.29590	-3.76820
O	8.31440	-8.46420	-1.61430
O	8.84920	-3.88740	-0.58250
C	10.79530	-6.53110	-2.64980
C	10.66130	-6.73590	-4.17190
C	11.63170	-5.26230	-2.38990
C	11.50350	-7.74850	-2.02410
H	8.25080	-6.67790	2.26940
H	9.68090	-6.34090	2.99690
H	10.06510	-4.42620	1.84360
H	8.31000	-4.33800	1.89640
H	9.54010	-6.90540	0.04120
H	8.95030	-5.39550	-2.44690
H	7.29230	-9.22430	-3.96570
H	6.02990	-8.46350	-2.96500
H	6.25110	-7.94090	-4.66870
H	10.11370	-5.91170	-4.64190
H	11.65680	-6.78110	-4.62680
H	10.14300	-7.66730	-4.42110
H	11.16480	-4.37680	-2.83320
H	11.75420	-5.07520	-1.31930
H	12.62960	-5.37840	-2.82750
H	11.65410	-7.61650	-0.94750
H	10.94180	-8.67550	-2.17500
H	12.49200	-7.87510	-2.47970

3. N0100t_GP

N	8.89260	-6.36140	2.01520
C	9.10300	-4.97220	1.57340
C	9.10990	-4.92900	0.03410
N	9.41210	-6.09850	-0.58370
C	9.46020	-6.19920	-2.02240
C	9.65160	-7.64420	-2.43460
O	9.67320	-7.76070	-3.77390
C	9.85910	-9.09480	-4.29230
O	9.77630	-8.57690	-1.66650
O	8.85190	-3.89800	-0.59090
C	10.41000	-4.36090	2.19930
C	10.32330	-4.46040	3.73960
C	10.51190	-2.86780	1.82600
C	11.67110	-5.10400	1.71520
H	8.98060	-6.44400	3.02260
H	7.95460	-6.67340	1.77580
H	8.27880	-4.30400	1.86600
H	9.60640	-6.91940	-0.01880
H	10.27760	-5.60020	-2.44630
H	8.53970	-5.80970	-2.47330
H	10.81480	-9.50240	-3.95500
H	9.04920	-9.74720	-3.95820
H	9.84590	-8.98630	-5.37600
H	10.41390	-5.48980	4.10460
H	11.14410	-3.89470	4.19330
H	9.38410	-4.03820	4.11710
H	9.65160	-2.30420	2.20480
H	11.41740	-2.43880	2.27010
H	10.54960	-2.71770	0.74510
H	11.83210	-4.96460	0.64120
H	12.55540	-4.71750	2.23420
H	11.60460	-6.17860	1.91150

4. N0110t_GP

N	8.85950	-6.35310	2.01470
C	9.09740	-4.96880	1.57130
C	9.10930	-4.92650	0.02730
N	9.41440	-6.10780	-0.58140
C	9.25780	-6.32070	-2.01170
C	8.16520	-7.37420	-2.20750
O	7.37540	-7.09360	-3.26130
C	6.32620	-8.04420	-3.53870
O	8.01340	-8.35100	-1.49800
O	8.85020	-3.89120	-0.58640
C	10.39640	-4.37280	2.22530
C	11.65060	-5.18130	1.83940
C	10.57200	-2.90390	1.78980
C	10.23460	-4.39510	3.76310
C	10.60650	-6.70200	-2.72950
C	10.34190	-6.98680	-4.22170
C	11.55840	-5.49520	-2.61550
C	11.25760	-7.93750	-2.07720
H	7.91080	-6.64490	1.79150
H	8.96550	-6.44260	3.01970
H	8.27730	-4.28950	1.84870
H	9.48880	-6.91490	0.03050
H	8.90400	-5.37760	-2.43460
H	5.80350	-7.65060	-4.40960
H	6.75020	-9.02830	-3.75260
H	5.65070	-8.12200	-2.68370
H	12.52510	-4.79270	2.37330
H	11.54140	-6.24040	2.09240
H	11.85930	-5.11000	0.76770
H	11.46260	-2.48310	2.27030
H	10.68310	-2.80690	0.70810
H	9.70990	-2.29530	2.08470
H	11.05880	-3.84550	4.23020
H	9.29900	-3.91570	4.07490
H	10.25950	-5.40840	4.18020
H	11.29400	-7.13260	-4.74330
H	9.74440	-7.89210	-4.37050
H	9.81700	-6.15530	-4.70430
H	12.51670	-5.72740	-3.09350
H	11.14030	-4.60870	-3.10420
H	11.75710	-5.24020	-1.57100
H	11.51830	-7.74900	-1.03070
H	10.60160	-8.81300	-2.11060
H	12.18400	-8.18980	-2.60520

5. N011'0t_GP

N	9.67810	-6.27420	2.01980
C	9.05320	-5.01500	1.58120
C	9.10960	-4.92790	0.03060
N	9.41320	-6.10290	-0.58250
C	9.54180	-6.23460	-2.02270
C	10.93780	-6.78390	-2.31880
O	11.48380	-6.20980	-3.40880
C	12.79340	-6.68270	-3.78590
O	11.50020	-7.63600	-1.65830
O	8.85110	-3.89470	-0.58860
C	9.55710	-3.71590	2.31370
C	9.58400	-3.98370	3.83510
C	8.58650	-2.54160	2.06350
C	10.97360	-3.33310	1.84010
C	8.39620	-7.10650	-2.66460
C	8.60830	-7.20180	-4.18870
C	7.05420	-6.39830	-2.39190
C	8.36000	-8.52300	-2.05820
H	9.31430	-6.57340	2.91780
H	10.68640	-6.17850	2.11610
H	7.98270	-5.11020	1.81160
H	9.65190	-6.87860	0.02720
H	13.06440	-6.10630	-4.66980
H	13.50580	-6.50940	-2.97630
H	12.76160	-7.75080	-4.01440
H	9.82230	-3.05770	4.36940
H	8.60780	-4.32960	4.19880
H	10.33630	-4.72620	4.12070
H	8.91770	-1.65970	2.62490
H	8.52890	-2.28880	1.00510
H	7.57560	-2.79190	2.41000
H	10.97630	-3.06090	0.78110
H	11.34520	-2.47460	2.41060
H	11.69200	-4.15000	1.98850
H	7.75580	-7.71330	-4.64870
H	8.69230	-6.20970	-4.64560
H	9.50950	-7.76680	-4.44720
H	6.23340	-6.97280	-2.83580
H	6.85930	-6.30850	-1.31950
H	7.03800	-5.39120	-2.82090
H	7.55310	-9.10220	-2.52070
H	9.29780	-9.06400	-2.21760
H	8.16580	-8.49470	-0.98080
H	9.47100	-5.22760	-2.44050

6. N1000t_GP

N	9.13650	-6.31130	2.11400
C	9.14080	-4.95790	1.55960
C	9.11010	-4.92960	0.02730
N	9.41280	-6.10160	-0.58060
C	9.46560	-6.21680	-2.01780
C	9.80340	-7.63990	-2.41160
O	9.84850	-7.76810	-3.74920
C	10.16860	-9.08260	-4.25160
O	10.00990	-8.54860	-1.63200
O	8.85090	-3.89430	-0.58730
C	9.70650	-6.51880	3.47130
C	9.39600	-7.97740	3.84660
C	9.08910	-5.57150	4.52390
C	11.23250	-6.32730	3.41560
H	8.18430	-6.67090	2.10700
H	10.06550	-4.44770	1.84710
H	8.31470	-4.31730	1.89920
H	9.62280	-6.90800	0.00050
H	10.21510	-5.54010	-2.44820
H	8.50940	-5.93380	-2.47540
H	11.15580	-9.39180	-3.90070
H	9.42220	-9.80740	-3.91900
H	10.15700	-8.98590	-5.33650
H	8.31350	-8.15070	3.89400
H	9.81970	-8.66390	3.10710
H	9.81290	-8.22210	4.82870
H	9.49550	-5.77580	5.52070
H	9.29610	-4.52140	4.29240
H	8.00100	-5.69880	4.57300
H	11.67800	-6.55900	4.38870
H	11.67300	-6.99040	2.66480
H	11.51240	-5.29780	3.16970

7. N1010t_GP

N	9.11200	-6.30530	2.11330
C	9.15790	-4.95420	1.55440
C	9.10970	-4.92800	0.02070
N	9.41480	-6.10950	-0.57810
C	9.35670	-6.31010	-2.01560
C	8.41910	-7.48750	-2.28200
O	7.66160	-7.29170	-3.37790
C	6.76220	-8.36400	-3.72800
O	8.35570	-8.48810	-1.59310
O	8.84930	-3.88810	-0.58310
C	10.77820	-6.50340	-2.66880
C	11.58880	-5.21460	-2.42570
C	11.52200	-7.70130	-2.04720
C	10.62890	-6.71850	-4.18810
C	9.67590	-6.52490	3.47130
C	9.31700	-7.97000	3.85520
C	11.20740	-6.38360	3.41350
C	9.09150	-5.55130	4.51870
H	8.14910	-6.63540	2.10800
H	10.10310	-4.47650	1.83120
H	8.35760	-4.28500	1.89950
H	9.52620	-6.90690	0.04120
H	8.91190	-5.40870	-2.44400
H	7.32200	-9.28100	-3.92720
H	6.05380	-8.54370	-2.91620
H	6.24470	-8.02460	-4.62470
H	12.58310	-5.31050	-2.87620
H	11.09610	-4.34200	-2.86640
H	11.72090	-5.01960	-1.35770
H	12.50770	-7.80730	-2.51390
H	11.68130	-7.56060	-0.97300
H	10.98010	-8.64160	-2.18760
H	10.13070	-7.66370	-4.42630
H	10.05460	-5.91060	-4.65430
H	11.61900	-6.74110	-4.65640
H	9.72790	-8.22340	4.83760
H	8.22960	-8.10650	3.90550
H	9.71510	-8.67470	3.11860
H	11.64590	-6.61870	4.38890
H	11.62640	-7.06920	2.67050
H	11.52030	-5.36650	3.15560
H	7.99980	-5.64190	4.56910
H	9.49170	-5.76420	5.51610
H	9.33330	-4.50990	4.28180

8. N1100t_GP

N	9.69340	-3.70790	2.11140
C	9.11630	-4.94530	1.58520
C	9.10940	-4.92750	0.04160
N	9.41130	-6.09540	-0.58740
C	9.38320	-6.21070	-2.02870
C	9.64140	-7.65070	-2.42350
O	9.62400	-7.79610	-3.75700
C	9.85120	-9.13170	-4.25760
O	9.83830	-8.55760	-1.63800
O	8.85310	-3.90260	-0.59470
C	7.67270	-5.19050	2.17720
C	6.74540	-3.99230	1.89530
C	7.80980	-5.37860	3.70060
C	7.05200	-6.46820	1.57680
C	11.16050	-3.61600	2.32330
C	11.99580	-3.95620	1.06660
C	11.43160	-2.15550	2.72650
C	11.56980	-4.53810	3.48640
H	9.40200	-2.95080	1.49530
H	9.73060	-5.79040	1.91680
H	9.58680	-6.94650	-0.06870
H	10.13810	-5.56730	-2.49760
H	8.41630	-5.89070	-2.43640
H	10.83330	-9.49130	-3.94330
H	9.07960	-9.81000	-3.88690
H	9.79980	-9.04320	-5.34190
H	5.75070	-4.18690	2.31280
H	7.13000	-3.08120	2.36330
H	6.63520	-3.80510	0.82310
H	6.82190	-5.51520	4.15530
H	8.41190	-6.26370	3.94150
H	8.28480	-4.50650	4.15590
H	7.70010	-7.34220	1.72120
H	6.09890	-6.68610	2.07100
H	6.84990	-6.36910	0.50550
H	13.06480	-3.81220	1.26190
H	11.85800	-4.99840	0.75920
H	11.71320	-3.31470	0.22520
H	12.49420	-2.00300	2.94260
H	11.15280	-1.46780	1.91900
H	10.85230	-1.89010	3.61620
H	10.99710	-4.29550	4.38660
H	11.40500	-5.59570	3.25370
H	12.63550	-4.41820	3.70890

9. N1110t_GP

N	8.84020	-6.31800	2.06750
C	9.13000	-4.96310	1.57630
C	9.10940	-4.92700	0.02700
N	9.41440	-6.10770	-0.58110
C	9.28960	-6.30420	-2.01660
C	8.23930	-7.38880	-2.25620
O	7.45430	-7.10670	-3.31290
C	6.44840	-8.08820	-3.63900
O	8.11460	-8.39180	-1.57830
O	8.85010	-3.89090	-0.58630
C	10.47070	-4.38420	2.17440
C	10.76420	-2.99020	1.58150
C	11.67270	-5.31080	1.89420
C	10.30450	-4.21430	3.70170
C	10.66260	-6.63130	-2.71720
C	11.57330	-5.39690	-2.56830
C	11.34080	-7.85570	-2.07270
C	10.43330	-6.89900	-4.21850
C	7.44810	-6.66010	2.47380
C	6.49060	-6.46730	1.28540
C	7.47460	-8.14690	2.86830
C	6.95940	-5.82230	3.67730
H	9.46000	-6.55920	2.83320
H	8.34590	-4.25040	1.86420
H	9.41260	-6.91630	0.03500
H	8.91470	-5.36600	-2.43320
H	5.92550	-7.68710	-4.50640
H	6.91500	-9.04720	-3.87690
H	5.76200	-8.22010	-2.79970
H	11.64870	-2.56640	2.07060
H	10.95140	-3.02740	0.50660
H	9.92600	-2.30430	1.74000
H	12.58170	-4.87980	2.32850
H	11.54660	-6.30900	2.32760
H	11.84230	-5.43500	0.82100
H	10.14470	-5.16340	4.22440
H	11.20840	-3.76280	4.12490
H	9.46100	-3.55630	3.93760
H	12.54650	-5.59130	-3.03270
H	11.13520	-4.51660	-3.05060
H	11.74610	-5.15100	-1.51710
H	12.28840	-8.06560	-2.58130
H	11.56650	-7.67810	-1.01630
H	10.71670	-8.75230	-2.13880
H	9.86720	-7.81980	-4.39320
H	9.89160	-6.07590	-4.69680
H	11.39850	-7.00670	-4.72520
H	5.47450	-6.74940	1.58020

H	6.45620	-5.42530	0.95050
H	6.78800	-7.09550	0.44160
H	6.48100	-8.48180	3.18430
H	7.79800	-8.76290	2.02410
H	8.16360	-8.32100	3.70450
H	6.93490	-4.75240	3.44690
H	5.94450	-6.11750	3.96590
H	7.61020	-5.96750	4.54700

10. N111'0t_GP

N	9.64300	-3.67420	2.09290
C	9.11470	-4.94130	1.58480
C	9.10900	-4.92560	0.03610
N	9.41320	-6.10290	-0.58550
C	9.66460	-6.20830	-2.01920
C	11.01110	-6.91520	-2.17120
O	11.71030	-6.46510	-3.22560
C	12.98600	-7.09950	-3.46140
O	11.41090	-7.78670	-1.42070
O	8.85160	-3.89700	-0.59100
C	7.69190	-5.24410	2.19750
C	7.14750	-6.58640	1.66950
C	6.69380	-4.11820	1.86590
C	7.84550	-5.35050	3.72770
C	8.50550	-6.92490	-2.81230
C	8.88040	-7.03570	-4.30400
C	8.22780	-8.32980	-2.24410
C	7.23690	-6.05800	-2.68780
C	11.10510	-3.52950	2.31120
C	11.53370	-4.39560	3.50990
C	11.96110	-3.88980	1.07380
C	11.32810	-2.04750	2.66130
H	9.33290	-2.94220	1.45620
H	9.76870	-5.75550	1.91820
H	9.73840	-6.87970	-0.02450
H	13.64080	-6.96260	-2.59810
H	12.85050	-8.16760	-3.64660
H	13.39490	-6.60320	-4.34050
H	6.21170	-6.83600	2.18150
H	6.93700	-6.55840	0.59680
H	7.84970	-7.40930	1.85650
H	5.71720	-4.34390	2.30950
H	7.03220	-3.16180	2.27600
H	6.55750	-3.99600	0.78720
H	8.25840	-4.42760	4.14070
H	6.87030	-5.53660	4.19230
H	8.50950	-6.17860	4.00570
H	8.02460	-7.42120	-4.86850
H	9.14910	-6.06110	-4.72560
H	9.72040	-7.71660	-4.47310
H	7.41450	-8.80200	-2.80600
H	9.10390	-8.98290	-2.31110
H	7.91890	-8.28420	-1.19500
H	6.92710	-5.94060	-1.64650
H	7.39320	-5.05680	-3.10160
H	6.41180	-6.53000	-3.23290
H	12.59490	-4.23910	3.73200
H	10.95030	-4.13530	4.39810

H	11.39810	-5.46510	3.31600
H	13.02300	-3.70240	1.27070
H	11.86290	-4.94810	0.80810
H	11.66390	-3.29140	0.20620
H	10.73160	-1.76650	3.53480
H	12.38310	-1.85480	2.88190
H	11.03730	-1.39960	1.82560
H	9.75550	-5.19070	-2.40710

11. N0000t_WP

N	9.71170	-6.13760	2.17170
C	9.07820	-4.95880	1.56200
C	9.11280	-4.94070	0.03210
N	9.41120	-6.09530	-0.57960
C	9.46600	-6.24230	-2.02260
C	9.86520	-7.66100	-2.38560
O	9.97620	-7.80560	-3.70720
C	10.33930	-9.12110	-4.21900
O	10.05450	-8.55580	-1.57490
O	8.84980	-3.88960	-0.59300
H	9.38230	-6.24610	3.13250
H	10.72280	-6.00450	2.23770
H	9.50340	-4.00090	1.89010
H	8.01670	-4.94140	1.84030
H	9.59190	-6.90600	0.00510
H	10.19100	-5.54680	-2.46640
H	8.49150	-6.02990	-2.48520
H	10.40790	-8.99380	-5.29940
H	11.30060	-9.43020	-3.80350
H	9.56300	-9.84550	-3.96330

12. N0010t_WP

N	9.06100	-6.27150	2.17060
C	9.14630	-4.93750	1.55770
C	9.11200	-4.93720	0.02650
N	9.41340	-6.10390	-0.57810
C	9.41010	-6.30790	-2.02390
C	8.47550	-7.48370	-2.32980
O	7.78030	-7.29640	-3.45590
C	6.89580	-8.36880	-3.89060
O	8.38140	-8.48380	-1.63200
O	8.84840	-3.88440	-0.58890
C	10.85750	-6.50410	-2.63030
C	10.76250	-6.62160	-4.16640
C	11.69550	-5.25510	-2.28520
C	11.55260	-7.75700	-2.06100
H	8.08660	-6.57650	2.21800
H	9.38600	-6.22410	3.13800
H	10.10180	-4.47610	1.83920
H	8.35750	-4.24530	1.87890
H	9.57160	-6.89650	0.03790
H	8.98320	-5.40910	-2.47560
H	7.47340	-9.27740	-4.07530
H	6.13350	-8.55410	-3.13070
H	6.44250	-8.00520	-4.81290
H	10.23550	-5.76360	-4.60020
H	11.77270	-6.64580	-4.59140
H	10.24910	-7.53470	-4.48540
H	11.24060	-4.34310	-2.68830
H	11.81400	-5.13360	-1.20390
H	12.69590	-5.35560	-2.72200
H	11.65140	-7.71020	-0.97020
H	11.01760	-8.67700	-2.31850
H	12.56340	-7.83310	-2.47830

13. N0100t_WP

N	8.53240	-6.17790	2.10520
C	9.12850	-4.92680	1.58160
C	9.11140	-4.93550	0.04340
N	9.41050	-6.09230	-0.58610
C	9.45980	-6.16970	-2.03490
C	10.60170	-5.33820	-2.62900
O	10.25740	-4.82420	-3.81780
C	11.25380	-4.03160	-4.52330
O	11.69630	-5.18480	-2.11310
O	8.85190	-3.89770	-0.59790
C	10.56780	-4.64890	2.16800
C	10.46140	-4.54810	3.70650
C	11.08370	-3.29310	1.63670
C	11.58530	-5.75020	1.80800
H	8.47430	-6.12360	3.12410
H	7.55900	-6.23370	1.79430
H	8.52270	-4.04870	1.84850
H	9.56980	-6.93140	-0.03830
H	8.51510	-5.84050	-2.47940
H	9.62960	-7.21460	-2.32120
H	11.54450	-3.17270	-3.91410
H	12.12780	-4.64450	-4.75430
H	10.76020	-3.70320	-5.43830
H	10.20690	-5.50700	4.17160
H	11.42820	-4.23740	4.11990
H	9.71330	-3.80540	4.00950
H	10.38850	-2.48010	1.87720
H	12.04890	-3.05990	2.10190
H	11.23050	-3.30260	0.55260
H	11.76470	-5.79790	0.72990
H	12.54340	-5.53500	2.29620
H	11.25230	-6.73680	2.14640

14. N0110t_WP

N	8.24670	-6.08810	2.03180
C	9.09150	-4.95740	1.57620
C	9.11130	-4.93450	0.03170
N	9.41300	-6.10240	-0.58120
C	9.30910	-6.34160	-2.02130
C	8.63610	-7.71020	-2.17780
O	7.94790	-7.82330	-3.31390
C	7.32820	-9.11230	-3.59400
O	8.73670	-8.60880	-1.35270
O	8.84950	-3.88860	-0.59110
C	10.51530	-4.96290	2.25060
C	11.33820	-6.23300	1.95070
C	11.30270	-3.72520	1.77070
C	10.32590	-4.85100	3.78080
C	10.69160	-6.26320	-2.79680
C	10.43050	-6.26330	-4.31820
C	11.39420	-4.93870	-2.43420
C	11.61850	-7.44070	-2.43240
H	7.31760	-5.99800	1.61180
H	8.08410	-5.99950	3.03690
H	8.64250	-3.98910	1.83930
H	9.48020	-6.92600	0.00780
H	8.65180	-5.57530	-2.44370
H	6.82070	-8.98100	-4.54960
H	8.09650	-9.88560	-3.66690
H	6.61470	-9.36170	-2.80560
H	12.25570	-6.22300	2.55080
H	10.78600	-7.14460	2.20210
H	11.63880	-6.28850	0.90010
H	12.27460	-3.68210	2.27630
H	11.49350	-3.75080	0.69250
H	10.76390	-2.79740	1.99600
H	11.30120	-4.71610	4.26300
H	9.69840	-3.99150	4.04610
H	9.87700	-5.75400	4.20990
H	11.38060	-6.11940	-4.84590
H	9.99290	-7.20200	-4.66960
H	9.75960	-5.44430	-4.60510
H	12.31820	-4.85260	-3.01720
H	10.76550	-4.07200	-2.66160
H	11.66650	-4.89990	-1.37600
H	11.79730	-7.50180	-1.35300
H	11.21580	-8.40360	-2.76660
H	12.58980	-7.30230	-2.92170

15. N011'0t_WP

N	9.64610	-6.29180	2.04350
C	9.04730	-5.02060	1.58500
C	9.11130	-4.93470	0.03420
N	9.41230	-6.09950	-0.58220
C	9.49620	-6.26880	-2.02580
C	10.85340	-6.90180	-2.35090
O	11.37410	-6.42150	-3.48470
C	12.63760	-6.98470	-3.94030
O	11.39880	-7.75220	-1.66090
O	8.85020	-3.89130	-0.59250
C	9.57330	-3.72790	2.31980
C	9.61550	-4.00740	3.83940
C	8.60550	-2.54440	2.10020
C	10.98820	-3.34960	1.83650
C	8.27920	-7.08790	-2.61620
C	8.42140	-7.20670	-4.14730
C	6.98400	-6.31040	-2.30050
C	8.19170	-8.49520	-1.99400
H	9.27200	-6.54950	2.95600
H	10.65830	-6.21080	2.15890
H	7.97120	-5.09210	1.80530
H	9.62460	-6.87900	0.03450
H	12.85400	-6.47350	-4.87870
H	13.41970	-6.78820	-3.20350
H	12.53070	-8.05990	-4.10120
H	9.86330	-3.08320	4.37410
H	8.64180	-4.35590	4.20680
H	10.36850	-4.75550	4.10620
H	8.94020	-1.67720	2.68300
H	8.54790	-2.25810	1.04980
H	7.59470	-2.80460	2.43950
H	10.98900	-3.06390	0.77980
H	11.36990	-2.49910	2.41360
H	11.69730	-4.17700	1.96810
H	7.51930	-7.67390	-4.55900
H	8.53530	-6.22220	-4.61620
H	9.27640	-7.82610	-4.43890
H	6.12380	-6.85140	-2.71120
H	6.82820	-6.20450	-1.22240
H	6.99930	-5.30970	-2.74660
H	7.32120	-9.02090	-2.40280
H	9.07930	-9.09690	-2.21540
H	8.06700	-8.45080	-0.90610
H	9.47230	-5.27310	-2.47470

16. N1000t_WP

N	9.66690	-6.20140	2.13480
C	9.08270	-4.98670	1.55980
C	9.11320	-4.94200	0.02530
N	9.41210	-6.09890	-0.57630
C	9.48820	-6.29380	-2.00990
C	9.96550	-7.70690	-2.30380
O	10.10430	-7.91040	-3.61490
C	10.55720	-9.22770	-4.04490
O	10.19170	-8.54960	-1.44870
O	8.84850	-3.88460	-0.58960
C	11.04930	-6.11820	2.70090
C	11.44490	-7.55060	3.10050
C	11.10570	-5.20790	3.94810
C	12.03470	-5.59940	1.63800
H	9.04620	-6.56900	2.85580
H	9.55120	-4.05640	1.90790
H	8.02390	-4.91790	1.84250
H	9.60120	-6.88510	0.04370
H	10.18850	-5.58400	-2.47100
H	8.51140	-6.14710	-2.49210
H	11.55390	-9.42500	-3.64420
H	9.85410	-9.99150	-3.70630
H	10.58120	-9.17760	-5.13330
H	10.74410	-7.95760	3.83990
H	11.44460	-8.21030	2.22590
H	12.44720	-7.56180	3.54390
H	12.12070	-5.18780	4.36180
H	10.82610	-4.17510	3.71190
H	10.42920	-5.57880	4.72730
H	13.04740	-5.59190	2.05570
H	12.03810	-6.24070	0.75100
H	11.80560	-4.57450	1.32480

17. N1010t_WP

N	9.22690	-6.34800	2.08430
C	9.15060	-4.98600	1.55440
C	9.11270	-4.93990	0.01700
N	9.41430	-6.10760	-0.57320
C	9.43360	-6.37380	-2.00350
C	8.56080	-7.61140	-2.24610
O	7.83630	-7.51550	-3.36550
C	7.00070	-8.65600	-3.71760
O	8.53120	-8.58100	-1.50240
O	8.84680	-3.87800	-0.58430
C	10.89720	-6.53840	-2.57880
C	11.65690	-5.21710	-2.33740
C	11.65430	-7.69250	-1.89200
C	10.82810	-6.80040	-4.09700
C	10.15670	-6.63680	3.21180
C	9.92500	-8.11010	3.59080
C	11.61310	-6.46230	2.74290
C	9.88750	-5.74180	4.44150
H	8.29230	-6.67330	2.33020
H	10.03020	-4.40060	1.84650
H	8.27620	-4.42360	1.90840
H	9.53500	-6.87220	0.09350
H	8.97010	-5.52260	-2.50940
H	7.62140	-9.54440	-3.85430
H	6.26110	-8.83010	-2.93280
H	6.51580	-8.37420	-4.65210
H	12.67100	-5.29880	-2.74540
H	11.16080	-4.37300	-2.83000
H	11.74520	-4.98940	-1.27030
H	12.67010	-7.75300	-2.29910
H	11.74450	-7.53450	-0.81170
H	11.17000	-8.66010	-2.05850
H	10.37090	-7.76800	-4.33060
H	10.25780	-6.01830	-4.61190
H	11.84290	-6.80650	-4.51120
H	10.58800	-8.40310	4.41250
H	8.89020	-8.27170	3.91750
H	10.12180	-8.76500	2.73480
H	12.29970	-6.72690	3.55490
H	11.82450	-7.11480	1.88860
H	11.83670	-5.42950	2.45220
H	8.85310	-5.85980	4.78570
H	10.55490	-6.01200	5.26830
H	10.05460	-4.68290	4.21360

18. N1100t_WP

N	9.79610	-3.85600	2.19380
C	9.09780	-4.98780	1.58300
C	9.11120	-4.93450	0.03930
N	9.41160	-6.09670	-0.58450
C	9.36040	-6.26170	-2.02670
C	9.35450	-7.73960	-2.37750
O	9.11850	-7.92690	-3.67710
C	9.09290	-9.29900	-4.16830
O	9.55120	-8.64070	-1.57560
O	8.85100	-3.89430	-0.59530
C	7.62620	-5.11810	2.15130
C	6.85130	-3.79150	2.03180
C	7.73490	-5.50500	3.64070
C	6.85260	-6.22890	1.41200
C	11.25850	-3.93910	2.44710
C	12.09330	-4.16270	1.16520
C	11.65180	-2.59010	3.07700
C	11.55520	-5.06180	3.45970
H	9.59330	-3.00720	1.66710
H	9.60860	-5.90990	1.87990
H	9.65620	-6.91590	-0.03630
H	10.22350	-5.79610	-2.52710
H	8.46010	-5.78960	-2.43740
H	10.06650	-9.76860	-4.01180
H	8.31140	-9.86220	-3.65350
H	8.87070	-9.21380	-5.23210
H	5.84580	-3.91330	2.45230
H	7.35070	-2.99180	2.58780
H	6.74610	-3.47590	0.98900
H	6.73570	-5.56500	4.08860
H	8.21610	-6.48380	3.76250
H	8.31800	-4.76380	4.19420
H	7.39400	-7.18250	1.43790
H	5.87970	-6.38540	1.89310
H	6.66390	-5.97350	0.36320
H	13.16500	-4.16690	1.39650
H	11.85320	-5.12100	0.69220
H	11.90540	-3.36530	0.43700
H	12.72530	-2.56560	3.29470
H	11.42610	-1.75960	2.39620
H	11.10180	-2.42760	4.01010
H	10.96660	-4.92380	4.37270
H	11.33850	-6.05750	3.05670
H	12.61750	-5.04650	3.72800

19. N1110t_WP

N	8.69120	-6.29570	2.06200
C	9.12040	-4.97410	1.57860
C	9.11150	-4.93540	0.02760
N	9.41370	-6.10490	-0.57910
C	9.34970	-6.33590	-2.01840
C	8.40600	-7.51840	-2.26770
O	7.64190	-7.33510	-3.35030
C	6.75150	-8.42070	-3.73740
O	8.35950	-8.51820	-1.56560
O	8.84860	-3.88510	-0.58900
C	10.50720	-4.54170	2.20200
C	10.99190	-3.21850	1.57320
C	11.59690	-5.61720	2.00330
C	10.30840	-4.29640	3.71640
C	10.77110	-6.55440	-2.67250
C	11.58250	-5.25530	-2.48950
C	11.52450	-7.72750	-2.01460
C	10.61490	-6.82720	-4.18280
C	7.24450	-6.49530	2.38330
C	6.37740	-6.20270	1.14650
C	7.09440	-7.97620	2.77130
C	6.77780	-5.61240	3.56350
H	9.22780	-6.56420	2.88360
H	8.41130	-4.18360	1.86090
H	9.44930	-6.90960	0.04410
H	8.90930	-5.44560	-2.47450
H	6.25090	-8.06900	-4.63980
H	7.33180	-9.32310	-3.94340
H	6.02750	-8.61120	-2.94200
H	11.89970	-2.88420	2.08860
H	11.23280	-3.32620	0.51160
H	10.23780	-2.42940	1.66970
H	12.53610	-5.27040	2.44920
H	11.34130	-6.56830	2.48300
H	11.78400	-5.81370	0.94310
H	9.99980	-5.19870	4.25560
H	11.25280	-3.96170	4.16050
H	9.55730	-3.51840	3.89690
H	12.57530	-5.37670	-2.93750
H	11.09170	-4.40540	-2.97770
H	11.72190	-5.01010	-1.43260
H	12.51840	-7.82070	-2.46750
H	11.66490	-7.57030	-0.93970
H	11.00210	-8.67920	-2.15600
H	10.12160	-7.78450	-4.38400
H	10.04200	-6.03470	-4.67860
H	11.60700	-6.86480	-4.64710
H	5.32760	-6.41550	1.37510

H	6.43770	-5.15270	0.83980
H	6.67520	-6.83040	0.30140
H	6.05560	-8.19940	3.03880
H	7.38620	-8.62490	1.93850
H	7.72500	-8.22010	3.63570
H	6.87750	-4.54430	3.34090
H	5.72200	-5.80340	3.78740
H	7.35960	-5.83010	4.46680

20. N111'0t_WP

N	9.53120	-3.66510	2.12850
C	9.12190	-4.96190	1.58780
C	9.11060	-4.93180	0.03540
N	9.41300	-6.10230	-0.58360
C	9.59130	-6.25190	-2.02890
C	10.83460	-7.12620	-2.22660
O	11.51180	-6.81680	-3.33330
C	12.67970	-7.62750	-3.65700
O	11.16430	-8.01910	-1.45660
O	8.85030	-3.89140	-0.59240
C	7.74440	-5.42570	2.21680
C	7.33420	-6.81890	1.69840
C	6.62290	-4.41320	1.91540
C	7.93570	-5.53020	3.74410
C	8.32170	-6.82880	-2.78010
C	8.57450	-6.83880	-4.30280
C	7.98100	-8.25420	-2.30260
C	7.12310	-5.89730	-2.50350
C	10.97350	-3.40390	2.37910
C	11.47820	-4.30670	3.52080
C	11.86370	-3.60420	1.13010
C	11.05940	-1.93450	2.82930
H	9.16700	-2.93250	1.52070
H	9.85420	-5.71980	1.88970
H	9.70760	-6.88660	-0.00970
H	13.42160	-7.54550	-2.85980
H	12.38270	-8.67010	-3.79160
H	13.06420	-7.21210	-4.58850
H	6.44990	-7.16750	2.24440
H	7.08080	-6.81340	0.63500
H	8.13100	-7.55740	1.85600
H	5.68980	-4.74050	2.38960
H	6.86880	-3.42220	2.31090
H	6.43590	-4.31830	0.84020
H	8.24760	-4.57290	4.16790
H	6.99360	-5.83360	4.21620
H	8.69340	-6.28270	3.99630
H	7.65040	-7.12470	-4.81860
H	8.86710	-5.84610	-4.66550
H	9.35140	-7.55180	-4.59520
H	7.05720	-8.58990	-2.78780
H	8.76910	-8.97170	-2.55560
H	7.81910	-8.29370	-1.22020
H	6.87260	-5.85560	-1.44050
H	7.32150	-4.87660	-2.84610
H	6.24360	-6.27320	-3.03880
H	12.51680	-4.05490	3.76350
H	10.86850	-4.16890	4.41970

H	11.45700	-5.36830	3.24960
H	12.90760	-3.35780	1.35720
H	11.84000	-4.64320	0.78210
H	11.53130	-2.96070	0.30790
H	10.45760	-1.77390	3.73030
H	12.09670	-1.65800	3.04770
H	10.68920	-1.26290	2.04450
H	9.79380	-5.26040	-2.44380

21. N0000c_GP

N	8.77670	-3.67320	2.20740
C	9.10150	-4.94150	1.58020
C	9.10840	-4.92330	0.04070
N	9.41220	-6.09880	-0.58820
C	9.72780	-7.36360	0.03740
C	10.00410	-8.40460	-1.03560
O	10.30450	-9.58920	-0.48210
C	10.58810	-10.66740	-1.40260
O	9.95870	-8.19350	-2.22940
O	8.85330	-3.90340	-0.59300
H	7.86740	-3.35530	1.87970
H	9.43440	-2.96200	1.89620
H	8.38710	-5.70190	1.92270
H	10.08620	-5.27310	1.93570
H	9.41470	-6.08420	-1.60340
H	8.90770	-7.74130	0.66310
H	10.61580	-7.30590	0.68140
H	10.80620	-11.52980	-0.77470
H	9.72050	-10.85810	-2.03790
H	11.44730	-10.41330	-2.02690

22. N0010c_GP

N	8.68150	-3.64670	2.17850
C	9.08490	-4.91160	1.58870
C	9.10800	-4.92200	0.04710
N	9.41160	-6.09660	-0.59140
C	9.84710	-7.36210	-0.01430
C	11.27340	-7.63600	-0.50400
O	12.08770	-8.00350	0.50420
C	13.45530	-8.29470	0.13990
O	11.63430	-7.52880	-1.65870
O	8.85420	-3.90690	-0.59620
C	8.86650	-8.55330	-0.33200
C	7.52750	-8.26750	0.37540
C	8.62210	-8.69800	-1.84700
C	9.46120	-9.86290	0.22380
H	7.73940	-3.41340	1.87350
H	9.27010	-2.90080	1.81440
H	8.41350	-5.69950	1.94890
H	10.08670	-5.16860	1.95690
H	9.50720	-6.00230	-1.59690
H	9.89060	-7.25830	1.07010
H	13.48990	-9.13430	-0.55820
H	13.91610	-7.42010	-0.32410
H	13.95440	-8.54770	1.07430
H	6.80860	-9.06370	0.15430
H	7.64920	-8.22490	1.46440
H	7.09110	-7.32230	0.03850
H	8.12870	-7.81270	-2.26150
H	9.55180	-8.86140	-2.39910
H	7.96240	-9.55290	-2.03140
H	8.73770	-10.67690	0.10970
H	10.37200	-10.15900	-0.30750
H	9.70470	-9.77750	1.28880

23. N0100c_GP

N	9.90610	-3.73430	2.06120
C	9.12390	-4.88510	1.59270
C	9.10850	-4.92400	0.05040
N	9.41080	-6.09310	-0.59230
C	9.80960	-7.36480	-0.02830
C	10.07260	-8.35270	-1.15430
O	10.43070	-9.54870	-0.66120
C	10.71200	-10.57910	-1.63530
O	9.97530	-8.09710	-2.33630
O	8.85460	-3.90840	-0.59860
C	7.67820	-4.86820	2.21130
C	6.95440	-3.53890	1.91730
C	7.81640	-5.04420	3.73680
C	6.84320	-6.03470	1.64580
H	10.89970	-3.90160	1.92510
H	9.66850	-2.92240	1.49380
H	9.61620	-5.78520	1.97790
H	9.40430	-6.04450	-1.60710
H	9.04000	-7.79920	0.62040
H	10.72930	-7.29390	0.56790
H	10.98070	-11.45850	-1.05210
H	9.82700	-10.77240	-2.24520
H	11.53850	-10.27260	-2.28000
H	7.48420	-2.69230	2.36500
H	6.86000	-3.35590	0.84380
H	5.94930	-3.56410	2.35380
H	6.83070	-5.00460	4.21430
H	8.26890	-6.01230	3.98690
H	8.44250	-4.25630	4.16240
H	7.31030	-7.00510	1.85570
H	5.85380	-6.04810	2.11570
H	6.69120	-5.94970	0.56460

24. N0110c_GP

N	9.90870	-3.71290	2.04740
C	9.11080	-4.86210	1.59920
C	9.10770	-4.92120	0.05700
N	9.41070	-6.09270	-0.59600
C	9.92220	-7.34740	-0.06170
C	11.44310	-7.25460	0.13880
O	11.80590	-7.69730	1.36230
C	13.22100	-7.67770	1.64960
O	12.23030	-6.83090	-0.68180
O	8.85540	-3.91150	-0.60150
C	7.66500	-4.81310	2.21370
C	6.95960	-3.47840	1.90140
C	6.81770	-5.97610	1.66230
C	7.80000	-4.97020	3.74150
C	9.52390	-8.57010	-0.96900
C	10.15110	-9.85120	-0.38080
C	7.98860	-8.70900	-0.94980
C	10.00590	-8.39520	-2.42470
H	9.68480	-2.90810	1.46480
H	10.89990	-3.89810	1.91660
H	9.59160	-5.75700	2.00570
H	9.53390	-5.94380	-1.59120
H	9.47280	-7.52380	0.91630
H	13.31310	-8.03880	2.67310
H	13.75640	-8.33230	0.95780
H	13.61220	-6.66210	1.55830
H	7.50130	-2.63300	2.33700
H	6.86760	-3.30910	0.82540
H	5.95430	-3.48350	2.33850
H	5.83360	-5.98360	2.14330
H	6.65600	-5.89270	0.58270
H	7.28460	-6.94790	1.86430
H	6.81480	-4.90800	4.21800
H	8.23710	-5.94170	4.00490
H	8.43870	-4.18660	4.15620
H	9.78940	-10.72570	-0.93140
H	11.24370	-9.84720	-0.45560
H	9.88490	-9.98440	0.67350
H	7.68240	-9.54480	-1.58820
H	7.61770	-8.90750	0.06190
H	7.49680	-7.80440	-1.31820
H	9.49610	-7.57010	-2.93460
H	11.08310	-8.21840	-2.48270
H	9.77680	-9.30300	-2.99310

25. N011'0c_GP

N	9.88050	-3.69150	2.05230
C	9.11180	-4.85600	1.59700
C	9.10820	-4.92300	0.05630
N	9.41020	-6.09090	-0.59520
C	9.76820	-7.40990	-0.09100
C	8.82550	-8.42310	-0.74860
O	8.36860	-9.33070	0.13460
C	7.48270	-10.34110	-0.39630
O	8.52350	-8.41370	-1.92490
O	8.85540	-3.91160	-0.60160
C	7.65670	-4.83530	2.19560
C	6.94170	-3.50280	1.89550
C	7.77790	-5.01320	3.72220
C	6.82540	-5.99850	1.61780
C	11.28620	-7.77130	-0.32580
C	11.54920	-9.21540	0.14700
C	12.14300	-6.80580	0.51650
C	11.67640	-7.63320	-1.81050
H	9.65480	-2.89470	1.45950
H	10.87730	-3.86170	1.94560
H	9.60530	-5.74010	2.01040
H	9.32000	-6.01580	-1.60340
H	6.59490	-9.87390	-0.82770
H	7.99270	-10.92630	-1.16500
H	7.21760	-10.96700	0.45470
H	5.92940	-3.52660	2.31520
H	7.46730	-2.65990	2.35500
H	6.86530	-3.31610	0.82110
H	8.41140	-4.23370	4.15300
H	6.78860	-4.95880	4.19080
H	8.21320	-5.98820	3.97650
H	7.30080	-6.97030	1.79850
H	5.84080	-6.02600	2.09750
H	6.66110	-5.89130	0.54020
H	11.03430	-9.95310	-0.47700
H	12.62100	-9.43330	0.09350
H	11.22670	-9.36810	1.18310
H	11.93130	-6.90850	1.58750
H	13.20640	-7.02170	0.36750
H	11.97370	-5.76420	0.22670
H	11.56780	-6.60180	-2.16120
H	12.72740	-7.91270	-1.94120
H	11.07210	-8.27760	-2.45540
H	9.58400	-7.44690	0.98220

26. N1000c_GP

N	8.41510	-3.78630	2.08140
C	9.14510	-4.92900	1.57160
C	9.10860	-4.92440	0.04160
N	9.41170	-6.09670	-0.58830
C	9.72690	-7.36720	0.02610
C	10.05690	-8.37930	-1.05900
O	10.38740	-9.56360	-0.52180
C	10.71180	-10.61770	-1.45670
O	10.01990	-8.14850	-2.24930
O	8.85350	-3.90430	-0.59380
C	8.80040	-3.22540	3.39170
C	8.64350	-4.31080	4.47170
C	7.81390	-2.07910	3.67240
C	10.24440	-2.66810	3.40850
H	8.41140	-3.06640	1.36050
H	8.71800	-5.86550	1.95310
H	10.21790	-4.94440	1.84800
H	9.41720	-6.07290	-1.60290
H	8.89010	-7.77100	0.61230
H	10.58940	-7.30420	0.70250
H	11.56960	-10.32840	-2.06760
H	10.94910	-11.48370	-0.84050
H	9.85660	-10.82370	-2.10380
H	9.35290	-5.13370	4.32780
H	8.82670	-3.89180	5.46670
H	7.62990	-4.72450	4.45070
H	6.78630	-2.45450	3.68070
H	8.02610	-1.60830	4.63820
H	7.88550	-1.30480	2.89920
H	10.36440	-1.88440	2.65190
H	10.48630	-2.23540	4.38590
H	10.98750	-3.44770	3.20720

27. N1010c_GP

N	8.30710	-3.79260	2.04680
C	9.12110	-4.89920	1.57920
C	9.10830	-4.92310	0.04810
N	9.41110	-6.09460	-0.59150
C	9.85880	-7.36600	-0.03670
C	11.21040	-7.69380	-0.67910
O	12.10460	-8.13490	0.22540
C	13.40460	-8.49440	-0.29310
O	11.45230	-7.57030	-1.86310
O	8.85450	-3.90780	-0.59710
C	8.81120	-8.52930	-0.23050
C	8.39550	-8.66900	-1.70820
C	9.42000	-9.85680	0.26420
C	7.56790	-8.20010	0.61850
C	8.58880	-3.22500	3.38090
C	10.00330	-2.60610	3.48740
C	7.53760	-2.12500	3.60720
C	8.41520	-4.32360	4.44520
H	8.31840	-3.06750	1.33150
H	8.74330	-5.84410	1.98230
H	10.18790	-4.83530	1.87080
H	9.49050	-5.99630	-1.59800
H	10.03030	-7.24900	1.03400
H	13.86940	-7.63200	-0.77550
H	13.98180	-8.81330	0.57370
H	13.31140	-9.30640	-1.01790
H	7.69170	-9.50180	-1.81320
H	7.89030	-7.76750	-2.06940
H	9.25180	-8.86550	-2.35970
H	9.78890	-9.77590	1.29300
H	10.25140	-10.18980	-0.36600
H	8.65830	-10.64320	0.24230
H	7.80780	-8.16660	1.68780
H	7.12720	-7.24020	0.33370
H	6.80410	-8.97210	0.47590
H	10.16600	-2.16980	4.47950
H	10.79030	-3.35150	3.32840
H	10.13440	-1.81240	2.74310
H	7.62040	-1.34330	2.84250
H	6.52890	-2.54530	3.55300
H	7.67160	-1.65160	4.58560
H	9.17070	-5.11120	4.34500
H	8.51460	-3.90210	5.45100
H	7.42560	-4.78400	4.35830

28. N1100c_GP

N	9.72760	-3.65990	2.08210
C	9.08580	-4.88360	1.59620
C	9.10840	-4.92400	0.05160
N	9.41060	-6.09230	-0.59290
C	9.85570	-7.34990	-0.03380
C	10.17760	-8.31600	-1.16310
O	10.55940	-9.50640	-0.67370
C	10.89590	-10.51670	-1.65110
O	10.10170	-8.05080	-2.34470
O	8.85480	-3.90930	-0.59930
C	7.61260	-4.99400	2.16190
C	7.70290	-5.12690	3.69470
C	6.90200	-6.24250	1.60110
C	6.78270	-3.74360	1.81080
C	11.19620	-3.63020	2.29350
C	11.53230	-2.17230	2.65650
C	11.56310	-4.53630	3.48280
C	12.01690	-4.03920	1.04810
H	9.47160	-2.90950	1.44300
H	9.63250	-5.74380	1.99450
H	9.42190	-6.03500	-1.60720
H	9.09680	-7.82250	0.60000
H	10.76270	-7.24400	0.57700
H	11.17640	-11.39430	-1.07060
H	10.03240	-10.72950	-2.28490
H	11.72830	-10.17830	-2.27170
H	6.69820	-5.17980	4.12980
H	8.23980	-6.03850	3.98670
H	8.22410	-4.26930	4.12660
H	5.89460	-6.31750	2.02460
H	6.79690	-6.20910	0.51170
H	7.43060	-7.16480	1.87230
H	6.70000	-3.59910	0.73020
H	5.77220	-3.84570	2.22320
H	7.22740	-2.84270	2.24390
H	12.60050	-2.06070	2.86950
H	11.28390	-1.49600	1.82990
H	10.96500	-1.85730	3.53770
H	12.63240	-4.45600	3.70590
H	10.99880	-4.24470	4.37370
H	11.35350	-5.59220	3.27930
H	11.75660	-3.41740	0.18540
H	13.09090	-3.92980	1.23780
H	11.84160	-5.08480	0.77220

29. N1110c_GP

N	9.70440	-3.58720	2.05170
C	9.09490	-4.84370	1.60740
C	9.10770	-4.92110	0.06050
N	9.41010	-6.09040	-0.59760
C	9.82200	-7.39290	-0.09420
C	11.34750	-7.44790	0.08140
O	11.68450	-7.94760	1.29050
C	13.09900	-8.09370	1.54310
O	12.15980	-7.09740	-0.74880
O	8.85610	-3.91400	-0.60340
C	7.63330	-4.95760	2.20400
C	7.75470	-5.03240	3.73870
C	6.92940	-6.23290	1.70600
C	6.77590	-3.73460	1.82230
C	9.32120	-8.56420	-1.02590
C	9.85490	-9.90310	-0.47430
C	7.78080	-8.59770	-1.00240
C	9.80270	-8.39270	-2.48240
C	11.17160	-3.47000	2.24220
C	11.63520	-4.43930	3.34400
C	11.98690	-3.70330	0.95000
C	11.40400	-2.02520	2.72280
H	9.40240	-2.85980	1.40650
H	9.66870	-5.67680	2.02270
H	9.50680	-5.94050	-1.59520
H	9.37400	-7.55090	0.88720
H	13.17190	-8.49660	2.55250
H	13.54270	-8.78070	0.81860
H	13.59800	-7.12500	1.47370
H	8.31140	-5.92470	4.05220
H	8.27060	-4.15210	4.12880
H	6.75900	-5.08480	4.19410
H	5.93620	-6.31120	2.16190
H	6.79200	-6.23360	0.62090
H	7.48230	-7.13740	1.98620
H	6.67590	-3.62910	0.73840
H	5.77260	-3.84160	2.25080
H	7.20760	-2.81080	2.21800
H	9.41990	-10.73340	-1.04010
H	10.94340	-9.98310	-0.56330
H	9.59040	-10.04040	0.57990
H	7.41910	-9.39340	-1.66260
H	7.39790	-8.79810	0.00360
H	7.35040	-7.65380	-1.34810
H	9.35110	-7.52100	-2.96930
H	10.88920	-8.29590	-2.54910
H	9.49980	-9.26800	-3.06720
H	12.69140	-4.26500	3.57580

H	11.05230	-4.28900	4.25810
H	11.53750	-5.48810	3.04420
H	13.05590	-3.53740	1.12870
H	11.87570	-4.72210	0.56850
H	11.66520	-3.01830	0.15860
H	10.85410	-1.83520	3.64950
H	12.46880	-1.84110	2.90070
H	11.06270	-1.30420	1.97020

30. N111'0c_GP

N	9.66880	-3.54870	2.04850
C	9.09650	-4.82330	1.60830
C	9.10750	-4.92050	0.06270
N	9.41000	-6.08990	-0.59870
C	9.64000	-7.45090	-0.13480
C	8.40360	-8.30630	-0.45090
O	8.16030	-9.21620	0.51300
C	7.03360	-10.09120	0.28980
O	7.73410	-8.19550	-1.45720
O	8.85640	-3.91520	-0.60450
C	7.62680	-4.95560	2.19070
C	7.73780	-5.04070	3.72550
C	6.93200	-6.23040	1.67910
C	6.76620	-3.73390	1.81270
C	10.93890	-8.09040	-0.77260
C	12.15880	-7.25750	-0.34030
C	11.11680	-9.53260	-0.25310
C	10.86400	-8.11080	-2.31330
C	11.11870	-3.39280	2.31450
C	11.99880	-3.60200	1.06280
C	11.28960	-1.94420	2.80970
C	11.55850	-4.35400	3.43310
H	9.38060	-2.83920	1.37790
H	9.68060	-5.64200	2.03830
H	9.24870	-5.99610	-1.59610
H	6.11470	-9.50730	0.20400
H	7.17880	-10.67200	-0.62410
H	6.99830	-10.74380	1.16130
H	6.73930	-5.09920	4.17400
H	8.29490	-5.93350	4.03730
H	8.24690	-4.16060	4.12500
H	5.93920	-6.31610	2.13540
H	6.78740	-6.21730	0.59380
H	7.48910	-7.13350	1.94970
H	6.67700	-3.61820	0.72890
H	5.75900	-3.85340	2.22820
H	7.18740	-2.81210	2.22330
H	13.07510	-7.70580	-0.73920
H	12.09630	-6.23380	-0.71620
H	12.25590	-7.21980	0.75110
H	12.07600	-9.92980	-0.60130
H	11.11510	-9.57160	0.84180
H	10.33280	-10.20590	-0.61170
H	9.99720	-8.66840	-2.67890
H	10.82150	-7.10090	-2.73490
H	11.76540	-8.58650	-2.71470
H	13.05650	-3.42560	1.29130
H	11.90940	-4.62210	0.68210

H	11.70310	-2.91750	0.26060
H	12.34070	-1.73040	3.03130
H	10.95770	-1.22980	2.04690
H	10.69750	-1.77450	3.71400
H	11.51700	-5.40340	3.12130
H	12.59440	-4.14550	3.72050
H	10.92400	-4.23400	4.31670
H	9.77720	-7.44210	0.94760

31. N0000c_WP

N	8.78130	-3.64290	2.18740
C	9.10240	-4.93770	1.58260
C	9.11140	-4.93560	0.04870
N	9.40950	-6.08840	-0.58840
C	9.73410	-7.38210	-0.00290
C	10.00370	-8.37130	-1.12940
O	10.31560	-9.57920	-0.66020
C	10.59990	-10.61880	-1.64280
O	9.94110	-8.08910	-2.31560
O	8.85290	-3.90150	-0.60090
H	7.86900	-3.32530	1.85120
H	9.44540	-2.93840	1.85790
H	8.38100	-5.69050	1.92850
H	10.08650	-5.27150	1.93850
H	9.40500	-6.04590	-1.60610
H	8.91330	-7.77960	0.61100
H	10.62940	-7.33760	0.63330
H	10.82190	-11.51100	-1.05750
H	9.72490	-10.77800	-2.27670
H	11.46000	-10.32820	-2.24990

32. N0010c_WP

N	8.42280	-3.72550	2.15140
C	9.11280	-4.89130	1.59100
C	9.11100	-4.93410	0.05390
N	9.40910	-6.08650	-0.59110
C	9.77240	-7.40220	-0.05900
C	11.04750	-7.84810	-0.78990
O	11.87260	-8.53260	0.00460
C	13.07300	-9.09800	-0.59810
O	11.26960	-7.61800	-1.96990
O	8.85380	-3.90490	-0.60340
C	8.60420	-8.46760	-0.20020
C	7.50420	-8.12050	0.82450
C	8.00380	-8.44480	-1.61950
C	9.12920	-9.88610	0.10810
H	7.43720	-3.74920	1.87770
H	8.79670	-2.87270	1.72920
H	8.66470	-5.79700	2.00890
H	10.16230	-4.88740	1.91850
H	9.44670	-5.99210	-1.60510
H	10.01940	-7.31240	1.00090
H	12.79980	-9.74630	-1.43390
H	13.72950	-8.29460	-0.94020
H	13.54680	-9.67250	0.19800
H	6.67710	-8.83340	0.72780
H	7.88010	-8.18510	1.85310
H	7.09300	-7.11830	0.66280
H	7.54090	-7.47860	-1.84670
H	8.75630	-8.65890	-2.38610
H	7.22340	-9.21060	-1.69320
H	8.28450	-10.58410	0.12310
H	9.83280	-10.24160	-0.65290
H	9.62450	-9.93520	1.08450

33. N0100c_WP

N	9.97910	-3.78860	2.06500
C	9.12650	-4.90200	1.59660
C	9.11120	-4.93500	0.05410
N	9.40910	-6.08650	-0.59090
C	9.80510	-7.37520	-0.03590
C	10.11700	-8.33550	-1.17500
O	10.42980	-9.54560	-0.71070
C	10.76500	-10.57520	-1.68770
O	10.08430	-8.03620	-2.35830
O	8.85350	-3.90400	-0.60370
C	7.67380	-4.85650	2.21430
C	6.99550	-3.48590	2.01380
C	7.77590	-5.14890	3.72660
C	6.78820	-5.94860	1.57370
H	10.94730	-3.96920	1.78790
H	9.71260	-2.92840	1.58180
H	9.58960	-5.82320	1.96680
H	9.40190	-6.02760	-1.60820
H	9.01500	-7.83080	0.57340
H	10.70360	-7.29900	0.59200
H	10.96100	-11.47150	-1.09920
H	9.92320	-10.73200	-2.36550
H	11.65410	-10.27730	-2.24780
H	7.55130	-2.68220	2.50870
H	6.89290	-3.23310	0.95440
H	5.99170	-3.51000	2.45520
H	6.77770	-5.12450	4.18030
H	8.20210	-6.14280	3.90920
H	8.39870	-4.40910	4.23760
H	7.21480	-6.94950	1.71150
H	5.80120	-5.94950	2.05080
H	6.63300	-5.78290	0.50160

34. N0110c_WP

N	9.99280	-3.73140	2.02170
C	9.15770	-4.87600	1.60100
C	9.11040	-4.93190	0.05660
N	9.40930	-6.08750	-0.59290
C	9.71550	-7.40960	-0.04710
C	11.18350	-7.49000	0.41110
O	11.29940	-8.23650	1.51640
C	12.63360	-8.47170	2.04820
O	12.12320	-6.94460	-0.14580
O	8.85410	-3.90610	-0.60420
C	7.73400	-4.85630	2.27810
C	7.00480	-3.51580	2.05790
C	6.85490	-5.99680	1.71870
C	7.91610	-5.09200	3.79300
C	9.33890	-8.54540	-1.07850
C	9.63590	-9.92680	-0.45580
C	7.82320	-8.45770	-1.36400
C	10.12710	-8.40860	-2.39790
H	9.66120	-2.88250	1.55910
H	10.94920	-3.86270	1.68300
H	9.65770	-5.77420	1.97610
H	9.45760	-5.97390	-1.60290
H	9.09890	-7.58530	0.83580
H	12.48460	-9.12180	2.91100
H	13.25440	-8.96430	1.29610
H	13.08630	-7.52500	2.35210
H	7.55750	-2.67450	2.48990
H	6.84030	-3.31260	0.99540
H	6.02610	-3.55170	2.55140
H	5.87990	-5.98620	2.21980
H	6.67080	-5.89480	0.64330
H	7.29800	-6.98230	1.90590
H	6.93870	-5.09350	4.29000
H	8.39110	-6.06120	3.98850
H	8.52950	-4.31150	4.25190
H	9.26830	-10.70800	-1.13050
H	10.70740	-10.09670	-0.30770
H	9.13170	-10.05150	0.50970
H	7.54190	-9.24940	-2.06740
H	7.23640	-8.59990	-0.44880
H	7.53810	-7.49910	-1.80870
H	9.91200	-7.47280	-2.92820
H	11.20790	-8.46880	-2.23390
H	9.84320	-9.22660	-3.06970

35. N011'0c_WP

N	9.94040	-3.66900	2.01030
C	9.15890	-4.85620	1.60430
C	9.11060	-4.93260	0.05970
N	9.40880	-6.08540	-0.59410
C	9.65330	-7.44710	-0.10400
C	8.82700	-8.38140	-1.00360
O	8.28260	-9.39250	-0.32560
C	7.53310	-10.38470	-1.08700
O	8.69440	-8.21590	-2.20760
O	8.85440	-3.90750	-0.60610
C	7.73920	-4.88930	2.28800
C	6.95090	-3.58570	2.05540
C	7.94270	-5.09360	3.80520
C	6.91000	-6.07700	1.75240
C	11.18140	-7.85980	-0.06810
C	11.30410	-9.34900	0.32230
C	11.89650	-7.01820	1.00880
C	11.86170	-7.62080	-1.42900
H	9.60050	-2.85170	1.49930
H	10.91400	-3.78040	1.71730
H	9.69760	-5.72630	1.98490
H	9.37850	-5.99100	-1.60870
H	6.68210	-9.90980	-1.58030
H	8.18560	-10.85440	-1.82690
H	7.19680	-11.11380	-0.34980
H	5.98260	-3.65100	2.56600
H	7.47710	-2.71460	2.46060
H	6.76090	-3.41070	0.99220
H	8.51510	-4.27390	4.24820
H	6.97030	-5.14250	4.30940
H	8.47270	-6.03180	4.01110
H	7.40040	-7.03730	1.95050
H	5.93790	-6.10440	2.25800
H	6.71690	-6.00020	0.67640
H	10.92230	-10.01650	-0.45770
H	12.36070	-9.59510	0.47740
H	10.76810	-9.56890	1.25320
H	11.47070	-7.18920	2.00490
H	12.95410	-7.30190	1.04940
H	11.85570	-5.94720	0.78500
H	11.82220	-6.56510	-1.71920
H	12.91750	-7.90600	-1.35950
H	11.40550	-8.21690	-2.22590
H	9.26450	-7.54680	0.90970

36. N1000c_WP

N	8.66480	-3.69770	2.13700
C	9.14120	-4.95960	1.57110
C	9.11110	-4.93440	0.04630
N	9.41040	-6.09170	-0.58760
C	9.74580	-7.36410	0.03540
C	10.31580	-8.31360	-1.00690
O	10.66540	-9.47530	-0.45210
C	11.22860	-10.50080	-1.32200
O	10.43200	-8.04780	-2.19280
O	8.85230	-3.89950	-0.59920
C	8.78950	-3.55020	3.62000
C	7.84020	-4.54160	4.31750
C	8.35220	-2.11140	3.94570
C	10.23270	-3.76470	4.13180
H	9.18570	-2.93710	1.69560
H	8.51080	-5.78750	1.91830
H	10.17620	-5.21460	1.86000
H	9.42370	-6.05810	-1.60550
H	8.86940	-7.84880	0.49070
H	10.49840	-7.24250	0.82500
H	12.15110	-10.13440	-1.77730
H	11.43150	-11.34740	-0.66580
H	10.50380	-10.77250	-2.09240
H	8.12870	-5.58270	4.13610
H	7.86270	-4.38000	5.40080
H	6.80910	-4.40260	3.97330
H	7.32400	-1.93080	3.61260
H	8.40060	-1.93270	5.02540
H	9.00770	-1.38110	3.45460
H	10.93350	-3.09330	3.62080
H	10.28630	-3.55390	5.20610
H	10.57300	-4.79540	3.98470

37. N1010c_WP

N	8.34370	-3.80580	2.11590
C	9.13900	-4.91290	1.58020
C	9.11070	-4.93270	0.05190
N	9.41000	-6.09000	-0.59060
C	9.77550	-7.39150	-0.02500
C	11.07320	-7.84800	-0.70630
O	11.88420	-8.48530	0.14200
C	13.11240	-9.05650	-0.39380
O	11.32680	-7.66490	-1.88740
O	8.85320	-3.90280	-0.60180
C	8.62190	-8.47140	-0.14840
C	8.14160	-8.61430	-1.60570
C	9.12050	-9.83840	0.36770
C	7.43900	-8.02320	0.73530
C	8.08650	-3.84350	3.58990
C	9.37150	-4.04930	4.42310
C	7.45890	-2.48640	3.95200
C	7.07720	-4.96380	3.90750
H	8.82360	-2.93170	1.89480
H	8.74710	-5.85050	1.97580
H	10.20510	-4.87770	1.86790
H	9.44620	-6.00780	-1.60550
H	10.00040	-7.26960	1.03570
H	13.75100	-8.26330	-0.78870
H	13.58790	-9.55070	0.45370
H	12.87450	-9.77830	-1.17860
H	7.34170	-9.36200	-1.65030
H	7.73430	-7.67380	-1.99320
H	8.94520	-8.94540	-2.27140
H	9.53210	-9.76300	1.38110
H	9.88610	-10.27580	-0.28180
H	8.27780	-10.53860	0.40000
H	7.72080	-7.99000	1.79460
H	7.05410	-7.04120	0.44130
H	6.61810	-8.74250	0.63610
H	9.14290	-3.98990	5.49340
H	9.82280	-5.03020	4.23730
H	10.11620	-3.27790	4.19250
H	8.15240	-1.66290	3.74080
H	6.53780	-2.31820	3.38260
H	7.21330	-2.45380	5.01890
H	7.48320	-5.95970	3.69920
H	6.81390	-4.93810	4.97080
H	6.15910	-4.83540	3.32370

38. N1100c_WP

N	9.68940	-3.57380	2.05240
C	9.10980	-4.84770	1.61430
C	9.11020	-4.93130	0.06300
N	9.40850	-6.08420	-0.59590
C	9.75240	-7.40060	-0.09000
C	8.68810	-8.45180	-0.41760
O	8.84670	-9.54320	0.34020
C	7.93470	-10.65870	0.11780
O	7.82740	-8.33010	-1.27280
O	8.85510	-3.91000	-0.60760
C	7.66550	-5.04250	2.23980
C	7.81690	-5.07790	3.77470
C	7.04050	-6.37590	1.78760
C	6.72730	-3.88140	1.85970
C	11.12420	-3.49860	2.43400
C	11.41250	-2.00720	2.68640
C	11.36240	-4.27500	3.74380
C	12.08140	-4.02440	1.33970
H	9.48410	-2.86090	1.35470
H	9.72140	-5.66070	2.01540
H	9.40740	-5.98470	-1.61060
H	9.91760	-7.38470	0.98790
H	10.69000	-7.74530	-0.55070
H	8.23900	-11.42150	0.83460
H	6.90690	-10.34100	0.30660
H	8.03620	-11.02610	-0.90570
H	6.83520	-5.21600	4.24330
H	8.45890	-5.90890	4.09240
H	8.25230	-4.14770	4.14730
H	6.06580	-6.51130	2.27020
H	6.87600	-6.41220	0.70590
H	7.66460	-7.22960	2.07960
H	6.54120	-3.84240	0.78160
H	5.76140	-4.00670	2.36430
H	7.14730	-2.92010	2.17240
H	12.45000	-1.86510	3.00830
H	11.25820	-1.41660	1.77470
H	10.75050	-1.61570	3.46650
H	12.40800	-4.16980	4.05540
H	10.72240	-3.88720	4.54280
H	11.16120	-5.34640	3.63250
H	11.93270	-3.48260	0.39840
H	13.12590	-3.89330	1.64690
H	11.92940	-5.09350	1.15030

39. N1110c_WP

N	9.78720	-3.64260	2.07590
C	9.08950	-4.84730	1.61290
C	9.10970	-4.92940	0.06510
N	9.40890	-6.08550	-0.59730
C	9.85440	-7.39320	-0.11210
C	11.39160	-7.47700	-0.15740
O	11.88620	-8.07880	0.93130
C	13.32040	-8.32860	0.97520
O	12.07980	-7.06440	-1.07800
O	8.85520	-3.91050	-0.60830
C	7.62050	-4.88360	2.21330
C	7.74840	-5.01610	3.74530
C	6.83630	-6.09920	1.69060
C	6.83700	-3.59830	1.88670
C	9.21240	-8.58880	-0.93050
C	9.80680	-9.92340	-0.42750
C	7.69140	-8.61490	-0.68330
C	9.47170	-8.45850	-2.44600
C	11.22310	-3.68080	2.45390
C	11.44040	-4.64120	3.63870
C	12.15330	-4.07220	1.28470
C	11.56930	-2.25060	2.90900
H	9.63050	-2.88770	1.41040
H	9.60070	-5.72210	2.02200
H	9.45540	-5.94370	-1.60490
H	9.54660	-7.51530	0.92780
H	13.49580	-8.81180	1.93660
H	13.60670	-8.98880	0.15280
H	13.86800	-7.38630	0.90960
H	8.26260	-5.94520	4.02170
H	8.30700	-4.17640	4.16600
H	6.75220	-5.03600	4.20320
H	5.84990	-6.13720	2.16790
H	6.67530	-6.05430	0.60900
H	7.34760	-7.03920	1.92760
H	6.67780	-3.48180	0.81050
H	5.85430	-3.63660	2.37240
H	7.36020	-2.71170	2.25780
H	9.26840	-10.75480	-0.89560
H	10.86600	-10.03460	-0.68470
H	9.70440	-10.02620	0.65920
H	7.24580	-9.43150	-1.26250
H	7.45940	-8.79050	0.37250
H	7.21140	-7.68270	-0.99420
H	8.98820	-7.57350	-2.87680
H	10.53940	-8.42020	-2.68140
H	9.04720	-9.33180	-2.95390
H	12.48430	-4.59200	3.96730

H	10.79940	-4.36510	4.48200
H	11.23320	-5.68490	3.37800
H	13.20510	-4.03920	1.59310
H	11.94860	-5.08700	0.93220
H	12.02210	-3.38780	0.43850
H	10.94700	-1.95770	3.76120
H	12.62190	-2.18660	3.20580
H	11.40260	-1.53090	2.09780

40. N111'0c_WP

N	9.66800	-3.56240	2.06820
C	9.11770	-4.84190	1.61400
C	9.10980	-4.92960	0.06480
N	9.40880	-6.08520	-0.59720
C	9.60260	-7.45730	-0.12470
C	8.40100	-8.29840	-0.59270
O	8.09360	-9.26800	0.27180
C	7.01090	-10.17390	-0.08420
O	7.80740	-8.10900	-1.64410
O	8.85530	-3.91070	-0.60820
C	7.67010	-5.03810	2.24200
C	7.84190	-5.16880	3.76940
C	6.98590	-6.31690	1.72500
C	6.76210	-3.83160	1.93520
C	10.97310	-8.08850	-0.60590
C	12.12530	-7.22600	-0.05810
C	11.12170	-9.51460	-0.03180
C	11.06850	-8.14090	-2.14370
C	11.10910	-3.38360	2.37910
C	12.01990	-3.48330	1.13580
C	11.22900	-1.96420	2.96770
C	11.56230	-4.39790	3.44540
H	9.37330	-2.82880	1.42630
H	9.73560	-5.65170	2.01150
H	9.31050	-5.98270	-1.60590
H	6.07970	-9.61480	-0.20030
H	7.25380	-10.69910	-1.01090
H	6.94090	-10.87340	0.74910
H	6.86390	-5.29280	4.24960
H	8.45420	-6.04310	4.02420
H	8.32440	-4.28050	4.18410
H	6.01070	-6.43370	2.21160
H	6.80330	-6.28330	0.64520
H	7.56960	-7.21330	1.95840
H	6.59050	-3.71630	0.85990
H	5.78790	-3.97380	2.41820
H	7.19410	-2.90240	2.31840
H	13.08430	-7.68190	-0.32880
H	12.10530	-6.21790	-0.47920
H	12.08830	-7.15130	1.03530
H	12.12400	-9.89120	-0.26530
H	11.00470	-9.52320	1.05840
H	10.39750	-10.21620	-0.45670
H	10.29070	-8.77400	-2.58300
H	10.99970	-7.14440	-2.59530
H	12.03960	-8.56010	-2.43010
H	13.06890	-3.31000	1.40540
H	11.95650	-4.47130	0.67410

H	11.73160	-2.73760	0.38580
H	12.27290	-1.73510	3.21000
H	10.87730	-1.21330	2.24910
H	10.62920	-1.87360	3.87930
H	11.54910	-5.43000	3.07850
H	12.59150	-4.17570	3.74910
H	10.92190	-4.34050	4.33170
H	9.61330	-7.46450	0.96620

41. N0000_Anti120GP

N	8.78610	-3.74660	2.27810
C	9.11430	-4.96140	1.55800
C	9.10040	-4.89210	0.03860
N	9.42600	-6.15290	-0.59610
C	8.33580	-6.67700	-1.42700
C	8.89840	-7.65100	-2.44430
O	7.94660	-8.48100	-2.90440
C	8.35320	-9.40210	-3.93970
O	10.05160	-7.65380	-2.82390
O	8.84740	-3.88050	-0.58300
H	7.86240	-3.41380	2.01370
H	9.43530	-3.00310	2.03430
H	8.43280	-5.77250	1.84980
H	10.10900	-5.32080	1.85360
H	10.22970	-6.00680	-1.21160
H	7.81630	-5.88990	-1.99880
H	7.59000	-7.18700	-0.80990
H	7.45940	-9.97610	-4.18060
H	8.71010	-8.85420	-4.81470
H	9.14710	-10.05570	-3.57200

42. N0010_Anti120GP

N	8.95610	-3.71310	2.26990
C	9.25490	-4.93170	1.54410
C	9.10100	-4.89420	0.02810
N	9.42730	-6.15790	-0.59090
C	8.40590	-6.91730	-1.35070
C	9.02610	-8.31460	-1.44170
O	8.23480	-9.25670	-0.89160
C	8.74910	-10.60510	-0.91310
O	10.11050	-8.55230	-1.93560
O	8.84560	-3.87340	-0.57770
C	7.93290	-6.40030	-2.76810
C	6.85720	-5.30750	-2.59620
C	9.11260	-5.84750	-3.58860
C	7.28580	-7.57990	-3.53130
H	7.98040	-3.45460	2.14910
H	9.50250	-2.93890	1.90110
H	8.64490	-5.76130	1.92560
H	10.29120	-5.24270	1.73620
H	10.24640	-6.02940	-1.18470
H	7.52020	-6.99850	-0.71290
H	8.90350	-10.93630	-1.94270
H	9.69660	-10.65610	-0.37200
H	7.98910	-11.21160	-0.42190
H	6.48870	-4.99480	-3.58000
H	5.99750	-5.68610	-2.02890
H	7.24830	-4.42490	-2.08880
H	9.52300	-4.94040	-3.13380
H	9.91330	-6.58680	-3.69300
H	8.76920	-5.58020	-4.59410
H	6.84550	-7.21550	-4.46550
H	8.01590	-8.35260	-3.79360
H	6.48430	-8.04760	-2.94790

43. N0100_Anti120GP

N	10.61200	-5.26030	1.91720
C	9.20300	-4.98640	1.57570
C	9.09930	-4.88780	0.05040
N	9.42590	-6.15230	-0.60250
C	8.34390	-6.62880	-1.47150
C	8.90590	-7.59770	-2.49340
O	7.94600	-8.39790	-2.98880
C	8.35410	-9.30990	-4.03110
O	10.06700	-7.62170	-2.84840
O	8.84870	-3.88540	-0.58840
C	8.61730	-3.80680	2.40300
C	9.42190	-2.50510	2.20430
C	8.66590	-4.20560	3.89370
C	7.14490	-3.57580	2.00270
H	10.90420	-6.14740	1.51600
H	11.22920	-4.53350	1.56370
H	8.64650	-5.89180	1.85180
H	10.23900	-6.00200	-1.20420
H	7.86130	-5.81530	-2.03850
H	7.56830	-7.12930	-0.88380
H	7.45390	-9.86180	-4.29840
H	8.73850	-8.75530	-4.89010
H	9.12770	-9.98530	-3.65970
H	10.45560	-2.61580	2.55130
H	9.43110	-2.19180	1.15740
H	8.97270	-1.69860	2.79480
H	8.26840	-3.39350	4.51280
H	8.05760	-5.09820	4.08550
H	9.68850	-4.42010	4.21320
H	6.56130	-4.50160	2.08890
H	6.68720	-2.83980	2.67310
H	7.05360	-3.20370	0.97960

44. N0110_Anti120GP

N	10.76100	-5.11850	1.79710
C	9.31550	-4.95210	1.55970
C	9.10000	-4.89040	0.03950
N	9.42720	-6.15740	-0.59700
C	8.41440	-6.86250	-1.41870
C	8.98590	-8.28150	-1.49600
O	8.13380	-9.19940	-0.99770
C	8.59770	-10.56590	-1.01000
O	10.08540	-8.55580	-1.93550
O	8.84670	-3.87780	-0.58300
C	8.71260	-3.79510	2.40800
C	9.43390	-2.45330	2.16250
C	7.21150	-3.64780	2.08360
C	8.86130	-4.17810	3.89620
C	8.03160	-6.31630	-2.85360
C	7.40670	-7.47210	-3.67080
C	6.96690	-5.20640	-2.72930
C	9.26680	-5.77600	-3.59700
H	11.30140	-4.37030	1.37040
H	11.08510	-6.00510	1.42120
H	8.84630	-5.88490	1.89610
H	10.26760	-6.03650	-1.16070
H	7.49540	-6.91730	-0.82690
H	7.79210	-11.14800	-0.56410
H	8.79040	-10.89250	-2.03460
H	9.51450	-10.65870	-0.42370
H	10.48780	-2.50670	2.45810
H	9.37440	-2.14810	1.11500
H	8.97130	-1.66820	2.77140
H	6.74740	-2.93570	2.77490
H	7.04760	-3.28460	1.06620
H	6.68520	-4.60430	2.19920
H	8.46540	-3.37840	4.53240
H	8.30400	-5.09410	4.12810
H	9.90920	-4.34480	4.15800
H	7.02710	-7.08520	-4.62230
H	8.13510	-8.25570	-3.90340
H	6.56420	-7.93140	-3.14100
H	6.66280	-4.87370	-3.72860
H	6.06890	-5.57760	-2.21910
H	7.34260	-4.33970	-2.18510
H	9.66780	-4.88400	-3.10530
H	10.05740	-6.53020	-3.66750
H	8.98730	-5.48760	-4.61640

45. N011'0_Anti120GP

N	10.60650	-5.22390	1.92610
C	9.19300	-4.98470	1.57490
C	9.09930	-4.88790	0.04780
N	9.42650	-6.15480	-0.60140
C	8.33470	-6.62270	-1.48500
C	8.41850	-5.90980	-2.83860
O	7.21270	-5.51720	-3.28550
C	7.20180	-4.81650	-4.54710
O	9.45730	-5.72880	-3.44500
O	8.84800	-3.88280	-0.58690
C	8.57460	-3.81780	2.39710
C	9.35450	-2.49950	2.20990
C	8.61420	-4.21770	3.88780
C	7.10250	-3.61650	1.98010
C	8.35240	-8.18680	-1.62460
C	7.25770	-8.63100	-2.61590
C	8.04590	-8.78820	-0.23900
C	9.72480	-8.69250	-2.10940
H	11.20900	-4.48710	1.56790
H	10.92190	-6.10810	1.53670
H	8.65560	-5.90120	1.84930
H	10.23840	-5.99200	-1.20160
H	7.81080	-3.91280	-4.47740
H	7.59210	-5.45670	-5.34180
H	6.15720	-4.56710	-4.72940
H	8.88150	-1.70340	2.79580
H	10.38580	-2.58980	2.56970
H	9.37050	-2.18500	1.16340
H	9.63700	-4.41420	4.21810
H	8.19600	-3.41370	4.50400
H	8.01970	-5.12110	4.07240
H	6.53690	-4.55410	2.05890
H	6.62180	-2.89060	2.64540
H	7.01560	-3.24580	0.95610
H	7.46960	-8.30340	-3.63890
H	7.19150	-9.72420	-2.62860
H	6.27440	-8.23800	-2.33450
H	7.04820	-8.49570	0.11080
H	8.07260	-9.88240	-0.28800
H	8.78010	-8.46050	0.50180
H	10.51440	-8.44400	-1.39320
H	9.70160	-9.78320	-2.20990
H	9.99700	-8.26830	-3.08030
H	7.37850	-6.35000	-1.02730

46. N1000_Anti120GP

N	8.62350	-3.75210	2.15810
C	9.14490	-4.95390	1.54530
C	9.10060	-4.89290	0.03840
N	9.42580	-6.15220	-0.59580
C	8.34710	-6.65540	-1.45530
C	8.92610	-7.60690	-2.48400
O	7.98770	-8.44150	-2.96310
C	8.41340	-9.34340	-4.00710
O	10.08210	-7.59030	-2.85600
O	8.84740	-3.88040	-0.58310
C	9.06030	-3.40460	3.52650
C	8.64490	-4.53320	4.48730
C	8.31390	-2.11310	3.90080
C	10.58460	-3.15910	3.62960
H	8.74900	-2.96810	1.52210
H	8.58430	-5.84920	1.84880
H	10.20110	-5.18830	1.78340
H	10.24200	-6.00740	-1.19520
H	7.83930	-5.85400	-2.01720
H	7.59060	-7.17630	-0.86090
H	9.20990	-9.99490	-3.64070
H	7.52740	-9.92240	-4.26500
H	8.77390	-8.77990	-4.87090
H	9.17510	-5.46740	4.27180
H	8.87420	-4.25830	5.52220
H	7.56960	-4.72330	4.40880
H	7.23220	-2.26750	3.84520
H	8.57280	-1.79320	4.91550
H	8.57640	-1.29710	3.21650
H	10.89370	-2.35300	2.95420
H	10.86610	-2.87120	4.64870
H	11.16250	-4.05460	3.37590

47. N1010_Anti120GP

N	8.75720	-3.72770	2.16150
C	9.24190	-4.93570	1.53170
C	9.10130	-4.89530	0.02700
N	9.42710	-6.15740	-0.59010
C	8.42850	-6.88220	-1.40960
C	9.03010	-8.28730	-1.49970
O	8.18930	-9.23020	-1.03140
C	8.67930	-10.58700	-1.06380
O	10.14080	-8.53060	-1.92900
O	8.84540	-3.87280	-0.57740
C	8.02860	-6.33390	-2.83920
C	9.25090	-5.76670	-3.58350
C	7.42040	-7.49370	-3.66250
C	6.94550	-5.24400	-2.69870
C	9.25700	-3.37790	3.50740
C	10.78320	-3.12590	3.53620
C	8.52380	-2.08990	3.91770
C	8.89410	-4.50720	4.48820
H	8.86090	-2.94740	1.51710
H	8.70920	-5.82720	1.88940
H	10.31440	-5.16100	1.69710
H	10.28860	-6.05750	-1.12590
H	7.51420	-6.95880	-0.81340
H	9.58820	-10.67480	-0.46430
H	7.87780	-11.19330	-0.64320
H	8.89460	-10.88890	-2.09160
H	8.96140	-5.47320	-4.59870
H	9.64060	-4.87390	-3.08460
H	10.05320	-6.50750	-3.66510
H	6.58820	-7.97170	-3.13330
H	8.16160	-8.26280	-3.90420
H	7.03090	-7.10600	-4.60980
H	6.05570	-5.63640	-2.19010
H	7.30670	-4.37650	-2.14550
H	6.63270	-4.90480	-3.69320
H	11.11450	-2.84190	4.54140
H	11.35070	-4.01750	3.24820
H	11.05470	-2.31490	2.85070
H	8.74530	-1.27410	3.21900
H	7.44170	-2.25070	3.91870
H	8.83320	-1.76600	4.91700
H	9.42420	-5.43680	4.25410
H	9.16380	-4.22550	5.51140
H	7.81850	-4.70830	4.45600

48. N1100_Anti120GP

N	10.01280	-3.72750	2.03890
C	9.20170	-4.86370	1.59000
C	9.09980	-4.89010	0.05260
N	9.42470	-6.14760	-0.60290
C	8.31830	-6.75980	-1.34990
C	8.88000	-7.59670	-2.48420
O	7.97810	-8.49430	-2.91840
C	8.37650	-9.30050	-4.04790
O	9.98680	-7.45370	-2.96310
O	8.84930	-3.88780	-0.59020
C	7.78900	-4.85310	2.29270
C	8.01460	-5.08490	3.79920
C	6.90900	-5.99240	1.74260
C	7.06730	-3.50790	2.08760
C	11.48770	-3.87560	2.13920
C	12.02900	-2.46840	2.44620
C	11.82700	-4.81090	3.31420
C	12.14930	-4.40490	0.84630
H	9.79250	-2.92170	1.45820
H	9.69340	-5.79220	1.88750
H	10.13820	-5.92620	-1.30270
H	7.65010	-6.01490	-1.81420
H	7.70930	-7.39670	-0.70450
H	7.52700	-9.95160	-4.25000
H	8.59180	-8.66480	-4.90980
H	9.26390	-9.88650	-3.79870
H	7.05960	-5.04070	4.33540
H	8.45860	-6.06980	3.98920
H	8.67970	-4.32260	4.21210
H	5.99040	-6.07560	2.33380
H	6.60470	-5.81190	0.70590
H	7.42250	-6.96010	1.79100
H	6.90980	-3.28510	1.02800
H	6.08690	-3.53600	2.57700
H	7.63620	-2.68540	2.53120
H	13.11480	-2.49280	2.58270
H	11.81210	-1.77560	1.62390
H	11.57220	-2.07150	3.35810
H	12.91230	-4.87480	3.44700
H	11.38460	-4.43610	4.24210
H	11.46000	-5.82910	3.14800
H	11.91950	-3.75590	-0.00640
H	13.23920	-4.44120	0.95430
H	11.81310	-5.42070	0.61100

49. N1110_Anti120GP

N	10.02910	-3.67690	1.99590
C	9.26210	-4.85320	1.57740
C	9.10040	-4.89200	0.04120
N	9.42620	-6.15380	-0.59730
C	8.45090	-6.94370	-1.38630
C	9.18010	-8.27750	-1.57210
O	8.46520	-9.31590	-1.09540
C	9.07770	-10.61640	-1.21980
O	10.27990	-8.39560	-2.07640
O	8.84720	-3.87970	-0.58440
C	7.87670	-4.90960	2.33310
C	8.16910	-5.13680	3.82870
C	7.02420	-6.08170	1.81070
C	7.09210	-3.59470	2.16390
C	7.91420	-6.38500	-2.76500
C	7.32530	-7.56170	-3.57800
C	6.77480	-5.37660	-2.50930
C	9.03470	-5.71210	-3.57850
C	11.51100	-3.75380	2.05800
C	11.92560	-4.65360	3.23670
C	12.16410	-4.27230	0.75600
C	11.99450	-2.31830	2.32900
H	9.75530	-2.88820	1.41450
H	9.80690	-5.75690	1.85750
H	10.24560	-5.99360	-1.18390
H	7.58690	-7.13920	-0.74920
H	8.36960	-11.31300	-0.77230
H	9.24570	-10.85840	-2.27200
H	10.03200	-10.63750	-0.68870
H	8.65920	-6.10350	3.99670
H	8.81760	-4.34920	4.21990
H	7.23430	-5.13320	4.40110
H	6.13770	-6.21060	2.44130
H	6.66480	-5.90160	0.79170
H	7.58000	-7.02620	1.82040
H	6.88800	-3.37120	1.11210
H	6.13060	-3.66850	2.68510
H	7.64030	-2.75170	2.59470
H	6.83580	-7.17770	-4.47930
H	8.09730	-8.26750	-3.90180
H	6.57420	-8.11490	-3.00290
H	6.36570	-5.03070	-3.46550
H	5.95310	-5.84220	-1.95060
H	7.12030	-4.50090	-1.95850
H	9.40050	-4.81030	-3.07790
H	9.87640	-6.39220	-3.74440
H	8.64870	-5.40980	-4.55870
H	13.01630	-4.67050	3.33700

H	11.49630	-4.28140	4.17170
H	11.59560	-5.68850	3.09860
H	13.25680	-4.25250	0.83490
H	11.87530	-5.30870	0.54880
H	11.87900	-3.65160	-0.10080
H	11.54070	-1.92710	3.24490
H	13.08320	-2.29090	2.44070
H	11.72710	-1.65040	1.50110

50. N111'0_Anti120GP

N	10.19430	-3.83830	2.00050
C	9.22620	-4.86310	1.58860
C	9.09960	-4.88910	0.05010
N	9.42610	-6.15290	-0.60210
C	8.30940	-6.76710	-1.35820
C	8.12300	-6.04660	-2.69810
O	6.83180	-5.75190	-2.93870
C	6.56060	-5.05340	-4.17200
O	9.03080	-5.77650	-3.46050
O	8.84820	-3.88350	-0.58850
C	7.84850	-4.65410	2.32670
C	8.08120	-4.91310	3.82820
C	6.80420	-5.66170	1.80840
C	7.31080	-3.22310	2.13820
C	8.53380	-8.31110	-1.53910
C	8.47470	-8.96820	-0.14600
C	7.40430	-8.89210	-2.41450
C	9.89840	-8.61510	-2.18710
C	11.63770	-4.16700	2.11140
C	12.24020	-4.77260	0.82330
C	12.34390	-2.83600	2.42450
C	11.85370	-5.13670	3.28780
H	10.07260	-3.01390	1.41770
H	9.60160	-5.84250	1.89110
H	10.13930	-5.92330	-1.29900
H	7.10030	-4.10430	-4.19050
H	6.86480	-5.66050	-5.02810
H	5.48370	-4.88810	-4.17910
H	7.15630	-4.73800	4.38970
H	8.39170	-5.94900	4.01230
H	8.85410	-4.24750	4.22030
H	5.89990	-5.61580	2.42530
H	6.49880	-5.44130	0.77960
H	7.17860	-6.69140	1.84860
H	7.15180	-2.98290	1.08330
H	6.35230	-3.11810	2.65960
H	8.00020	-2.48590	2.56060
H	8.66250	-10.04450	-0.22910
H	9.22730	-8.53900	0.52060
H	7.48860	-8.83790	0.31560
H	7.48890	-9.98350	-2.45300
H	6.41500	-8.64640	-2.01240
H	7.45040	-8.52370	-3.44490
H	9.99880	-8.14410	-3.16900
H	10.72270	-8.27100	-1.55430
H	10.01160	-9.69740	-2.31510
H	13.31790	-4.93640	0.93680
H	11.78430	-5.73980	0.58850

H	12.09290	-4.10150	-0.03100
H	13.41830	-2.99210	2.56460
H	12.21580	-2.12010	1.60340
H	11.93490	-2.38870	3.33570
H	11.37040	-6.10420	3.11740
H	12.92290	-5.32830	3.42850
H	11.45260	-4.71300	4.21330
H	7.38870	-6.64220	-0.78530

51. N0000_Anti-120GP

N	8.79550	-3.74440	2.27830
C	9.09680	-4.96590	1.55790
C	9.10040	-4.89210	0.03870
N	9.42600	-6.15280	-0.59620
C	10.64950	-6.09040	-1.40420
C	10.63700	-7.20920	-2.42800
O	11.87750	-7.48470	-2.86570
C	11.97750	-8.48290	-3.90460
O	9.63120	-7.75740	-2.83010
O	8.84740	-3.88050	-0.58300
H	7.87590	-3.39690	2.01860
H	9.45540	-3.01180	2.02980
H	8.38790	-5.75640	1.83830
H	10.07700	-5.35640	1.86470
H	8.66160	-6.40190	-1.22840
H	11.53660	-6.18750	-0.77110
H	10.74230	-5.14780	-1.96930
H	13.04100	-8.56130	-4.12600
H	11.58340	-9.43750	-3.54920
H	11.41930	-8.16630	-4.78850

52. N0010_Anti-120GP

N	8.81270	-3.74140	2.27730
C	9.11140	-4.96340	1.55630
C	9.10040	-4.89200	0.03570
N	9.42680	-6.15600	-0.59490
C	10.64670	-6.08460	-1.43040
C	10.30160	-5.50580	-2.80640
O	11.21010	-4.60790	-3.22630
C	10.95290	-3.99950	-4.50970
O	9.32110	-5.82820	-3.44940
O	8.84660	-3.87760	-0.58130
C	11.35020	-7.48540	-1.52480
C	11.84900	-7.85850	-0.11490
C	10.37680	-8.56940	-2.02630
C	12.55950	-7.39390	-2.47790
H	7.89130	-3.39570	2.02160
H	9.46970	-3.00860	2.02160
H	8.40790	-5.75570	1.84550
H	10.09640	-5.34790	1.85410
H	8.65090	-6.40250	-1.21360
H	11.35280	-5.39870	-0.95200
H	10.93450	-4.75960	-5.29440
H	9.99470	-3.47570	-4.48950
H	11.77370	-3.30080	-4.66730
H	12.32500	-8.84520	-0.13190
H	12.59220	-7.13790	0.24860
H	11.02040	-7.88960	0.59750
H	9.53790	-8.69850	-1.33500
H	9.97380	-8.33150	-3.01500
H	10.89890	-9.53010	-2.09500
H	13.11800	-8.33580	-2.45820
H	12.25510	-7.21500	-3.51460
H	13.24520	-6.59050	-2.18580

53. N0100_Anti-120GP

N	9.57920	-3.64450	2.14720
C	9.11860	-4.91310	1.57860
C	9.10010	-4.89110	0.04680
N	9.42530	-6.15010	-0.60000
C	10.61450	-6.05120	-1.45590
C	10.58770	-7.16300	-2.48670
O	11.81770	-7.41330	-2.96890
C	11.89890	-8.40180	-4.01820
O	9.57920	-7.72780	-2.85890
O	8.84840	-3.88430	-0.58720
C	7.71790	-5.31980	2.18410
C	6.69860	-4.17760	2.00370
C	7.92470	-5.59000	3.68750
C	7.17320	-6.59860	1.51710
H	10.58190	-3.54210	2.01690
H	9.14180	-2.86700	1.65760
H	9.82390	-5.69890	1.87020
H	8.64500	-6.40950	-1.20850
H	11.52830	-6.13170	-0.85930
H	10.66220	-5.10260	-2.01640
H	12.95590	-8.46360	-4.27350
H	11.52940	-9.36460	-3.65870
H	11.30900	-8.08640	-4.88180
H	7.00370	-3.27950	2.54930
H	6.56130	-3.91180	0.95020
H	5.72450	-4.48700	2.39890
H	6.96360	-5.80710	4.16760
H	8.57960	-6.45530	3.84580
H	8.37420	-4.72430	4.18000
H	7.90600	-7.41110	1.53330
H	6.27710	-6.93630	2.04950
H	6.88020	-6.43080	0.47450

54. N0110_Anti-120GP

N	9.58470	-3.64050	2.14140
C	9.11980	-4.91010	1.57780
C	9.10010	-4.89110	0.04400
N	9.42610	-6.15300	-0.59880
C	10.63110	-6.05300	-1.45480
C	10.26930	-5.42490	-2.80490
O	11.16730	-4.50200	-3.19420
C	10.89670	-3.84540	-4.45040
O	9.28980	-5.73320	-3.45620
O	8.84770	-3.88140	-0.58570
C	7.71900	-5.30890	2.18710
C	6.70180	-4.16620	1.99990
C	7.17080	-6.59140	1.53040
C	7.92680	-5.56920	3.69210
C	11.32780	-7.45200	-1.60970
C	12.52230	-7.33070	-2.57820
C	11.84820	-7.87630	-0.22230
C	10.34360	-8.51560	-2.13330
H	9.15630	-2.86450	1.64110
H	10.58890	-3.54600	2.01640
H	9.82360	-5.69570	1.87250
H	8.64570	-6.39210	-1.21520
H	11.34910	-5.38780	-0.96490
H	11.71210	-3.13550	-4.58640
H	10.87720	-4.57390	-5.26450
H	9.93550	-3.32900	-4.40230
H	7.01260	-3.26370	2.53490
H	6.56080	-3.91040	0.94440
H	5.72860	-4.46840	2.40300
H	6.27290	-6.92210	2.06410
H	6.88080	-6.43150	0.48570
H	7.90120	-7.40620	1.55700
H	6.96630	-5.78450	4.17440
H	8.58350	-6.43230	3.85620
H	8.37540	-4.69970	4.17840
H	13.07700	-8.27490	-2.60170
H	12.20190	-7.11340	-3.60270
H	13.21600	-6.54100	-2.26840
H	12.31910	-8.86390	-0.28130
H	12.60040	-7.17190	0.15400
H	11.03110	-7.92840	0.50210
H	9.51580	-8.66760	-1.43360
H	9.92540	-8.24150	-3.10610
H	10.86210	-9.47440	-2.24450

55. N011'0_Anti-120GP

N	9.46170	-3.69160	2.18780
C	9.01580	-4.94230	1.56730
C	9.10090	-4.89370	0.03500
N	9.42670	-6.15550	-0.59410
C	10.65940	-6.29510	-1.40720
C	10.80470	-7.81570	-1.51680
O	11.97610	-8.25180	-1.01270
C	12.18980	-9.67860	-1.05520
O	9.95970	-8.55270	-1.98580
O	8.84630	-3.87630	-0.58140
C	7.56760	-5.32370	2.07190
C	6.58960	-4.15210	1.85530
C	7.67570	-5.62790	3.57980
C	7.02950	-6.57500	1.35000
C	10.76560	-5.60030	-2.82500
C	11.86060	-6.32110	-3.64670
C	11.20230	-4.12950	-2.65890
C	9.42920	-5.66770	-3.58600
H	9.07530	-2.89810	1.68090
H	10.47320	-3.61010	2.13040
H	9.68300	-5.74560	1.89700
H	8.63230	-6.46170	-1.15520
H	11.41120	-10.19420	-0.48820
H	12.17860	-10.03380	-2.08830
H	13.16810	-9.83740	-0.60250
H	5.58590	-4.44510	2.18350
H	6.88260	-3.27420	2.43910
H	6.52430	-3.86040	0.80190
H	8.11850	-4.78440	4.11500
H	6.68160	-5.82560	3.99710
H	8.29580	-6.51460	3.75950
H	7.73950	-7.40690	1.38910
H	6.09790	-6.90010	1.82620
H	6.79480	-6.37830	0.29740
H	11.57500	-7.34560	-3.90700
H	12.03150	-5.78110	-4.58420
H	12.81400	-6.35590	-3.10680
H	12.16440	-4.06140	-2.13510
H	11.33320	-3.66980	-3.64550
H	10.46430	-3.54350	-2.11050
H	8.65810	-5.07300	-3.08650
H	9.55710	-5.25690	-4.59390
H	9.07430	-6.69870	-3.68710
H	11.49380	-5.93500	-0.79740

56. N1000_Anti-120GP

N	8.61520	-3.74830	2.15510
C	9.11780	-4.95950	1.54650
C	9.10070	-4.89320	0.03890
N	9.42560	-6.15130	-0.59590
C	10.67650	-6.10280	-1.36220
C	10.67780	-7.21360	-2.39470
O	11.92740	-7.51190	-2.79050
C	12.04340	-8.50420	-3.83330
O	9.67600	-7.73800	-2.83620
O	8.84750	-3.88100	-0.58350
C	9.06710	-3.39270	3.51560
C	8.68590	-4.52620	4.48470
C	8.30680	-2.11280	3.90150
C	10.58910	-3.12350	3.59140
H	8.73360	-2.97020	1.51070
H	8.53020	-5.84160	1.83470
H	10.16420	-5.21930	1.80460
H	8.67830	-6.37540	-1.25720
H	11.54000	-6.22050	-0.70080
H	10.80470	-5.15790	-1.91660
H	11.52270	-8.16990	-4.73340
H	13.11230	-8.60240	-4.01850
H	11.61830	-9.45310	-3.49950
H	9.23010	-5.45130	4.26470
H	8.92410	-4.24370	5.51550
H	7.61340	-4.73650	4.42160
H	7.22720	-2.28700	3.87090
H	8.58280	-1.78350	4.90880
H	8.53860	-1.29560	3.20760
H	10.87130	-2.30670	2.91700
H	10.88790	-2.84030	4.60690
H	11.17510	-4.00780	3.31680

57. N1010_Anti-120GP

N	8.74830	-3.74350	2.24660
C	9.04320	-4.98160	1.55170
C	9.10050	-4.89240	0.03660
N	9.42660	-6.15530	-0.59520
C	10.66040	-6.08490	-1.40970
C	10.34120	-5.49730	-2.78830
O	11.26180	-4.60300	-3.18900
C	11.02950	-3.98740	-4.47370
O	9.36940	-5.81090	-3.44900
O	8.84670	-3.87780	-0.58190
C	11.35820	-7.48910	-1.50000
C	10.38760	-8.56490	-2.02390
C	12.58470	-7.40010	-2.43090
C	11.82970	-7.87290	-0.08370
C	9.86580	-3.00800	2.89060
C	11.04070	-2.71520	1.92870
C	9.27040	-1.67840	3.38270
C	10.36580	-3.81540	4.10260
H	8.26860	-3.11950	1.60130
H	8.27970	-5.74950	1.76220
H	9.98010	-5.43470	1.89340
H	8.66070	-6.38920	-1.23130
H	11.36200	-5.40530	-0.91600
H	10.07480	-3.45710	-4.46690
H	11.85760	-3.29380	-4.61510
H	11.01860	-4.74410	-5.26180
H	10.90500	-9.52850	-2.08750
H	9.53540	-8.69150	-1.34880
H	10.00430	-8.31990	-3.01880
H	13.26970	-6.60230	-2.12280
H	12.29970	-7.21450	-3.47190
H	13.13720	-8.34550	-2.40620
H	12.57120	-7.15940	0.29660
H	10.98810	-7.90240	0.61360
H	12.29920	-8.86280	-0.09740
H	11.82510	-2.14120	2.43540
H	11.50320	-3.63880	1.56180
H	10.70060	-2.14060	1.06110
H	8.90440	-1.07730	2.54140
H	8.43230	-1.86300	4.06170
H	10.02500	-1.08680	3.91110
H	10.80170	-4.77590	3.80610
H	11.14200	-3.25930	4.63940
H	9.53950	-4.01680	4.79110

58. N1100_Anti-120GP

N	9.40300	-3.62480	2.16440
C	9.05840	-4.92020	1.58010
C	9.10020	-4.89130	0.04850
N	9.42500	-6.14860	-0.60070
C	10.64950	-6.08370	-1.40910
C	10.58700	-7.14160	-2.49410
O	11.81330	-7.44620	-2.95200
C	11.86960	-8.39260	-4.04100
O	9.55770	-7.62470	-2.92050
O	8.84870	-3.88560	-0.58830
C	7.64050	-5.41130	2.10350
C	7.75950	-5.62750	3.62440
C	7.22190	-6.74450	1.45140
C	6.56350	-4.34620	1.82320
C	10.81910	-3.33210	2.50160
C	10.85490	-1.84870	2.90910
C	11.24870	-4.19220	3.70420
C	11.79900	-3.55640	1.32670
H	9.05270	-2.89570	1.54690
H	9.77820	-5.67890	1.90110
H	8.66640	-6.36380	-1.25310
H	11.53590	-6.25140	-0.79120
H	10.77570	-5.11510	-1.92100
H	12.92930	-8.51220	-4.26310
H	11.42680	-9.34380	-3.73750
H	11.33310	-8.00310	-4.90930
H	6.78690	-5.91630	4.03900
H	8.47150	-6.42860	3.85740
H	8.09180	-4.71350	4.12200
H	6.32380	-7.12630	1.95000
H	6.97430	-6.63290	0.39070
H	8.00580	-7.50370	1.53170
H	6.45910	-4.13880	0.75280
H	5.59230	-4.69730	2.19000
H	6.79420	-3.40700	2.33420
H	11.86250	-1.55570	3.22130
H	10.56430	-1.20410	2.07070
H	10.16510	-1.66340	3.73810
H	12.27130	-3.94050	4.00560
H	10.58330	-4.02010	4.55520
H	11.23200	-5.26230	3.47180
H	11.51100	-2.95990	0.45470
H	12.81850	-3.27280	1.61130
H	11.83090	-4.61010	1.02730

59. N1110_Anti-120GP

N	9.41840	-3.59790	2.14390
C	9.08580	-4.90660	1.58130
C	9.10010	-4.89100	0.04600
N	9.42570	-6.15170	-0.59970
C	10.63130	-6.07010	-1.45630
C	10.27680	-5.43130	-2.80370
O	11.18420	-4.51410	-3.18690
C	10.91860	-3.84570	-4.43790
O	9.29470	-5.72440	-3.45810
O	8.84800	-3.88290	-0.58680
C	7.68270	-5.41040	2.13360
C	7.82440	-5.60040	3.65610
C	7.27260	-6.75990	1.51050
C	6.58640	-4.36540	1.85270
C	11.29870	-7.48260	-1.62120
C	12.49160	-7.37840	-2.59370
C	11.81630	-7.92680	-0.23890
C	10.29280	-8.52440	-2.14790
C	10.82370	-3.29440	2.51510
C	11.21920	-4.11870	3.75390
C	11.83830	-3.55230	1.37750
C	10.84740	-1.79990	2.88020
H	9.08320	-2.88350	1.50150
H	9.82240	-5.65080	1.89740
H	8.64370	-6.38830	-1.21470
H	11.36170	-5.42110	-0.96710
H	11.73910	-3.14080	-4.56830
H	10.89400	-4.56750	-5.25790
H	9.96110	-3.32300	-4.38620
H	8.55510	-6.38300	3.89390
H	8.14390	-4.67200	4.13480
H	6.86280	-5.90130	4.08730
H	6.38600	-7.14450	2.02690
H	7.00940	-6.66940	0.45140
H	8.06680	-7.50810	1.59620
H	6.45830	-4.18140	0.78050
H	5.62710	-4.72030	2.24620
H	6.81660	-3.41330	2.33920
H	13.02840	-8.33260	-2.62500
H	12.17120	-7.14930	-3.61550
H	13.20110	-6.60360	-2.28200
H	12.26360	-8.92480	-0.30640
H	12.58680	-7.24290	0.13760
H	11.00190	-7.96430	0.48930
H	9.46690	-8.66880	-1.44430
H	9.87340	-8.23450	-3.11560
H	10.79400	-9.49100	-2.27080
H	12.23780	-3.86680	4.06860

H	10.53870	-3.91330	4.58530
H	11.19640	-5.19540	3.55530
H	12.84670	-3.24700	1.67900
H	11.88860	-4.61650	1.12210
H	11.56990	-2.99250	0.47530
H	10.13050	-1.59020	3.68010
H	11.84450	-1.49860	3.21760
H	10.58600	-1.17970	2.01420

60. N111'0_Anti-120GP

N	9.31030	-3.67480	2.19890
C	8.96100	-4.94730	1.56670
C	9.10080	-4.89340	0.03740
N	9.42640	-6.15440	-0.59520
C	10.68080	-6.31970	-1.37070
C	10.81760	-7.84350	-1.44870
O	11.95900	-8.27960	-0.87770
C	12.16090	-9.70850	-0.88280
O	9.99240	-8.58350	-1.94680
O	8.84670	-3.87780	-0.58260
C	7.49410	-5.39120	1.99080
C	7.51900	-5.65510	3.50920
C	7.05330	-6.68600	1.27900
C	6.47820	-4.27350	1.68710
C	10.82320	-5.65380	-2.79790
C	11.23090	-4.17310	-2.65020
C	11.95500	-6.37750	-3.56530
C	9.51430	-5.75590	-3.60210
C	10.70760	-3.44220	2.64440
C	11.76240	-3.69590	1.54330
C	10.77220	-1.96570	3.07240
C	11.01230	-4.33040	3.86480
H	9.02880	-2.92170	1.57510
H	9.63120	-5.73460	1.92210
H	8.64610	-6.42700	-1.19210
H	11.35000	-10.20680	-0.34670
H	12.19810	-10.08360	-1.90830
H	13.11370	-9.86720	-0.37870
H	6.51310	-5.91010	3.86200
H	8.18100	-6.49380	3.75670
H	7.86280	-4.77100	4.05100
H	6.11670	-7.04300	1.72170
H	6.85810	-6.53120	0.21220
H	7.79890	-7.48140	1.37270
H	6.44400	-4.02720	0.62060
H	5.47470	-4.59590	1.98720
H	6.71660	-3.36200	2.24260
H	11.38500	-3.73240	-3.64210
H	10.46550	-3.58940	-2.13770
H	12.17370	-4.07670	-2.09730
H	12.15400	-5.85410	-4.50670
H	12.88840	-6.39020	-2.99050
H	11.69050	-7.41030	-3.81460
H	9.17420	-6.79270	-3.69030
H	8.71920	-5.15830	-3.14460
H	9.67190	-5.36650	-4.61410
H	12.76860	-3.45590	1.90510
H	11.77480	-4.74770	1.23590

H	11.56460	-3.08070	0.65910
H	11.76430	-1.71790	3.46350
H	10.57260	-1.30100	2.22300
H	10.02980	-1.75990	3.84960
H	10.96530	-5.39660	3.61940
H	12.02100	-4.12690	4.24060
H	10.29670	-4.13570	4.66890
H	11.50240	-5.95380	-0.74930

61. N0000_Syn60GP

N	10.46030	-5.16780	2.06520
C	9.10730	-4.85740	1.59010
C	9.09930	-4.88820	0.06040
N	9.42170	-6.13550	-0.60680
C	8.55490	-7.27270	-0.34500
C	8.91090	-8.16580	0.84200
O	7.95560	-9.08880	1.04620
C	8.19900	-10.04940	2.09730
O	9.93570	-8.09870	1.49470
O	8.85290	-3.90170	-0.59410
H	10.60170	-6.17070	2.14960
H	10.63870	-4.73150	2.96300
H	8.83780	-3.84220	1.88760
H	8.32840	-5.53770	1.97820
H	10.40380	-6.36510	-0.46730
H	8.55210	-7.92910	-1.22610
H	7.51880	-6.93660	-0.23580
H	7.32790	-10.70330	2.09610
H	9.10950	-10.61520	1.88860
H	8.29960	-9.54060	3.05850

62. N0010_Syn60GP

N	10.51630	-5.09750	2.02890
C	9.13590	-4.85360	1.58930
C	9.09980	-4.89040	0.05850
N	9.42130	-6.13390	-0.60540
C	8.60870	-7.30970	-0.28590
C	9.10500	-8.04750	0.96220
O	8.09540	-8.56630	1.68660
C	8.46700	-9.32410	2.85830
O	10.27630	-8.15160	1.28300
O	8.85280	-3.90120	-0.59370
C	8.51550	-8.26460	-1.54130
C	7.70870	-9.53240	-1.19690
C	7.78390	-7.49230	-2.65630
C	9.92110	-8.66650	-2.02860
H	10.73760	-6.08830	2.07320
H	10.68770	-4.68300	2.93840
H	8.83490	-3.84890	1.89220
H	8.39720	-5.56360	1.99990
H	10.41740	-6.33010	-0.52810
H	7.58790	-6.97290	-0.07760
H	9.09100	-10.17560	2.57750
H	9.01470	-8.69240	3.56120
H	7.52700	-9.66070	3.29370
H	6.72900	-9.28720	-0.77270
H	7.54280	-10.11730	-2.10780
H	8.23110	-10.17910	-0.48420
H	6.75120	-7.26310	-2.36690
H	8.29250	-6.55180	-2.87980
H	7.74760	-8.09770	-3.56880
H	10.49060	-7.79500	-2.36560
H	10.49690	-9.17640	-1.24930
H	9.83360	-9.34910	-2.88100

63. N0100t_Syn60GP

N	10.47150	-5.36600	1.97810
C	9.12640	-4.89430	1.61180
C	9.09980	-4.89010	0.06130
N	9.42080	-6.13200	-0.60660
C	8.54580	-7.26380	-0.30870
C	9.14490	-8.52940	-0.89660
O	8.19130	-9.44670	-1.14860
C	8.65520	-10.72320	-1.63690
O	10.33070	-8.71310	-1.07140
O	8.85330	-3.90340	-0.59510
C	8.61140	-3.59590	2.31990
C	8.62940	-3.84530	3.84470
C	7.15280	-3.31940	1.89630
C	9.49030	-2.36790	2.00690
H	11.15840	-4.61980	1.90900
H	10.50020	-5.72490	2.92630
H	8.43790	-5.69560	1.91470
H	10.36920	-6.40030	-0.33740
H	7.55330	-7.09960	-0.73730
H	8.40870	-7.47600	0.76760
H	9.18400	-10.59520	-2.58390
H	9.32420	-11.18770	-0.90880
H	7.75630	-11.32300	-1.77410
H	9.64600	-3.98290	4.22900
H	8.19820	-2.98720	4.37150
H	8.03840	-4.72950	4.11650
H	6.51250	-4.18970	2.09140
H	6.74620	-2.48090	2.47310
H	7.08040	-3.06870	0.83640
H	9.47690	-2.12660	0.94230
H	9.12310	-1.49700	2.56170
H	10.53130	-2.52290	2.31680

64. N0110_Syn60GP

N	10.67410	-4.72140	1.79570
C	9.22380	-4.85920	1.60100
C	9.10020	-4.89190	0.05870
N	9.42080	-6.13210	-0.60500
C	8.57850	-7.29110	-0.27600
C	9.33730	-8.25820	0.63340
O	8.53950	-8.81590	1.56960
C	9.16340	-9.78470	2.43860
O	10.52440	-8.50080	0.53800
O	8.85280	-3.90150	-0.59420
C	8.29050	-3.85910	2.36150
C	8.64350	-2.38260	2.09260
C	6.82940	-4.12770	1.94600
C	8.43280	-4.13780	3.87360
C	8.04770	-7.99190	-1.58640
C	7.18530	-9.21670	-1.22330
C	7.17620	-6.96330	-2.33310
C	9.21700	-8.42380	-2.48970
H	10.96940	-5.01030	2.72190
H	10.98420	-3.76770	1.63650
H	8.96490	-5.85970	1.96700
H	10.40690	-6.35340	-0.46900
H	7.69170	-6.95350	0.27280
H	8.38010	-10.10290	3.12590
H	9.53460	-10.63180	1.85680
H	9.99570	-9.32970	2.98040
H	7.96750	-1.73230	2.65930
H	9.66180	-2.14370	2.42180
H	8.55570	-2.13520	1.03310
H	6.14420	-3.51870	2.54580
H	6.65900	-3.88290	0.89390
H	6.55630	-5.17870	2.10590
H	7.76630	-3.48340	4.44580
H	8.17080	-5.17460	4.11900
H	9.45280	-3.95090	4.22840
H	6.72500	-9.62470	-2.12960
H	7.77370	-10.01870	-0.76520
H	6.37950	-8.95390	-0.52840
H	6.81600	-7.39030	-3.27560
H	6.29810	-6.67990	-1.73940
H	7.74550	-6.05780	-2.55740
H	9.82510	-7.56170	-2.77770
H	9.86830	-9.15360	-1.99810
H	8.82680	-8.88290	-3.40490

65. N011'0_Syn60GP

N	10.63750	-5.21960	1.86760
C	9.22950	-4.86480	1.60730
C	9.09910	-4.88770	0.06450
N	9.42130	-6.13410	-0.60870
C	8.70230	-7.37050	-0.22520
C	7.27510	-7.21440	-0.77410
O	6.60840	-6.30620	-0.02230
C	5.29090	-5.94350	-0.48490
O	6.77910	-7.76690	-1.73110
O	8.85340	-3.90380	-0.59620
C	8.68450	-3.59100	2.33710
C	8.79620	-3.83250	3.85920
C	7.19440	-3.39060	1.98990
C	9.47690	-2.31690	1.97840
C	9.45630	-8.64710	-0.71560
C	8.62000	-9.89050	-0.34260
C	10.80370	-8.74160	0.03740
C	9.72510	-8.63090	-2.23430
H	10.76400	-5.57900	2.80800
H	11.24470	-4.41080	1.76420
H	8.63150	-5.70790	1.97320
H	10.41370	-6.28500	-0.43500
H	4.92870	-5.20070	0.22470
H	5.34960	-5.52090	-1.49010
H	4.63980	-6.82040	-0.49610
H	8.35960	-2.99070	4.40770
H	8.25890	-4.73950	4.16390
H	9.83740	-3.92610	4.18750
H	6.77920	-2.56780	2.58280
H	7.05810	-3.15170	0.93330
H	6.61270	-4.29160	2.21860
H	9.40920	-2.08990	0.91270
H	9.07840	-1.46350	2.53890
H	10.53620	-2.40330	2.25090
H	9.17670	-10.79990	-0.59350
H	8.40780	-9.92160	0.73380
H	7.67230	-9.91680	-0.88480
H	11.29900	-9.68490	-0.21560
H	11.50040	-7.93880	-0.22820
H	10.66350	-8.71640	1.12430
H	10.29750	-9.52310	-2.51400
H	8.79230	-8.62640	-2.80100
H	10.30330	-7.75030	-2.52860
H	8.60200	-7.47220	0.86840

66. N1010_Syn60GP

N	10.53400	-5.05010	2.00810
C	9.14260	-4.84720	1.58920
C	9.09980	-4.89030	0.05950
N	9.42120	-6.13360	-0.60580
C	8.60710	-7.30820	-0.28740
C	9.11650	-8.05400	0.95080
O	8.11650	-8.59410	1.67280
C	8.50150	-9.35850	2.83560
O	10.29000	-8.14660	1.26610
O	8.85290	-3.90170	-0.59420
C	8.49670	-8.25350	-1.54880
C	7.76070	-7.46780	-2.65170
C	9.89580	-8.65960	-2.05090
C	7.68460	-9.51910	-1.20850
C	10.94820	-4.49340	3.31980
C	12.37840	-5.00430	3.56320
C	10.96890	-2.95630	3.24190
C	10.03200	-4.95340	4.47640
H	10.76100	-6.04140	1.97660
H	8.80610	-3.85300	1.88340
H	8.43280	-5.57870	2.01570
H	10.41660	-6.33110	-0.52480
H	7.58970	-6.97010	-0.06440
H	9.13720	-10.19770	2.54440
H	9.04100	-8.72590	3.54390
H	7.56720	-9.71300	3.26910
H	7.71330	-8.06570	-3.56870
H	6.73170	-7.23510	-2.35200
H	8.27290	-6.52840	-2.87150
H	9.79790	-9.33420	-2.90850
H	10.46800	-7.78830	-2.38390
H	10.47470	-9.18000	-1.28070
H	8.20890	-10.17610	-0.50680
H	6.71080	-9.27120	-0.77220
H	7.50570	-10.09400	-2.12340
H	12.77280	-4.61450	4.50710
H	12.40270	-6.09950	3.61620
H	13.04000	-4.68740	2.75140
H	11.36670	-2.53510	4.17140
H	11.59940	-2.62790	2.41040
H	9.96930	-2.53420	3.09630
H	10.02010	-6.04740	4.55200
H	10.38140	-4.55530	5.43550
H	9.00100	-4.61050	4.33540

67. N1100_Syn60GP

N	10.20160	-3.76180	1.97110
C	9.18790	-4.76750	1.61770
C	9.09880	-4.88680	0.07260
N	9.42060	-6.13100	-0.61260
C	8.59500	-7.30850	-0.39660
C	9.02740	-8.22310	0.74710
O	8.12400	-9.19890	0.95260
C	8.45680	-10.18390	1.95520
O	10.07010	-8.12270	1.36390
O	8.85440	-3.90770	-0.60050
C	7.80450	-4.41260	2.29380
C	7.36570	-2.96800	1.98470
C	7.97840	-4.56660	3.81760
C	6.70300	-5.38230	1.82380
C	11.61390	-4.13650	2.22770
C	12.27110	-4.94610	1.08580
C	12.36770	-2.80530	2.39950
C	11.70320	-4.94060	3.53750
H	10.15860	-2.99630	1.30410
H	9.50500	-5.73260	2.01870
H	10.39940	-6.35610	-0.44800
H	8.61760	-7.92730	-1.30460
H	7.54860	-7.02880	-0.26110
H	9.38240	-10.69810	1.68730
H	8.57650	-9.70660	2.93030
H	7.61770	-10.87840	1.96580
H	6.41820	-2.75600	2.49380
H	8.10540	-2.24860	2.34810
H	7.22220	-2.80450	0.91360
H	7.04760	-4.30420	4.33340
H	8.23300	-5.59800	4.09120
H	8.77110	-3.90950	4.18380
H	6.96660	-6.42810	2.02200
H	5.77090	-5.17800	2.36220
H	6.48500	-5.26970	0.75550
H	13.34330	-5.07710	1.27070
H	11.83650	-5.94840	1.01410
H	12.15990	-4.43190	0.12380
H	13.41690	-2.98590	2.65390
H	12.34560	-2.21760	1.47340
H	11.91420	-2.20700	3.19580
H	11.28870	-4.36500	4.37050
H	11.16230	-5.88960	3.47000
H	12.74820	-5.17750	3.76510

68. N1110_Syn60GP

N	8.31410	-5.83430	2.25100
C	9.07190	-4.75400	1.61750
C	9.10020	-4.89190	0.06860
N	9.41930	-6.12630	-0.60940
C	8.55750	-7.31180	-0.48110
C	9.27620	-8.38680	0.32950
O	8.42580	-9.22110	0.95590
C	9.02490	-10.30210	1.69810
O	10.48730	-8.50050	0.39500
O	8.85430	-3.90730	-0.59970
C	10.52030	-4.54550	2.22150
C	11.30480	-3.53900	1.35280
C	11.33610	-5.85030	2.33110
C	10.37470	-3.94100	3.63540
C	8.11750	-7.82540	-1.91170
C	7.34380	-6.67670	-2.58860
C	9.34700	-8.18470	-2.76550
C	7.18630	-9.04850	-1.79690
C	6.92480	-5.57360	2.72190
C	6.04340	-5.11500	1.54620
C	6.40000	-6.91570	3.26140
C	6.85310	-4.51630	3.84810
H	8.84240	-6.25940	3.00500
H	8.56890	-3.79110	1.76470
H	10.38510	-6.39600	-0.42980
H	7.65150	-7.05410	0.06620
H	8.19000	-10.85700	2.12510
H	9.61410	-10.94010	1.03490
H	9.67240	-9.90980	2.48580
H	12.25460	-3.29330	1.84000
H	11.53920	-3.93840	0.36080
H	10.74500	-2.60940	1.20820
H	12.31720	-5.62560	2.76470
H	10.86410	-6.59200	2.98470
H	11.50620	-6.33510	1.36750
H	9.82370	-4.59910	4.31440
H	11.36510	-3.77500	4.07320
H	9.85590	-2.97680	3.60620
H	7.04920	-6.97030	-3.60250
H	6.42830	-6.43400	-2.03470
H	7.95450	-5.77290	-2.65060
H	9.02800	-8.49480	-3.76680
H	10.01000	-7.32170	-2.87580
H	9.92450	-9.00740	-2.32960
H	7.70160	-9.93390	-1.41320
H	6.33410	-8.84760	-1.13750
H	6.78960	-9.29930	-2.78690
H	5.01840	-4.94940	1.89350

H	6.39700	-4.17800	1.10530
H	6.00790	-5.87040	0.75570
H	5.36860	-6.81450	3.61490
H	6.43310	-7.68520	2.48500
H	7.00660	-7.26150	4.10820
H	7.23580	-3.54450	3.52080
H	5.81660	-4.36840	4.17150
H	7.43280	-4.83330	4.72200

69. N111'0_Syn60GP

N	10.20690	-3.76010	1.95690
C	9.18870	-4.76960	1.61690
C	9.09910	-4.88780	0.06820
N	9.42110	-6.13310	-0.61040
C	8.53160	-7.31090	-0.46180
C	7.59970	-7.24340	-1.68600
O	6.58680	-6.38450	-1.44080
C	5.72380	-6.08370	-2.55630
O	7.74030	-7.81770	-2.74510
O	8.85370	-3.90460	-0.59840
C	7.82220	-4.40310	2.32290
C	6.72290	-5.41880	1.96240
C	7.34260	-2.98800	1.94400
C	8.05540	-4.45900	3.84650
C	9.30400	-8.65910	-0.31720
C	8.28150	-9.81640	-0.35420
C	10.35860	-8.86960	-1.42350
C	9.99850	-8.68500	1.06260
C	11.62810	-4.10820	2.18640
C	11.75410	-4.96900	3.45600
C	12.30490	-4.83280	0.99950
C	12.34560	-2.76510	2.41900
H	10.14000	-2.99160	1.29520
H	9.50460	-5.73590	2.02170
H	10.37510	-6.38820	-0.37300
H	6.30190	-5.62910	-3.36390
H	5.24180	-6.99380	-2.92060
H	4.98520	-5.38200	-2.16970
H	5.80150	-5.17130	2.50120
H	6.48690	-5.42130	0.89440
H	7.00280	-6.43720	2.25960
H	6.42010	-2.75870	2.48970
H	8.08430	-2.23210	2.21900
H	7.13860	-2.89720	0.87450
H	8.82870	-3.74970	4.14980
H	7.12900	-4.20900	4.37600
H	8.36130	-5.46190	4.16970
H	8.78400	-10.76380	-0.13200
H	7.49380	-9.67710	0.39670
H	7.81230	-9.90730	-1.33630
H	10.85410	-9.83600	-1.27600
H	9.90000	-8.85880	-2.41340
H	11.13980	-8.10140	-1.40530
H	10.75410	-7.89920	1.17120
H	9.27540	-8.57330	1.87900
H	10.51260	-9.64170	1.20220
H	12.80760	-5.18270	3.66580
H	11.32730	-4.44760	4.31750

H	11.24110	-5.93100	3.35320
H	13.38290	-4.93530	1.16670
H	11.90980	-5.84680	0.87460
H	12.16000	-4.28180	0.06370
H	11.88280	-2.22330	3.24920
H	13.40300	-2.92560	2.65260
H	12.29400	-2.13160	1.52510
H	7.89700	-7.21420	0.42630

70. N0000_Syn-60GP

N	7.99640	-5.79040	2.03820
C	9.04980	-4.87310	1.58940
C	9.09940	-4.88850	0.06020
N	9.42160	-6.13530	-0.60660
C	10.71680	-6.72970	-0.32360
C	10.80170	-7.69550	0.85710
O	12.07510	-8.06200	1.08110
C	12.29380	-9.03180	2.12930
O	9.85450	-8.12370	1.48990
O	8.85290	-3.90170	-0.59410
H	7.61810	-5.49840	2.93260
H	8.34530	-6.74150	2.12030
H	10.05020	-5.10540	1.99570
H	8.79990	-3.85320	1.88800
H	8.65980	-6.80190	-0.50020
H	11.46800	-5.94450	-0.19130
H	11.04860	-7.29850	-1.20310
H	13.36990	-9.19880	2.14610
H	11.94850	-8.63700	3.08730
H	11.76050	-9.95800	1.90460

71. N0010_Syn-60GP

N	8.24970	-6.02020	2.08900
C	9.01140	-4.87030	1.58800
C	9.09980	-4.89010	0.05550
N	9.42230	-6.13790	-0.60410
C	10.66670	-6.82900	-0.18340
C	11.82920	-5.96220	-0.69340
O	11.92810	-4.85220	0.07790
C	12.88140	-3.85890	-0.35470
O	12.55810	-6.17330	-1.63720
O	8.85180	-3.89750	-0.59190
C	10.69100	-8.31100	-0.67440
C	12.02840	-8.95060	-0.24220
C	9.54860	-9.08050	0.02890
C	10.51670	-8.43500	-2.20170
H	7.24950	-5.84850	2.03150
H	8.46470	-6.20890	3.06300
H	10.03370	-4.93110	1.97580
H	8.60830	-3.89020	1.87180
H	8.64580	-6.76920	-0.41160
H	10.76060	-6.86270	0.91400
H	13.88910	-4.28010	-0.36230
H	12.62540	-3.50590	-1.35590
H	12.80500	-3.04890	0.36990
H	12.17510	-8.87600	0.84290
H	12.03120	-10.01480	-0.50150
H	12.87960	-8.48210	-0.74140
H	9.58800	-8.96310	1.11740
H	8.55420	-8.76420	-0.30640
H	9.63070	-10.14800	-0.20160
H	9.58820	-7.96290	-2.53590
H	11.34470	-7.96520	-2.73520
H	10.47960	-9.49470	-2.48020

72. N0100_Syn-60GP

N	9.36300	-6.13710	2.20080
C	8.96580	-4.85250	1.60960
C	9.10000	-4.89110	0.06340
N	9.41990	-6.12850	-0.60730
C	10.75500	-6.68650	-0.40510
C	10.67730	-8.16320	-0.06320
O	11.88990	-8.73520	-0.07460
C	11.93980	-10.13830	0.26210
O	9.64520	-8.75780	0.18980
O	8.85400	-3.90590	-0.59660
C	9.73920	-3.65820	2.29180
C	9.62820	-3.80760	3.82680
C	9.10250	-2.30640	1.90870
C	11.22830	-3.66560	1.89680
H	9.11470	-6.16620	3.18410
H	8.88890	-6.91940	1.75730
H	7.89150	-4.63760	1.76240
H	8.71490	-6.84490	-0.43590
H	11.29130	-6.20500	0.42090
H	11.36820	-6.57130	-1.30750
H	11.57470	-10.29530	1.27950
H	11.33130	-10.71680	-0.43610
H	12.99000	-10.41540	0.18210
H	10.18740	-4.66980	4.20360
H	10.04320	-2.91840	4.31330
H	8.58270	-3.89650	4.14870
H	8.04290	-2.27570	2.19040
H	9.61240	-1.49750	2.44450
H	9.16750	-2.11110	0.83820
H	11.36050	-3.47460	0.82630
H	11.76670	-2.87870	2.43670
H	11.69690	-4.62310	2.14590

73. N0110_Syn-60GP

N	9.95040	-3.69450	1.96300
C	9.09690	-4.82360	1.59950
C	9.09970	-4.89000	0.05850
N	9.42170	-6.13570	-0.60550
C	10.76710	-6.67390	-0.32390
C	11.78540	-5.90090	-1.17960
O	12.77180	-5.40210	-0.40110
C	13.79420	-4.65200	-1.08460
O	11.74620	-5.75860	-2.38170
O	8.85240	-3.89980	-0.59360
C	7.62440	-4.82380	2.18000
C	6.87330	-3.52070	1.85190
C	6.84010	-6.02340	1.60890
C	7.71730	-4.98860	3.71120
C	10.84050	-8.21790	-0.59540
C	12.30630	-8.67730	-0.44530
C	9.99890	-8.95140	0.47230
C	10.32750	-8.58430	-2.00170
H	9.94330	-3.51950	2.96280
H	9.65000	-2.85300	1.47870
H	9.57180	-5.73290	1.98750
H	8.72770	-6.82640	-0.32640
H	11.06670	-6.52110	0.72270
H	14.47230	-4.30900	-0.30380
H	14.31920	-5.28810	-1.80190
H	13.35110	-3.80480	-1.61300
H	5.86240	-3.55830	2.27390
H	7.37510	-2.64840	2.28390
H	6.78800	-3.35870	0.77420
H	5.85680	-6.09790	2.08630
H	6.66350	-5.92200	0.53140
H	7.36160	-6.97120	1.79180
H	6.71510	-5.01330	4.15320
H	8.22650	-5.92060	3.98500
H	8.25430	-4.15770	4.18270
H	12.36430	-9.76760	-0.52890
H	12.94570	-8.25480	-1.22680
H	12.72270	-8.39110	0.52710
H	10.08600	-10.03430	0.33440
H	10.34130	-8.71460	1.48650
H	8.93040	-8.71420	0.41000
H	9.27490	-8.31750	-2.13430
H	10.89410	-8.06700	-2.77900
H	10.42420	-9.66510	-2.15630

74. N011'0_Syn-60GP

N	8.30410	-5.99220	2.10170
C	8.96840	-4.78370	1.61200
C	9.09980	-4.89070	0.06910
N	9.41930	-6.12630	-0.60990
C	10.70370	-6.80180	-0.40780
C	10.52000	-8.08950	0.39540
O	11.63800	-8.45100	1.05410
C	11.57370	-9.69030	1.79200
O	9.48330	-8.72450	0.44490
O	8.85470	-3.90860	-0.59970
C	10.22070	-4.24580	2.42790
C	9.65580	-3.51960	3.67080
C	11.01970	-3.22890	1.59030
C	11.15650	-5.36350	2.92440
C	11.40520	-7.06070	-1.80730
C	12.78410	-7.72470	-1.62580
C	11.60230	-5.68570	-2.47540
C	10.51310	-7.94130	-2.70020
H	7.91400	-5.85740	3.02870
H	8.90200	-6.81160	2.12150
H	8.22640	-3.98440	1.69920
H	8.65160	-6.78730	-0.49180
H	12.55870	-9.80810	2.24260
H	10.79960	-9.63580	2.56130
H	11.35300	-10.52170	1.11840
H	10.46930	-3.15460	4.30750
H	9.04250	-2.65830	3.38320
H	9.03740	-4.18630	4.28400
H	11.79280	-2.75790	2.20780
H	11.52120	-3.70170	0.73860
H	10.37390	-2.44070	1.19150
H	11.63320	-5.92190	2.11560
H	11.95970	-4.92540	3.52750
H	10.62870	-6.08060	3.56280
H	13.29880	-7.76210	-2.59210
H	13.41780	-7.16000	-0.93260
H	12.71170	-8.74930	-1.25070
H	12.04720	-5.81440	-3.46830
H	10.65110	-5.15990	-2.58430
H	12.28010	-5.05270	-1.88900
H	10.98640	-8.07810	-3.67880
H	10.34990	-8.93470	-2.26800
H	9.53790	-7.47320	-2.86090
H	11.38490	-6.15440	0.15140

75. N1000_Syn-60GP

N	8.61480	-6.06730	2.20200
C	9.09230	-4.83060	1.59040
C	9.10050	-4.89300	0.05300
N	9.42140	-6.13470	-0.60220
C	10.73020	-6.68410	-0.24780
C	10.79570	-8.13940	-0.66980
O	12.07040	-8.55100	-0.80310
C	12.25570	-9.94370	-1.13160
O	9.82800	-8.85500	-0.82490
O	8.85190	-3.89790	-0.59130
C	8.80820	-6.23870	3.66730
C	8.05330	-7.52310	4.04750
C	8.25350	-5.05230	4.48690
C	10.30610	-6.42760	3.96480
H	7.62180	-6.17210	2.00240
H	10.13040	-4.65710	1.90140
H	8.53100	-3.92490	1.85850
H	8.72120	-6.83990	-0.36610
H	10.94140	-6.67810	0.83490
H	11.52460	-6.12360	-0.74880
H	11.82810	-10.57780	-0.35130
H	11.77810	-10.17320	-2.08650
H	13.33410	-10.08350	-1.19520
H	6.97830	-7.42010	3.85410
H	8.42420	-8.37340	3.46720
H	8.17740	-7.74490	5.11220
H	8.35360	-5.24090	5.56140
H	8.78600	-4.12140	4.26570
H	7.19010	-4.89310	4.27240
H	10.45680	-6.62790	5.03050
H	10.70670	-7.27270	3.39630
H	10.89340	-5.53590	3.72050

76. N1010_Syn-60GP

N	7.66790	-5.62470	1.85110
C	8.91470	-4.89740	1.57750
C	9.09970	-4.88980	0.05760
N	9.42210	-6.13730	-0.60510
C	10.64480	-6.86110	-0.19000
C	11.83140	-5.97980	-0.61090
O	11.85240	-4.86880	0.16260
C	12.82040	-3.85960	-0.19420
O	12.63800	-6.18540	-1.49050
O	8.85210	-3.89850	-0.59300
C	10.66980	-8.31320	-0.76350
C	9.48860	-9.10210	-0.15220
C	10.55450	-8.34430	-2.30110
C	11.97790	-9.00190	-0.31700
C	7.38760	-6.01790	3.25820
C	5.95020	-6.56430	3.26790
C	8.35600	-7.13800	3.67600
C	7.49660	-4.83080	4.23980
H	6.88950	-5.06020	1.51510
H	9.74750	-5.41990	2.05350
H	8.92060	-3.85470	1.92250
H	8.61760	-6.74660	-0.46510
H	10.71600	-6.95830	0.90740
H	13.83250	-4.26420	-0.12090
H	12.63880	-3.51140	-1.21330
H	12.67250	-3.05080	0.52060
H	9.55700	-10.15400	-0.44910
H	9.49870	-9.06320	0.94340
H	8.51200	-8.74330	-0.49680
H	10.51550	-9.38470	-2.64440
H	9.64640	-7.83860	-2.64180
H	11.41010	-7.85730	-2.77230
H	12.85570	-8.52200	-0.75470
H	12.08250	-8.99150	0.77550
H	11.97240	-10.04970	-0.63630
H	5.67440	-6.91130	4.26870
H	5.23140	-5.78890	2.97580
H	5.85240	-7.40140	2.56980
H	8.11390	-7.49190	4.68350
H	8.28230	-7.98330	2.98520
H	9.39680	-6.79770	3.69530
H	6.82190	-4.01790	3.94670
H	7.22810	-5.13980	5.25590
H	8.51450	-4.42880	4.27660

77. N1100_Syn-60GP

N	9.44320	-3.50880	2.12250
C	9.01420	-4.81080	1.61050
C	9.09920	-4.88820	0.06980
N	9.42060	-6.13120	-0.61100
C	10.71050	-6.75600	-0.37190
C	10.80710	-7.73640	0.79520
O	12.09310	-8.04420	1.05250
C	12.32260	-9.04330	2.06980
O	9.86310	-8.22430	1.38370
O	8.85400	-3.90610	-0.59930
C	7.52100	-5.10790	2.08700
C	6.60030	-3.91410	1.76940
C	7.56220	-5.32650	3.61150
C	6.94010	-6.37480	1.42550
C	10.83860	-3.27910	2.56540
C	11.90270	-3.61720	1.49670
C	10.92490	-1.77780	2.89630
C	11.11630	-4.08900	3.84460
H	9.18430	-2.80060	1.44020
H	9.63440	-5.60060	2.04070
H	8.66420	-6.80260	-0.50780
H	11.48710	-5.99480	-0.27320
H	10.98580	-7.33390	-1.26560
H	11.92500	-8.70540	3.02930
H	11.84370	-9.98440	1.79070
H	13.40440	-9.15970	2.12050
H	5.57940	-4.14450	2.09470
H	6.92510	-3.01340	2.29680
H	6.56790	-3.69470	0.69730
H	6.54700	-5.47050	3.99860
H	8.14730	-6.21710	3.87040
H	8.00160	-4.46130	4.11400
H	7.59450	-7.24190	1.55420
H	5.97380	-6.61240	1.88390
H	6.74480	-6.22720	0.35560
H	12.90170	-3.31580	1.83150
H	11.94440	-4.69360	1.30340
H	11.69120	-3.10200	0.55350
H	11.91580	-1.52200	3.28520
H	10.74960	-1.16800	2.00180
H	10.17490	-1.50820	3.64600
H	10.40530	-3.82000	4.63110
H	11.03860	-5.16800	3.67290
H	12.12980	-3.88820	4.20880

78. N1110_Syn-60GP

N	8.46580	-6.06920	2.18730
C	8.90420	-4.79230	1.61840
C	9.09960	-4.88970	0.06800
N	9.42010	-6.12910	-0.60970
C	10.63740	-6.86840	-0.26210
C	11.84380	-6.14940	-0.88850
O	12.88020	-6.09000	-0.01120
C	14.09050	-5.48880	-0.51090
O	11.91100	-5.70720	-2.01260
O	8.85420	-3.90670	-0.59880
C	10.07570	-4.09470	2.42780
C	11.22630	-5.04540	2.81760
C	9.46870	-3.52100	3.72900
C	10.66010	-2.91880	1.61870
C	10.56150	-8.35440	-0.78570
C	9.47680	-9.11130	0.01070
C	10.23460	-8.41960	-2.29050
C	11.91260	-9.05310	-0.52340
C	7.01490	-6.30620	2.42400
C	6.24710	-6.24770	1.09100
C	6.90330	-7.72330	3.01260
C	6.38170	-5.30300	3.41640
H	8.97440	-6.28420	3.03750
H	8.09170	-4.05650	1.66850
H	8.62240	-6.74300	-0.46570
H	10.80840	-6.92970	0.81950
H	14.79240	-5.51240	0.32250
H	14.47800	-6.06060	-1.35780
H	13.90160	-4.46170	-0.83130
H	11.94160	-4.49860	3.44240
H	10.89030	-5.90450	3.41000
H	11.77500	-5.42140	1.95340
H	10.24630	-3.01660	4.31250
H	8.68370	-2.78780	3.51480
H	9.03720	-4.30100	4.36410
H	9.88080	-2.22220	1.29670
H	11.37660	-2.37030	2.24030
H	11.18680	-3.25500	0.72120
H	9.46890	-10.16490	-0.28930
H	9.66260	-9.06360	1.08870
H	8.46870	-8.72460	-0.17330
H	10.19560	-9.46730	-2.61020
H	9.27040	-7.95680	-2.51610
H	10.98880	-7.90310	-2.88910
H	12.72020	-8.62580	-1.12580
H	12.20600	-8.98670	0.53020
H	11.83550	-10.11350	-0.78600
H	5.18300	-6.44000	1.26170

H	6.32660	-5.26760	0.60990
H	6.61030	-7.00830	0.39220
H	5.85720	-7.98120	3.20730
H	7.32280	-8.46330	2.32520
H	7.44400	-7.79690	3.96440
H	6.44730	-4.27170	3.05620
H	5.32030	-5.52950	3.56660
H	6.87520	-5.35370	4.39290

79. N111'0_Syn-60GP

N	9.40270	-3.48830	2.11150
C	9.03260	-4.81280	1.61110
C	9.09970	-4.89000	0.06700
N	9.42050	-6.13070	-0.60920
C	10.74860	-6.71900	-0.38540
C	10.70460	-7.80100	0.69420
O	11.78570	-7.76590	1.49970
C	11.85990	-8.79050	2.51470
O	9.80160	-8.60610	0.82410
O	8.85370	-3.90480	-0.59830
C	7.56940	-5.18600	2.12200
C	7.04660	-6.49820	1.50270
C	6.57750	-4.05370	1.79310
C	7.64600	-5.36470	3.65040
C	11.34750	-7.26360	-1.74050
C	12.71190	-7.93440	-1.48570
C	10.38920	-8.27050	-2.40320
C	11.54540	-6.05310	-2.67320
C	10.78560	-3.18530	2.55380
C	11.10950	-3.97800	3.83270
C	11.86620	-3.46430	1.48470
C	10.78900	-1.68110	2.88380
H	9.11640	-2.80230	1.41710
H	9.70940	-5.56550	2.02770
H	8.70100	-6.83100	-0.44500
H	10.99690	-8.72880	3.18110
H	11.88740	-9.77980	2.05210
H	12.78350	-8.59240	3.05730
H	6.10860	-6.78140	1.99270
H	6.81470	-6.38870	0.43620
H	7.74610	-7.33090	1.62800
H	5.57680	-4.33020	2.14380
H	6.86270	-3.12280	2.28990
H	6.51350	-3.86490	0.71640
H	8.04130	-4.46390	4.12570
H	6.64730	-5.56020	4.05720
H	8.28670	-6.21270	3.92210
H	13.17520	-8.20020	-2.44180
H	13.39950	-7.26630	-0.95500
H	12.61990	-8.85570	-0.90040
H	10.82510	-8.63110	-3.34160
H	10.19940	-9.13910	-1.76480
H	9.43000	-7.80140	-2.64140
H	10.60560	-5.51450	-2.81830
H	12.28310	-5.35220	-2.26370
H	11.91090	-6.38780	-3.65040
H	12.10950	-3.71730	4.19610
H	10.38510	-3.75160	4.62040

H	11.09880	-5.05930	3.65900
H	12.84470	-3.09860	1.81640
H	11.97480	-4.53700	1.29890
H	11.62160	-2.96910	0.53890
H	10.02630	-1.45230	3.63430
H	11.76500	-1.37070	3.27070
H	10.57890	-1.08270	1.98900
H	11.42890	-5.93680	-0.04220

80. N0000_Anti120WP

N	8.78480	-3.69720	2.24560
C	9.12870	-4.94120	1.56030
C	9.10040	-4.89220	0.04650
N	9.42570	-6.15170	-0.59960
C	8.31310	-6.61230	-1.45770
C	8.72960	-7.81870	-2.27510
O	7.68230	-8.33190	-2.93230
C	7.92940	-9.45380	-3.82630
O	9.86800	-8.25300	-2.35500
O	8.84770	-3.88160	-0.58740
H	7.83850	-3.40990	1.98590
H	9.39480	-2.94230	1.92380
H	8.45890	-5.75400	1.87870
H	10.13730	-5.27540	1.84580
H	10.23930	-5.96800	-1.20090
H	7.96440	-5.84580	-2.16640
H	7.45490	-6.89150	-0.83410
H	6.95720	-9.69160	-4.25800
H	8.63580	-9.15810	-4.60480
H	8.31980	-10.30280	-3.26190

81. N0010_Anti120WP

N	8.95260	-3.63440	2.20680
C	9.27820	-4.89460	1.54000
C	9.10080	-4.89330	0.03350
N	9.42770	-6.15970	-0.59360
C	8.41600	-6.89410	-1.41570
C	9.17460	-8.14160	-1.88490
O	8.46400	-9.26370	-1.72970
C	9.03850	-10.50050	-2.23970
O	10.30090	-8.11530	-2.36110
O	8.84530	-3.87250	-0.58040
C	7.68720	-6.17850	-2.63260
C	6.51930	-5.31690	-2.09890
C	8.66080	-5.32850	-3.46970
C	7.05710	-7.25310	-3.55130
H	7.97350	-3.39830	2.02760
H	9.49040	-2.87410	1.78410
H	8.67980	-5.71700	1.95730
H	10.32550	-5.17380	1.72630
H	10.23760	-5.97000	-1.19630
H	7.63130	-7.23870	-0.73330
H	9.23230	-10.40590	-3.31050
H	9.96370	-10.73170	-1.70770
H	8.28360	-11.26410	-2.05170
H	5.98480	-4.86280	-2.94110
H	5.80370	-5.93700	-1.54570
H	6.84950	-4.51020	-1.44580
H	9.08210	-4.50450	-2.89030
H	9.48380	-5.93700	-3.86120
H	8.12590	-4.89910	-4.32540
H	6.45620	-6.75410	-4.31960
H	7.81120	-7.85700	-4.06880
H	6.39670	-7.92520	-2.99300

82. N0100_Anti120WP

N	10.68200	-4.80420	1.88580
C	9.23250	-4.92530	1.58570
C	9.09900	-4.88720	0.05800
N	9.42610	-6.15300	-0.60610
C	8.32380	-6.61320	-1.47960
C	7.17450	-7.22070	-0.67900
O	6.04940	-7.25070	-1.40980
C	4.86040	-7.82820	-0.79840
O	7.24730	-7.63610	0.46550
O	8.84870	-3.88530	-0.59240
C	8.34460	-3.91670	2.38910
C	8.85100	-2.46240	2.29970
C	8.34720	-4.35210	3.87190
C	6.88540	-3.98630	1.88250
H	11.18940	-5.61290	1.52150
H	11.08780	-3.97660	1.44410
H	8.93710	-5.93440	1.89120
H	10.22200	-5.95210	-1.22450
H	8.70790	-7.40350	-2.13730
H	7.91850	-5.82260	-2.12400
H	4.08570	-7.75810	-1.56170
H	5.04320	-8.87020	-0.52780
H	4.57860	-7.25250	0.08580
H	9.85230	-2.35690	2.73200
H	8.87670	-2.10430	1.26680
H	8.18090	-1.80590	2.86830
H	7.73790	-3.65890	4.46430
H	7.91970	-5.35510	3.99060
H	9.35750	-4.35670	4.29170
H	6.50370	-5.01440	1.90530
H	6.24080	-3.38370	2.53350
H	6.78090	-3.59980	0.86460

83. N0110_Anti120WP

N	10.79110	-4.87100	1.76260
C	9.32130	-4.93260	1.55840
C	9.10040	-4.89220	0.04010
N	9.42710	-6.15720	-0.59680
C	8.37770	-6.83000	-1.40750
C	8.79760	-8.30730	-1.38000
O	7.82280	-9.09970	-0.91090
C	8.09190	-10.52750	-0.83000
O	9.89340	-8.72370	-1.72790
O	8.84630	-3.87610	-0.58380
C	8.52090	-3.89530	2.40770
C	9.03680	-2.45100	2.24920
C	7.02870	-3.95980	2.00980
C	8.63390	-4.30650	3.89200
C	8.07470	-6.33280	-2.88270
C	7.41020	-7.48200	-3.67800
C	7.06680	-5.16270	-2.84840
C	9.36290	-5.89200	-3.60160
H	11.19900	-4.07350	1.27040
H	11.23230	-5.70870	1.37690
H	9.00530	-5.92880	1.88910
H	10.27140	-6.02470	-1.15950
H	7.44140	-6.76550	-0.84340
H	7.17480	-10.96860	-0.43910
H	8.31760	-10.92280	-1.82280
H	8.92840	-10.71200	-0.15240
H	10.07710	-2.35640	2.58080
H	8.96950	-2.11000	1.21300
H	8.43410	-1.77630	2.86940
H	6.43090	-3.35720	2.70380
H	6.85500	-3.57480	1.00100
H	6.65080	-4.98920	2.05610
H	8.07850	-3.60020	4.52020
H	8.21140	-5.30500	4.05800
H	9.67460	-4.31520	4.22990
H	7.10770	-7.11260	-4.66440
H	8.08890	-8.32700	-3.84020
H	6.51080	-7.85110	-3.17120
H	6.82720	-4.85590	-3.87370
H	6.12990	-5.46550	-2.36370
H	7.46430	-4.29650	-2.31900
H	9.80740	-5.01080	-3.12660
H	10.11070	-6.69260	-3.62140
H	9.13240	-5.62370	-4.63910

84. N011'0_Anti120WP

N	10.80990	-4.97930	1.74950
C	9.33210	-4.92480	1.56200
C	9.09970	-4.88940	0.04540
N	9.42770	-6.15940	-0.59990
C	8.42870	-6.87520	-1.43440
C	7.19460	-7.14160	-0.57680
O	6.53250	-8.24680	-0.94430
C	5.29210	-8.54180	-0.24130
O	6.83090	-6.41690	0.33600
O	8.84650	-3.87670	-0.58600
C	8.66340	-3.80130	2.41820
C	9.30750	-2.41580	2.20240
C	8.80930	-4.19160	3.90610
C	7.15380	-3.71140	2.10400
C	8.04610	-6.27640	-2.86290
C	7.64290	-7.44900	-3.78460
C	9.29010	-5.59750	-3.47110
C	6.88210	-5.26420	-2.80670
H	11.26680	-4.19550	1.27760
H	11.17860	-5.82860	1.31710
H	8.93840	-5.88400	1.91240
H	10.27300	-6.01440	-1.15680
H	5.49090	-8.70120	0.82080
H	4.58380	-7.72030	-0.37040
H	4.91250	-9.45320	-0.70300
H	8.80350	-1.67620	2.83660
H	10.36810	-2.41090	2.47780
H	9.21540	-2.08810	1.16370
H	9.85930	-4.26650	4.20310
H	8.32890	-3.43660	4.53960
H	8.32560	-5.15480	4.10970
H	6.66010	-4.67690	2.24870
H	6.68330	-2.98530	2.77780
H	6.96760	-3.38830	1.07650
H	6.76380	-7.98170	-3.41060
H	7.40660	-7.06540	-4.78440
H	8.46350	-8.17050	-3.88380
H	10.15340	-6.27540	-3.48670
H	9.07660	-5.31060	-4.50710
H	9.56550	-4.69320	-2.92240
H	7.10250	-4.42880	-2.13910
H	6.70350	-4.85890	-3.81000
H	5.94790	-5.73470	-2.47720
H	8.89090	-7.84750	-1.63880

85. N1000_Anti120WP

N	8.80680	-3.74550	2.28950
C	9.18460	-4.95960	1.56590
C	9.09920	-4.88770	0.05160
N	9.42640	-6.15430	-0.60310
C	8.32990	-6.68270	-1.44410
C	7.30090	-7.46520	-0.63160
O	6.22730	-7.75770	-1.38380
C	5.15610	-8.52560	-0.76340
O	7.41600	-7.79570	0.53680
O	8.84820	-3.88340	-0.58900
C	7.38450	-3.61550	2.75190
C	6.42730	-3.57610	1.54770
C	7.29720	-2.27650	3.50620
C	6.96280	-4.75400	3.71020
H	9.40270	-3.68260	3.11700
H	8.63360	-5.86510	1.86100
H	10.23800	-5.19780	1.77670
H	10.20370	-5.93680	-1.23930
H	8.75610	-7.38010	-2.17550
H	7.80210	-5.90740	-2.01620
H	4.74150	-7.97290	0.08240
H	4.40540	-8.65020	-1.54400
H	5.53130	-9.49570	-0.43130
H	6.41450	-4.52170	0.99520
H	5.40510	-3.39800	1.90010
H	6.69960	-2.77550	0.85320
H	7.55570	-1.43940	2.84840
H	6.28060	-2.11680	3.88250
H	7.97810	-2.26440	4.36610
H	7.61990	-4.78980	4.58740
H	5.93810	-4.58750	4.06290
H	6.98080	-5.73630	3.22610

86. N1010_Anti120WP

N	8.91220	-3.74600	2.27700
C	9.28950	-4.95200	1.54310
C	9.10010	-4.89070	0.03450
N	9.42820	-6.16160	-0.59470
C	8.42700	-6.87910	-1.43850
C	9.07480	-8.25090	-1.67100
O	8.24110	-9.26350	-1.40250
C	8.71870	-10.61570	-1.65400
O	10.22300	-8.40870	-2.06260
O	8.84550	-3.87310	-0.58030
C	7.91400	-6.25180	-2.80550
C	9.04880	-5.54670	-3.56880
C	7.33190	-7.37710	-3.69580
C	6.76120	-5.26220	-2.52440
C	7.50760	-3.66220	2.79870
C	7.20430	-4.76200	3.84280
C	7.37580	-2.28320	3.46820
C	6.49080	-3.76270	1.64790
H	9.53960	-3.65880	3.07900
H	8.79970	-5.87610	1.89160
H	10.36490	-5.14380	1.67590
H	10.26570	-5.99060	-1.16240
H	7.54440	-7.05390	-0.81450
H	9.57410	-10.83660	-1.01170
H	7.87660	-11.26480	-1.41170
H	8.99800	-10.72370	-2.70460
H	8.67160	-5.18430	-4.53240
H	9.43140	-4.68490	-3.01510
H	9.88100	-6.22970	-3.77220
H	6.55070	-7.94060	-3.17400
H	8.09850	-8.08220	-4.03640
H	6.88160	-6.93120	-4.58970
H	5.93500	-5.76460	-2.00630
H	7.07950	-4.41090	-1.92260
H	6.37090	-4.87480	-3.47320
H	6.18860	-4.64100	4.23660
H	7.26750	-5.76770	3.41240
H	7.90230	-4.70270	4.68660
H	8.10290	-2.17030	4.28190
H	7.54270	-1.48070	2.74160
H	6.37370	-2.15910	3.89420
H	6.50100	-4.74990	1.17200
H	5.48020	-3.61010	2.04390
H	6.68100	-3.00600	0.88150

87. N1100_Anti120WP

N	10.21770	-3.85550	2.01370
C	9.22760	-4.85850	1.59690
C	9.09950	-4.88890	0.05850
N	9.42530	-6.14970	-0.60590
C	8.29930	-6.72510	-1.37310
C	8.78360	-7.91640	-2.18150
O	7.76490	-8.70250	-2.54760
C	8.07080	-9.86230	-3.37390
O	9.94840	-8.12220	-2.48470
O	8.84910	-3.88680	-0.59320
C	7.84680	-4.63590	2.33200
C	8.07060	-4.91070	3.83250
C	6.79480	-5.62890	1.79990
C	7.32960	-3.19560	2.16140
C	11.63920	-4.24040	2.22040
C	12.38150	-2.93480	2.55830
C	11.75270	-5.20180	3.41900
C	12.28720	-4.88680	0.97600
H	10.16200	-3.03140	1.41770
H	9.58130	-5.84720	1.89700
H	10.14860	-5.90940	-1.29510
H	7.84450	-6.01500	-2.08360
H	7.51010	-7.06260	-0.69560
H	7.11160	-10.35110	-3.54380
H	8.51410	-9.54070	-4.31840
H	8.75370	-10.52720	-2.84140
H	7.14140	-4.73460	4.38720
H	8.37470	-5.94970	4.00740
H	8.84330	-4.25320	4.23910
H	5.87950	-5.56010	2.39940
H	6.51360	-5.41400	0.76250
H	7.15190	-6.66440	1.86110
H	7.13070	-2.95390	1.11330
H	6.39360	-3.07310	2.71980
H	8.05000	-2.47150	2.55430
H	13.44120	-3.13740	2.74760
H	12.31790	-2.22040	1.72790
H	11.95180	-2.46640	3.45040
H	12.80650	-5.43420	3.61020
H	11.32950	-4.74700	4.32040
H	11.23840	-6.15220	3.23860
H	12.21870	-4.21740	0.11030
H	13.34750	-5.09960	1.15590
H	11.79890	-5.83370	0.72230

88. N1110_Anti120WP

N	10.18740	-3.70740	1.94320
C	9.32290	-4.83190	1.57600
C	9.10000	-4.89050	0.04700
N	9.42680	-6.15590	-0.60030
C	8.42660	-6.94120	-1.38380
C	9.20290	-8.21670	-1.74030
O	8.54760	-9.32920	-1.38800
C	9.16720	-10.60600	-1.71310
O	10.29910	-8.22240	-2.28280
O	8.84710	-3.87910	-0.58720
C	7.96010	-4.78710	2.37610
C	8.27890	-5.02790	3.86470
C	7.01940	-5.90600	1.88560
C	7.25870	-3.42350	2.22690
C	7.72630	-6.31980	-2.66230
C	7.11350	-7.47120	-3.49570
C	6.55780	-5.40690	-2.22790
C	8.71620	-5.53370	-3.54070
C	11.65890	-3.90540	2.01960
C	11.99670	-4.83930	3.19750
C	12.27320	-4.47190	0.71910
C	12.25470	-2.51370	2.29690
H	9.97800	-2.90360	1.35370
H	9.81190	-5.76730	1.85530
H	10.19000	-5.93210	-1.24780
H	7.63120	-7.24170	-0.69670
H	8.46570	-11.36090	-1.35740
H	9.30770	-10.68780	-2.79340
H	10.12730	-10.69650	-1.19990
H	8.71870	-6.02070	4.02130
H	8.97950	-4.27710	4.23990
H	7.35850	-4.97070	4.45820
H	6.13020	-5.95470	2.52490
H	6.66450	-5.72690	0.86410
H	7.50760	-6.88790	1.91920
H	7.00150	-3.20620	1.18520
H	6.32910	-3.42240	2.80910
H	7.89070	-2.61270	2.60360
H	6.52460	-7.04850	-4.31730
H	7.87820	-8.11740	-3.94170
H	6.44440	-8.09390	-2.89080
H	6.03440	-5.03610	-3.11730
H	5.82930	-5.96160	-1.62340
H	6.89470	-4.54090	-1.65750
H	9.11760	-4.66410	-3.01300
H	9.55200	-6.16200	-3.86660
H	8.20030	-5.17150	-4.43770
H	13.08380	-4.93080	3.30140

H	11.59060	-4.44130	4.13310
H	11.59790	-5.84930	3.05150
H	13.36430	-4.53700	0.80230
H	11.90170	-5.48130	0.51040
H	12.03800	-3.82850	-0.13710
H	11.84180	-2.09550	3.22120
H	13.34340	-2.57710	2.39850
H	12.03230	-1.82130	1.47520

89. N111'0_Anti120WP

N	10.20800	-3.88740	2.02110
C	9.20250	-4.87320	1.59330
C	9.10030	-4.89200	0.05200
N	9.42550	-6.15080	-0.60210
C	8.32470	-6.75530	-1.38960
C	8.27900	-6.15190	-2.80110
O	7.02920	-5.92350	-3.22060
C	6.85420	-5.40070	-4.56820
O	9.27260	-5.91260	-3.47450
O	8.84800	-3.88260	-0.59040
C	7.82490	-4.63210	2.32870
C	8.04770	-4.92360	3.82760
C	6.75000	-5.60120	1.79880
C	7.32980	-3.18290	2.16750
C	8.43990	-8.32960	-1.41520
C	8.24780	-8.84500	0.02640
C	7.32380	-8.92450	-2.30070
C	9.81440	-8.78960	-1.93870
C	11.62510	-4.29580	2.21570
C	12.25340	-4.95780	0.96890
C	12.39050	-3.00100	2.54280
C	11.73240	-5.25570	3.41590
H	10.16460	-3.06080	1.42740
H	9.54190	-5.86740	1.88970
H	10.19680	-5.95540	-1.24840
H	7.35400	-4.43350	-4.66030
H	7.25980	-6.10640	-5.29670
H	5.77650	-5.29290	-4.69460
H	7.12110	-4.74500	4.38630
H	8.34150	-5.96830	3.99110
H	8.82880	-4.27920	4.23970
H	5.83740	-5.50970	2.39930
H	6.47160	-5.38190	0.76120
H	7.08110	-6.64400	1.86030
H	7.13470	-2.93400	1.12000
H	6.39510	-3.05120	2.72620
H	8.06070	-2.47110	2.56420
H	8.36930	-9.93430	0.04990
H	8.98330	-8.40280	0.70490
H	7.24340	-8.61490	0.40220
H	7.33230	-10.01710	-2.21000
H	6.33240	-8.57010	-1.99220
H	7.45950	-8.68130	-3.36100
H	9.98970	-8.46280	-2.96930
H	10.62940	-8.41270	-1.31080
H	9.86110	-9.88520	-1.92190
H	13.31320	-5.18000	1.14120
H	11.75350	-5.90150	0.72620

H	12.18490	-4.29320	0.09910
H	13.44770	-3.21910	2.72880
H	12.33400	-2.29110	1.70820
H	11.97190	-2.52060	3.43370
H	11.20110	-6.19760	3.24080
H	12.78360	-5.50480	3.60010
H	11.32270	-4.79240	4.31910
H	7.37300	-6.51850	-0.90730

90. N0000_Anti-120WP

N	8.79500	-3.69240	2.24420
C	9.09860	-4.94790	1.56030
C	9.10040	-4.89210	0.04640
N	9.42580	-6.15200	-0.59960
C	10.60690	-6.01070	-1.47770
C	10.82200	-7.26650	-2.29830
O	11.96010	-7.18580	-2.99880
C	12.28030	-8.28530	-3.89740
O	10.05580	-8.21600	-2.34460
O	8.84770	-3.88150	-0.58730
H	7.89110	-3.33360	1.92870
H	9.47700	-2.97950	1.97600
H	8.38230	-5.73100	1.84970
H	10.08070	-5.33050	1.87630
H	8.61510	-6.40260	-1.18020
H	11.50240	-5.83630	-0.86800
H	10.52730	-5.17080	-2.18400
H	13.21470	-7.99540	-4.37750
H	12.40870	-9.20750	-3.32730
H	11.48610	-8.40310	-4.63720

91. N0010_Anti-120WP

N	8.86360	-3.66490	2.22640
C	9.10620	-4.94220	1.55720
C	9.10080	-4.89390	0.04330
N	9.42580	-6.15220	-0.59770
C	10.63490	-6.01650	-1.45240
C	10.32690	-5.19070	-2.71210
O	11.21960	-4.20790	-2.89310
C	11.03260	-3.32350	-4.03180
O	9.38320	-5.39570	-3.46230
O	8.84720	-3.87950	-0.58600
C	11.28210	-7.41080	-1.77720
C	11.82470	-8.00890	-0.46260
C	10.26590	-8.38530	-2.40330
C	12.46590	-7.20670	-2.74710
H	7.98730	-3.26000	1.88890
H	9.58910	-2.99760	1.95290
H	8.35690	-5.69090	1.85430
H	10.07100	-5.36290	1.87430
H	8.62420	-6.41330	-1.18410
H	11.37770	-5.45210	-0.87780
H	11.08130	-3.89240	-4.96280
H	10.06960	-2.81390	-3.95020
H	11.85290	-2.60770	-3.97370
H	12.27760	-8.98720	-0.66040
H	12.59560	-7.36630	-0.02060
H	11.02800	-8.14410	0.27380
H	9.43360	-8.59690	-1.72300
H	9.85610	-7.99650	-3.34020
H	10.76140	-9.33840	-2.62140
H	12.98820	-8.15900	-2.89320
H	12.13900	-6.85610	-3.73280
H	13.18790	-6.48470	-2.34830

92. N0100_Anti-120WP

N	9.70370	-3.62200	2.10190
C	9.17790	-4.89710	1.58510
C	9.10040	-4.89230	0.04950
N	9.42550	-6.15070	-0.60090
C	10.52800	-5.99620	-1.56940
C	10.83280	-7.33070	-2.22290
O	12.09080	-7.37600	-2.67770
C	12.50820	-8.57720	-3.38630
O	10.02830	-8.24150	-2.34830
O	8.84800	-3.88250	-0.58910
C	7.80240	-5.29070	2.26110
C	6.79410	-4.12470	2.20690
C	8.07670	-5.66080	3.73330
C	7.18230	-6.51860	1.56040
H	10.65440	-3.48520	1.75090
H	9.16190	-2.84320	1.72320
H	9.89300	-5.68640	1.84540
H	8.59190	-6.48570	-1.09480
H	11.42350	-5.63960	-1.05090
H	10.31240	-5.28190	-2.37970
H	13.55540	-8.40760	-3.63850
H	12.40310	-9.44810	-2.73540
H	11.90760	-8.70380	-4.29020
H	7.13490	-3.25910	2.78490
H	6.59900	-3.79930	1.17890
H	5.83950	-4.44900	2.63870
H	7.13600	-5.91180	4.23770
H	8.73730	-6.53280	3.80430
H	8.54160	-4.83300	4.27610
H	7.88560	-7.35620	1.51630
H	6.29610	-6.84980	2.11410
H	6.85430	-6.28970	0.53900

93. N0110_Anti-120WP

N	9.57070	-3.60850	2.12200
C	9.15130	-4.91280	1.57920
C	9.10120	-4.89510	0.04470
N	9.42530	-6.15000	-0.59800
C	10.59640	-6.04810	-1.49780
C	10.21040	-5.43130	-2.84870
O	11.09100	-4.50530	-3.24970
C	10.84680	-3.84470	-4.52190
O	9.21710	-5.74370	-3.49080
O	8.84740	-3.88040	-0.58720
C	7.80340	-5.42390	2.23200
C	6.70270	-4.34800	2.14340
C	7.29760	-6.71050	1.54360
C	8.08600	-5.75460	3.71270
C	11.31520	-7.44300	-1.63670
C	12.52500	-7.31120	-2.58490
C	11.82510	-7.85240	-0.23860
C	10.35830	-8.52900	-2.16550
H	8.99070	-2.86700	1.72510
H	10.51920	-3.40520	1.79770
H	9.92110	-5.64750	1.84380
H	8.61080	-6.45770	-1.13770
H	11.32060	-5.37000	-1.03320
H	11.66300	-3.13060	-4.63300
H	10.86080	-4.57680	-5.33240
H	9.88430	-3.32910	-4.49470
H	6.96390	-3.44550	2.70650
H	6.49690	-4.06110	1.10560
H	5.77190	-4.74230	2.56870
H	6.44360	-7.10780	2.10450
H	6.95120	-6.52720	0.51970
H	8.07190	-7.48330	1.50920
H	7.16230	-6.08070	4.20530
H	8.81690	-6.56750	3.80050
H	8.47280	-4.88620	4.25290
H	13.08680	-8.25260	-2.59370
H	12.22430	-7.09740	-3.61670
H	13.20510	-6.51680	-2.25510
H	12.33020	-8.82350	-0.29800
H	12.54500	-7.12300	0.15330
H	10.99880	-7.93450	0.47250
H	9.49890	-8.66750	-1.50070
H	9.98680	-8.29220	-3.16740
H	10.88960	-9.48650	-2.22150

94. N011'0_Anti-120WP

N	9.36910	-3.68420	2.19010
C	8.94650	-4.95290	1.56320
C	9.10100	-4.89450	0.03770
N	9.42680	-6.15600	-0.59510
C	10.66410	-6.30600	-1.41590
C	10.65150	-7.80230	-1.76310
O	11.78030	-8.42300	-1.39880
C	11.89480	-9.84310	-1.70010
O	9.71370	-8.37100	-2.30380
O	8.84600	-3.87500	-0.58310
C	7.48610	-5.37130	2.00830
C	6.47250	-4.23740	1.75380
C	7.53250	-5.69050	3.51760
C	7.01280	-6.63930	1.26530
C	10.91850	-5.41180	-2.69700
C	12.01590	-6.08790	-3.55460
C	11.47170	-4.03400	-2.26840
C	9.64670	-5.23780	-3.54660
H	8.91580	-2.90150	1.71460
H	10.37000	-3.55300	2.02690
H	9.61910	-5.74350	1.91720
H	8.63290	-6.38110	-1.20420
H	11.10460	-10.39840	-1.18990
H	11.83030	-10.00140	-2.77900
H	12.87640	-10.13480	-1.32590
H	5.47210	-4.56920	2.05720
H	6.70760	-3.33710	2.33140
H	6.42370	-3.96470	0.69410
H	7.87570	-4.83060	4.09900
H	6.53190	-5.96680	3.87080
H	8.20460	-6.53310	3.72000
H	7.74300	-7.45080	1.34500
H	6.06910	-6.98430	1.70320
H	6.82330	-6.45370	0.20190
H	11.67860	-7.03180	-3.99820
H	12.29260	-5.42030	-4.37830
H	12.91990	-6.28480	-2.96650
H	12.38470	-4.14820	-1.67080
H	11.72910	-3.44970	-3.15950
H	10.75060	-3.45700	-1.69040
H	8.86420	-4.70930	-2.99540
H	9.88270	-4.64930	-4.44110
H	9.24960	-6.20390	-3.87630
H	11.51570	-6.14880	-0.74480

95. N1000_Anti-120WP

N	8.71950	-3.75520	2.30530
C	9.00370	-4.97990	1.56310
C	9.09960	-4.88930	0.05000
N	9.42640	-6.15430	-0.60200
C	10.65060	-6.07190	-1.42850
C	11.91930	-6.12100	-0.58150
O	12.99510	-5.81050	-1.32230
C	14.29460	-5.82470	-0.66440
O	11.97340	-6.40140	0.60510
O	8.84780	-3.88190	-0.58850
C	9.86340	-2.90740	2.77480
C	10.70630	-3.68700	3.80190
C	9.23150	-1.68920	3.47400
C	10.77390	-2.41120	1.62950
H	8.08980	-3.17090	1.75280
H	8.20940	-5.72530	1.73820
H	9.91400	-5.46660	1.92550
H	8.64780	-6.34730	-1.24570
H	10.69330	-5.17520	-2.06170
H	10.67600	-6.93940	-2.09970
H	14.51550	-6.82660	-0.29030
H	15.00700	-5.53510	-1.43710
H	14.30660	-5.10480	0.15710
H	11.22260	-4.54560	3.35890
H	11.47800	-3.02870	4.21680
H	10.08410	-4.04550	4.62950
H	8.62400	-2.00200	4.33040
H	10.01210	-1.01130	3.83720
H	8.59210	-1.12440	2.78400
H	10.20040	-1.83610	0.89480
H	11.56590	-1.76630	2.02910
H	11.26330	-3.24020	1.10780

96. N1010_Anti-120WP

N	8.82510	-3.71830	2.20830
C	9.15330	-4.97410	1.54140
C	9.10120	-4.89520	0.03880
N	9.42630	-6.15390	-0.59540
C	10.66300	-6.08520	-1.42060
C	10.29510	-5.84070	-2.88830
O	11.18570	-5.07060	-3.51980
C	10.97440	-4.81770	-4.93830
O	9.29770	-6.30370	-3.42580
O	8.84640	-3.87630	-0.58390
C	11.56560	-7.36490	-1.21890
C	10.79550	-8.65590	-1.55460
C	12.82970	-7.27280	-2.10070
C	12.00960	-7.39930	0.25880
C	9.07080	-3.67860	3.68510
C	10.53580	-4.00360	4.06070
C	8.74470	-2.24520	4.13750
C	8.12400	-4.66780	4.38780
H	9.37900	-2.97480	1.77580
H	8.46210	-5.77700	1.83340
H	10.15940	-5.36550	1.77020
H	8.63820	-6.41930	-1.19620
H	11.26050	-5.22710	-1.09330
H	10.02810	-4.29200	-5.08620
H	11.81490	-4.19390	-5.24350
H	10.97310	-5.76080	-5.48860
H	11.43510	-9.52510	-1.36190
H	9.89710	-8.75960	-0.93660
H	10.49690	-8.68900	-2.60820
H	13.37300	-6.33670	-1.92590
H	12.60030	-7.34130	-3.16940
H	13.50250	-8.10230	-1.85400
H	12.59110	-6.50650	0.52070
H	11.15160	-7.46270	0.93360
H	12.64520	-8.27450	0.43410
H	10.68660	-3.86730	5.13790
H	10.80230	-5.03960	3.82540
H	11.23220	-3.33840	3.53580
H	9.40120	-1.51910	3.64150
H	7.70510	-1.98960	3.90380
H	8.88920	-2.14450	5.21870
H	8.34410	-5.70670	4.11970
H	8.23580	-4.58080	5.47400
H	7.07900	-4.45660	4.13410

97. N1100_Anti-120WP

N	8.75450	-3.57790	2.11200
C	8.94420	-4.93410	1.58540
C	9.10150	-4.89660	0.05440
N	9.42270	-6.13970	-0.60190
C	10.73500	-6.14290	-1.27350
C	10.93550	-7.47180	-1.98370
O	12.23030	-7.70430	-2.23160
C	12.56880	-8.91960	-2.95960
O	10.03210	-8.22240	-2.31530
O	8.84970	-3.88910	-0.59310
C	7.78010	-5.93170	2.05180
C	7.51670	-5.71470	3.55720
C	8.19070	-7.41000	1.88220
C	6.48170	-5.64390	1.27430
C	9.89210	-2.90160	2.80480
C	9.38980	-1.48830	3.15200
C	10.25070	-3.63330	4.11130
C	11.15390	-2.78960	1.91770
H	8.46740	-2.97870	1.33890
H	9.87370	-5.37280	1.97500
H	8.71700	-6.25120	-1.33870
H	11.53520	-6.01480	-0.53670
H	10.84920	-5.34640	-2.02810
H	13.65580	-8.90830	-3.04110
H	12.23250	-9.79450	-2.39920
H	12.10630	-8.90340	-3.94880
H	6.72010	-6.39550	3.88030
H	8.40730	-5.94010	4.15400
H	7.21150	-4.68950	3.77360
H	7.41640	-8.05300	2.31840
H	8.33310	-7.68790	0.83840
H	9.12990	-7.61420	2.41220
H	6.58090	-5.87280	0.20620
H	5.66280	-6.25880	1.66570
H	6.18660	-4.59270	1.37210
H	10.16980	-0.91820	3.66880
H	9.11180	-0.93510	2.24610
H	8.51140	-1.54240	3.80490
H	11.04930	-3.08930	4.62840
H	9.38610	-3.68950	4.77940
H	10.61390	-4.65120	3.93070
H	10.93360	-2.24610	0.99180
H	11.94830	-2.25060	2.44770
H	11.55100	-3.77670	1.65310

98. N1110_Anti-120WP

N	9.40860	-3.60550	2.16690
C	9.10500	-4.91340	1.58380
C	9.10110	-4.89500	0.04670
N	9.42510	-6.14940	-0.59890
C	10.62010	-6.11090	-1.47290
C	10.21000	-5.77850	-2.91390
O	11.10130	-5.00450	-3.54150
C	10.84030	-4.67340	-4.93520
O	9.18140	-6.18540	-3.43790
O	8.84760	-3.88120	-0.58830
C	7.72510	-5.47630	2.15300
C	7.89900	-5.67810	3.67140
C	7.34460	-6.83500	1.53030
C	6.59040	-4.46660	1.90180
C	11.43310	-7.46120	-1.37410
C	12.64940	-7.42430	-2.32250
C	11.94500	-7.59830	0.07460
C	10.54970	-8.67630	-1.71550
C	10.79710	-3.30270	2.60990
C	11.16570	-4.17370	3.82650
C	11.85430	-3.49540	1.49880
C	10.78800	-1.82610	3.04390
H	9.09250	-2.87410	1.53220
H	9.86660	-5.64050	1.87880
H	8.61870	-6.44790	-1.15370
H	11.28650	-5.31160	-1.13540
H	11.68270	-4.05420	-5.24350
H	10.79320	-5.58660	-5.53270
H	9.90220	-4.12040	-5.01690
H	8.66490	-6.43340	3.88640
H	8.18820	-4.74530	4.16080
H	6.95540	-6.02380	4.11020
H	6.48160	-7.24760	2.06600
H	7.05640	-6.74960	0.47720
H	8.16470	-7.55680	1.59970
H	6.42130	-4.30130	0.83150
H	5.65530	-4.84580	2.33040
H	6.81010	-3.50270	2.37060
H	13.27000	-8.31120	-2.15040
H	12.35510	-7.42860	-3.37780
H	13.27190	-6.53930	-2.14490
H	12.49390	-8.54000	0.18740
H	12.62880	-6.78150	0.33580
H	11.11540	-7.59730	0.78660
H	9.68790	-8.74390	-1.04350
H	10.18380	-8.63870	-2.74710
H	11.13530	-9.59610	-1.60210
H	12.16890	-3.91200	4.18160

H	10.45730	-4.01420	4.64560
H	11.17410	-5.24210	3.58460
H	12.84970	-3.21070	1.85940
H	11.91560	-4.54210	1.18120
H	11.62030	-2.87850	0.62330
H	10.05340	-1.66540	3.84030
H	11.77480	-1.52940	3.41590
H	10.53380	-1.17030	2.20170

99. N111'0_Anti-120WP

N	8.66480	-3.61260	2.11080
C	8.81290	-4.96520	1.55990
C	9.10150	-4.89670	0.04680
N	9.42410	-6.14510	-0.59850
C	10.73080	-6.39350	-1.27320
C	10.59980	-7.85920	-1.71520
O	11.60630	-8.62340	-1.27410
C	11.59920	-10.03030	-1.65130
O	9.67240	-8.28610	-2.38900
O	8.84830	-3.88370	-0.58890
C	7.55220	-5.88830	1.90480
C	7.24810	-5.73220	3.41080
C	7.84170	-7.38620	1.66490
C	6.31850	-5.44700	1.09480
C	11.22780	-5.47070	-2.45680
C	11.79920	-4.15630	-1.88080
C	12.38790	-6.19520	-3.18090
C	10.11090	-5.16600	-3.47100
C	9.81150	-3.00440	2.84940
C	11.11600	-2.96660	2.02020
C	9.38150	-1.56310	3.17760
C	10.06620	-3.75970	4.16620
H	8.42890	-2.98910	1.33980
H	9.67240	-5.47140	2.01990
H	8.70710	-6.25050	-1.32600
H	10.70350	-10.51650	-1.25860
H	11.63390	-10.12550	-2.73900
H	12.49780	-10.45060	-1.19840
H	6.38660	-6.35930	3.66960
H	8.09620	-6.06240	4.02250
H	7.02220	-4.69720	3.67360
H	7.01560	-7.98130	2.07310
H	7.95770	-7.63380	0.60990
H	8.76140	-7.69340	2.17990
H	6.45200	-5.61090	0.01840
H	5.43780	-6.02040	1.40710
H	6.09920	-4.38450	1.25290
H	12.22630	-3.55660	-2.69310
H	11.03730	-3.55070	-1.38910
H	12.60200	-4.35890	-1.16050
H	12.82360	-5.52240	-3.92790
H	13.18380	-6.48020	-2.48270
H	12.05280	-7.09540	-3.70880
H	9.70470	-6.08320	-3.91010
H	9.29220	-4.60750	-3.00950
H	10.51630	-4.55520	-4.28620
H	11.91560	-2.47520	2.58720
H	11.46720	-3.97450	1.76960

H	10.97080	-2.41050	1.08760
H	10.17150	-1.04070	3.72840
H	9.17840	-0.99500	2.26120
H	8.47360	-1.56230	3.79080
H	10.37850	-4.79590	3.99430
H	10.86910	-3.26560	4.72530
H	9.16760	-3.76960	4.79070
H	11.50790	-6.36570	-0.50140

100. N0010_Syn-60WP

N	8.89580	-3.56230	2.17150
C	9.03460	-4.90110	1.58170
C	9.09980	-4.89020	0.06420
N	9.42210	-6.13720	-0.60790
C	10.71070	-6.76600	-0.20820
C	11.84120	-5.75270	-0.44170
O	12.79000	-5.85030	0.50050
C	13.94630	-4.97350	0.38470
O	11.88480	-4.95170	-1.36120
O	8.85200	-3.89810	-0.59670
C	10.97560	-8.12430	-0.97640
C	12.09540	-8.88950	-0.23520
C	9.69440	-8.98640	-0.93490
C	11.39240	-7.90550	-2.44420
H	9.67300	-2.97160	1.86430
H	9.00310	-3.64510	3.18590
H	8.15670	-5.51770	1.83400
H	9.90090	-5.45450	1.96920
H	8.65650	-6.79170	-0.42210
H	10.72440	-7.02050	0.85950
H	14.50590	-5.21110	-0.52300
H	13.62340	-3.93030	0.36530
H	14.54640	-5.17580	1.27210
H	11.81270	-9.09900	0.80390
H	12.27560	-9.84760	-0.73580
H	13.03960	-8.33580	-0.22490
H	9.31400	-9.10450	0.08760
H	8.89690	-8.57530	-1.56410
H	9.92390	-9.98620	-1.32010
H	10.66340	-7.29680	-2.98830
H	12.37070	-7.41880	-2.52170
H	11.46790	-8.87650	-2.94700

101. N0100_Syn-60WP

N	8.43000	-6.11080	2.15600
C	8.86550	-4.81040	1.61120
C	9.10050	-4.89340	0.07700
N	9.41830	-6.12230	-0.61270
C	10.61260	-6.90130	-0.26920
C	11.93560	-6.34940	-0.79750
O	11.91770	-5.04530	-1.06560
C	13.16900	-4.42370	-1.48270
O	12.93470	-7.05220	-0.89780
O	8.85500	-3.90990	-0.60480
C	9.97700	-4.04340	2.44720
C	9.39670	-3.79950	3.85890
C	10.29500	-2.67080	1.81780
C	11.28480	-4.84810	2.58100
H	9.20910	-6.68220	2.48870
H	7.84000	-5.94810	2.97470
H	7.98650	-4.15580	1.65350
H	8.58730	-6.72100	-0.62770
H	10.49950	-7.89770	-0.70650
H	10.76970	-7.05730	0.80910
H	13.93270	-4.57080	-0.71480
H	13.49990	-4.85060	-2.43270
H	12.93550	-3.36510	-1.59660
H	9.19800	-4.73670	4.38850
H	10.11020	-3.22520	4.46080
H	8.46150	-3.22930	3.80960
H	9.38940	-2.06450	1.70320
H	10.98710	-2.12460	2.47030
H	10.76730	-2.76030	0.83520
H	11.80810	-4.93770	1.62480
H	11.96440	-4.33410	3.27060
H	11.11900	-5.85280	2.98700

102. N0110_Syn-60WP

N	9.73140	-3.57460	2.02260
C	9.05840	-4.82030	1.60580
C	9.09980	-4.89060	0.06970
N	9.42080	-6.13180	-0.61030
C	10.75900	-6.69430	-0.31680
C	11.78620	-5.98470	-1.21930
O	12.91550	-5.71070	-0.54940
C	14.00990	-5.10560	-1.29370
O	11.61750	-5.70970	-2.39600
O	8.85330	-3.90310	-0.60000
C	7.57210	-5.00910	2.14420
C	6.68580	-3.79340	1.81520
C	6.92790	-6.27730	1.54460
C	7.65210	-5.19010	3.67570
C	10.80280	-8.26130	-0.49960
C	12.23860	-8.76050	-0.22440
C	9.86390	-8.91140	0.54220
C	10.37290	-8.69680	-1.91470
H	9.63950	-3.43940	3.03120
H	9.28410	-2.76920	1.58140
H	9.63710	-5.65700	2.01470
H	8.69280	-6.81970	-0.41430
H	11.06560	-6.49910	0.71940
H	14.81670	-4.98780	-0.56960
H	14.31590	-5.76550	-2.10890
H	13.70050	-4.13580	-1.68950
H	5.67960	-3.95640	2.21830
H	7.07440	-2.87220	2.26220
H	6.59230	-3.63690	0.73570
H	5.94770	-6.44640	2.00500
H	6.75800	-6.18930	0.46380
H	7.53370	-7.17010	1.73690
H	6.64750	-5.34610	4.08500
H	8.26370	-6.06100	3.93970
H	8.07460	-4.31170	4.17610
H	12.25040	-9.85570	-0.25080
H	12.95210	-8.40580	-0.97570
H	12.59400	-8.44310	0.76310
H	9.98350	-9.99970	0.50640
H	10.10070	-8.58190	1.56090
H	8.80500	-8.70740	0.34650
H	9.35040	-8.38280	-2.15030
H	11.03990	-8.29340	-2.68230
H	10.40660	-9.79040	-1.97980

103. N011'0_Syn-60WP

N	8.59160	-6.13220	2.18950
C	8.88600	-4.81080	1.60910
C	9.10080	-4.89450	0.07270
N	9.41860	-6.12330	-0.61050
C	10.68630	-6.82330	-0.31780
C	10.37020	-8.32140	-0.23670
O	11.32260	-9.01560	0.39050
C	11.16850	-10.46240	0.45430
O	9.35710	-8.82880	-0.69950
O	8.85450	-3.90760	-0.60270
C	9.93810	-3.92230	2.40570
C	9.34820	-3.68100	3.81310
C	10.14270	-2.54970	1.72990
C	11.31150	-4.60880	2.55810
C	11.81950	-6.50570	-1.39020
C	13.19180	-7.01550	-0.89830
C	11.92300	-4.97710	-1.57290
C	11.48580	-7.14510	-2.75050
H	8.01680	-6.01350	3.02740
H	9.43250	-6.62010	2.49970
H	7.94890	-4.24300	1.65880
H	8.62750	-6.76360	-0.50800
H	12.04530	-10.81530	0.99630
H	10.25130	-10.71660	0.98950
H	11.14490	-10.87900	-0.55550
H	10.01050	-3.02330	4.38780
H	8.36440	-3.19970	3.75250
H	9.24040	-4.61340	4.37580
H	10.77420	-1.92100	2.36950
H	10.63780	-2.63250	0.75910
H	9.18960	-2.03140	1.57980
H	11.83850	-4.69800	1.60170
H	11.94980	-4.00730	3.21620
H	11.23720	-5.60600	3.00990
H	13.96530	-6.69520	-1.60590
H	13.44930	-6.59940	0.08350
H	13.23450	-8.10530	-0.82450
H	12.73720	-4.75380	-2.27190
H	11.00240	-4.55320	-1.97960
H	12.15610	-4.46700	-0.63030
H	12.22780	-6.83550	-3.49540
H	11.50310	-8.23980	-2.70670
H	10.50030	-6.82830	-3.10420
H	11.09560	-6.53390	0.65780

104. N1000_Syn-60WP

N	8.70230	-6.06730	2.21800
C	9.13750	-4.82200	1.59040
C	9.10040	-4.89290	0.06590
N	9.42080	-6.13200	-0.60790
C	10.78050	-6.62490	-0.29400
C	11.02520	-7.94260	-1.01130
O	12.33180	-8.22130	-1.08770
C	12.71570	-9.48650	-1.70320
O	10.14550	-8.67620	-1.43760
O	8.85260	-3.90060	-0.59840
C	8.98470	-6.23710	3.67260
C	8.34060	-7.57400	4.07760
C	8.38420	-5.10040	4.53050
C	10.50420	-6.32000	3.90750
H	7.69490	-6.17310	2.07650
H	10.19020	-4.62370	1.84110
H	8.57320	-3.92280	1.87930
H	8.76400	-6.83710	-0.24900
H	10.93960	-6.80740	0.78060
H	11.52870	-5.89350	-0.62380
H	12.25660	-10.31970	-1.16490
H	12.41030	-9.49750	-2.75410
H	13.80190	-9.52500	-1.61910
H	7.25710	-7.55210	3.91130
H	8.76360	-8.39760	3.49260
H	8.51450	-7.77620	5.14080
H	8.54520	-5.30080	5.59640
H	8.84450	-4.13190	4.30370
H	7.30350	-5.01670	4.36180
H	10.70090	-6.53360	4.96440
H	10.94830	-7.12240	3.30850
H	11.01580	-5.38010	3.67020

105. N1010_Syn-60WP

N	8.17350	-5.93790	2.09960
C	9.02550	-4.85700	1.59450
C	9.10060	-4.89340	0.06520
N	9.42080	-6.13190	-0.60750
C	10.66440	-6.79840	-0.14920
C	11.86420	-5.90310	-0.49010
O	12.79920	-5.96210	0.46860
C	14.03150	-5.21940	0.26070
O	11.97360	-5.22500	-1.49880
O	8.85250	-3.90020	-0.59820
C	10.80600	-8.23900	-0.78150
C	9.58100	-9.08150	-0.36120
C	10.89250	-8.19200	-2.31780
C	12.07120	-8.92410	-0.21910
C	8.33200	-6.31460	3.53720
C	7.18710	-7.29440	3.84890
C	9.67820	-7.03340	3.74640
C	8.24030	-5.09740	4.48440
H	7.19750	-5.67420	1.94450
H	10.04670	-4.98090	1.97910
H	8.70430	-3.84450	1.87400
H	8.63340	-6.76030	-0.43210
H	10.67350	-6.94310	0.93740
H	14.52600	-5.56050	-0.65250
H	13.81580	-4.15000	0.19610
H	14.64610	-5.43690	1.13510
H	9.71500	-10.11260	-0.70680
H	9.46390	-9.10600	0.72700
H	8.64620	-8.71850	-0.80200
H	10.93530	-9.21450	-2.70920
H	10.02130	-7.69670	-2.75660
H	11.79080	-7.66700	-2.65690
H	12.99110	-8.41920	-0.53040
H	12.05800	-8.96230	0.87660
H	12.11790	-9.95430	-0.59040
H	7.24940	-7.63660	4.88780
H	6.21040	-6.81300	3.70940
H	7.23750	-8.17020	3.19220
H	9.76720	-7.35890	4.78900
H	9.74850	-7.91910	3.10610
H	10.53640	-6.38270	3.54070
H	7.29400	-4.56160	4.34070
H	8.29020	-5.42440	5.52920
H	9.06380	-4.39250	4.32140

106. N1100_Syn-60WP

N	9.23740	-3.48550	2.13280
C	8.99830	-4.83280	1.61730
C	9.09970	-4.89020	0.07610
N	9.41970	-6.12760	-0.61320
C	10.72480	-6.75150	-0.34060
C	10.70780	-8.17150	-0.88890
O	11.93740	-8.67690	-1.00310
C	12.05240	-10.05660	-1.46040
O	9.68570	-8.78310	-1.16030
O	8.85450	-3.90760	-0.60340
C	7.59180	-5.37300	2.14650
C	6.45810	-4.39550	1.78410
C	7.70300	-5.48190	3.68160
C	7.24740	-6.77180	1.59490
C	10.60770	-3.05510	2.51760
C	11.63310	-3.16030	1.36550
C	10.47420	-1.57860	2.93720
C	11.09740	-3.87240	3.72770
H	8.85060	-2.80770	1.47820
H	9.74380	-5.52080	2.03030
H	8.68650	-6.81640	-0.43830
H	10.97620	-6.83900	0.72970
H	11.53070	-6.18790	-0.82270
H	11.56050	-10.72470	-0.75030
H	11.60250	-10.15700	-2.44970
H	13.12320	-10.25430	-1.50070
H	5.50670	-4.77070	2.17940
H	6.63400	-3.40630	2.21710
H	6.34590	-4.28450	0.69940
H	6.74280	-5.80150	4.10360
H	8.46100	-6.22010	3.97100
H	7.97420	-4.51960	4.12130
H	8.07380	-7.48000	1.73180
H	6.38390	-7.17300	2.13780
H	6.96050	-6.74980	0.53630
H	12.61010	-2.77840	1.68410
H	11.78050	-4.19930	1.04840
H	11.30620	-2.57780	0.49670
H	11.44410	-1.17810	3.25160
H	10.11080	-0.96480	2.10320
H	9.76920	-1.47840	3.76940
H	10.39300	-3.78540	4.56160
H	11.21960	-4.93520	3.48960
H	12.07280	-3.49800	4.05860

107. N1110_Syn-60WP

N	8.44740	-6.05280	2.19810
C	8.89920	-4.78590	1.62950
C	9.09990	-4.89110	0.08030
N	9.41890	-6.12430	-0.61480
C	10.64810	-6.84580	-0.23590
C	11.83870	-6.15320	-0.92630
O	12.91990	-6.13420	-0.13410
C	14.14840	-5.57560	-0.67460
O	11.82410	-5.68160	-2.05290
O	8.85510	-3.91010	-0.60600
C	10.07100	-4.09080	2.45150
C	11.22920	-5.03860	2.82910
C	9.45720	-3.54960	3.76480
C	10.64540	-2.89190	1.66950
C	10.57900	-8.37800	-0.61910
C	9.46800	-9.05160	0.21490
C	10.29410	-8.59270	-2.11810
C	11.91770	-9.05540	-0.25240
C	6.99130	-6.26530	2.44700
C	6.22060	-6.23160	1.11480
C	6.86550	-7.66350	3.07720
C	6.37330	-5.23000	3.41620
H	8.96050	-6.28570	3.04290
H	8.09440	-4.03990	1.66630
H	8.61360	-6.74240	-0.50500
H	10.82750	-6.81440	0.84300
H	14.87490	-5.64930	0.13540
H	14.47490	-6.15770	-1.54030
H	13.99290	-4.53310	-0.96110
H	11.91950	-4.50320	3.49150
H	10.89360	-5.92730	3.37680
H	11.80730	-5.36530	1.96230
H	10.23060	-3.03060	4.34230
H	8.64900	-2.83650	3.56720
H	9.05920	-4.35080	4.39550
H	9.86110	-2.17900	1.39430
H	11.37490	-2.36800	2.29750
H	11.15950	-3.19630	0.75210
H	9.48360	-10.13130	0.02930
H	9.61390	-8.88450	1.28620
H	8.46530	-8.69980	-0.04930
H	10.24390	-9.66780	-2.32510
H	9.33990	-8.15060	-2.42060
H	11.08210	-8.16690	-2.74570
H	12.74700	-8.70040	-0.87180
H	12.18020	-8.88610	0.79810
H	11.82910	-10.13640	-0.40730
H	5.15730	-6.42430	1.29360

H	6.29350	-5.25570	0.62100
H	6.58620	-7.00110	0.42620
H	5.81610	-7.89380	3.29100
H	7.26120	-8.43140	2.40580
H	7.41980	-7.71500	4.02300
H	6.44310	-4.20750	3.02980
H	5.31040	-5.44780	3.57140
H	6.87010	-5.26310	4.39200

108. N111'0_Syn-60WP

N	9.31220	-3.52350	2.16350
C	8.99280	-4.83930	1.61090
C	9.10020	-4.89190	0.06920
N	9.42050	-6.13080	-0.60970
C	10.76770	-6.70270	-0.39160
C	10.61960	-8.11830	0.18030
O	11.64420	-8.47010	0.96210
C	11.64740	-9.82440	1.50170
O	9.66440	-8.84670	-0.05160
O	8.85320	-3.90280	-0.60000
C	7.53940	-5.28800	2.10020
C	7.07990	-6.61640	1.46610
C	6.49870	-4.19880	1.77950
C	7.61820	-5.48740	3.62750
C	11.64490	-6.67180	-1.70920
C	13.07240	-7.18010	-1.41800
C	11.00300	-7.52620	-2.81700
C	11.73920	-5.20670	-2.18180
C	10.68530	-3.17310	2.61540
C	11.12980	-4.10630	3.75640
C	11.73830	-3.19630	1.48440
C	10.58130	-1.73640	3.16310
H	8.97820	-2.80260	1.52670
H	9.68660	-5.58140	2.01850
H	8.69970	-6.83890	-0.47410
H	10.77710	-9.97240	2.14510
H	11.64020	-10.55050	0.68570
H	12.57060	-9.89830	2.07680
H	6.15190	-6.94400	1.94880
H	6.85650	-6.51840	0.39660
H	7.81600	-7.41670	1.60360
H	5.51430	-4.51450	2.14410
H	6.75050	-3.25330	2.26790
H	6.41240	-4.02180	0.70160
H	7.96540	-4.57680	4.12160
H	6.62750	-5.74270	4.02170
H	8.30260	-6.30510	3.88720
H	13.69660	-7.02840	-2.30620
H	13.53370	-6.63070	-0.58810
H	13.09680	-8.24650	-1.17410
H	11.58520	-7.42950	-3.74030
H	10.97640	-8.58890	-2.55090
H	9.97990	-7.19870	-3.02750
H	10.75250	-4.78720	-2.39310
H	12.23020	-4.57400	-1.43270
H	12.33610	-5.15690	-3.09940
H	12.09970	-3.77610	4.14490
H	10.40540	-4.08870	4.57710

H	11.25280	-5.14390	3.42560
H	12.70960	-2.84680	1.85420
H	11.89120	-4.20790	1.09440
H	11.43520	-2.54810	0.65430
H	9.86500	-1.69190	3.99010
H	11.55620	-1.39110	3.52500
H	10.24720	-1.04280	2.38130
H	11.31290	-6.11520	0.35530

109. N0000_Syn60WP

N	8.68940	-3.67040	2.21040
C	9.25600	-4.86710	1.57310
C	9.09970	-4.88990	0.06310
N	9.42250	-6.13870	-0.60760
C	8.58610	-7.32170	-0.30430
C	7.09710	-7.02620	-0.30350
O	6.38410	-8.15480	-0.19690
C	4.94190	-8.02470	-0.03570
O	6.60270	-5.91070	-0.33890
O	8.85170	-3.89680	-0.59610
H	8.81110	-3.75500	3.22300
H	7.67740	-3.65190	2.06110
H	8.86250	-5.80870	1.98120
H	10.34390	-4.89950	1.74270
H	10.40300	-6.37040	-0.41360
H	8.80750	-7.77760	0.67390
H	8.78670	-8.09100	-1.05870
H	4.58030	-9.04290	0.10830
H	4.72040	-7.40970	0.83920
H	4.50120	-7.58070	-0.93080

110. N0010_Syn60WP

N	9.43720	-5.86300	2.44560
C	9.19510	-4.69300	1.59450
C	9.10120	-4.89600	0.07430
N	9.41820	-6.12180	-0.61080
C	8.69850	-7.39030	-0.32470
C	9.40950	-8.46430	-1.16280
O	9.50240	-9.62680	-0.50380
C	10.06260	-10.75910	-1.22590
O	9.83530	-8.30080	-2.29620
O	8.85450	-3.90770	-0.60400
C	7.14890	-7.35350	-0.65890
C	6.56320	-8.77590	-0.49020
C	6.41450	-6.43940	0.34930
C	6.86720	-6.86260	-2.09170
H	8.55490	-6.27650	2.75140
H	9.89040	-5.54840	3.30550
H	10.04400	-4.00360	1.69680
H	8.30750	-4.10950	1.87630
H	10.43130	-6.26220	-0.52330
H	8.79000	-7.69420	0.72380
H	9.44960	-10.98030	-2.10340
H	11.08840	-10.53950	-1.53010
H	10.03680	-11.58920	-0.51970
H	6.78850	-9.19360	0.49790
H	5.47340	-8.72880	-0.59380
H	6.93370	-9.47250	-1.24950
H	6.59770	-6.74760	1.38590
H	6.67110	-5.38150	0.24110
H	5.33470	-6.51260	0.17520
H	7.23570	-5.84590	-2.25560
H	7.32400	-7.51800	-2.84020
H	5.78410	-6.86200	-2.26410

111. N0100_Syn60WP

N	10.28390	-3.86070	1.87320
C	9.15560	-4.79030	1.61480
C	9.09950	-4.88940	0.07340
N	9.42040	-6.13020	-0.61220
C	8.53110	-7.27410	-0.34480
C	8.50340	-8.25200	-1.51750
O	7.58620	-9.20600	-1.29610
C	7.38780	-10.21670	-2.32540
O	9.19630	-8.19330	-2.51930
O	8.85400	-3.90580	-0.60170
C	7.79500	-4.42070	2.32830
C	8.06540	-4.43000	3.84990
C	6.72310	-5.48700	2.02300
C	7.24880	-3.03730	1.92400
H	10.03960	-2.91540	1.56940
H	10.47390	-3.80460	2.87510
H	9.45880	-5.77010	2.00420
H	10.38210	-6.38210	-0.35040
H	7.50070	-6.92600	-0.22720
H	8.78550	-7.84420	0.55980
H	6.99900	-9.74930	-3.23340
H	8.32710	-10.73200	-2.53540
H	6.65420	-10.90730	-1.90800
H	8.74890	-3.62860	4.15060
H	7.12720	-4.27950	4.39540
H	8.49450	-5.38640	4.17190
H	7.07890	-6.49600	2.26040
H	5.83040	-5.29950	2.63120
H	6.40560	-5.46110	0.97380
H	6.94650	-3.00920	0.87350
H	6.36710	-2.80710	2.53420
H	7.98020	-2.23880	2.09110

112. N0110_Syn60WP

N	10.57690	-4.17890	1.79730
C	9.24890	-4.80750	1.60670
C	9.10010	-4.89180	0.06710
N	9.42060	-6.13120	-0.60880
C	8.56090	-7.30570	-0.32060
C	9.49230	-8.49270	-0.03370
O	8.86690	-9.51620	0.54610
C	9.64260	-10.72170	0.80470
O	10.68510	-8.49760	-0.30740
O	8.85310	-3.90250	-0.59880
C	8.04330	-4.15230	2.37760
C	7.83180	-2.66410	2.03280
C	6.74770	-4.93160	2.06760
C	8.33610	-4.28110	3.88850
C	7.52560	-7.60690	-1.48690
C	6.47530	-8.64380	-1.02910
C	6.77240	-6.30720	-1.83740
C	8.25000	-8.12190	-2.74440
H	10.86680	-4.25220	2.77400
H	10.54260	-3.18290	1.57160
H	9.34000	-5.83170	1.98900
H	10.39670	-6.36580	-0.40030
H	7.96870	-7.14890	0.59110
H	8.93820	-11.41840	1.25900
H	10.03070	-11.12220	-0.13430
H	10.46360	-10.49680	1.48870
H	7.04270	-2.25170	2.67320
H	8.73720	-2.07100	2.20760
H	7.53140	-2.52740	0.99120
H	5.91990	-4.54000	2.67080
H	6.45360	-4.83450	1.01680
H	6.85040	-5.99760	2.30790
H	7.48590	-3.90420	4.46810
H	8.50100	-5.32750	4.17280
H	9.21760	-3.70180	4.18500
H	5.70350	-8.73460	-1.80250
H	6.90400	-9.63510	-0.86620
H	5.97970	-8.32990	-0.10180
H	6.04600	-6.51710	-2.63060
H	6.21350	-5.91920	-0.97780
H	7.44730	-5.52600	-2.19340
H	9.01560	-7.41470	-3.07940
H	8.72740	-9.09430	-2.57450
H	7.52840	-8.24940	-3.55940

113. N011'0_Syn60WP

N	10.47560	-3.94890	1.78440
C	9.26230	-4.78170	1.60790
C	9.09980	-4.89050	0.07100
N	9.42040	-6.13020	-0.61090
C	8.71010	-7.35760	-0.19660
C	7.26040	-7.29030	-0.71150
O	6.45360	-8.08450	0.00350
C	5.06060	-8.18860	-0.40200
O	6.87870	-6.61550	-1.65440
O	8.85370	-3.90480	-0.60060
C	7.97730	-4.31240	2.38880
C	8.30030	-4.37820	3.89740
C	6.81340	-5.28150	2.09720
C	7.53860	-2.88000	2.02650
C	9.44270	-8.66450	-0.72090
C	8.92220	-9.89220	0.06080
C	10.95920	-8.54680	-0.44840
C	9.21030	-8.88070	-2.23070
H	10.75350	-3.94010	2.76750
H	10.28750	-2.97900	1.52330
H	9.51560	-5.77530	1.99740
H	10.43160	-6.25200	-0.51560
H	4.60960	-8.88210	0.30850
H	4.57710	-7.21050	-0.34450
H	4.99350	-8.58220	-1.41900
H	7.40600	-4.13490	4.48210
H	8.63070	-5.38280	4.18820
H	9.08220	-3.66530	4.18200
H	5.94220	-5.01740	2.70780
H	6.49470	-5.23170	1.04920
H	7.07970	-6.31740	2.33580
H	7.21760	-2.80530	0.98410
H	6.69420	-2.58850	2.66240
H	8.34010	-2.15070	2.19050
H	9.46120	-10.78550	-0.27440
H	9.09440	-9.77900	1.13880
H	7.85440	-10.06470	-0.09720
H	11.43420	-9.51020	-0.66270
H	11.45040	-7.80650	-1.09120
H	11.16580	-8.29970	0.60050
H	9.80790	-9.73250	-2.57370
H	8.16080	-9.10750	-2.45250
H	9.50200	-8.00330	-2.81770
H	8.65610	-7.45530	0.89720

114. N1010_Syn60WP

N	7.67970	-4.98440	1.99850
C	9.08620	-4.83010	1.59140
C	9.10060	-4.89330	0.06090
N	9.42190	-6.13640	-0.60560
C	8.44620	-7.27090	-0.47690
C	9.26950	-8.54840	-0.68220
O	8.82720	-9.55950	0.07210
C	9.47590	-10.85260	-0.09780
O	10.22210	-8.64810	-1.44280
O	8.85140	-3.89580	-0.59580
C	7.21770	-7.20600	-1.47590
C	6.34630	-5.97250	-1.15750
C	7.67030	-7.13720	-2.94540
C	6.33340	-8.45920	-1.27040
C	7.40200	-5.23400	3.44240
C	5.87210	-5.15960	3.59740
C	7.87640	-6.64660	3.82720
C	8.06160	-4.18260	4.36030
H	7.17240	-4.14620	1.71360
H	9.68350	-5.64730	2.00590
H	9.54490	-3.87820	1.89680
H	10.29630	-6.45450	-0.17200
H	8.02710	-7.31480	0.53510
H	9.36010	-11.19620	-1.12680
H	10.53520	-10.77570	0.15330
H	8.96230	-11.52270	0.59170
H	5.42190	-6.03190	-1.74310
H	6.08150	-5.93090	-0.09590
H	6.84410	-5.03730	-1.42280
H	6.78920	-7.06320	-3.59240
H	8.29800	-6.25880	-3.12710
H	8.22900	-8.02770	-3.24550
H	6.83020	-9.38260	-1.58910
H	6.03500	-8.57850	-0.22270
H	5.42260	-8.35310	-1.86740
H	5.58340	-5.35280	4.63640
H	5.49830	-4.16580	3.32240
H	5.38160	-5.90160	2.95650
H	7.60990	-6.86200	4.86890
H	7.39950	-7.39850	3.19090
H	8.96500	-6.75440	3.73810
H	7.74140	-3.17230	4.08790
H	7.77950	-4.35230	5.40560
H	9.15490	-4.22330	4.30020

115. N1100_Syn60WP

N	10.28870	-3.85040	1.98440
C	9.19820	-4.76660	1.62290
C	9.09930	-4.88900	0.07970
N	9.42000	-6.12860	-0.61510
C	8.57750	-7.30910	-0.39750
C	9.07520	-8.28300	0.66960
O	8.17560	-9.24790	0.90400
C	8.54880	-10.31030	1.82920
O	10.16870	-8.22440	1.20800
O	8.85460	-3.90800	-0.60500
C	7.84320	-4.31780	2.31040
C	7.55360	-2.81770	2.10280
C	7.98440	-4.58490	3.82400
C	6.65450	-5.14180	1.77590
C	11.66170	-4.31740	2.30590
C	12.37460	-5.00140	1.11800
C	12.44300	-3.04780	2.69740
C	11.63350	-5.27340	3.51260
H	10.32400	-3.05810	1.34610
H	9.43930	-5.75680	2.01510
H	10.40980	-6.35860	-0.51210
H	8.52700	-7.88950	-1.32970
H	7.54750	-7.01620	-0.17850
H	9.42910	-10.83700	1.45480
H	8.74970	-9.89230	2.81760
H	7.68640	-10.97600	1.86070
H	6.63620	-2.54630	2.64010
H	8.36700	-2.20230	2.50040
H	7.41310	-2.56980	1.04680
H	7.07850	-4.25930	4.35000
H	8.12720	-5.65310	4.03190
H	8.83620	-4.03800	4.23910
H	6.80660	-6.21790	1.92510
H	5.74130	-4.86800	2.31750
H	6.46390	-4.95620	0.71180
H	13.42460	-5.20470	1.35920
H	11.90790	-5.96210	0.87750
H	12.34830	-4.35930	0.22970
H	13.47370	-3.30250	2.96700
H	12.47900	-2.33540	1.86390
H	11.97070	-2.55420	3.55300
H	11.17010	-4.79120	4.37960
H	11.08860	-6.19880	3.29780
H	12.65810	-5.55520	3.78040

116. N1110_Syn60WP

N	8.25610	-5.71330	2.28160
C	9.12790	-4.73670	1.62110
C	9.10080	-4.89440	0.07470
N	9.41880	-6.12420	-0.61140
C	8.55340	-7.31570	-0.44680
C	9.42860	-8.49150	-0.00430
O	8.74350	-9.42230	0.66450
C	9.46070	-10.61650	1.08540
O	10.62800	-8.57710	-0.23890
O	8.85430	-3.90690	-0.60380
C	10.60270	-4.69280	2.19210
C	11.47490	-3.78760	1.29660
C	11.26480	-6.08250	2.30300
C	10.55080	-4.06410	3.60270
C	7.74090	-7.63970	-1.76760
C	6.94190	-6.37970	-2.16130
C	8.68430	-8.02000	-2.92250
C	6.73570	-8.78370	-1.51680
C	6.92590	-5.27640	2.79970
C	6.05330	-4.75180	1.64570
C	6.27230	-6.53440	3.39990
C	7.02020	-4.19180	3.89750
H	8.74850	-6.20860	3.01960
H	8.74120	-3.72100	1.76710
H	10.40310	-6.36970	-0.49560
H	7.83490	-7.13810	0.35540
H	8.71370	-11.23520	1.58470
H	9.87210	-11.13300	0.21540
H	10.26320	-10.34840	1.77670
H	12.46280	-3.65950	1.75250
H	11.62990	-4.21200	0.29780
H	11.02610	-2.79510	1.17650
H	12.25490	-5.96760	2.75870
H	10.69920	-6.77160	2.94110
H	11.40950	-6.56370	1.33220
H	9.95850	-4.66500	4.30030
H	11.56630	-3.99190	4.00920
H	10.12560	-3.05410	3.57500
H	6.35680	-6.58370	-3.06470
H	6.23860	-6.08780	-1.37250
H	7.59990	-5.53200	-2.36470
H	8.10800	-8.15170	-3.84510
H	9.42700	-7.23520	-3.09910
H	9.21470	-8.95890	-2.72540
H	7.22780	-9.74130	-1.32390
H	6.08040	-8.56200	-0.66570
H	6.10120	-8.90580	-2.40250
H	5.06170	-4.48100	2.02440

H	6.48080	-3.85770	1.17900
H	5.91960	-5.51600	0.87390
H	5.28390	-6.29460	3.80750
H	6.15540	-7.31300	2.63920
H	6.88290	-6.94100	4.21620
H	7.48700	-3.27220	3.52790
H	6.01690	-3.92990	4.25380
H	7.59990	-4.55170	4.75480

117. N111'0_Syn60WP

N	10.21000	-3.79510	1.97670
C	9.17130	-4.77890	1.62360
C	9.09910	-4.88780	0.07710
N	9.42080	-6.13200	-0.61430
C	8.52370	-7.28860	-0.38620
C	7.44650	-7.27870	-1.48930
O	6.23710	-7.56210	-0.98840
C	5.12080	-7.64050	-1.91670
O	7.65230	-7.05320	-2.67150
O	8.85400	-3.90570	-0.60330
C	7.80240	-4.40690	2.33040
C	6.67390	-5.37470	1.92690
C	7.36310	-2.96460	2.01040
C	8.02420	-4.53360	3.85270
C	9.29280	-8.66420	-0.33150
C	8.26880	-9.82010	-0.28170
C	10.21960	-8.85970	-1.54740
C	10.12390	-8.71590	0.97010
C	11.61180	-4.18360	2.27740
C	11.66260	-5.04040	3.55610
C	12.31690	-4.93180	1.12300
C	12.35450	-2.85850	2.53470
H	10.19710	-3.01930	1.31790
H	9.47000	-5.75540	2.01630
H	10.37940	-6.38080	-0.37070
H	4.98750	-6.68140	-2.42240
H	5.29840	-8.43130	-2.64960
H	4.25290	-7.87510	-1.29940
H	5.75350	-5.10530	2.45860
H	6.44920	-5.33580	0.85510
H	6.90870	-6.41100	2.19940
H	6.46090	-2.72530	2.58600
H	8.13780	-2.24380	2.28920
H	7.13050	-2.83410	0.94960
H	8.81250	-3.85740	4.19260
H	7.09940	-4.28220	4.38530
H	8.30330	-5.55700	4.13340
H	8.79970	-10.76560	-0.12490
H	7.55610	-9.69300	0.54110
H	7.70210	-9.91170	-1.21440
H	10.72010	-9.83130	-1.46660
H	9.65850	-8.84490	-2.48650
H	11.00090	-8.09360	-1.60580
H	10.91070	-7.95470	1.00480
H	9.48910	-8.59580	1.85610
H	10.62290	-9.68810	1.04720
H	12.70330	-5.28280	3.79920
H	11.22670	-4.49920	4.40170

H	11.12590	-5.98880	3.44300
H	13.38300	-5.06490	1.34080
H	11.89990	-5.93520	0.98260
H	12.22830	-4.37670	0.18170
H	11.88180	-2.30610	3.35370
H	13.39900	-3.05210	2.80110
H	12.34550	-2.22400	1.63950
H	7.99270	-7.20430	0.56660
